# Isocyanides inhibit bacterial pathogens by covalent targeting of essential metabolic enzymes†

**DOI:** 10.1039/d4sc01940g

**Published:** 2024-06-26

**Authors:** Alexandra Geißler, Howard Junca, Andreas M. Kany, Lena J. Daumann, Anna K. H. Hirsch, Dietmar H. Pieper, Stephan A. Sieber

**Affiliations:** a Center for Functional Protein Assemblies, Department of Bioscience, TUM School of Natural Sciences, Technical University of Munich Ernst-Otto-Fischer-Straße 8 85748 Garching Germany stephan.sieber@tum.de; b Microbial Interactions and Processes Research Group, Helmholtz Centre for Infection Research Inhoffenstraße 7 38124 Braunschweig Germany; c Helmholtz Institute for Pharmaceutical Research Saarland (HIPS) – Helmholtz Centre for Infection Research (HZI) Campus E8.1 66123 Saarbrücken Germany; d Deutsches Zentrum für Infektionsforschung (DZIF) e.V. 38124 Braunschweig Germany; e Chair of Bioinorganic Chemistry, Heinrich-Heine-Universität Düsseldorf Universitätsstraße 1 40225 Düsseldorf Germany; f Saarland University, Department of Pharmacy 66123 Saarbrücken Germany

## Abstract

Isonitrile natural products, also known as isocyanides, demonstrate potent antimicrobial activities, yet our understanding of their molecular targets remains limited. Here, we focus on the so far neglected group of monoisonitriles to gain further insights into their antimicrobial mode of action (MoA). Screening a focused monoisonitrile library revealed a potent *S. aureus* growth inhibitor with a different MoA compared to previously described isonitrile antibiotics. Chemical proteomics *via* competitive cysteine reactivity profiling, uncovered covalent modifications of two essential metabolic enzymes involved in the fatty acid biosynthetic process (FabF) and the hexosamine pathway (GlmS) at their active site cysteines. In-depth studies with the recombinant enzymes demonstrated concentration-dependent labeling, covalent binding to the catalytic site and corresponding functional inhibition by the isocyanide. Thermal proteome profiling and full proteome studies of compound-treated *S. aureus* further highlighted the destabilization and dysregulation of proteins related to the targeted pathways. Cytotoxicity and the inhibition of cytochrome P450 enzymes require optimization of the hit molecule prior to therapeutic application. The here described novel, covalent isocyanide MoA highlights the versatility of the functional group, making it a useful tool and out-of-the-box starting point for the development of innovative antibiotics.

## Abstract 2

Isocyanides inhibit the growth of bacterial pathogens by covalently targeting essential metabolic enzymes at their active site cysteines.

## Graphical abstract

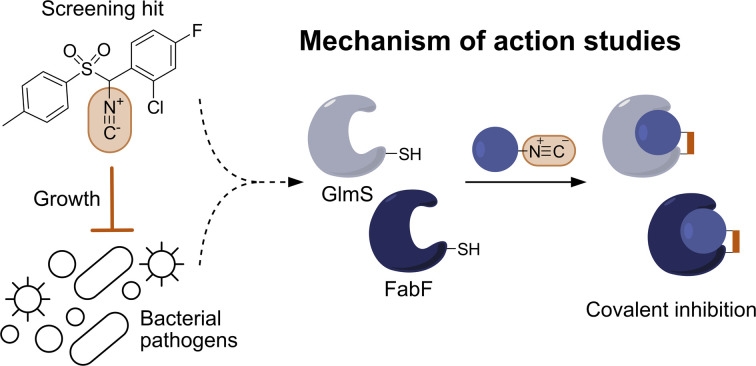

## Introduction

**Fig. 1:**
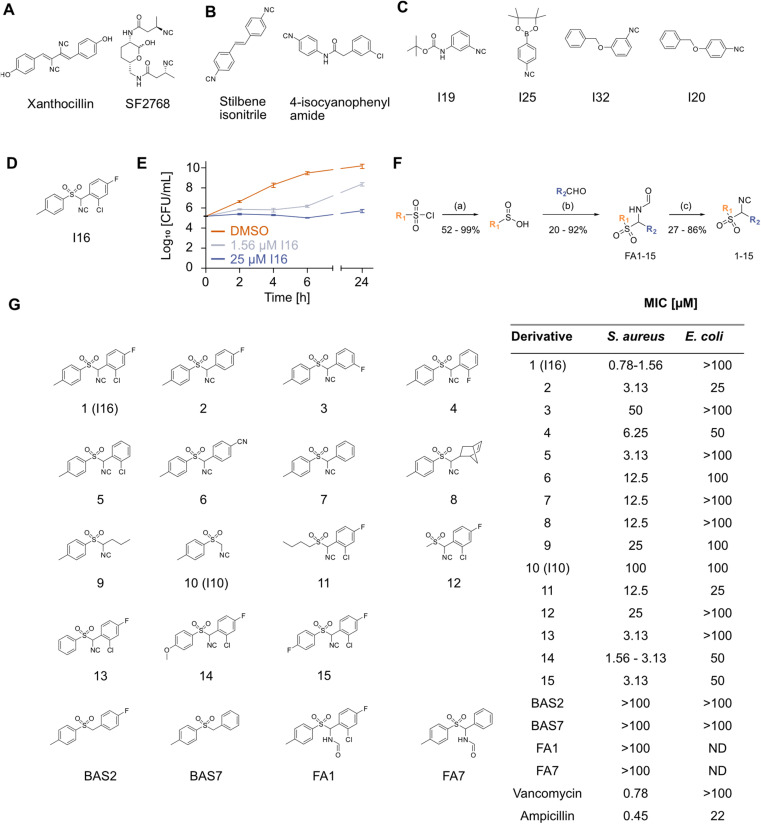
(A) Structures of antibiotic natural diisonitriles xanthocillin (Xan) and SF2768. (B) Structures of antibiotic synthetic aryl isocyanides (*E*)-1,2-bis(4-isocyanophenyl)ethane (stilbene isonitrile) and 2-(3-chlorophenyl)-*N*-(4-isocyanophenyl)acetamide (4-isocyanophenyl amide). (C) Structures of active antibiotic isocyanides identified in the isocyanide screening. (D) Structure of the screening hit molecule I16. (E) Growth of *S. aureus* NCTC 8325 in the presence of DMSO (1%) or I16 (1.56 μM or 25 μM). After 0, 2, 4, 6 and 24 h viable cells (CFU mL^−1^) were determined in quadruplicates (0 h) and triplicates (2–24 h). Data represent mean values ± SEM of *n* = 3 independent experiments. (F) General synthesis scheme for generating I16 derivatives. Reagents and conditions: (a) Na_2_SO_3_, NaHCO_3_, water, 80 °C; HCl, water, r.t.; (b) formamide, TMSCl, MeCN, toluene, 50 °C; (c) POCl_3_, NEt_3_, −40/0 °C. (G) Structures and antibiotic activities (minimum inhibitory concentration, MIC) of I16 derivates (1–15), controls (BAS2, BAS7, FA1, and FA7), and reference antibiotics (vancomycin, ampicillin) against *S. aureus* NCTC 8325 and *E. coli* K12. MICs were determined in technical triplicates and confirmed in *n* = 3 independent experiments. The MIC is reported as mean of technical triplicates; if the MIC varied between biological replicates, concentration ranges are indicated. ND = not determined.

In light of the rising number of multidrug resistant bacteria, deciphering novel antibiotics is an urgent task. Given the lack of novel chemical entities with antibacterial activity, a compelling strategy is to find privileged antibiotic scaffolds and exploit these for undiscovered mechanisms of action (MoA). Among those is the isocyanide (isonitrile) moiety, which was first discovered in nature within the fungal metabolite xanthocillin (Xan) in the 1940s.^1–4^ To date, over 200 structurally diverse natural isocyanides have been identified, many demonstrating potent antibacterial behavior.^3,5^ The core scaffolds are diverse (*e.g.*, terpenes, terpenoids, indole alkaloids, lipopeptides, cyclopentanes, and many more) and often, not only one but two isocyanide moieties are present.^6,7^ The isocyanide is often attached to vinyl groups as well as secondary and tertiary carbons, which provide enhanced stability.^8^ Strikingly, some of these compounds exhibit strong antibacterial effects even against Gram-negative bacteria, which are challenging to address due to their largely impermeable double membrane layer.^1,9–11^ Despite these intriguing properties, the systematic exploitation of isocyanides is still in its infancy. Research has focused on the MoA analysis of a few natural products and only recently on structure–activity relationship (SAR) studies of synthetic compounds in medicinal chemistry.^5,12–19^ Among the studied natural products are SF2768 (ref. 20) and Xan, both bearing a diisonitrile moiety (Fig. 1A). The isocyano group has strong coordination properties with the lone electron pair at the isonitrile terminal carbon serving as a σ donor.^5^ In case of SF2768, which is produced by a nonribosomal peptide synthetase in *Streptomyces thioluteus* to ensure copper acquisition, both isocyanide moieties are necessary for the chalcophore action.^20–22^ In bacteria, SF2768 causes a decrease in essential intra-cellular copper concentrations through its chelating capacity, leading to an increase in reactive oxygen species (ROS) and thus causing growth inhibition.^15^ In contrast, MoA studies of xanthocillin revealed complexation of free heme, probably *via* iron coordination, and corresponding dysregulation of the heme biosynthesis pathway, leading to the rapid death of bacterial microbes such as the critical priority pathogen *Acinetobacter baumannii.*^16^ Initial efforts to expand the chemical space of synthetic antibiotic isocyanides have been made after the discovery of an antibiotic stilbene carrying isocyanide.^14^ Subsequent SAR studies yielded optimized stilbene diisonitrile and 4-isocyanophenylamide (Fig. 1B).^12–14^ Both compounds are active against Gram-positive bacteria, showing bacteriostatic activity.^13^ However, their MoA remains unknown.^12,13^ Given the scarce information on the isocyanide MoA, a systematic analysis will be crucial for identifying novel antibiotic mechanisms as inspiration for developing the next generation of drugs addressing unprecedented targets.

The purpose of this study is to provide a more comprehensive understanding of the MoA of the neglected class of monoisonitriles, and to expand the chemical space of these compounds, serving as a potential starting point for the development of innovative antibiotics. We initiated our work by screening a panel of commercially available isocyanide compounds for their antibiotic activity. The most active hit molecule was dissected *via* chemical derivatization, highlighting the importance of the isocyanide moiety for bacterial growth inhibition. Chemical proteomics revealed covalent binding to two essential metabolic enzymes, which were confirmed as targets contributing to the antibacterial effect.

## Results

### Screening of commercial isocyanides for antibiotic activity

To cover a broad range of structurally diverse compounds, we gathered commercially available primary, secondary, tertiary, vinyl and aromatic isocyanides for antibacterial testing (Table S1†). Previous investigations suggest that secondary and tertiary isocyanides are preferred due to higher metabolic stability.^8^ The minimum inhibitory concentration (MIC) of forty-two compounds was determined in Gram-positive *Staphylococcus aureus* and Gram-negative *Escherichia coli* cells up to 50 μM. While none of the compounds displayed activity against *E. coli*, isocyanides I19, I25, and I32 reduced growth and I20 fully inhibited growth of *S. aureus* at 50 μM (Fig. 1C and Table S1†). Among the screened compounds, I16, an aromatic sulfonyl isonitrile, stood out with a potent MIC of 1.6 μM (Fig. 1D and Table S1†). A time-kill assay showed the bacteriostatic activity of I16 (Fig. 1E), distinguishing it from bactericidal Xan.^16^ The compound was tested against a panel of Gram-positive and Gram-negative pathogens. Although no antibiotic effects against Gram-negative strains were observed, *Bacillus subtilis*, *Listeria monocytogenes*, *Staphylococcus epidermidis*, and *Streptococcus pneumoniae* could be inhibited at low micromolar concentrations of I16 (Table S2†). The lack of activity against Gram-negative bacteria was likely caused by limited uptake as testing against lipopolysaccharide (LPS)-deficient *E. coli* or the addition of polymyxin B nonapeptide (PMBN), a membrane permeabilizer, resulted in growth inhibition (Fig. S1†). Toxicity against human HeLa cells occurred with an IC_50_ of 25 μM, suggesting a narrow application window (Fig. S2†). This was further corroborated by evaluation I16 in four different human cytochrome P450 enzymes (CYPs). I16 inhibited all tested CYP isoforms (2C9, 2D6, 1A2, and 3A4) at nanomolar concentrations which is in a comparable range with known inhibitors (Table S3†).

To decipher core structural elements essential for I16 bioactivity in bacteria, we conducted SAR studies and synthesized fifteen analogs bearing variations in the substitution of both aromatic ring systems, and replacements of the aromatic rings with aliphatic chains according to previously published procedures (Fig. 1F).^23–28^ The first reaction step consists of the reduction of sulfonyl chloride to sulfinic acid. Subsequently the acid reacts with an aldehyde and formamide to produce a formamide derivative. In the final step, the dehydration reaction with phosphoryl chloride yields the corresponding isocyanides. The isocyanide derivatives (1–15) were next tested for antibiotic activity (Fig. 1G). While, the replacement of either aromatic ring or variations of the substitution pattern of the eastern aromatic system impaired their activity, halogen substitutions in *ortho*- and *para*-position of the eastern aromatic system performed best. Increased electron density in the western aromatic system appeared to be beneficial for the bioactivity. Overall, none of the derivatives exceeded the anti *S. aureus* potency of the initial hit I16. Remarkably, some analogs displayed moderate activity against *E. coli*, *i.e.* compounds 2 and 11 inhibited the growth of the Gram-negative pathogen with an MIC of 25 μM. Additionally, two benzyl aryl sulfones lacking the isonitrile group, BAS2 and BAS7 (Fig. 1G), were synthesized through a nucleophilic substitution reaction between *p*-toluene sulfinic acid and the respective benzyl chloride, following a published procedure.^29^ Neither compound exhibited any antibiotic effect. Furthermore, the formamide analogs FA1 and FA7 proved to be inactive against *S. aureus* (Fig. 1G). These results underscore the critical role of the signature motif for antibacterial activity. We thus commenced our studies with an in-depth investigation of the underlying MoA.

### I16 exhibits a different MoA compared to diisonitriles

To find the molecular targets of I16, we first excluded general effects on the membrane and DNA binding. Neither the *S. aureus* membrane potential nor its integrity was influenced by I16 treatment (Fig. S3†). Moreover, no MIC changes were obtained by adding DNA to the culture (Fig. S4†). Given the importance of the metal binding and chelating properties for the MoA of the natural isocyanides Xan and SF2678, we first investigated if I16 displays similar traits. No significant copper binding of I16 could be observed *via* isothermal titration calorimetry (ITC). In addition, growth assays in the presence of metal and I16 did not show synergistic or antagonistic effects (Fig. S5 and S6†). I16 also binds to a lesser extent to heme than Xan (Fig. S7†). The difference in the MoA between I16 and Xan was further corroborated by re-investigating Xan-resistant *A. baumannii* strains,^16^ which were sensitive to I16 with MIC comparable to that of wt cells (PMBN was used as permeabilizer to facilitate entry of I16) (Table S4†). Moreover, while Xan induced oxidative stress, which could be partially rescued by thiourea addition, no effect of thiourea could be seen in case of I16 (Fig. S8†). Finally, inductively coupled plasma mass spectrometry (ICP-MS) studies with I16-treated *S. aureus* cells showed no significant changes in metal ion abundance, including Cu, Fe, Zn, Mn, Mo, and Ni, ruling out any effects of I16 on metal homeostasis (Fig. S9†). These results point towards a different MoA compared to previously investigated natural isonitriles.

### Proteome changes upon I16-induced stress

**Fig. 2:**
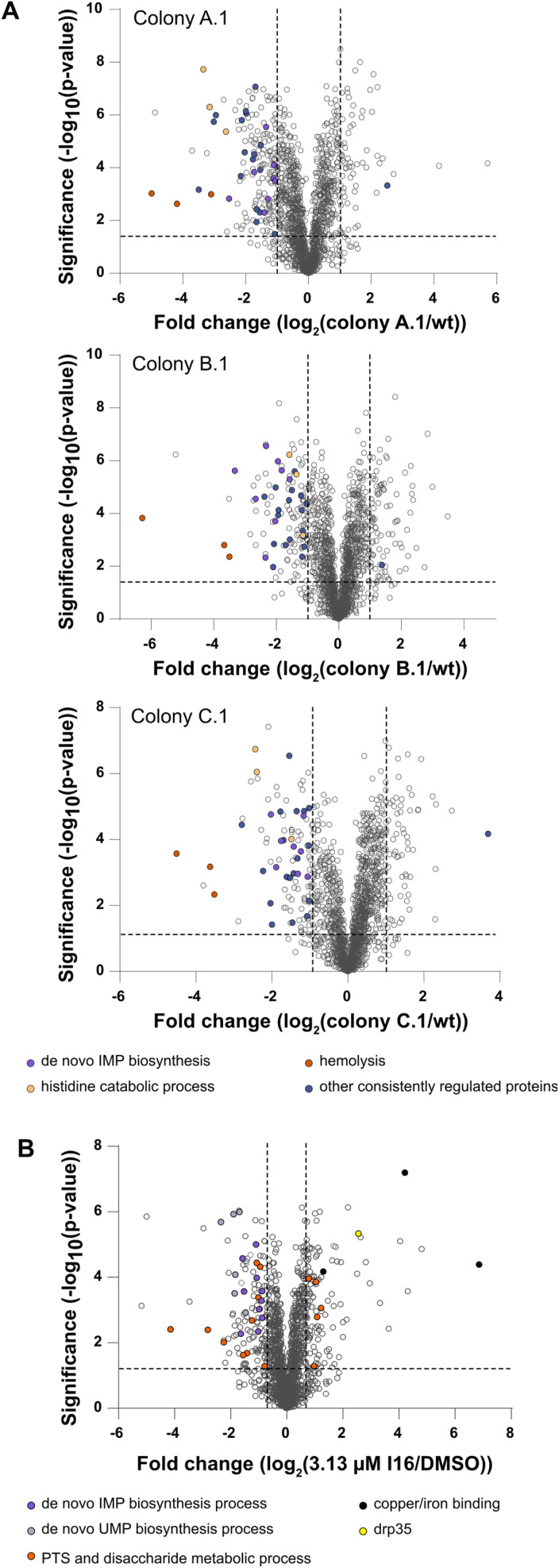
(A) Full proteome analysis of I16-resistant colonies compared to *S. aureus* NCTC 8325 wt. The volcano plots show the fold change of protein levels. The dashed lines indicate significance cut-offs at −log 10(*p*) = 1.3 and log 2 = 1 (two-sided two-sample *t*-test, *n* = 4 independent experiments per group). Proteins consistently regulated in all three colonies are shown in violet. Orange (hemolysis in other organisms), beige (histidine degradation to formate/formamide), and purple (*de novo* IMP biosynthesis) dots represent consistently downregulated pathways. (B) Full proteome analysis of I16-treated *S. aureus* NCTC 8325 compared to a vehicle control (1% DMSO). The volcano plots show the fold change of protein levels. The dashed lines indicate significance cut-offs at −log 10(*p*) = 1.3 and log 2 = 0.7 (two-sided two-sample *t*-test, *n* = 4 independent experiments per group). The *de novo* IMP biosynthesis process (purple), the *de novo* UMP biosynthesis process (violet) and the phosphotransferase system and disaccharide metabolic process (orange) are pathways/clusters that are significantly regulated. Among the strongest upregulated proteins are copper/iron-binding proteins (black). Drp35 (yellow) is a lactonase that could hint towards a MoA targeting the membrane biosynthesis process.

As direct target identification *via* chemical proteomics failed in the case of Xan, the selection of resistant bacterial strains turned out to be a successful strategy. Thus, we also initiated our MoA studies by serial passaging of *S. aureus* treated with I16 and selected resistant derivatives. Although resistance development was slow, we isolated three colonies of each strain for sequencing and obtained a high number of individual, strain-specific mutations also in non-coding regions (Fig. S10†). To gain more insights into the physiological consequences of these mutations, we performed MS-based whole proteome and functional enrichment analyses (using the STRING database^30,31^) of a colony of each strain and, despite the differences in the individual mutation pattern, observed common downregulated pathways (Fig. 2A and Table S5†). Among those, the “*de novo* IMP biosynthetic pathway” (count: 8/12; indicates the number of term-related up- and downregulated proteins in comparison to all proteins associated with the term) the “histidine degradation pathway to glutamate and formate/formamide” (count: 3/4), and “hemolysis in another organism” (count: 3/7) were significantly downregulated compared to the wt. No common functional enrichment could be detected among the upregulated proteins in each colony. Overall, there is a more significant overlap of downregulated proteins than upregulated proteins between the mutants (Fig. S11†).

Interestingly, whole proteome and functional enrichment (STRING database^30,31^) analysis of I16 treated *S. aureus* wt (Fig. 2B and Table S6†) shows similar downregulation of the “*de novo* IMP biosynthesis pathway” (count: 10/12) and additionally the “UMP biosynthesis pathway” is also significantly downregulated (count: 6/7). A cluster that shows up- and down regulations is the “phosphotransferase system (PTS) and disaccharide metabolic process” network (count: 15/63). Among the significantly downregulated PTS proteins some play crucial roles in the cell wall recycling process: murP (MurNAc–GlcNAc transporter; Uniprot ID: Q2G1G5), mupG (6-phospho-*N*-acetylmuramidase; Uniprot ID: Q2G1G7) and murQ (*N*-acetylmuramic acid 6-phosphate etherase; Uniprot ID: Q2G1G6). Also enzymes of the peptidoglycan hydrolysis, recycling, and salvaging pathways (bifunctional autolysin atl (Uniprot ID: Q2FZK7) and peptidoglycan hydrolase sle1 (Uniprot ID: Q2G0U9)) are downregulated even at the cost of survival fitness.^32–34^

Interestingly, the copper-exporting P-type ATPase copA (Uniprot ID: Q2FV64), the copper chaperone copZ (Uniprot ID: Q2FV63), and the Fe/B12 periplasmic-binding domain-containing protein (Uniprot ID: Q2G071) are among the most upregulated proteins. Those proteins mediate intracellular transportation and export of copper and iron ions.^35,36^ However, given the lack of significant intracellular changes of copper or iron levels, the upregulation likely stems from a secondary effect or binding to another yet undeciphered substrate of this channel.

Furthermore, elevated levels of lactonase drp35 (Uniprot ID: Q2FUS8) are observed, which is induced by membrane-acting antibiotics and detergents.^37,38^ As we could rule out a direct membrane perturbation from I16, the expression changes of drp35 and cell wall recycling processes points towards a protein target linked to cell wall biosynthesis.^34,37,38^

**Fig. 3:**
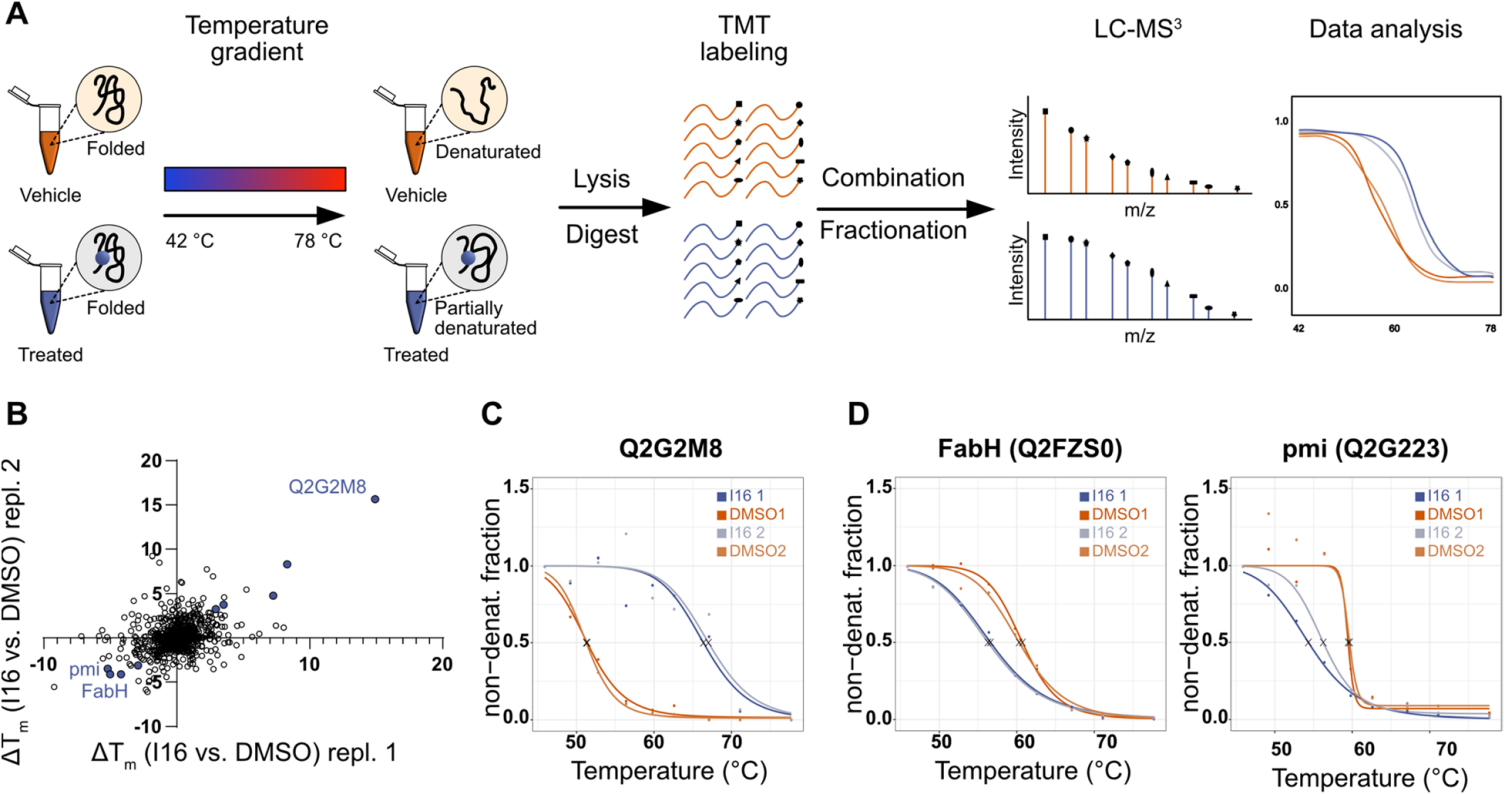
Evaluation of target engagement and targeted pathways of I16 in *S. aureus* NCTC 8325 using thermal proteome profiling (TPP). (A) Schematic workflow of the TPP experiment. Living *S. aureus* cells were treated with 10 μM I16 or DMSO (1%, vehicle control) for 1 hour. Next, aliquots were subjected to 10 different temperatures ranging from 42 to 78 °C. After cell lysis, removal of denatured proteins, and tryptic digest, the resulting peptides were labeled with TMT reagents. Subsequently, aliquots of one sample were pooled, fractionated, and subjected to LC-MS^3^ measurement. Melting curves were determined. (B) Scatterplot of *T*_m_ shifts determined from two biological replicates of I16*vs.* DMSO treatment. Proteins with melting-point shifts passing all significance criteria (Fig. S12†) are shown in violet. (C) Thermal-response curves and calculated melting points (*X*) for the most stabilized protein nucleoside triphosphate/diphosphate phosphatase (Uniprot ID: Q2G2M8) of I16-treated (violet) and DMSO-treated (orange) cells. Two biological replicates are shown. (D) Thermal-response curves and calculated melting points (*X*) of I16-treated (violet) and DMSO-treated (orange) cells for the most destabilized hits. Two biological replicates are shown.

To obtain a complementary picture of I16 affected pathways we performed thermal proteome profiling (TPP) which reports stabilized and destabilized proteins upon compound treatment. Bacterial cells were incubated with I16 or DMSO and subsequently heated up to temperatures ranging from 42.3 °C to 77.7 °C. Cells were lysed and soluble proteins isolated, tryptically digested and labeled with isobaric tandem mass tag labels (TMT). Samples were pooled, fractionated, and finally analyzed by LC-MS^3^ (Fig. 3A). Proteins are more stable upon compound-binding to heat denaturation and remain soluble compared to the untreated control.^39,40^ Destabilizations can be expected up- and downstream of direct targets due to changes of metabolite concentrations or destabilized protein complexes.^39^ For each protein a characteristic melting curve is obtained.^41,42^

Several proteins were significantly stabilized or destabilized upon treatment with I16 (Fig. 3B and Table S7†). The most stabilized hit in the TPP experiment passing all significance criteria is a nucleoside triphosphate/diphosphate phosphatase (Uniprot ID: Q2G2M8) (Fig. 3C). It is noteworthy that this non-essential phosphatase has been reported to play a crucial role in virulence and is required for hemolysin production,^43^ providing a link to the compound-treated wild type and resistant strains (Tables S5 and S6†).

Interestingly, the treatment with I16 resulted in the characteristic destabilization of proteins involved either in fatty acid biosynthesis (beta-ketoacyl-[acyl-carrier-protein] synthase III, FabH, Uniprot ID: Q2FZS0) or in amino sugar and nucleotide sugar metabolism (mannose-6-phosphate isomerase, pmi, Uniprot ID: Q2G223) (Fig. 3D).

These data narrow down putative target areas and, together with the diverse mutations and slow resistance development, suggest that multiple targets could be responsible for the MoA.

### I16 binds essential metabolic enzymes

**Fig. 4:**
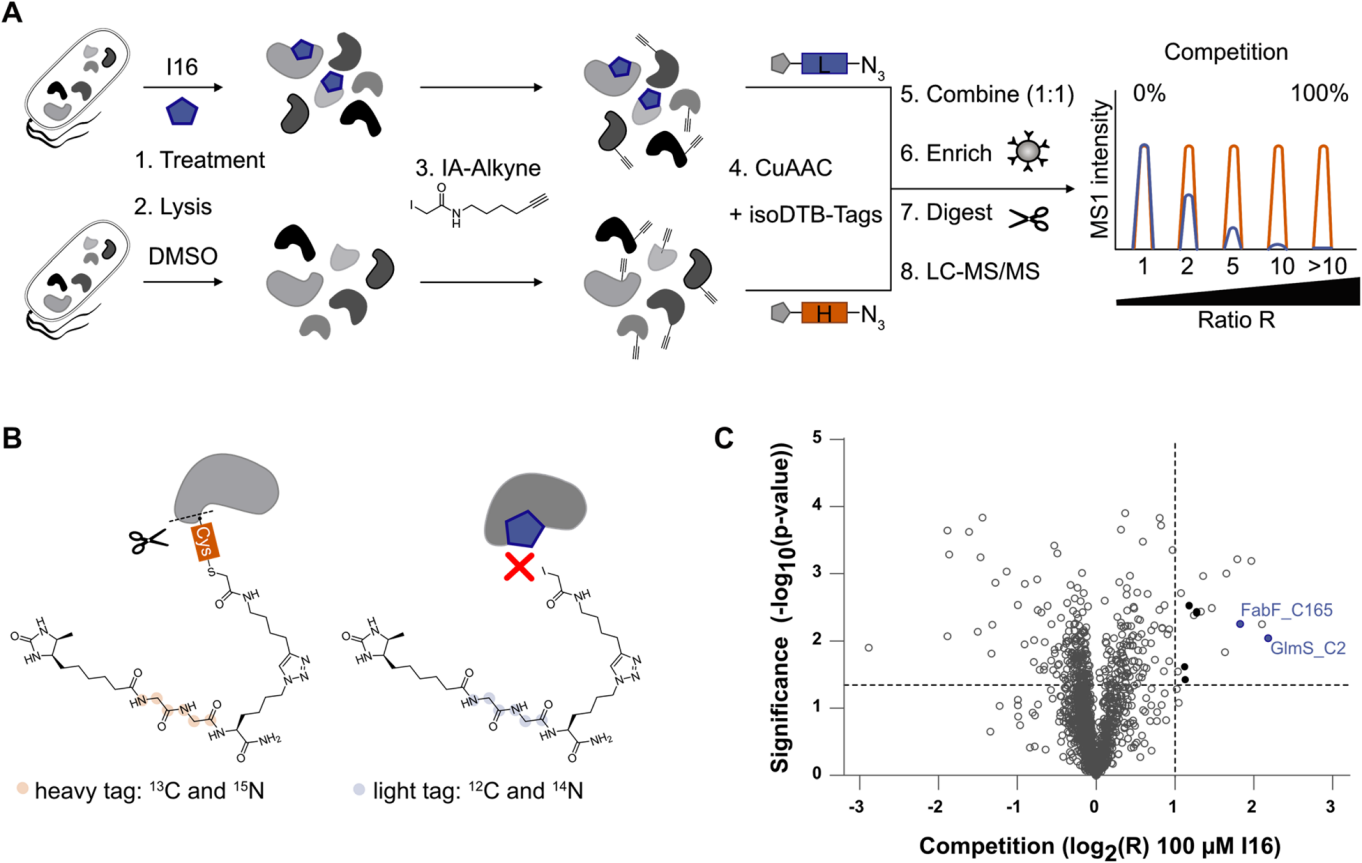
Analysis of covalently modified protein targets of I16 in *S. aureus* NCTC 8325. (A) Schematic workflow for competitive, residue-specific proteomics with the isoDTB-ABPP platform. Living cells were treated with I16 (100 μM) or DMSO (1%) and subsequently lysed, labeled with IA-alkyne (100 μM), and clicked to either a light (compound) or heavy (DMSO) isoDTB-tag. Samples were combined in a 1 : 1 ratio, enriched on streptavidin beads, tryptically digested, and analyzed *via* LC-MS/MS. The ratio between detected heavy- and light-tagged peptides indicates which cysteines are covalently engaged with I16. dTB: desthiobiotin. (B) Schematic representation of the competition between compound and IA-alkyne. Only peptides with cysteines bound to IA-alkyne can be detected, leading to a higher heavy to light ratio (*R*) for compound-engaged cysteines. (C) Volcano plot for the isoDTB experiment with 100 μM I16. Essential active-site cysteines that are compound-engaged are highlighted in violet. Proteins binding iron–sulfur clusters are shown in black. The dashed lines indicate cut-offs at −log 10(*p*) = 1.3 and log 2(*R*) = 1 that were used as criteria for hit selection. Data represent *n* = 4 independent experiments.

To find direct protein targets, activity-based protein profiling (ABPP) is an established chemical proteomics tool.^44,45^ Given the essential role of the isocyanide group for the bioactivity of I16 and its electrophilic properties towards nucleophiles, we reasoned that reactive cysteine residues could be directly modified. To test this hypothesis, we performed competitive, residue-specific isoDTB-ABPP experiments, that are derived from isoTOP-ABPP (isotopic tandem orthogonal proteolysis-ABPP).^46–48^I16 was added to living *S. aureus* cells, followed by lysis and treatment with a cysteine targeting iodoacetamide alkyne (IA-alkyne). In parallel, samples lacking the I16 pretreatment were prepared. After click-reaction to isotopically labeled desthiobiotin azide (isoDTB) tags,^48^ the compound treated samples and the control samples were combined, enriched on streptavidin beads, and the tryptically digested proteins were analyzed *via* LC-MS/MS (Fig. 4A and B). The resulting peptides were quantified relative to each other and their ratio *R* between the heavy-labeled (vehicle control) and light-labeled (I16-treated) samples revealed cysteines that are engaged *via* covalent binding to the isocyanide compound. Notably, among the 18 significantly engaged cysteines, five are involved in iron–sulfur cluster binding. Overall, the engaged cysteines belong to proteins that have various molecular functions. Only two essential enzymes, glutamine–fructose-6-phosphate aminotransferase (GlmS, Uniprot ID: Q2FWA0) and 3-oxoacyl-[acyl-carrier-protein] synthase 2 (FabF, Uniprot ID: Q2FZR9), were identified among the major hits (Fig. 4C, Tables S8 and S9†).^49^ Importantly, I16 modified these enzymes at their active site cysteines C2 for GlmS and C165 for FabF (Table S9†) suggesting an impact on their catalytic activity. GlmS belongs to the amidotransferase enzyme family. Its monomer has two distinct enzymatic domains, the N-terminal glutaminase and the C-terminal isomerase domain.^50–52^ GlmS is the rate-limiting enzyme in the bacterial hexosamine pathway that converts glutamine and fructose-6-phosphate (Fru-6P; from glucose) to glutamate and d-glucosamine-6-phosphate (GlcN-6P).^53^ GlcN-6P is a precursor of UDP-*N*-acetylglucosamine (UDP-GlcNAc), an essential building block of the bacterial peptidoglycan.^52,54^ FabF catalyzes the Claisen condensation of an acyl thioester (acyl-ACP) with malonyl-ACP to form 3-ketoacyl-ACP and ACP, which is an essential step in the elongation cycle of the fatty acid synthesis pathway.^55,56^ These crucial roles in cell wall metabolism fit the observed protein expression changes of I16-treated cells, meriting further validation studies.

### Validation of GlmS and FabF as covalent targets of I16

**Fig. 5:**
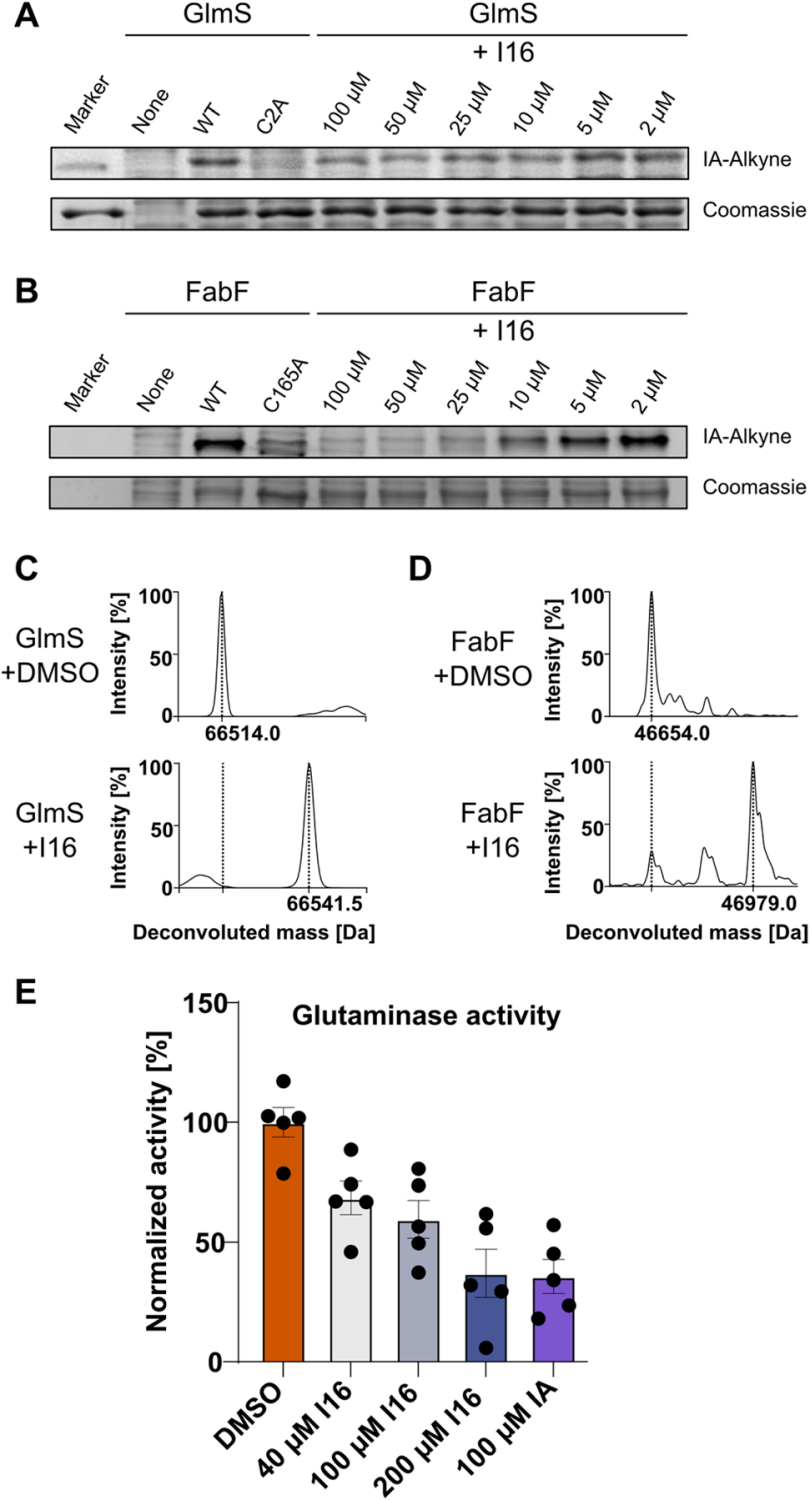
Validation of the cysteine reactivity of I16. (A) Gel-based labeling experiments with GlmS. Recombinant wild-type GlmS, the C2A mutant (2 μM) or no enzyme (none) were added to soluble lysate of *S. aureus* NCTC 8325, treated with I16, labeled with IA-alkyne and clicked to TAMRA-azide. SDS-PAGE was used for analysis, and the results of in-gel fluorescence scanning and Coomassie staining are shown. (B) Gel-based labeling experiments with FabF. Recombinant wild-type FabF, the C165A mutant (2 μM) or no enzyme (none) were added to soluble lysate of *S. aureus* NCTC 8325, treated with I16, labeled with IA-alkyne and clicked to TAMRA-azide. SDS-PAGE was used for analysis, and the results of in-gel fluorescence scanning and Coomassie staining are shown. (C) Treatment of recombinant GlmS (1 μM) with I16 (40 μM) increases the protein mass by 27.5 Da. (D) Treatment of recombinant FabF (1 μM) with I16 (20 μM) leads to a partial protein modification that corresponds to the addition of I16 (+324.0 Da). (E) Glutaminase activity assay with GlmS. GlmS (20 μg mL^−1^) was treated with I16, iodoacetamide (IA), or DMSO as control in the presence of Fru-6P. GDH, glutamine, and APAD were added, and the reaction progress was followed (363 nm). The glutaminase activity was calculated by a linear fit of the linear range of the curve and normalized to the DMSO control. The graph shows mean ± SEM of *n* = 5 independent experiments.

First, we verified I16 cysteine reactivity in an LC-MS-based assay where it formed a covalent adduct with *N*-acetyl-l-cysteine methyl ester (Fig. S13†). Second, I16 binding to GlmS and FabF was validated by recombinant expression of the proteins, followed by competitive labeling in gel-based experiments (Fig. 5A and B). Strong labeling by IA-alkyne could be observed for the wild type proteins which was reduced for the GlmS_C2A and the FabF_C165A mutants. Furthermore, increasing concentrations of I16 diminished the IA-alkyne labeling of the wild type enzymes, highlighting covalent modifications of cysteines through I16 (full size gels are shown in Fig. S14 and S15,† additional labeling experiments are shown in Fig. S16–S19†). Third, to directly show modifications on the proteins of interest after compound treatment we applied intact protein MS (IPMS, Fig. 5C and D). Remarkably, while FabF showed a modification corresponding to the addition of I16 (+324.0 Da), GlmS exhibited only a shift of 27.5 Da after compound-treatment.

To evaluate if binding of I16 to GlmS leads to enzyme inhibition, a glutamate dehydrogenase (GDH) coupled spectrophotometric assay was applied.^51,57,58^ We first confirmed that neither I16 nor iodoacetamide affected GDH turnover and that the GlmS_C2A mutant was inactive (Fig. S20†). Iodoacetamide, which is known to inhibit GlmS by covalently modifying the thiol group of C2,^54,57^ reduced the turnover to about 35% at a concentration of 100 μM (Fig. 5E). Satisfyingly, the enzyme assay demonstrates that glutamate release rate decreased concentration-dependently by I16, corroborating its interaction with the active site cysteine.

## Discussion

In light of the underexploited potential of isonitriles, we performed an antibiotic screening of diverse structural analogs and identified I16 as a potent bacteriostatic agent against Gram-positive bacteria. The therapeutic potential of I16 is restricted due to its cytotoxicity in human cells. The inhibition of different cytochrome P450 enzymes by I16 may be a potential reason for this effect and requires further structural refinement of the hit molecule.

SAR studies confirmed the importance of the isocyanide moiety and the aromatic ring systems with specific substitution patterns responsible for the antibiotic activity. A set of tailored assays revealed that this compound class exhibits a distinct MoA, beyond metal or heme complexation, involving multiple targets which was further corroborated by the generation and sequencing of resistant strains. Given the intrinsic electrophilic character of the isocyanide group, we speculated that covalent interactions with proteins might be responsible for the bioactivity. In fact, competitive residue-specific isoDTB-ABPP experiments revealed direct binding of I16 to nucleophilic cysteines in enzyme active sites. Among those, two essential active site cysteines of the enzymes FabF and GlmS were identified and validated.

I16 targeting of FabF could explain the downregulation of two enzymes of the acetyl-CoA carboxylase complex (Uniprot ID: Q2FXX1/Uniprot ID: Q2FXX0) in compound-treated *S. aureus* cells (Table S6†). This complex is responsible for the biosynthesis of malonyl-CoA and is the first committed and rate-limiting step in bacterial fatty acid synthesis.^55,59^ Malonyl is transferred to the acyl carrier protein (ACP) and serves as substrate for the condensing enzymes of the fatty acid synthesis pathway FabF and FabH.^55,59^ In line with this observation is the destabilization of FabH in TPP experiments, which is encoded upstream of FabF.^55^

Regarding GlmS, concentration-dependent labeling of the recombinant protein and inhibition of its glutaminase activity was demonstrated. Within the GlmS glutaminase domain, the thiol group of the active cysteine (C2) is responsible for the nucleophilic attack on the δ-carbonyl group of l-glutamine, forming a thioester intermediate that is subsequently hydrolyzed by water, yielding glutamate and ammonia.^52,54,60,61^ Considering the truncated I16 modification seen in IPMS studies, based on the enzyme's mechanism we suggest that the thiol group of C2 attacks the isocyanide moiety of I16, leading to an iminomethyl-adduct. Subsequent hydrolysis by water results in a formylated cysteine which matches the obtained mass shift. GlmS is the initiating enzyme of the cell wall synthesis pathway in *S. aureus*.^53^ Its expression is strictly regulated through feedback inhibition *via* a GlcN-6P binding riboswitch,^62,63^ changes in protein expression and dysregulation in metabolic pathways are expected when GlmS is functionally impaired. The upregulation of drp35, an indicator of disturbed membrane synthesis, as well as dysregulations of the PTS and notably the downregulation of enzymes of the peptidoglycan recycling process are in line with this notion. The observed destabilization of the upstream to the GlmS gene located mannose-6-phosphate isomerase (pmi) is another indicator of an imbalanced hexosamine pathway.

## Conclusions

Many natural isocyanides exhibit extraordinarily potent bioactivities highlighting a unique role of this functional group. Despite exhibiting this role as a privileged structural motif, isonitriles have been overlooked for many years. Previously discovered antibiotic MoAs of natural isocyanides rely on the metal coordinative capacity of the functional group, leading to either copper or heme dysregulations within the treated cells. In this work, we disclosed a third antibiotic isocyanide MoA, based on its electrophilic character, allowing interactions with nucleophilic cysteines.

Even though our hit molecule will not serve as a future antibiotic candidate, because of toxicity issues, Brunelli *et al.* have shown that extensive SAR studies can lead to an antibiotic isocyanide hit that overcomes cytotoxicity.^13^ Their lead structure 2-(3-chlorophenyl)-*N*-(4-isocyanophenyl)acetamide (Fig. 1B) is active against *S. aureus* (2 μM MIC against *S. aureus* ATCC 25923) without exhibiting cytotoxicity against HaCaT and hOBs cells and exhibiting decent metabolic stability.^13^ To exploit isocyanides in a therapeutic set-up, careful cytotoxicity and off-target investigations are needed.

Given the chameleonic nature of the isocyanide function that allows its carbon atom to undergo a large variety of reactions in organic chemistry,^5^ it is likely that more protein targets will be deciphered in which interactions with the electrophilic, nucleophilic or coordinative properties of other isocyanides of natural and synthetic origin are crucial. Isocyanides can serve bioorganic chemists as an excellent tool for discovering those hidden targets and for finding novel resistance-free antibacterial MoA. Overall, isocyanides can be considered an out-of-the-box starting point for the development of innovative antibiotics with a restricted direct therapeutic applicability.

## Data availability

The mass spectrometry proteomics data have been deposited to the ProteomeXchange Consortium *via* the PRIDE partner repository^64^ with the dataset identifier PXD050437. Whole-genome sequencing data and metadata are available on the NCBI SRA repository Bioproject Number PRJNA1088756 available at: https://www.ncbi.nlm.nih.gov/bioproject/1088756.

## Author contributions

A. G. and S. A. S. conceived and designed the project; A. G. performed the isocyanide antibiotic screening, synthesis, mass spectrometry experiments, protein purification, biological assays and data analysis; H. J. performed genome and transcriptome sequence data analysis of *S. aureus* mutants; A. G. and L. J. D. did sample preparation for ICP-MS measurements; A. M. K. performed CYP assays and data evaluation; A. G. and S. A. S. wrote the manuscript with input from all authors.

## Conflicts of interest

S. A. S is co-founder of Smartbax Limited.

## Supplementary Material

SC-015-D4SC01940G-s001

SC-015-D4SC01940G-s002

SC-015-D4SC01940G-s003

SC-015-D4SC01940G-s004
