# Supplementary material for: Isocyanides inhibit bacterial pathogens by covalent targeting of essential metabolic enzymes
Source: Chem Sci. 2024 Jun 26;15(30):11946–55. doi: 10.1039/d4sc01940g (PMC11290450; doi:10.1039/d4sc01940g)

Electronic Supplementary Material (ESI) for Chemical Science.
This journal is © The Royal Society of Chemistry 2024

Supplementary Information

Isocyanides inhibit bacterial pathogens by covalent targeting of essential metabolic enzymes

Alexandra Geißler,[a] Howard Junca,[b] Andreas M. Kany,[c,d] Lena J. Daumann,[e] Anna K. H. Hirsch,[c,f,d] Dietmar H. Pieper[b] and Stephan A. Sieber*[a,c]

a. Center for Functional Protein Assemblies, Department of Bioscience, TUM School of Natural Sciences, Technical University of Munich, Ernst-Otto-Fischer-Straße 8, 85748 Garching, Germany. E-mail: stephan.sieber@tum.de

b. Microbial Interactions and Processes Research Group, Helmholtz Centre for Infection Research, Inhoffenstraße 7, 38124 Braunschweig, Germany.

c. Helmholtz Institute for Pharmaceutical Research Saarland (HIPS) – Helmholtz Centre for Infection Research (HZI), Campus E8.1, 66123 Saarbrücken, Germany

d. Deutsches Zentrum für Infektionsforschung (DZIF) e.V., 38124 Braunschweig, Germany

e. Chair of Bioinorganic Chemistry, Heinrich-Heine-Universität Düsseldorf, Universitätsstraße 1, 40225 Düsseldorf, Germany

f. Saarland University, Department of Pharmacy, 66123 Saarbrücken, Germany

Table of contents

Supplementary Figures 4
Supplementary Tables 17
Methods 25
- Bacterial culture conditions 25
- Cell culture 25
- Minimum inhibitory concentration (MIC) 25
- DNA shift assay 25
- Checkerboard assays 26
- Time-kill assay 26
- Membrane potential assay 27
- Membrane integrity assay 27
- Isothermal titration calorimetry (ITC) 27
- UV-vis spectroscopy 27
- Elemental analysis by inductively coupled plasma mass spectrometry (ICP-MS) 28
- MTT assay 28
- CYP inhibition 28
- Generation and sequencing of I16 resistant *S. aureus* NCTC 8325 29
- Full proteome analysis 30
- isoDTB-ABPP experiments in living bacteria 31
- Thermal proteome profiling (TPP) 34
- MS-based analysis of I16 cysteine reactivity 37
- Cloning 37
- Protein overexpression and purification 38
- SDS-PAGE 39
- Intact protein MS (IPMS) experiments 39
- Gel-based labeling experiments 39
- GDH-coupled GlmS assay 40

Chemical Synthesis 41
- General information 41
- Analytical Methods 41
- Synthetic procedures and analytical data 41
  - General synthesis scheme 41
  - Sulfinic acids 42
  - Formamides 44
  - Isocyanides 49
  - Benzyl aryl sulfones 54

Supplementary references 55

Appendix 57
Amino acid sequences of expressed proteins 57
Sequences of plasmids used in this study 58
^{1}H-, ^{13}C-NMR-Spectra 63

Supplementary Figures

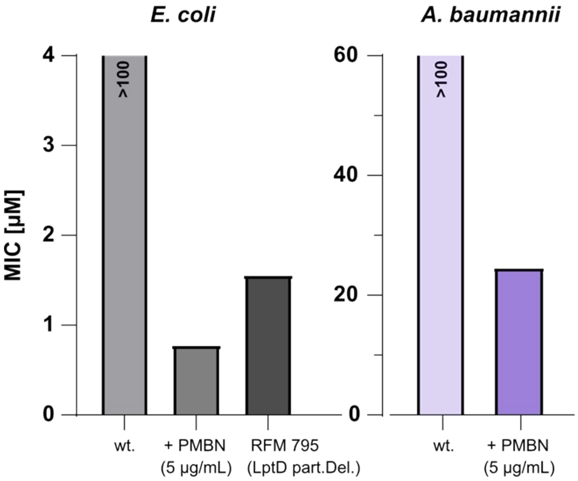

Figure S1. Activity of **I16** in permeabilized Gram-negative strains. The MIC of **I16** in wild type *E. coli* K12 and *A. baumannii* ATCC 19606 is above 100 µM. In the presence of the outer-membrane permeabilizer polymyxin B nonapeptide (PMBN) the MIC drops to 0.78 µM in *E. coli* and to 25 µM in *A. baumannii*. In the lipopolysaccharide (LPS) deficient strain *E. coli* RFM 795 (LptD part. Del.)[1] the MIC is 1.56 µM. Data represent means of three (n = 3) independent experiments.

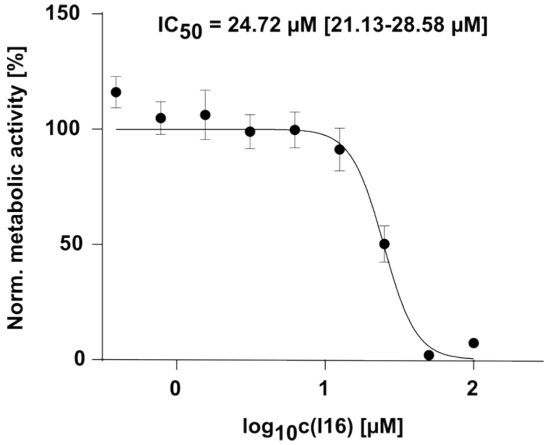

Figure S2. MTT assay. HeLa cells were incubated with different concentrations of **I16** (0.39-100 µM) for 24 h. The remaining metabolic activity was analyzed by quantifying the reduction of 3-(4,5-Dimethylthiazol-2-yl)-2,5-diphenyltetrazoliumbromid (MTT) to its insoluble formazan. The experiment was performed in technical sextuplicates (n = 6) in two independent experiments. Mean values and a non-linear fit of one exemplary experiment are shown. Error bars indicate SD.

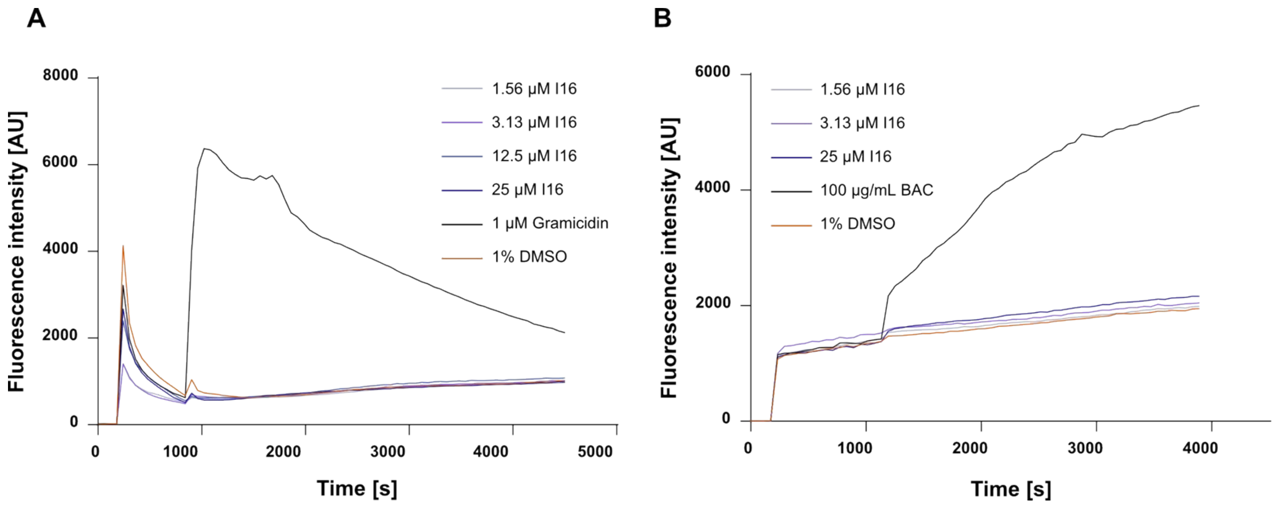

Figure S3. Evaluation of cell membrane permeability. (A) Membrane potential assay. *S. aureus* NCTC 8325 cells were treated with the fluorescent dye DiSC3(5). After complete integration into the cell membrane, different amounts of **I16** (1.56-25 µM), the pore-forming toxin Gramicidin (1 µM) or a vehicle control (1% DMSO) were added and the fluorescence (λ_{ex} = 610 nm and λ_{em} = 660 nm) recorded. When the cell membrane is depolarized, the fluorescence intensity increases as dye is released. The experiment was performed in technical triplicates and repeated twice. Mean values of technical triplicates of one representative experiment are shown. (B) Membrane integrity assay. *S. aureus* NCTC 8325 cells were incubated with propidium iodide (10 µM) for 15 minutes. Next, different amounts of **I16** (1.56-25 µM), the detergent benzalkonium chloride (100 µg/mL) or a vehicle control (1% DMSO) were added and the fluorescence (λ_{ex} = 535 nm and λ_{em} = 617 nm) recorded. Under damaged cell wall conditions, propidium iodide can intercalate in released DNA which leads to an increase in fluorescence. The experiment was performed in technical triplicates and mean values are shown.

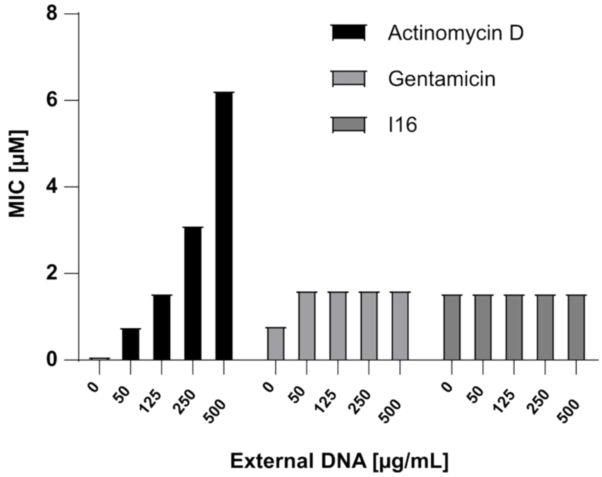

Figure S4. DNA shift assay. The MICs of Actinomycin D, Gentamicin and **I16** against *S. aureus* NCTC 8325 were determined in the presence of external DNA (0-500 µg/mL). The depicted data represent averaged technical quadruplicates from one experiment and are representative for n = 2 independent experiments.

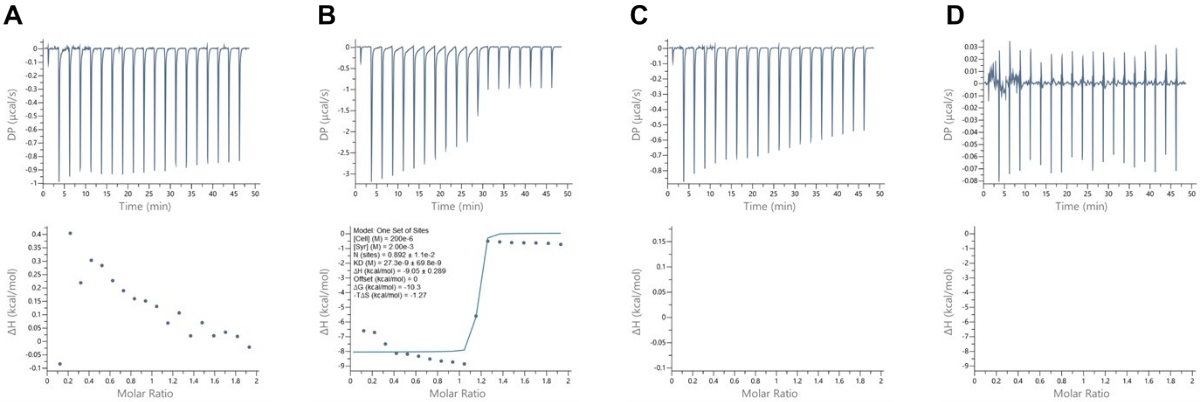

Figure S5. ITC measurements to determine binding between copper(II) chloride ($CuCl_2$, 2 mM), and (A) **I16** (200 µM), and the positive control (B) elesclomol (200 µM). (C) As background control experiment, the titration of $CuCl_2$ to buffer was used. (D) The heat development from a titration of buffer to buffer was neglectable. Experiments were performed in duplicates (n = 2) and representative examples are depicted.

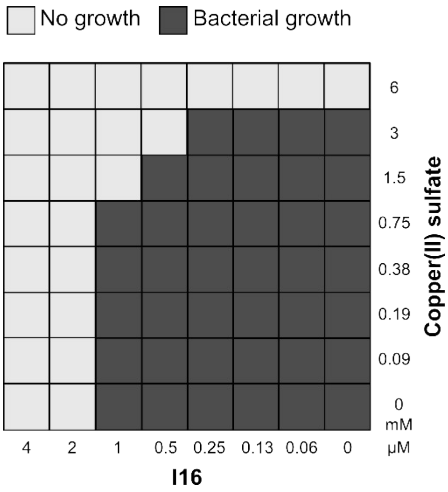

Figure S6. Antimicrobial effects of combinations of **I16** and copper(II) sulfate on *S. aureus* NCTC 8325. The combination of both compounds shows only an additive effect (FIC = 0.5). The checkerboard assay was performed in n = 3 independent experiments and the depicted data are representative for all biological replicates.

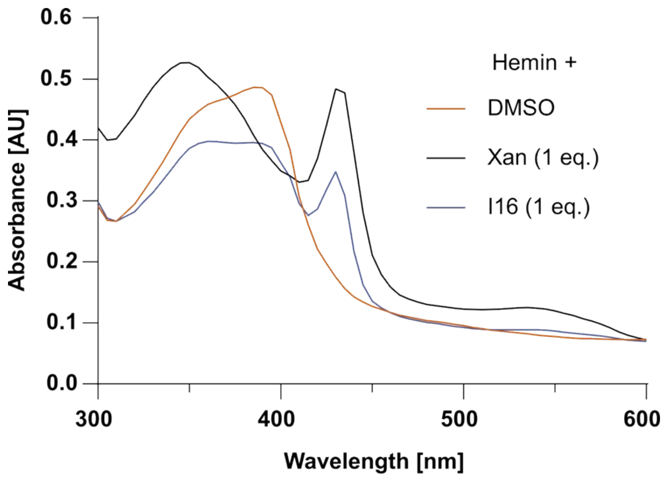

Figure S7. UV-vis spectra of hemin (20 µM) in the presence of DMSO (1%), xanthocillin (20 µM) and **I16** (20 µM) in 200 mM HEPES (pH 7.0). Data represent averaged technical duplicates, and the figure is representative of n = 3 independent experiments.

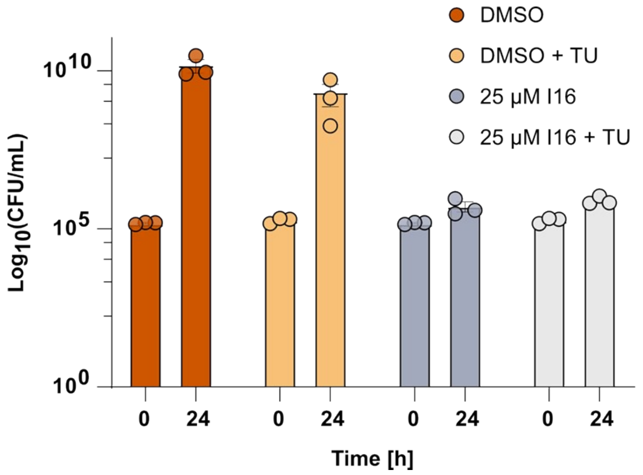

Figure S8. *S. aureus* NCTC 8325 cells were incubated for 24 h with DMSO (1%) or **I16** (25 µM) in the presence or absence of thiourea (TU, 100 mM). After 0 and 24 h viable cells (CFU/mL) were determined in quadruplicates (0 h) and triplicates (24 h). Data represent mean values ± SEM of n = 3 independent experiments.

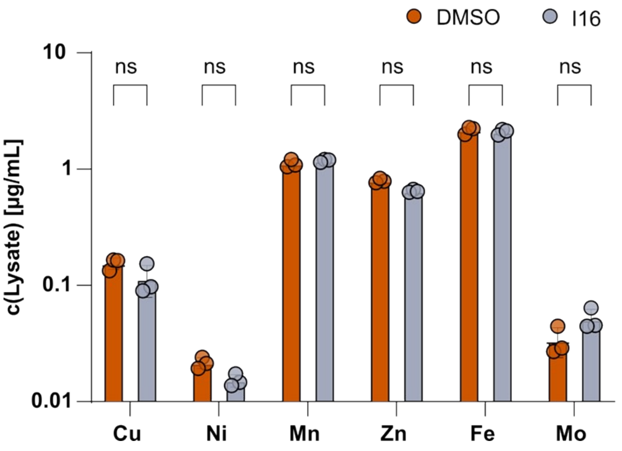

Figure S9. Quantification of intra-cellular transition metal concentrations via ICP-MS. *S. aureus* NCTC 8325 cells were grown in the presence of **I16** (3.13 µM) or a vehicle control (1% DMSO). Same amounts of exponentially growing cells were harvested, washed with EDTA and buffer and lysed. The lysates were subjected to ICP-MS measurements. Data represent mean values ± SD of n = 3 independent experiments.

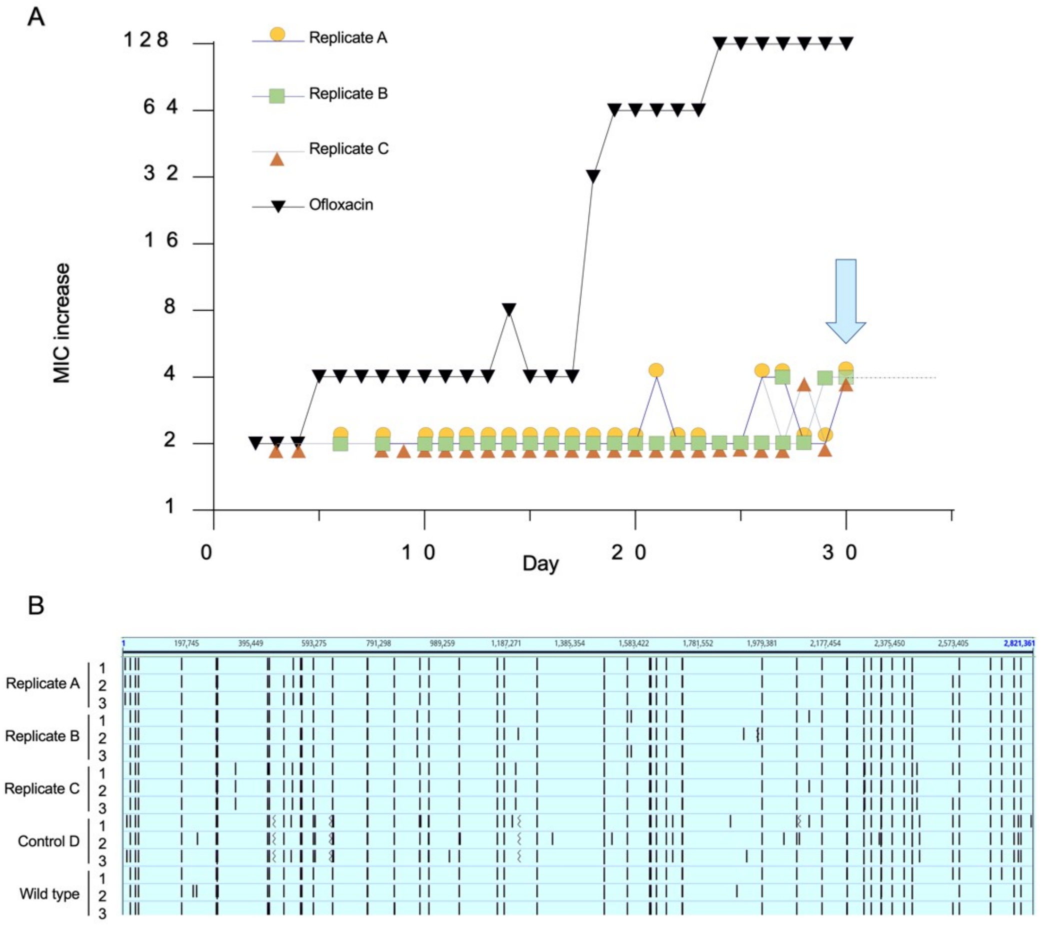

Figure S10. (A) Resistance development of *S. aureus* NCTC 8325 during serial passaging was observed against **I16** (Replicates A-C) or ofloxacin which served as a positive control. Bacteria were grown in sub-MIC concentrations of the antibiotic compounds, and bacteria from the second lowest concentration that allowed growth were used as inoculum for the following passage. Replicates reached the maximum MIC increase obtained at day 30 and three colonies were collected from each replicate. (B) Overview of mutations (polymorphisms) detected in coding and non-coding regions of the genomes of S. aureus NCTC 8325 derivatives showing 4X MIC increase. The **I16**-treated biological replicates (A,B,C) and a negative controls of cultures grown without isocyanide (D) were obtained from three independent colonies of the wild-type strain of NCTC 8325 stock maintained in our lab (WT) compared against the reference genome for the same strain https://www.ncbi.nlm.nih.gov/nuccore/NC_007795. No single mutation in coding regions was commonly detected in the replicate cultures of derivatives with increased MIC. Overview of all polymorphisms and coding regions showing mutations at protein level at: https://zenodo.org/doi/10.5281/zenodo.10826124

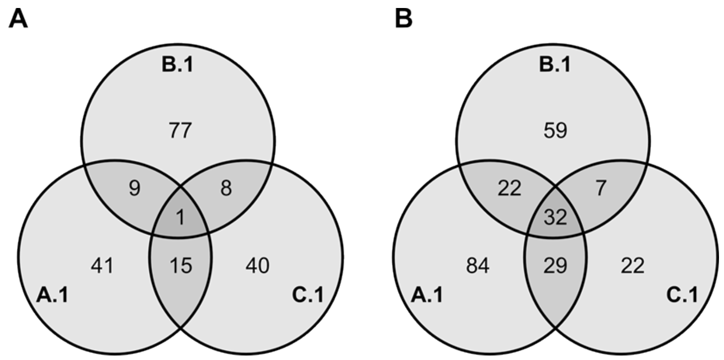

Figure S11. Venn-diagrams showing consistently up- (A) and downregulated (B) proteins in all three *S. aureus* **I16**-mutant colonies that are above the set thresholds of full proteome analysis (p-value = 0.05 (-$\log_{10}$(p-value) = 1.3) and an enrichment factor of 2 ($\log_2(x)$ = 1).

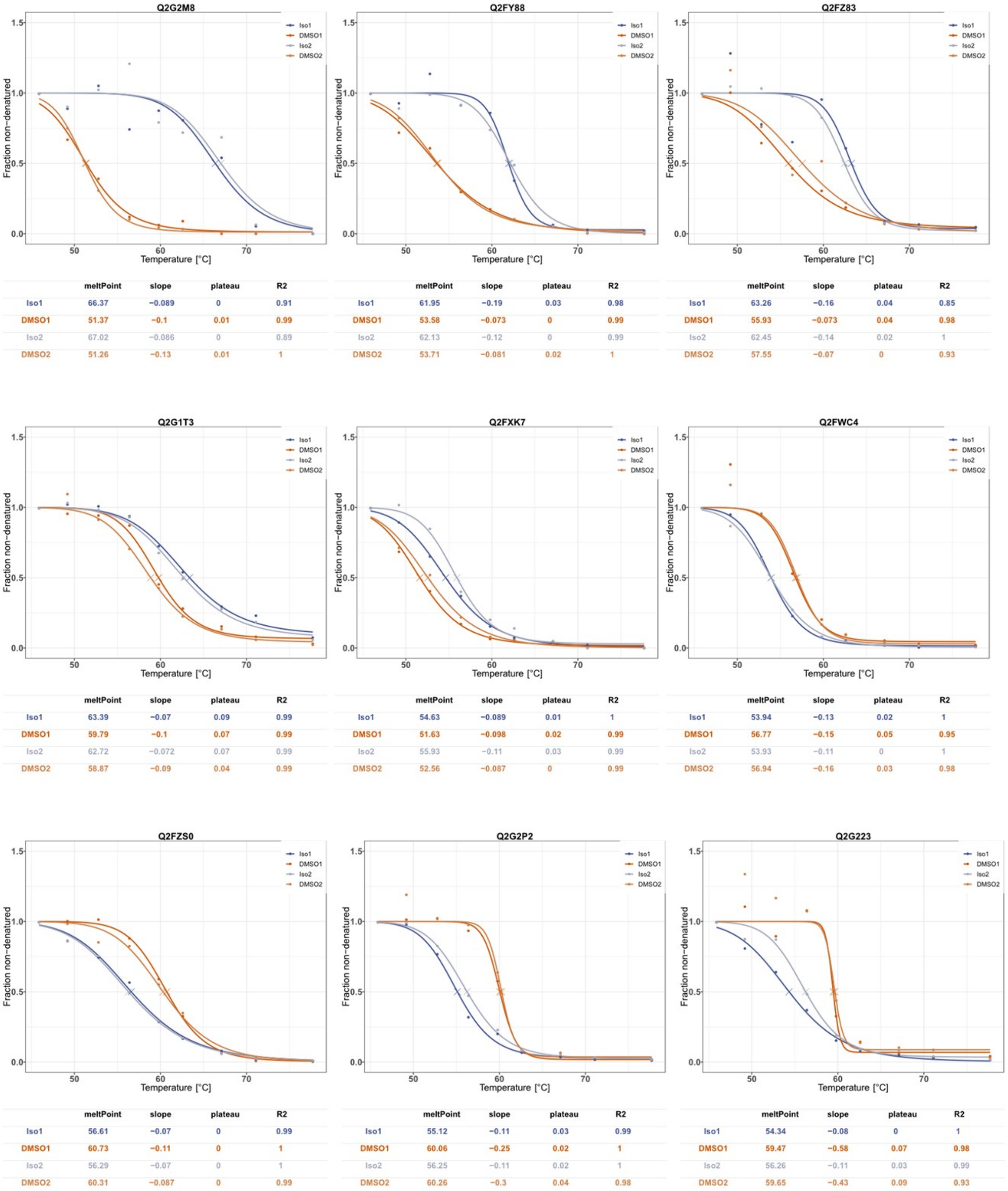

Q2G2M8

	meltPoint	slope	plateau	R2
Iso1	66.37	−0.089	0	0.91
DMSO1	51.37	−0.1	0.01	0.99
Iso2	67.02	−0.086	0	0.89
DMSO2	51.26	−0.13	0.01	1

Q2FY88

	meltPoint	slope	plateau	R2
Iso1	61.95	−0.19	0.03	0.98
DMSO1	53.58	−0.073	0	0.99
Iso2	62.13	−0.12	0	0.99
DMSO2	53.71	−0.081	0.02	1

Q2FZ83

	meltPoint	slope	plateau	R2
Iso1	63.26	−0.16	0.04	0.85
DMSO1	55.93	−0.073	0.04	0.98
Iso2	62.45	−0.14	0.02	1
DMSO2	57.55	−0.07	0	0.93

Q2G1T3

	meltPoint	slope	plateau	R2
Iso1	63.39	−0.07	0.09	0.99
DMSO1	59.79	−0.1	0.07	0.99
Iso2	62.72	−0.072	0.07	0.99
DMSO2	58.87	−0.09	0.04	0.99

Q2FXK7

	meltPoint	slope	plateau	R2
Iso1	54.63	−0.089	0.01	1
DMSO1	51.63	−0.098	0.02	0.99
Iso2	55.93	−0.11	0.03	0.99
DMSO2	52.56	−0.087	0	0.99

Q2FWC4

	meltPoint	slope	plateau	R2
Iso1	53.94	−0.13	0.02	1
DMSO1	56.77	−0.15	0.05	0.95
Iso2	53.93	−0.11	0	1
DMSO2	56.94	−0.16	0.03	0.98

Q2FZS0

	meltPoint	slope	plateau	R2
Iso1	56.61	−0.07	0	0.99
DMSO1	60.73	−0.11	0	1
Iso2	56.29	−0.07	0	1
DMSO2	60.31	−0.087	0	0.99

Q2G2P2

	meltPoint	slope	plateau	R2
Iso1	55.12	−0.11	0.03	0.99
DMSO1	60.06	−0.25	0.02	1
Iso2	56.25	−0.11	0.02	1
DMSO2	60.26	−0.3	0.04	0.98

Q2G223

	meltPoint	slope	plateau	R2
Iso1	54.34	−0.08	0	1
DMSO1	59.47	−0.58	0.07	0.98
Iso2	56.26	−0.11	0.03	0.99
DMSO2	59.65	−0.43	0.09	0.93

Figure S12. TPP Hits. Thermal-response curves and calculated melting points for proteins of **I16**-treated (violet) and DMSO-treated (orange) *S. aureus* NCTC 8325 cells. All shown proteins were significantly stabilized or destabilized by **I16**-treatment (see data analysis for TPP experiments). The Uniprot IDs are indicated.

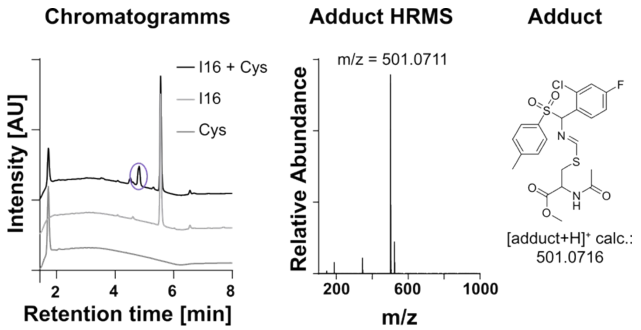

Figure S13. Incubation of **I16** with *N*-acetyl-L-cysteine methyl ester (Cys) can lead to the formation of an adduct. LCMS analysis shows the appearance of an additional signal with a molecular mass corresponding to the suggested molecular structure.

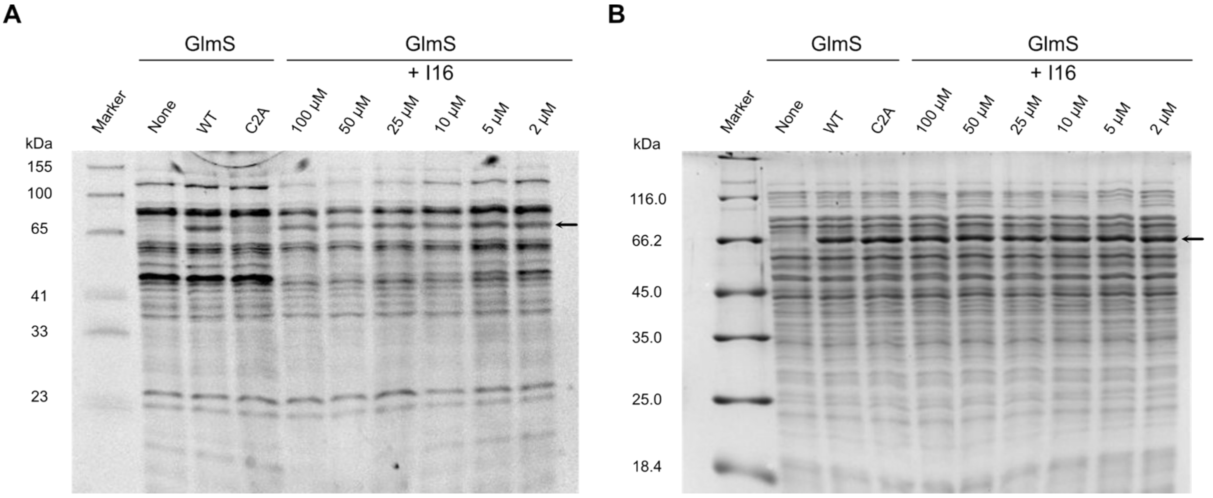

Figure S14. Gel-based labeling experiments with GlmS. 2 µM recombinant wild-type (WT) GlmS was added to 1 mg/mL soluble lysate of *S. aureus* NCTC 8325. As controls, 2 µM of the C2A mutant or no enzyme (None) were included. Samples were treated with the indicated concentrations of **I16** or DMSO, labeled with IA-alkyne (40 µM), and clicked to TAMRA-azide. SDS-PAGE was used for analysis, and the results of in-gel fluorescence scanning (A) and Coomassie staining (B) are shown.

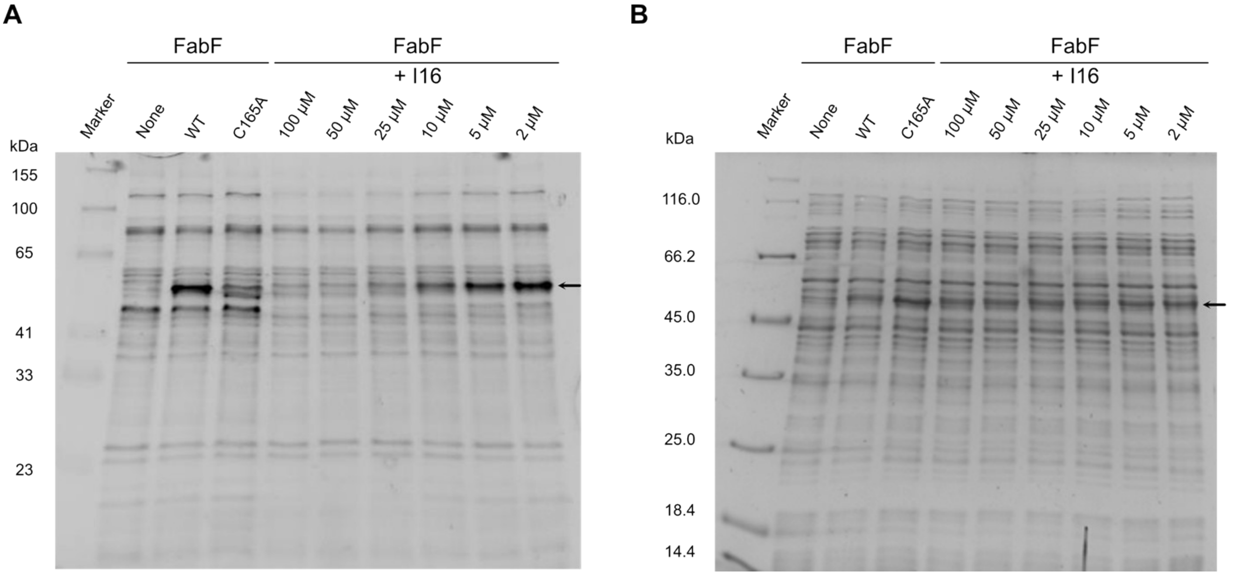

Figure S15. Gel-based labeling experiments with FabF. 2 µM recombinant wild-type (WT) FabF was added to 1 mg/mL soluble lysate of *S. aureus* NCTC 8325. As controls, 2 µM of the C165A mutant or no enzyme (None) were included. Samples were treated with the indicated concentrations of **I16** or DMSO, labeled with IA-alkyne (40 µM), and clicked to TAMRA-azide. SDS-PAGE was used for analysis, and the results of in-gel fluorescence scanning (A) and Coomassie staining (B) are shown.

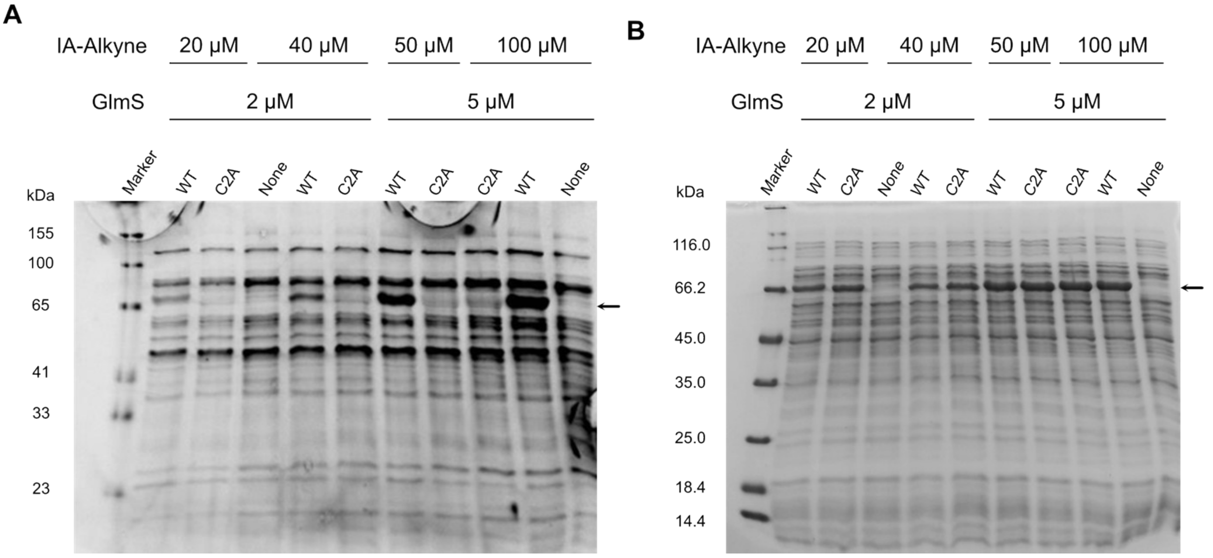

Figure S16. Labeling optimization with GlmS and its C2A mutant. Indicated concentrations of the recombinant wild-type (WT) GlmS and C2A mutant were added to 1 mg/mL soluble lysate of *S. aureus* NCTC 8325. As controls, lysates were included without adding the enzyme (None). Samples were labeled with indicated concentrations of IA-alkyne and clicked to TAMRA-azide. SDS-PAGE was used for analysis, and the results of in-gel fluorescence scanning (A) and Coomassie staining (B) are shown.

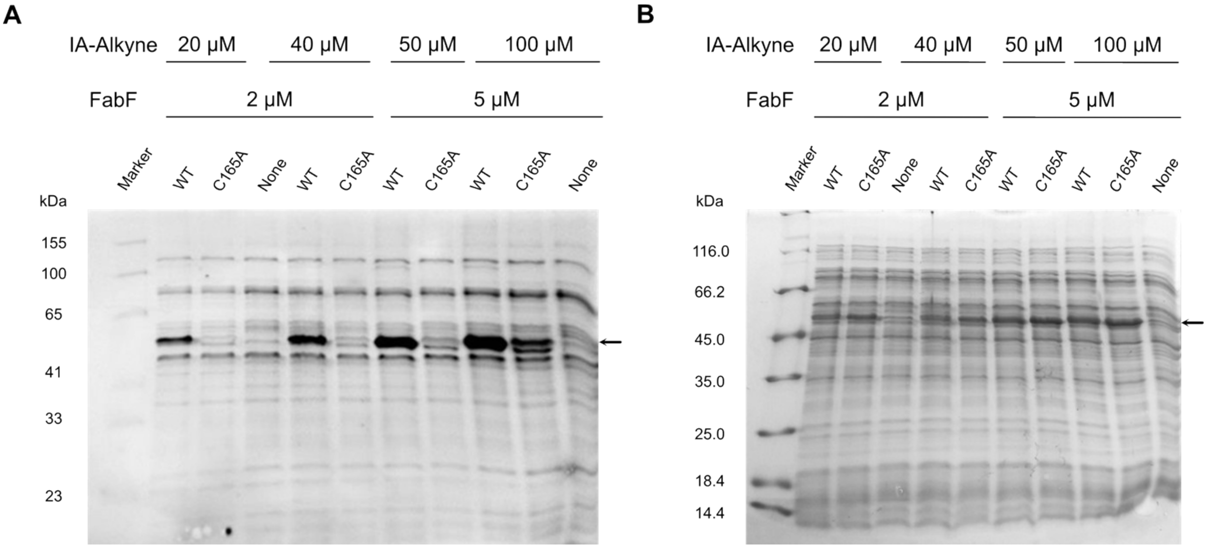

Figure S17. Labeling optimization with FabF and its C165A mutant. Indicated concentrations of the recombinant wild-type (WT) FabF and C165A mutant were added to 1 mg/mL soluble lysate of *S. aureus* NCTC 8325. As controls, lysates were included without adding the enzyme (None). Samples were labeled with indicated concentrations of IA-alkyne and clicked to TAMRA-azide. SDS-PAGE was used for analysis, and the results of in-gel fluorescence scanning (A) and Coomassie staining (B) are shown.

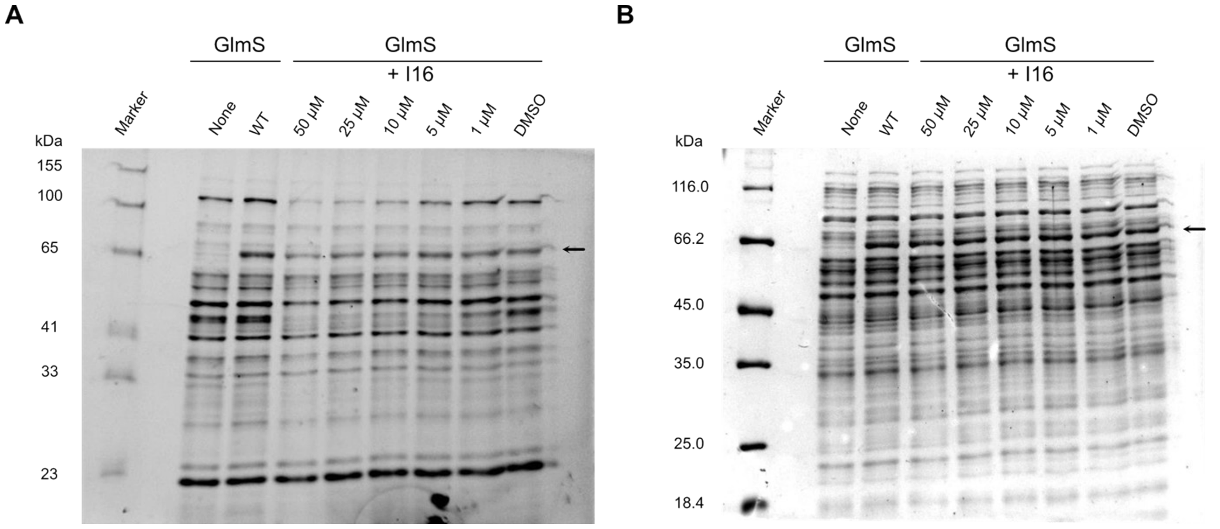

Figure S18. Additional results of gel-based labeling experiments with GlmS. 1 µM recombinant wild-type (WT) GlmS was added to 1 mg/mL soluble lysate of *S. aureus* NCTC 8325. As a control, lysate was included without adding the enzyme (None). Samples were treated with the indicated concentrations of **I16** or DMSO, labeled with IA-alkyne (10 µM), and clicked to TAMRA-azide. SDS-PAGE was used for analysis, and the results of in-gel fluorescence scanning (A) and Coomassie staining (B) are shown.

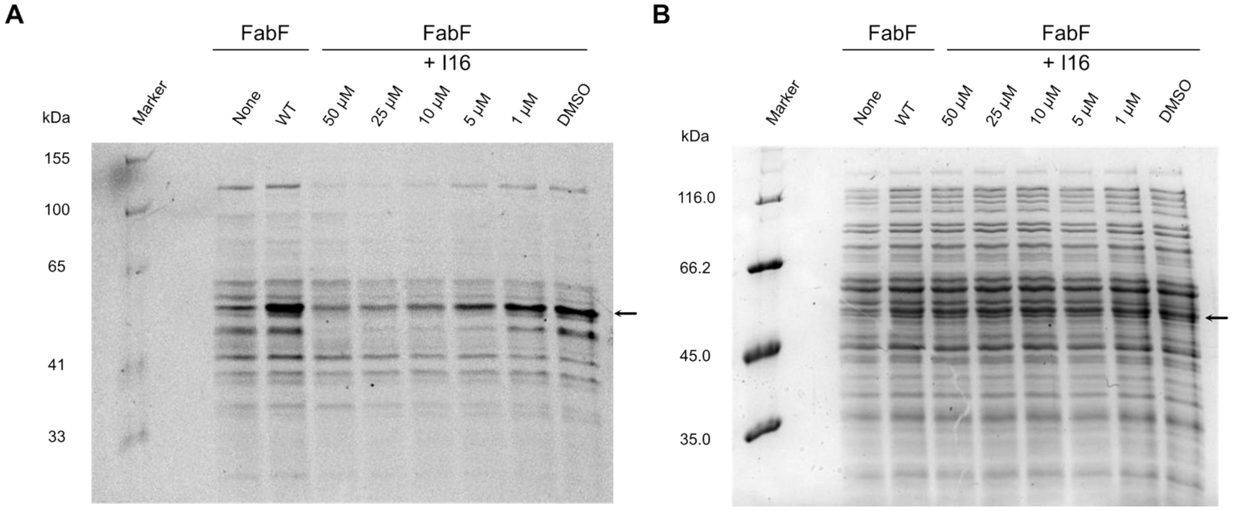

Figure S19. Additional results of gel-based labeling experiments with FabF. 1 µM recombinant wild-type (WT) FabF was added to 1 mg/mL soluble lysate of *S. aureus* NCTC 8325. As a control, lysate was included without adding the enzyme (None). Samples were treated with the indicated concentrations of I16 or DMSO, labeled with IA-alkyne (10 µM), and clicked to TAMRA-azide. SDS-PAGE was used for analysis, and the in-gel fluorescence scanning (A) and Coomassie staining (B) are shown.

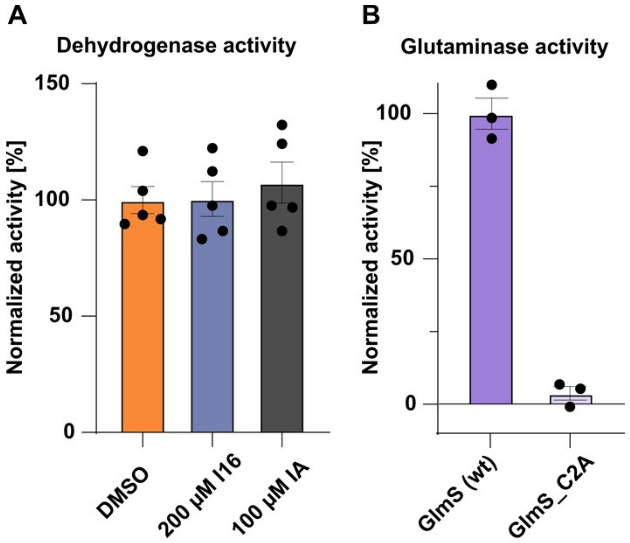

Figure S20. (A) Glutamate dehydrogenase (GDH) activity was determined in the presence of **I16** (200 µM), iodoacetamide (IA, 100 µM) or the vehicle control (3% DMSO). Data represent mean values ± SEM of n = 5 independent experiments normalized to the vehicle control. (B) The glutaminase activity of GlmS and the C2A mutant were measured in a GDH coupled enzyme assay. Data represent mean values ± SEM of n = 3 independent experiments normalized to the wild type.

Supplementary Tables

Table S1: Screening of commercial isocyanides for activity against *S. aureus* and *E. coli*. MICs of each compound were determined in technical triplicates at concentrations up to 50 µM. MIC values represent means of technical triplicates. The term “reduced growth” indicates an OD_{600} value < 20% compared to the growth control.

**Compound**	**Structure**	**MIC (µM)**	
		***S. aureus* USA 300 Lac J2**	***E. coli* K12**
**I1**	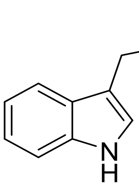	> 50	> 50
**I2**	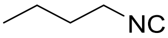	> 50	> 50
**I3**	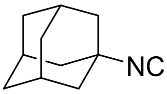	> 50	> 50
**I4**	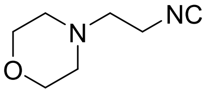	> 50	> 50
**I5**	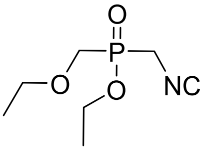	> 50	> 50
**I6**	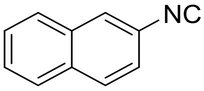	> 50	> 50
**I7**	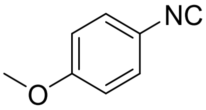	> 50	> 50
**I8**	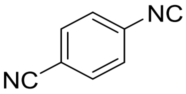	> 50	> 50
**I9**	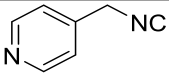	> 50	> 50
**I10**	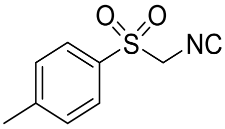	> 50	> 50
**I11**	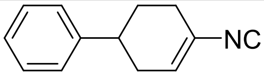	> 50	> 50

**I12**	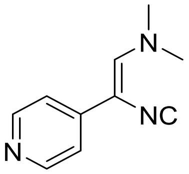	> 50	> 50
**I13**	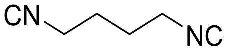	> 50	> 50
**I14**	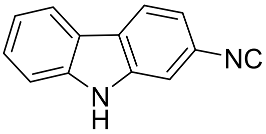	> 50	> 50
**I15**	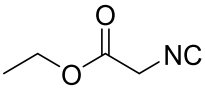	> 50	> 50
**I16**	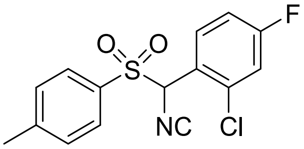	1.56	> 50
**I17**	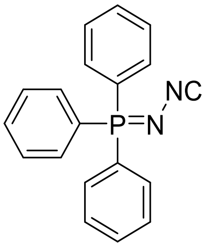	> 50	> 50
**I18**	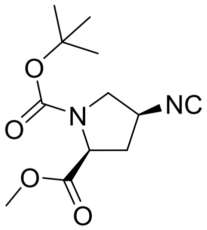	> 50	> 50
**I19**	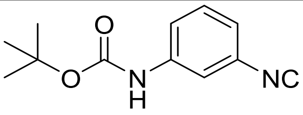	50 reduced growth	> 50
**I20**	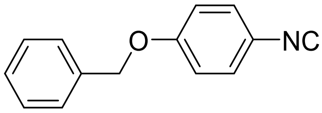	50	> 50
**I21**	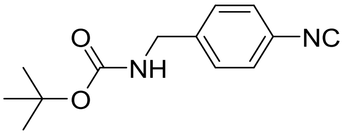	> 50	> 50
**I22**	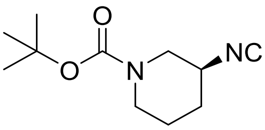	> 50	> 50

**I23**	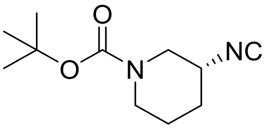	> 50	> 50
**I24**	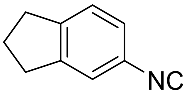	> 50	> 50
**I25**	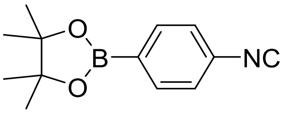	50 reduced growth	> 50
**I26**	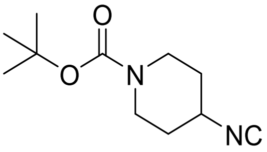	> 50	> 50
**I27**	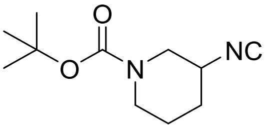	> 50	> 50
**I28**	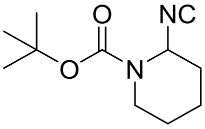	> 50	> 50
**I29**	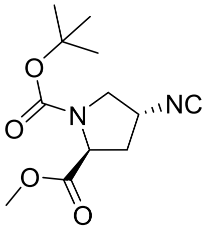	> 50	> 50
**I30**	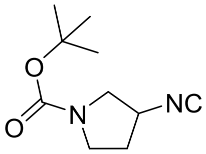	> 50	> 50
**I31**	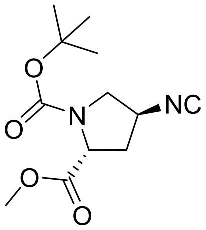	> 50	> 50
**I32**	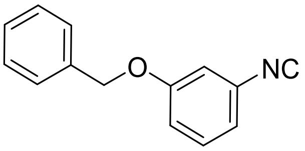	50 reduced growth	> 50

**I33**	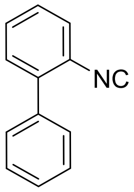	> 50	> 50
**I34**	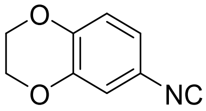	> 50	> 50
**I35**	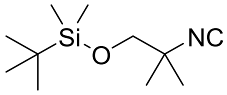	> 50	> 50
**I36**	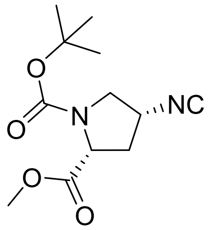	> 50	> 50
**I37**	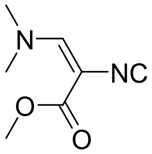	> 50	> 50
**I38**	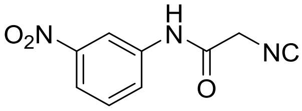	> 50	> 50
**I39**	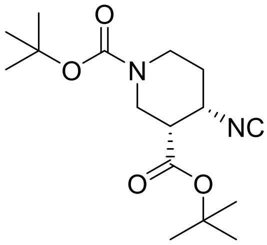	> 50	> 50
**I40**	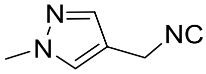	> 50	> 50
**I41**	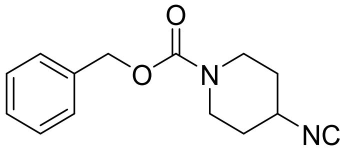	> 50	> 50
**I42**	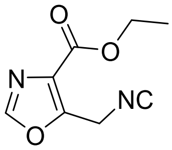	> 50	> 50

Table S2: Antibiotic activity of **I16** in different bacterial strains. Data represent means of technical triplicates and results were confirmed in at least n = 2 independent experiments. If the MIC varied between biological replicates, concentration ranges are indicated.

	**Strain**	**MIC (µM)**
gram-positive	*B. subtilis* 168	12.5
	E. faecalis V583	> 100
	E. faecium DSM17050	> 100
	L. monocytogenes EGD-e	1
	S. epidermidis RP62 A	1
	S. aureus NCTC 8325	0.78 - 1.56
	S. aureus USA300 LAC J2	1.56
	S. pneumoniae DSM 20567	1.56
	S. pyogenes ATCC 700294	> 100
gram-negative	*A. baumannii* ATCC 19606	> 100
	A. baumannii 5075	> 100
	E. coli K12	> 100
	E. coli UTI89	> 100
	K. pneumoniae DSM 30104	> 100
	P. aeruginosa PAO1	> 100
	S. typhimurium LT2	> 100

Table S3: Cytochrome P450 (CYP) inhibition. The inhibition of 4 CYP enzymes was tested in human liver microsomes and evaluated by monitoring the conversion of CYP isoform specific substrates. IC_{50} were determined both from mixtures of all substrates (n = 3) and against individual substrates (n = 1) resulting in no significant difference in inhibition. IC_{50} represent means of all replicates ± SD. The experiment was carried out in n = 3 biological experiments.

Isoform	Substrate	Metabolite	Compound and Control	IC_{50} [µM]
2C9	Diclofenac	4-OH-Diclofenac	**I16**	0.144 ± 0.053
			Sulfaphenazole	0.196 ± 0.085
2D6	Dextrometorphan	Dextrorphan	**I16**	0.0593 ± 0.020
			Quinidine	0.0109 ± 0.0039
1A2	Phenacetin	Paracetamol	**I16**	0.0608 ± 0.017
			Fluvoxamine	0.0126 ± 0.0053
3A4	Midazolam	1-OH-Midazolam	**I16**	0.0261 ± 0.008
			Ketoconazole	0.0855 ± 0.0171

Table S4: Antibiotic activity of **I16** in wild type *A. baumannii* (ATCC 19606) and in three xanthocillin resistant *A. baumannii* strains generated by Hübner et al.[2] Whole-genome sequencing data and metadata for the resistant strains are available on the SRA repository under the Bioproject number PRJNA639720.[2] Xan exhibits an MIC of 0.25-0.5 µM in wild type *A. baumannii*, whereas the MIC in the resistant strains is > 25 µM.[2] MICs of **I16** were determined in the presence of PMBN. Data represent means of technical triplicates and results were confirmed in n = 2 independent experiments.

Strain + 5 µg/mL PMBN	MIC (µM)
A. baumannii ATCC 19606 (wt)	25
Xan. res. *A. baumannii* ATCC 19606 – A.3	25
Xan. res. *A. baumannii* ATCC 19606 – B.3	25
Xan. res. *A. baumannii* ATCC 19606 – C.3	25

Table S5.

See separate Excel file. Full proteome analysis of **I16**-mutant *S. aureus* colonies A.1, B.1 and C.1 compared to *S. aureus* NCTC 8325 wt. Includes protein annotation, MS data, statistics, and LFQ intensities.

Table S6.

See separate Excel file. Full proteome analysis of **I16**-treated (3.13 µM) *S. aureus* NCTC 8325 cells compared to DMSO-treated cells. Includes protein annotation, MS data, statistics, and LFQ intensities.

Table S7: Calculated p-value, slope and curve fit for significantly stabilized and destabilized proteins after treating living *S. aureus* NCTC 8325 cells with **I16** (for significance criteria see data analysis for TPP experiments).

T_m shift repl. 1/2 [°C]	p-value adj. repl. 1/2	R^2 curve fit (**I16**-1/DMSO-1/**I16**-2/DMSO-2	Min. slope **I16** vs. DMSO repl. 1/2	Uniprot ID
14.99/15.76	4.7E-43/5.1E-49	0.99/0.91/1.00/0.88	-0.10/-0.13	Q2G2M8
8.37/8.43	4.0E-21/1.5E-13	0.99/0.98/1.00/0.99	-0.19/-0.12	Q2FY88
7.33/4.90	3.6E-16/1.9E-04	0.98/0.85/0.93/1.00	-0.16/-0.14	Q2FZ83
3.60/3.85	2.6E-02/9.0E-02	0.99/0.99/0.99/0.99	-0.10/-0.09	Q2G1T3
3.00/3.37	9.4E-02/3.4E-02	0.99/1.00/0.99/0.99	-0.10/-0.11	Q2FXK7
-2.83/-3.02	8.2E-02/9.0E-02	0.95/1.00/0.98/1.00	-0.15/-0.16	Q2FWC4
-4.12/-4.01	8.6E-03/5.2E-02	1.00/0.99/0.99/1.00	-0.11/-0.09	Q2FZS0
-4.94/-4.02	6.9E-05/6.4E-03	1.00/0.99/0.98/1.00	-0.25/-0.30	Q2G2P2
-5.13/-3.40	3.3E-05/3.5E-02	0.98/1.00/0.93/0.99	-0.58/-0.43	Q2G223

Table S8.

See separate Excel file. Proteomics data of competitive isoDTB labelling experiment. Includes identified peptide sequences, $log_{10}(2)$ transformed H/L ratios, annotations, and statistical analysis results.

Table S9: Hits of the competitive isoDTB experiments in living *S. aureus* NCTC 8325 bacteria.

Uniprot ID	Modified C	Gene/ORF	essential	Molecular function	$Log_2(R)$
Q2G198	C2	SAOUHSC_00247	no	hydrolase activity	2.10204
Q2FWA0	C2	glmS	yes	glutamine-fructose-6-phosphate transaminase (isomerizing) activity	2.17978
Q2FWD5	C4	SAOUHSC_02364	no	transcription regulation	1.9696
Q2FWC2	C63	SAOUHSC_02376	no	uncharacterized	1.79181
Q2FZR9	C165	SAOUHSC_00921 (FabF)	yes	3-oxoacyl-[acyl-carrier-protein] synthase activity	1.82601
Q2G1J0	C290	aldA	no	aldehyde dehydrogenase (NAD+) activity	1.63388
Q2G1A8	C229	SAOUHSC_00237	no	S-adenosylmethionine-dependent methyltransferase activity	1.65188
P0A0K3	C105	SAOUHSC_02013	no	uncharacterized	1.46549
Q2FWG2	C190	thiM	no	hydroxyethylthiazole kinase activity	1.35776
Q2FWD5	C32	SAOUHSC_02364	no	transcription regulation	1.32936
Q2FZW2	C51<br>C48	SAOUHSC_00873	no	iron-sulfur cluster assembly; [4Fe-4S] binding	1.27374<br>1.27526
Q2G1D0	C379	SAOUHSC_00195	no	acetyl-CoA C-acetyltransferase activity	1.23911
Q2G081	C50	queF	no	preQ1 synthase activity	1.03532
Q2FZX4	C68	lipA	no	lipoate synthase activity; [4Fe-4S] binding	1.17927
Q2G0B1	C12	mgrA	no	Transcription regulation	1.07577
P69848	C24<br>C278	moA	no	GTP 3',8'-cyclase activity; [4Fe-4S] binding	1.12888<br>1.12166

Methods

Bacterial culture conditions

For pre-cultures, 5 mL media were inoculated with 5 µL of a bacterial glycerol stock and grown at 37 °C, 200 rpm, for 11-18 h. *Streptococcus pneumoniae* DSM30104 and *Streptococcus pyogenes* ATCC700294 were cultured under 5% CO_2 atmosphere at 37 °C without shaking. A sterile control (medium containing no bacteria) was always included. Unless otherwise stated, modified lysogeny broth (B; 10 g/L casein peptone, 5 g/L NaCl, 5 g/L yeast extract, 1 g/L K_2HPO_4) was used for cultivation of all *S. aureus* strains. Lysogeny broth (LB; 10 g/L casein peptone, 5 g/L NaCl, 5 g/L yeast extract) was used for cultivation of *B. subtilis*, *E. coli*, *P. aeruginosa*, *A. baumannii* and *S. typhimurium* strains. *L. monocytogenes*, *K. pneumoniae*, *E. faecalis*, *E. faecium*. *S. epidermidis, S. pyogene*s and *S. pneumonia* strains were cultivated in brain heart infusion medium (BHB, 7.5 g/L brain infusion, 10 g/L heart infusion, 10 g/L casein peptone, 5 g/L NaCl, 2.5 g/L Na_2HPO_4, 2 g/L glucose).

Cell culture

Cell culture media and supplements were purchased from *Merck*. HeLa cells were cultured in Dulbecco's Modified Eagle Medium (DMEM, high glucose, 4.5 g/L) supplemented with 10% fetal bovine serum (FBS) and 2 mM L-glutamine at 37 °C in a 5% CO_2 atmosphere. Accutase (*Merck*) was used for cell detachment.

Minimum inhibitory concentration (MIC)

The minimum inhibitory concentration (MIC) was determined by using the broth microdilution assay. 1 µL of compound stock solution in DMSO was pipetted to the wells of row 1 of a sterile flat bottom 96-well plate containing 100 µL growth medium and thoroughly mixed. Subsequently, a 1:1 serial dilution was performed in the growth medium from row 1 to row 10. 50 µL of bacterial suspension (1:10,000 dilution of an overnight culture in the respective growth medium) was added to each well containing compound and the growth control (row 11). A sterile control containing only medium was included (row 12). The final volume was 100 µL per well. Plates were incubated for 20 h (37 °C, 200 rpm; for *Streptococcus pneumoniae* DSM30104 and *Streptococcus pyogenes* ATCC700294: 5% CO_2 atmosphere, 37 °C without shaking), and the dilution series were analyzed for microbial growth, indicated by turbidity and/or a pellet. The lowest concentration in the dilution series at which no growth of bacteria could be observed was defined as the MIC of the compound. MIC values were determined in technical triplicates and confirmed in two or three independent biological experiments. The isocyanide library was screened once in technical triplicates. The MIC is reported as mean of technical triplicates. If the MIC varied between biological replicates, concentration ranges are indicated.

For MIC assays in the presence of 5 µg/mL polymyxin B nonapeptide hydrochloride (PMBN purchased from *Merck*), a sterile 5 mg/mL stock solution in double-distilled water was 1:1000 diluted in the respective growth medium. This PMBN medium was further used for the dilution series of the compound and bacteria as described above.

For xanthocillin mutants, pre-cultures were grown in the presence of 500 nm xanthocillin, while MIC assays were performed in the absence of xanthocillin to avoid interfering effects.

DNA shift assay

The assay was adapted from a previously described protocol.[3] Low molecular weight salmon sperm DNA (*Merck*) was dissolved in TE buffer (10 mM Tris-HCl, 0.1 mM EDTA, pH 8). Serial dilutions of the test compound were performed analogously to the above-described MIC protocol in B medium with or without addition of DNA (0, 500, 250, 125, 50 µg/mL) in a sterile,

flat bottom 96-well plate. The plate was preincubated at 37 °C for 30 minutes, followed by the addition of 50 µL *S. aureus* NCTC 8325 bacterial suspension (1:10,000 dilution of an overnight culture in B medium). The plate was then incubated at 200 rpm and 37 °C for 20 h. The MIC was determined as described above. As a positive control, the DNA intercalator actinomycin D was included. Gentamycin served as a negative control. The experiment consisted of two independent biological replicates with technical quadruplicates.

Checkerboard assays

The checkerboard assay was performed with I16 and copper(II) sulfate. A sterile metal salt stock solution was prepared in double-distilled water. For each component of the checkerboard assay, a serial dilution series was prepared in B medium in a sterile 96-well plate. For **I16**, 2 µL of a 1.6 mM stock solution in DMSO were added to 200 µL medium, and for the metal salt, 20 µL of a sterile 240 mM stock in double-distilled water were added to 180 µL medium. For both compounds 1:1 dilution series were performed either from row to row or column to column. 25 µL from each well of the respective, individual compound plate were transferred to a new 96-well plate. The resulting checkerboard contained each combination of the two test molecules. To each well, 50 µL *S. aureus* NCTC 8325 bacterial suspension (1:10,000 dilution of an overnight culture in B medium) were added. Two-fold the MIC of each molecule was used as the highest concentration tested. The plate was incubated for 20 h (37 °C, 200 rpm). The lowest concentration in the dilution series without bacterial growth was defined as the MIC. The experiment consisted of three independent biological replicates.

To evaluate the interactions between both molecules, the fractional inhibitory concentration (FIC) index is used: $\Sigma FIC = FIC(A) + FIC(B) = A/MIC_A + B/MIC_B$. The FIC values for each component are determined by the compound's MIC in combination (A or B) divided by its MIC without presence of the second one (MIC_A or MIC_B). If the combination of both test substrates leads to a FIC index that is < 0.5, the inhibitory activity of one or both compounds is increased (synergy). Values ≥ 0.5 and < 4 are considered additive or indifferent and values > 4 show an antagonistic effect between the tested compounds.

Time-kill assay

The assay was adapted from previously described procedures.[2, 4] An overnight culture of *S. aureus* NCTC 8325 was diluted into fresh B medium to an OD_{600} = 0.025. Cells were grown to the mid-exponential phase (OD_{600} = 0.4-0.5; 37 °C and 200 rpm) and then diluted to $1x10^6$ CFU in B. Subsequently, the cells were further diluted 1:4 in fresh medium and split into 3 mL aliquots into culture tubes containing **I16** at 1x MIC (1.56 µM, 1% DMSO final concentration), at 16x MIC (25 µM, 1% DMSO final concentration) and DMSO (1%). Cells were incubated (37 °C, 200 rpm) and serial dilutions were plated on B agar plates at indicated time points for the determination of viable cells (CFU/mL). Three independent biological replicates were analyzed. CFU determination for the inoculum was performed in quadruplicates. Three replicates were considered at indicated time points.

A shortened version of this experiment was performed to evaluate if a ROS scavenger can ensure bacterial survival or reestablish bacterial growth. For this purpose, bacteria were diluted to $1x10^6$ CFU in B medium supplemented with 100 mM thiourea (TU) and then further diluted 1:4 into the same medium. 3 mL aliquots were added into culture tubes containing **I16** at 16x MIC (25 µM, 1% DMSO) and DMSO (1%). Cells were incubated (37 °C, 200 rpm) and serial dilutions were plated on B agar plates after 24 h for the determination of viable cells (CFU/mL). Three independent biological replicates were analyzed. CFU determination for the inoculum was performed in quadruplicates. Three technical replicates were considered after 24h.

Membrane potential assay

The experiment was adapted from a previously published protocol.[5] An overnight culture of *S. aureus* NCTC 8325 was diluted 1:100 into fresh B medium. Cells were grown to an OD_{600} = 0.3 (37 °C, 200 rpm). 100 µL aliquots of the cell suspension were transferred to a black 96-well plate. Fluorescence measurements were performed in an Infinite M200 Pro microplate reader (*Tecan Group*) with λ_{ex} = 610 nm and λ_{em} = 660 nm at 37 °C. First the background fluorescence was recorded for 3 minutes, then 1 µL of 100 µM 3,3'-dipropylthiadicarbo-cyanide iodide (DiSC3(5)) in DMSO was added to each well (1 µM final concentration). The integration into the cell membrane was ensured for 10 minutes, while measuring the fluorescence. 1 µL of DMSO, **I16** compound stock (1x MIC, 2x MIC, 8x MIC, 16x MIC final concentration) or the positive control gramicidin (1 µM final concentration) was added and the fluorescence recorded for another hour. Fluorescence quenching by the compound in the absence of bacteria in sterile B medium was excluded before performing the experiment. The experiment was performed in triplicates and repeated twice.

Membrane integrity assay

As described previously[4], an overnight culture of *S. aureus* NCTC 8325 was diluted into fresh B medium (1:100) and grown to an OD_{600} value of 0.7 (37 °C, 200 rpm). The cells were harvested (6,000 x g, 4 °C, 5 min), washed with 5 mM HEPES-NaOH buffer (pH 7.2, supplemented with 5 mM glucose) and then resuspended in the same buffer to an OD_{600} of 0.4. 100 µL aliquots of the cell suspension were transferred to a black 96-well plate. Fluorescence measurements were performed in an Infinite M200 Pro microplate reader (*Tecan Group*) with λ_{ex} = 535 nm and λ_{em} = 617 nm at 37 °C. First the background fluorescence was recorded for 3 minutes, then 1 µL of 1 mM propidium iodide in DMF was added to each well (10 µM final concentration). The plate was incubated for 15 minutes while measuring the fluorescence. Next, 1 µL of **I16** compound stock (1x MIC, 2x MIC, 16x MIC final concentration) was added. As a positive control, 100 µg/mL of the detergent benzalkonium chloride (BAC) were added to the cells, while 1 µL DMSO was added for a baseline fluorescence control. The fluorescence was recorded for 45 minutes. As a negative control, the assay was also conducted in assay buffer without any cells. The experiment was performed in triplicates.

Isothermal titration calorimetry (ITC)

ITC measurements were conducted on a MircoCal PEAQ-ITC instrument (*Malvern*) at 25 °C. Each experiment consisted of 19 injections with an injection length of 4.2 seconds and an interval of 150 seconds (initial injection with 0.4 µL followed by 18 injections with 2.1 µL). It was constantly stirred with a speed of 750 rpm. 50 mM Tris/HCl (pH 7.4, 10% DMSO) was used as assay buffer and all solutions were prepared with this buffer. 2 mM copper (II) chloride was titrated to 200 µM **I16** or the known copper ionophore elesclomol in the cell. The K_D value and error were determined with the MicroCal PQAQ ITC analysis software. The data points were fitted to a single binding site model and a fitted offset of the control experiment was used as control parameter. As control experiment, the copper solution was titrated to buffer. Each experiment was repeated twice.

UV-vis spectroscopy

The assay was performed as previously described.[2] In a transparent 96-well plate (transparent Nunc 96-well flat bottom, *Thermo Fisher Scientific*), 4 µL 1 mM hemin in 0.1 N NaOH were dissolved in 196 µL 200 mM HEPES, pH 7.0 (20 µM final concentration). 4 µL of DMSO, 1 mM xanthocillin in DMSO or 1 mM **I16** in DMSO were added (20 µM final concentration each). The plate was then incubated for 1h at room temperature in the dark without shaking. The UV-vis spectrum was recorded with an Infinite M200 Pro microplate reader (*Tecan Group*). The

respective compound, xanthocillin or **I16**, in buffer served as blank control. The assay was performed in three independent experiments consisting of two technical replicates each.

Elemental analysis by inductively coupled plasma mass spectrometry (ICP-MS)

The protocol was adapted from a previously published procedures.[6, 7] 500 µL of a *S. aureus* NCTC 8325 pre-culture were used to inoculate 50 mL B medium in 250 mL baffled flasks. 50 µL of DMSO (final: 1%) or 100x compound stock (final concentration: 3.13 µM) were added and cultures were grown to mid-exponential phase (200 rpm, 37 °C). 40 mL of bacterial cultures at an OD_{600} of 3.0 were harvested (6000 x g, 10 min, 4 °C) and subsequently washed with 10 mL 10 mM EDTA (pH 8.0) and twice with 10 mL 20 mM Tris buffer (pH 7.5). Extra liquid was thoroughly removed, and the dried pellets were lysed through addition of 1 mL of 69% nitric acid (trace metal grade, *Merck*) and microwave treatment (*Anton Paar Microwave 5000*). Digested cells were then diluted 1:10 and analyzed by using an inductively coupled plasma mass spectrometer (Nexion 350D, *Perkin Elmer*). The experiment consists of three biological replicates.

MTT assay

To assess the impact of I16 on the metabolic activity of human cells, the MTT assay was performed in HeLA cells. Cells were seeded at a density of 4000 cells/well into a 96-well plate (transparent Nunc 96-well flat bottom, *Thermo Fisher Scientific*) and grown for 24h. Subsequently, the medium was carefully removed and 100 µL of medium with DMSO (1%) or a concentration range of compound (1% final DMSO concentration) was added. After 24h incubation (37 °C, 5% CO_2), 20 µL of thiazolyl blue tetrazolium bromide (MTT, 5 mg/mL in PBS, *Merck*) were added and cells were again incubated for further 4h (37 °C, 5% CO_2) to allow the metabolization of MTT to formazan. After removal of the medium, the resulting formazan crystals were dissolved in 200 µL DMSO. The optical density was measured at 570 nm with background subtraction at 630 nm with an Infinite M200 Pro microplate reader (*Tecan Group*). The measured values were normalized to the DMSO-treated controls (100% metabolic activity). IC_{50} values and 95% confidence intervals were calculated with GraphPad Prism (Version 10.1.0). The experiment was performed in two independent experiments consisting of six technical replicates each.

CYP inhibition

The inhibition of 4 CYP enzymes was tested in human liver microsomes (HLM, Xenotech, Kansas City, USA) monitoring the conversion of the following reference substrates to the corresponding metabolites: midazolam > 1-OH-midazolam (CYP3A4), phenacetin > paracetamol (CYP1A2), dextrometorphan > dextrorphan (CYP2D6), diclofenac > 4-OH-diclofenac (CYP2C9). Ketoconazole, fluvoxamine, quinidine and sulfaphenazole were used as reference inhibitors, respectively. Test compound and control inhibitors were serially diluted in 19% MeCN / 1% DMSO / PBS, followed by 1:10 dilution into the incubation mixture and incubated at 37°C for 10 min, 700 rpm on a microplate shaker (*Eppendorf*, Hamburg, Germany). Substrates were added by 1:10 dilution from 20% acetonitrile/PBS and the mixture incubated at 37°C for 60 min, 700 rpm. Final incubation conditions were 0.1 mg/mL HLM, 1 mM NADPH, 10 mM $MgCl_2$ in 100 mM potassium phosphate buffer pH 7.4, 0.1 µM substrate, test compound concentration range 0.01–10 µM or 0.001–1 µM. The incubation was stopped by adding aliquots to 2 volumes of cold internal standard solution (15 nM diphenhydramine in 10% MeOH/acetonitrile). Precipitated protein was removed by centrifugation (15 min, 4 °C, 4,000 x g), and the amount of metabolite and parent compound analyzed by HPLC-MS/MS (Vanquish Flex coupled to a TSQ Altis Plus, *Thermo Fisher*, Dreieich, Germany). HPLC

conditions were as follows: eluent A: H_2O + 0.1% formic acid, eluent B: acetonitrile + 0.1% formic acid, flow: 0.7 mL/min. The gradient was set to 10% B from 0 to 0.2 min, 10-40% B from 0.2 to 0.5 min, 40% B from 0.5 to 2.7 min, 40 to 90% B from 2.7 to 4.0 min, 90% B from 4.0 to 4.5 min and 10% B from 4.5 to 5.0 min. MS parameters: Spray voltage 2600 V (positive mode), vaporizer temperature 350 °C, ion transfer tube temperature 380 °C, sheath gas 30 (arb), auxiliary gas 10 (arb), ion sweep gas 2 (arb). Mass transitions for the metabolites were as given in the table below. Percentage inhibition at the respective concentration was calculated relative to the amount of metabolite formed in blank samples containing no inhibitor. IC_{50} was calculated using the nonlinear regression function of GraphPad Prism 10 (GraphPad Software, San Diego, CA, USA). IC_{50} were determined both from mixtures of all substrates (n = 3) and against individual substrates (n = 1) resulting in no significant difference in inhibition.

Table S10: MS settings for the detection of CYP metabolites.

Metabolite	Precursor (m/z)	Product (m/z)	Collision energy (V)	RF lens (V)
Paracetamol	152	92<br>110	22<br>16	50
Dextrorphan	258	133<br>157	31<br>40	65
1-OH-midazolam	342	168<br>297	41<br>29	70
4-OH-diclofenac	312	230<br>294	34<br>10	48

Generation and sequencing of I16 resistant *S. aureus* NCTC 8325

The protocol was adapted from previously reported procedures.[2,4] For resistance development by sequential passaging, a 96-well set-up was chosen. An overnight culture of *S. aureus* NCTC 8325 was adjusted to OD_{600} = 1 and subsequently diluted 1:50 in fresh B medium. In a sterile 96-well plate, a dilution series of **I16**, ofloxacin (control), or DMSO (growth control) was performed in B-medium (see MIC protocol above). 50 µL bacterial inoculate were added and the cells were incubated (37 °C, 200 rpm) and passaged in 20-24 h intervals to a fresh compound plate. Cultures from the second lowest concentration that allowed growth (0.25 x MIC) were diluted 1:50 into fresh media and used as inoculum. The serial passaging was repeated for 30 days in three independent biological replicates. The MIC shifts were calculated by dividing the respective daily MICs by the respective initial MIC.

For sequencing, clones of **I16** resistant mutants and of the wild-type laboratory strain were isolated. From the last passaging plate, cells from the second lowest concentration that allowed growth (0.25 x MIC) were diluted 1:10^9 and plated on B-agar plates (supplemented with **I16**). For the wild-type control, the cryostock of the laboratory strain was streaked on a B-agar plate. Plates were incubated over night at 37 °C. Three colonies of each independent biological experiment (A.1, A.2, A.3 (replicate A); B.2, B.2, B.3 (replicate B); C.1, C.2, C.3 (replicate C)), the DMSO control (D.1, D.2, D.3), and the laboratory strain (Wt.1, Wt.2, Wt.3) were isolated and subjected to genome sequencing.

DNA was extracted and purified from 300 mg wet weight of the *S. aureus* NCTC 8325 bacterial cell pellets from centrifuged from overnight independent BHB liquid cultures inoculated with the 12 colonies of the experimental and technical biological replicates described above. For total DNA extractions we used the DNeasy Blood & Tissue kit (Qiagen) following manufacturer's instructions. DNA sequencing libraries were prepared using the NEBNext Ultra II FS DNA Library kit with fragment size selection (200 bp - 450 bp) following manufacturer's instructions. The genomic DNAs were sequenced using MiSeq Reagent Kits v3 (2x300bp) on an Illumina MiSeq platform. Raw sequencing data derived from each independent colony are

available on the NCBI SRA repository under BioProject number 1088756 https://www.ncbi.nlm.nih.gov/bioproject/1088756

To determine mutations of the isocyanide resistant derivatives vs the wild type, raw reads were filtered and trimmed for quality using Trimmomatic v0.39 using as parameters ILLUMINACLIP:TruSeq3-PE.fa:2:30:10:2:True LEADING:3 TRAILING:3 MINLEN:120. A consensus sequence of the resulting assembly combining all the raw genomic data obtained from the wt colonies mapped to the reference genome sequence of strain NCTC 8325 was generated using Bowtie2 with the following parameters bowtie2-build-s input.fasta myIndex bowtie2-align-s -I 0 -X 800 -p 8 --sensitive -q -x myIndex -1 forwardReads.fastq -2 reverseReads.fastq -S output.sam and used to identify already present mutations due to genetic drift of the NCTC 8325 strain maintained in our lab. This consensus sequence was used to detect the new mutations in the genomes of the colonies after the resistance adaptation experiment. Mapping of the filtered/trimmed BBMerge paired reads of the resistant colonies to the wild type consensus was used to determine mutations and referred to the coordinates of the reference annotated nucleotide sequence for strain NCTC 8325, GenBank accession number NC_007795. The coverage obtained per colony sequenced was on average 161-fold +/-8.2 of the reference 2.8 Mb genome. SNPs and Indels were found using Geneious Prime 2023.0 variant finder with default values generating the corresponding type of mutation, coordinates to reference genome, amino acid, codon changes when found in a protein coding region, coverage, polymorphism type, and variant frequency in the dataset.

Full proteome analysis

For the analysis of the proteome changes under compound stress, *S. aureus* NCTC 8325 precultures were diluted 1:100 into fresh B medium containing **I16** (3.13 µM, 1% DMSO final concentration) or DMSO (1%). For the proteome analysis of **I16**-resistant mutants, precultures of *S. aureus* mutants (A.1, B.1, C.1) grown in presence of 1.56 µM **I16** (1% DMSO final concentration) and the wild type control were diluted 1:100 into fresh B medium. Cultures were grown to OD_{600} = 1.5 (37 °C, 200 rpm) and harvested (4 mL, 6,000 x g, 4 °C, 10 min). Pellets were washed with PBS (500 µL, 6,000 x g, 4 °C, 10 min), air dried and stored at -80 °C. Cells were lysed after resuspension in 1 mL lysis buffer (0.4% (w/v) sodium dodecyl sulfate (SDS) in PBS, supplemented with EDTA-free protease inhibitor cocktail (*Roche*)) by bead disruption (3 x 6,500 rpm, 30 s, 30 s cooling breaks with liquid nitrogen; Precellys Ceramic Kit CK01L, 2.0 mL tubes; Precellys 24 Homogenizer, *Bertin Technologies*). Lysates were cleared by centrifugation (21,000 x g, 4 °C, 15 min) and a defined protein amount per sample (50 µg for the mutant analysis, 80 µg for the stress analysis; determined by using the biuret assay (ROTI Quant universal, *Roth*)) was precipitated using 4 volumes of acetone (-20 °C, 1 h). The precipitated protein was pelletized (16,900 x g, 4 °C, 15 min), washed with cold methanol (2 x 1 mL) with resuspension by sonication (10 s, 10% intensity; Sonopuls HD 2070 ultrasonic rod Sonicator, Bandelin electronic). The air-dried pellets were dissolved in 200 µL denaturation buffer (7 M urea, 2 M thiourea in 20 µM HEPES buffer, pH 7.5). After reduction with 2 µL tris(2-carboxyethyl)phosphine (TCEP, 500 mM, 1 h, 37 °C, 800 rpm), the samples were alkylated with 4 µL 2-iodoacetamide (IAA. 500 mM, 30 min, 25 °C, 800 rpm) and finally quenched with 4 µL dithiothreitol (DTT, 500 mM, 30 min, 25 °C, 800 rpm). The samples were diluted with 600 µL triethylammonium bicarbonate (TEAB) buffer (50 mM in ddH2O) for the enzymatic digestion with 2 µL trypsin (0.5 µg/µL in 50 mM acetic acid, *Promega*; 16 h, 37 °C, 800 rpm). The digest was stopped by adding 10 µL formic acid (FA). The samples were centrifuged (13,000 x g, RT, 3 min) and the supernatants loaded on 50 mg SepPak C18 columns (Waters), equilibrated with acetonitrile and 0.1% trifluoroacetic acid (TFA). Peptides were washed three times with 1 mL 0.1% TFA and once with 500 µL 0.1% FA before being eluted with three times 250 µL elution buffer (80% acetonitrile, 0.5% FA). Desalted peptides were dried using a centrifugal vacuum concentrator (*Eppendorf*), then reconstituted in 100 µL 1% FA. The samples were filtered through 0.22 µM Ultrafree-MC centrifugal filters (3,000 x g, 3 min; UFC30GVNB, *Millipore*), which were equilibrated with 300 µL 1% FA. Filtrates were transferred

to MS vials and stored at -20 °C until LC-MS/MS measurement. The experiment was performed in n = 4 independent experiments.

MS-measurements were performed on an Orbitrap Fusion instrument (*Thermo Fisher Scientific*) coupled to an UltiMate 300 nano HPLC system (*Thermo Fisher Scientific*), using an Acclaim C18 PepMap100 75 µm ID x 2 cm trap column (*Thermo Fisher Scientific*) and an Aurora series AUR2-25075C18A separation column (75 µm ID x 25 cm, *IonOpticks*) under constant heating to 40 °C. Samples were first loaded on the trap column with 0.1% TFA (7 min, 5 µL/min flow rate) and were then transferred to the separation column. The separation method was run using buffer A (0.1% FA in water) and buffer B (0.1% FA in acetonitrile) at a flow rate of 400 nL/min. The 152 min gradient consisted of the following steps: 7 min B at 5%, over 105 min gradient increase of B to 22%, over 10 min to 32%, over 10 min to 90%, wash step for 10 min at 90%, decrease to 5% in 0.1 min and equilibration at 5% for 9.9 min. The peptides were ionized at 1.8 kV and transferred with a capillary temperature of 275 °C. MS data on the Orbitrap Fusion instrument were acquired in data-dependent mode with a full cycle time of 3 s. Full scans were acquired in the orbitrap at a resolution of R = 120,000. The automatic gain control (AGC) ion target was set to 2×10^5 and the scan range was 300-1,500 m/z with a maximum injection time of 50 ms. Fluoranthene cations (Easy-IC) were used for internal calibration. Monoisotopic precursor selection as well as the dynamic exclusion (mass tolerance of 10 ppm low/high) for 60 s was enabled. Precursors with charge states 2-7 and intensities greater than 5×10^3 were selected for fragmentation with the higher-energy collisional dissociation (HCD) cell at 30% collision energy. The isolation was performed with the quadrupole with a window of 1.6 m/z. MS2 scans were recorded in the ion trap using the rapid scan rate. The AGC target was set to 1×10^4 with a maximum injection time of 35 ms.

MaxQuant (version 2.1.0.0) was used to analyze the raw files with the Andromeda search engine using the following parameters for a label free quantification (LFQ)[8]: fixed modifications: carbamidomethylation (cysteine); variable modifications: oxidation (methionine), acetylation (N-terminus); proteolytic enzyme: trypsin/P; max. missed cleavages: 2; 'No fractions', 'LFQ', 'Requantify', 'Match between runs' and 'Second Peptide' were enabled. Other default settings were not changed. Searches were performed with the UniProt database for *S. aureus* NCTC 8325 (taxon identifier: 93061; downloaded on 21.06.2022).

Statistical analysis was performed with Perseus 1.6.15.0.[9] LFQ intensities were log2(x)-transformed. Putative contaminants, reverse peptides, and peptides only identified by site were removed. Data was annotated (categorical derived from data banks; in rows depending on the treatment/control) and filtered for four valid values in at least one group. Missing values were imputated separately for each column. For statistical evaluation, a two-sided two-sample Student's t-test with permutation-based FDR (0.05) was performed. The significance cut-off was set at p-value = 0.05 ($-\log_{10}$(p-value) = 1.3) (or T-Test significant) and an enrichment factor of 2 ($\log_2(x) = 1$) (mutant analysis) or 1.6 ($\log_2(x) = 0.7$) (treated wild-type analysis) as indicated in the plots.

Protein-protein association networks and functional enrichment analyses were performed using the STRING database version 12.0.[10, 11] Proteins fulfilling the significance criteria were submitted to the "multiple proteins" search option. As network type, the "full STRING network" was chosen. The network edges were set to "evidence" and sources included "Textmining", "Experiments" and "Databases". For the displayed interactors, only the query proteins were allowed and the minimum required interaction score was set to "medium confidence (0.400)". Functional enrichments for biological processes from Gene Ontology (GO) and local network clusters (STRING) were analyzed.

isoDTB-ABPP experiments in living bacteria

The experiment was adapted from published procedures.[12, 13] *S. aureus* NCTC 8325 precultures were diluted 1:100 into fresh B medium and grown to the early stationary phase. Cells were harvested (25 mL, 6,000 x g, 4 °C, 10 min) and then washed with PBS (25 mL,

6,000 x g, 4 °C, 10 min). Cells were resuspended in PBS and adjusted to OD_{600} = 40. 1 mL cell aliquots were treated with 10 µL 10 mM **I16** (in DMSO) or 10 µL DMSO and incubated for 1 h (37 °C, 200 rpm). Bacteria were harvested (6,000 x g, 4 °C, 10 min) and washed with PBS (2 x 1 mL, 6,000 x g, 4 °C, 10 min), prior to storage at -80 °C. For lysis, the pellets were resuspended in 1 mL PBS and 5 µL lysostaphin (10 mg/mL) were added. The samples were incubated for 1 h (37 °C, 1400 rpm), then 20 µL 20% (w/v) SDS were added, and sonication was performed (20 s at 20% intensity, HD 2070 ultrasonic rod Sonicator, *Bandelin electronic*). The lysates were cleared by centrifugation (21,000 x g, 1 h, 20 °C). The protein concentration was determined (ROTI Quant universal, *Roth*) and adjusted to 1 mg/mL. 1 mL aliquots of the adjusted samples were treated with 20 µL iodoacetamide-alkyne (5 mM stock in DMSO for a final concentration of 100 µM) and incubated for 1 h (20 °C, 1400 rpm). The samples were clicked to the heavy (DMSO-treated) and light (compound-treated) isoDTB-tags by adding 120 µL of the following mixture: 60 µL TBTA ligand (0.9 mg/mL in 4:1 t BuOH/DMSO), 20 µL copper sulfate (50 mM in ddH_2O), 20 µL TCEP (13 mg/mL in ddH_2O) and 20 µL of the respective isoDTB tag (5 mM stock in DMSO). After incubation at room temperature for 1 h, 1 mL of the compound treated sample and 1 mL of the DMSO treated sample were combined in a falcon tube and 8 mL cold acetone were added for protein precipitation. Precipitates were stored at -20 °C overnight. The precipitated protein was pelletized (3,500 x g, 10 min), washed with cold methanol (2 x 1 mL) with resuspension by sonication (10 s, 20% intensity; Sonopuls HD 2070 ultrasonic rod Sonicator, Bandelin electronic).

The pellets were dissolved in 300 µL denaturation buffer (8 M urea, 0.1 M TEAB in H_2O). 900 µL 0.1 M TEAB were added to obtain a concentration of 2 M urea. The sample solutions were added to 1.2 mL of washed high-capacity streptavidin agarose beads (50 µL initial volume; 10733315, *Fisher Scientific*) in 0.2% NP40 in PBS. The samples were incubated at room temperature for 1 h under continuous rotation. The samples were centrifuged (1,000 x g, 1 min) and the supernatant was removed. The beads were resuspended in 600 µL 0.1 % NP40 in PBS and transferred to centrifuge columns (1894131, *Fisher Scientific*). Beads were washed with 2 x 600 µL 0.1% NP40 in PBS, 3 x 600 µL PBS and 3 x 600 µL H_2O. After resuspension in 600 µL denaturation buffer (8 M urea, 0.1 M TEAB in H_2O), the beads were transferred to a fresh polypropylene centrifuge tube. The samples were centrifuged (1,000 x g, 1 min) and the supernatant was removed, before adding again in 300 µL fresh denaturation buffer (8 M urea, 0.1 M TEAB in H_2O). After reduction with 15 µL DTT (31 mg/mL in H_2O, 45 min, 37 °C, 200 rpm), proteins were alkylated with 15 µL IAA (74 mg/mL in H_2O, 30 min, rotation at room temperature), and the alkylation was quenched by adding 15 µL DTT (31 mg/mL in H_2O, 30 min, 37 °C, 200 rpm). Samples were diluted with 900 µL 0.1 M TEAB and centrifuged (1,000 x g, 1 min). The supernatant was removed, and beads were taken up in 200 µL buffer (2 M urea, 0.1 M TEAB in H_2O). Enzymatic digestion was started by adding 4 µL trypsin (0.5 µg/µL in 50 mM acetic acid, *Promega*; 16 h, 37 °C, 200 rpm). The following day, samples were diluted with 400 µL 0.1% NP40 in PBS and transferred to centrifuge columns (1894131, *Fisher Scientific*). Beads were washed with 3 x 600 µL 0.1% NP40 in PBS, 3 x 600 µL PBS and 3 x 600 µL H_2O. Peptides were eluted into low-bind centrifuge tubes with 1 x 200 µL and 2 x 100 µL 0.1% TFA in 50% acetonitrile, followed by a final centrifugation step (3,000 x g, 3 min). The samples were dried using a centrifugal vacuum concentrator (*Eppendorf*). Peptides were reconstituted in 30 µL 0.1% TFA, filtered through 0.22 µM Ultrafree-MC centrifugal filters (3,000 x g, 3 min; UFC30GVNB, *Millipore*), which were equilibrated with 300 µL 0.1% TFA. Filtrates were transferred to MS vials and stored at -20 °C until LC-MS/MS measurement. The experiment was performed in n = 4 independent experiments.

MS-measurements were performed on a Qexactive Plus mass spectrometer (*Thermo Fisher Scientific*) coupled to an UltiMate 300 nano HPLC system (*Thermo Fisher Scientific*), using an Acclaim C18 PepMap100 75 µm ID x 2 cm trap column (*Thermo Fisher Scientific*) and an Aurora series AUR2-25075C18A separation column (75 µm ID x 25 cm, *IonOpticks*) under constant heating to 40 °C. 5 µL of the samples were first loaded on the trap column with 0.1%

TFA (7 min, 5 µL/min flow rate) and were then transferred to the separation column. The separation method was run using buffer A (0.1% FA in water) and buffer B (0.1% FA in acetonitrile) at a flow rate of 400 nL/min. The 152 min gradient consisted of the following steps: 7 min B at 5%, over 105 min gradient increase of B to 40%, over 10 min to 60% B, over another 10 min to 90% B, 90% B plateau for 10 min, decrease to 5% B in 0.1 min and equilibration at 5% for 9.9 min. The peptides were ionized at 1.8 kV and transferred with a capillary temperature of 275 °C. The mass spectrometer was run in a TOP10 data-dependent mode. MS full scans were collected in the orbitrap in a scan range of 300-1500 m/z at a resolution of R = 70,000, an AGC target of 3×10^6 and a maximum injection time of 80 ms. The most intense peaks were selected for MS2 measurement with a minimum AGC target of 1×10^3. Isotopic exclusion and dynamic exclusion (60 s) were enabled and peaks with unassigned charge or a charge of +1 were excluded. Peptide match was 'preferred'. MS2 spectra were collected at a resolution of R = 17,500, the AGC target set to 1×10^5 and a maximum injection time of 100 ms in the orbitrap. The isolation was performed in the quadrupole with a window of 1.6 m/z. Fragments were generated using HCD (normalized collision energy: 27%).

A FASTA database for S. aureus NCTC 8325 (Taxonomy ID: 93061) was obtained from uniprot.org. Quantification with MaxQuant[14] was performed according to a published procedure[12], using a workaround to allow quantification of peptides containing one or more carbamidomethylated cysteines in addition to the isoDTB tag-labelled cysteine. In brief, in this workaround "U" (usually signifying selenocysteine) is used as a substituent amino acid for the modified cysteine. To accomplish this, all selenocysteine-containing proteins were deleted from the FASTA database. Then, each cysteine in the file was individually replaced with a "U" generating n different sequences with a single "U" for a protein with n cysteines. For each individual replacement, an entry in the FASTA database was created, named in the format "UniProt code"_"C""number of the cysteine". The unmodified sequence was deleted from the FASTA database, except if the protein did not contain any cysteine. In this case, the unmodified entry was renamed to "UniProt Code""C0" and kept in the database. Thus, a unique sequence was created, in which the modified cysteines were marked as "modified" (by being replaced by the placeholder "U") and all other cysteines were marked as "unmodified" (remaining "C" in the database). Therefore, it was ensured that there is always only one modified cysteine in each peptide to be detected and quantified. This allows also to detect and quantify all peptides that contain several cysteines but are only modified with IA.Alkyne and the isoDTB tags at one of them. MS raw data were analyzed using MaxQuant software (version 1.6.17.0). The standard settings were used with the following changes and additions: The modified FASTA database with individual substitutions of cysteines with the placeholder "U" was used. Labels were set on the placeholder amino acid "U" for the light isoDTB tag as light label ($C_{28}H_{46}N_{10}O_6S_1Se_{-1}$) and the heavy isoDTB tag as heavy label ($C_{24}{}^{13}C_4H_{46}N_8{}^{15}N_2O_6S_1Se_{-1}$). The multiplicity was set to 2 and the maximum number of labelled amino acids to 1. The proteolytic enzyme was set to Trypsin/P with a maximum number of misses cleavages of 2. No variable modifications were included and carbamidomethylation (cysteine) was used as fixed modification. The "Re-quantify" option was enabled. Contaminants were included. Peptides were searched with a minimum peptide length of 7 and a maximum peptide mass of 4,600 Da. The "Second peptides" as well as the "Dependent peptides" option was disabled. "Match between runs" was active with a Match time window of 0.7 min and an alignment window of 20 min. An FDR of 0.01 was used for Protein FDR, PSM FDR and XPSM FDR.

As previously described[12], for further analysis, the "peptides.txt" file of the MaxQuant analysis was used. All peptide sequences without a modified cysteine (placeholder "U") and all reverse sequences were deleted. Only the columns "Sequence", "Leading Razor Protein", "Start Position" and the columns for "Ratio H/L" for all replicates were kept. The "Leading Razor Protein" was renamed to the UniProt Code without the indicator for the number of the cysteine. All individual ratios were filtered out, if they were "NaN", and all other values were transformed

into log2-scale. For each peptide, the data was filtered out, if it was not present in at least two replicates or if the standard deviation between the replicates exceeded a value of 1.41. For each peptide, an identifier was generated in the form "UniProt Code""C""residue number of the modified cysteine". The data for the same replicate for all peptides with the same identifier, and therefore the same modified cysteine, were combined. Here, the median of the data was used. The data was filtered out if the standard deviation exceeded a value of 1.41. Each modified cysteine was kept in the dataset once with the shortest peptide sequence as the reported sequence. For each modified cysteine, the values of the replicates were combined, but the individual values were also reported. The values were combined as the median and the data was filtered out if there was data not in at least two of the replicates or if the standard deviation exceeded a value of 1.41. These are the final ratios that are reported. The information on the "Gene Name" and "Name" was linked back from the FASTA database.

Downstream analysis was performed with the Perseus[9] software according to a published procedure.[12] All individual values for each modified cysteine for the same condition were loaded into Perseus and analyzed using a one-sample t-test against a value of $\log_2(R) = 0$. Conditions with $p < 0.05$ were considered significant; for the identification as "hit", the requirements were: the statistical significance $p < 0.05$ and the median ratio $\log_2(R) > 1$. For volcano plots, the median values for $\log_2(R)$ and the -log10(p) values derived from Perseus are used. For the final data analysis, all cysteines that were quantified in less than two out of four replicates were excluded.

To determine the protein essentiality, the data for the database of essential genes (DEG)[15] was used as previously described.[12] It was obtained from aureowiki (aureowiki.med.uni-grweifswald.de). The information was linked to the information in the FASTA database through the "ordered locus name" that is reported in both aureowiki and Uniprot.[16]

For the functional site analysis, for all entries in the FASTA database of the analyzed strain the information in the categories "Active site", "Binding Site", "DNA-binding", "Nucleotide-binding", "Site" and "Metal-binding" were downloaded from uniprot.org.[16] With the information of all amino acid residues that are in the respective functional site, it was determined for all cysteines, whether they are in these functional sites or less than six amino acids in the primary sequence in distance from a residue in a respective site. In such case, the cysteine is considered as being at a functional site.

As previously reported[12], Gene Ontology (GO)[17] terms for all entries in the FASTA database for *S. aureus* NCTC 8325 in the category "GO – Molecular function" were downloaded from uniprot.org.[16] For each term that was present in the database at least once, a functional class was annotated manually. Thus, each term for each protein was assigned to a functional class. If a protein was only associated with terms from one functional class, it was attributed to it. If a protein was linked to terms from different functional classes, the functional class of the protein was determined from the following priority order: enzyme, modulator / scaffolding / adaptor, receptor / transporter / channel and then gene expression / nucleic acid-binding. The protein was classified as "not assigned", if no functional class could be assigned to any of the terms.

Thermal proteome profiling (TPP)

To determine protein-compound interactions, a thermal proteome profiling experiment was conducted.[18] Two *S. aureus* NCTC 8325 precultures were diluted 1:100 into fresh B medium and grown to the early stationary phase. Cells were harvested (8 mL, 6,000 x g, 4 °C, 5 min) and then washed with PBS (10 mL, 6,000 x g, 4 °C, 10 min). Cells were resolved in PBS and adjusted to $OD_{600} = 15$. From each biological replicate, one 1.4 mL aliquot was treated with 14 µL 1 mM **I16** in DMSO (10 µM final concentration) and the second aliquot with 14 µL DMSO and incubated for 1 h (37 °C, 200 rpm). From this point, samples were kept on ice if not

otherwise described. Each sample was split into 10 x 100 µL and transferred to PCR tubes for a 3 min temperature treatment in a PCR-Cycler. The 10 treatment temperatures were: 42.3 °C, 45.9 °C, 49.2 °C, 52.8 °C, 56.4 °C, 59.8 °C, 62.6 °C, 67.1 °C, 71.1 °C, 77.7 °C. The heat treatment was followed by 3 min at room temperature. For cell lysis, 100 µL lysis buffer (50 µg/mL lysostaphin, 30 µg/mL DNAse I, 1.6% NP40) were added and samples were incubated for 30 min (37 °C, 200 rpm) and subsequently snap-frozen in liquid nitrogen and stored at -80 °C. A freeze-thaw cycle (3 x -196 °C, 3 x 20 °C) completed the lysis procedure. Aggregated proteins were pelletized (6,000 x g, 4 °C, 10 min) and 160 µL of the supernatant were filtered through a MultiScreen$_{HTS}$-HV 0.45 µm 96-well filter plate (6,000 x g, 4 °C, 10 min; MSHVN4550, *Millipore*). The protein concentration was determined for the two lowest temperature points (ROTI Quant universal, *Roth*) and adjusted to 50 µg in 70 µL for each sample in a 96-well LoBind PCR-plate (*Eppendorf*). To reduce and alkylate cysteines, 3 µL of a 1:2 mix of 500 mM TCEP and 500 mM IAA were added and the plate was incubated in a shaker (10 min, 25 °C, 950 rpm). The reaction was quenched by addition of 2 µL 500 mM DTT (5 min, 25 °C, 950 rpm). Meanwhile, 2 mL of a 1:1 mixture of hydrophobic and hydrophilic carboxylated-magnetic beads (65152105050250, 45152105050250, *Cytiva*) were prepared. 10 µL bead mixture per sample were transferred into a fresh tube and placed inside a magnetic rack. After bead assembly, the supernatant was removed and it was washed with 3 x 1 mL ddH_2O. Subsequently, the beads were resuspended in the original volume in ddH_2O.10 µL beads were added to each sample and proteins were precipitated by addition of 120 µL MS-grade ethanol (5 min, 25 °C, 950 rpm). By using a magnetic rack, the supernatant was removed. The samples were further washed with 80% ethanol (3 x 180 µL, 5 min, 25 °C, 950 rpm) and acetonitrile (1 x 180 µL, 5 min, 25 °C, 950 rpm). After careful removal of the washing solution, the beads were resuspended in 50 µL 50 mM TEAB containing 0.01 µg/µL trypsin (*Promega*). The plate was sealed with an adhesive film, and proteins were digested overnight at 37 °C at 950 rpm. The following day, the supernatant from each sample was transferred to fresh centrifuge tubes and the remaining beads were washed with 80 µL 50 mM TEAB. The combined TEAB peptide solutions were dried using a centrifugal vacuum concentrator (*Eppendorf*) and stored at -80°C. The peptides were reconstituted in 7.5 µL labelling buffer (50 mM HEPES, 20% acetonitrile, pH 8.0). Tandem Mass Tag (TMT) isobaric labels (TMT10plexTM isobaric Labels Reagent Set 1x 0.8 mg, *Thermo Fisher Scientific*) were solubilized in 90 µL dry acetonitrile. 5 µL of each label solution were added to a sample according to applied temperature (Table S9) and incubated under gentle mixing (1 h, 25 °C, 400 rpm). The labeling reaction was stopped by adding 5% hydroxylamine and incubation (20 min, 25 °C, 400 rpm). 188.5 µL 0.1% FA were added to each sample. Labeled peptides were combined corresponding to their initial experimental condition and dried using a centrifugal vacuum concentrator. Dried peptides were stored at -80 °C. The samples were resolved in 500 µL 0.5% FA and loaded onto equilibrated (0.1% TFA) SepPak C18 columns (50 mg, *Waters*). The trapped peptides were desalted (3 x 1 mL 0.1% TFA, 1 x 0.5 mL 0.5% FA) and then eluted (3 x 0.25 mL 80% acetonitrile, 0.5% FA) into fresh centrifuge tubes. The eluates were dried in a centrifugal vacuum concentrator. For hydrophilic interaction liquid chromatography (HILIC) purification, samples were reconstituted in HILIC buffer A (110 µl; 95% acetonitrile 5% water 0.1% TFA), vortexed and centrifuged (21,000 x g, 4 °C,10 min). Peptide fractionation was carried out using an UltiMate 300 HPLC system (*Dionex*) equipped with an YMC-Pack PVA-Sil column (5 µm, 150 x 2.1 mm, 120 Å, *YMC Europe*). Gradient elution was carried out with HILIC buffer A (95% acetonitrile 5% water 0.1% TFA) and B (95% water 5% acetonitrile 0.1% TFA.). 100 µL sample were injected and separated using a 62.5 min gradient (7.5 min 0% B, 50 min to 30 % B, 3.5 min to 50 % B, 2.5 min to 100 % B) at a flow rate of 0.2 mL/min, followed by a washing and equilibration step. During separation, an on-line UV detector set at 215 nm was used to monitor peptide elution. Fractions were collected into a 96-well plate and pooled into 5 greater fractions. Fractions were dried in a centrifugal vacuum concentrator, resolved in 100 µL 1% FA, filtered through 0.22 µM Ultrafree-MC centrifugal filters (3,000 x g, 3 min; UFC30GVNB, *Millipore*), which were equilibrated with 300 µL 1% FA. Filtrates were transferred to MS vials and stored at -20 °C until LC-MS/MS measurement. The experiment was carried out in duplicates.

Table S11: Overview of reporter ion isotopic distributions as stated in product data sheet for TMT10plexTM Label reagent set (LOT number: WL320956) and fraction associated temperature (T) point.

Mass Tag	Reporter Ion	-2 (%)	-1 (%)	Monoisotop. (%)	+1 (%)	+2 (%)	T point (°C)
TMT^{10}-126	126.127726	0.0	0.0	100	7.4 (127C)	0.0 (128C)	42.3
TMT^{10}-127N	127.124761	0.0	0.1	100	7.8 (128N)	0.1 (129N)	45.9
TMT^{10}-127C	127.131081	0.0	0.2 (126)	100	6.6 (128C)	0.0 (129C)	49.2
TMT^{10}-128N	128.128116	0.0	1.2 (127N)	100	6.3 (129N)	0.0 (130N)	52.8
TMT^{10}-128C	128.134436	0.0 (126)	1.3 (127C)	100	5.7 (129C)	0.1 (130C)	56.4
TMT^{10}-129N	129.131471	0.0 (127N)	1.5 (128N)	100	5.7 (130N)	0.1 (131)	59.8
TMT^{10}-129C	129.137790	0.3 (127C)	2.7 (128C)	100	4.8 (130C)	0.0	62.6
TMT^{10}-130N	130.134825	0.0 (128N)	2.2 (129N)	100	4.6 (131)	0.0	67.1
TMT^{10}-130C	130.141145	0.0 (128C)	3.1 (129C)	100	3.6	0.0	71.1
TMT^{10}-131	131.138180	0.0 (129N)	8.7 (130N)	100	3.4	0.0	77.7

MS-measurements were performed on an Orbitrap Fusion instrument (*Thermo Fisher Scientific*) coupled to an UltiMate 300 nano HPLC system (*Thermo Fisher Scientific*), using an Acclaim C18 PepMap100 75 µm ID x 2 cm trap column (*Thermo Fisher Scientific*) and an Aurora series AUR2-25075C18A separation column (75 µm ID x 25 cm, *IonOpticks*) under constant heating to 40 °C. Samples (2 µL) were first loaded on the trap column with 0.1% TFA (7 min, 5 µL/min flow rate) and were then transferred to the separation column. The separation method was run using buffer A (0.1% FA in water) and buffer B (0.1% FA in acetonitrile) at a flow rate of 400 nL/min. The 152 min gradient consisted of the following steps: 7 min B at 5%, over 105 min gradient increase of B to 22%, over 10 min to 32%, over 10 min to 90%, wash step for 10 min at 90%, decrease to 5% in 0.1 min and equilibration at 5% for 9.9 min. The peptides were ionized at 1.8 kV and transferred with a capillary temperature of 275 °C. MS data on the Orbitrap Fusion instrument were acquired in data-dependent mode with a full cycle time of 3 s. Fluoranthene cations (Easy-IC) were used for internal calibration. MS full scans (scan range 375-1,500 m/z) were performed in the orbitrap at a resolution of R = 120,000 with an AGC ion target of 2×10^5, maximum injection time of 50 ms and RF Lens amplitude set to 60%. Monoisotopic precursor selection as well as the dynamic exclusion (mass tolerance of 10 ppm low/high) for 90 s was enabled. Precursors with charge states 2-7 and intensities greater than 5×10^3 were selected for fragmentation by collision-induced dissociation (CID) at 35% collision energy (fixed energy mode). The isolation was performed with the quadrupole with a window of 1.0 m/z. MS2 scans were recorded in the ion trap using the rapid scan rate. The AGC target was set to 1×10^4 with a maximum injection time of 100 ms. For MS3 scans, synchronous precursor selection (SPS) with 10 precursors was activated. The MS isolation window was set to 2.5 m/z and the MS2 isolation window to 3 m/z. Fragmentation was performed in the HCD cell at 55% collision energy. MS3 scans were recorded in the orbitrap at a resolution of R = 60,000 with an AGC ion target of 5×10^4 with a maximum injection time of 118 ms.

MaxQuant (version 2.2.0.0) was used to analyze the raw files with the Andromeda search engine using the following parameters for TMT quantification: experiments and fractions were set corresponding to the experimental protocol; type: Reporter Ions MS3, 10plex TMT (N-terminal and lysine; indication of correction factors of TMT Label batch); fixed modifications: carbamidomethylation (cysteine); variable modifications: oxidation (methionine), acetylation (N-terminus); proteolytic enzyme: trypsin/P; max. missed cleavages: 2. Other default settings were not changed. Searches were performed with the UniProt database for *S. aureus* NCTC 8325 (taxon identifier: 93061; downloaded on 21.06.2022).

For downstream analysis, the corrected reporter intensities (obtained from the protein groups table from the MaxQuant analysis) were used for the determination of melting curves and T_m shifts. The results were obtained by using R (version 4.1.1 "Kick things") and the TPP R package (version 3.20.1) obtained from Bioconductor.[18, 19] Intensities of channel TMT1-126C were excluded from the MaxQuant outputfile after identification of DMSO1_TMT1 as an outlier. Corrected intensities were normalized to the lowest applied temperature TMT channel. Data analysis was carried out as stated by the authors with minor changes (fcColumn = c(6,8,9)).

Data were filtered using the following criteria: $R^2 > 0.7$ for fitted curves (vehicle and treatment); plateau of < 0.3 for vehicle curves; minimum slope control vs. vehicle < -0.05; melting point difference for vehicles < 5.0.

To be considered as a hit, the following additional criteria were applied: melting point shifts for both paired replicates have the same direction; melting point difference treatment vs. vehicle > vehicle vs. vehicle; melting point difference for vehicles < 2.0.; minimum slope control vs. vehicle < -0.6; adjusted p values for both replicates are < 0.1.

Data visualization was carried out using Graphpad Prism 10.

MS-based analysis of I16 cysteine reactivity

An MS-based assay was used to evaluate the cysteine reactivity of **I16**. **I16** (2 µL, 20 mM stock in acetonitrile, 400 µM final concentration) was incubated with *N*-acetyl-L-cysteine methyl ester (2 µL, 200 mM stock in acetonitrile, 4 mM final concentration) in 100 µL of a 1:1 mixture of acetonitrile and water for 1 h (37 °C, 200 rpm). As controls, the isocyanide and the cysteine derivative were incubated alone. 5 µL of the samples were analyzed using a LTQ-FT Ultra (*Thermo Fisher Scientific*) coupled to an UltiMate 3000 HPLC system (*Thermo Fisher Scientific*) operated in ESI mode in a scan range of m/z = 100-1000. The separation was carried out on a Waters XBridge C18 3.5 µM column (4.6 x 100 mm) with a flow of 1.10 mL/min. The buffers consisted of 0.1% FA in water (A) and 0.1% FA in 10% water in acetonitrile (B). The separation gradient consisted of a washing step at 20% B for 0.5 min, followed by an increase to 98% over 4.5 min and a 2 min plateau at 98%. Within 0.5 min it is decreased again to 20% B and equilibrated for 2 min. The obtained UV- and HRMS-spectra were compared for the identification of adducts. The experiment was carried out in quadruplicates.

Cloning

The recombinant proteins Glutamine-fructose-6-phosphate aminotransferase (gene: GlmS, UniProt ID: Q2FWA0) and 3-oxoacyl-[acyl-carrier-protein] synthase 2 (gene: FabF; UniProt ID: Q2FZR9) from S. aureus NCTC 8325 wild type (Taxon ID: 93061) were expressed by using the *Invitrogen* Gateway Technology according to the manufacturer's protocols. Genomic DNA was isolated by using the peqGOLD Bacterial DNA Kit (*VWR*). The target genes were amplified by PCR with the Phusion High-Fidelity DNA Polymerase (*New England Biolabs*) according to the manufacturer's instructions. The used primers are listed in the table below (Table S10). PCR products were extracted with an E.Z.N.A. Gel Extraction Kit (*Omega Bio-Tek*). The purified attB-PCR products were integrated into the donor vector pDONR207 (*Invitrogen*) through recombination using the BP Clonase II Enzyme Mix (*Invitrogen*). After transformation into competent Top10 *E. coli* (Invitrogen), clones of transformed cells were selected and plasmid DNA was obtained by using the peqGOLD Plasmid Miniprep Kit I (*VWR*). The purified plasmids were cloned into the destination vectors pET301/CT-Dest (used for GlmS, *Invitrogen*) and pET300/NT-Dest (used for FabF, *Invitrogen*) by the LR Clonase II Enzyme Mix (*Invitrogen*). The expression clones were transformed in chemically competent *E. coli* BL21 (DE3). Clones were selected and the sequence verified via Sanger sequencing (*Genewiz*). The sequences of the expressed constructs are shown in the appendix.

Table S12. AttB1- and attB2-sequences, respectively, are in lower case, the Shine-Delgarno-sequence is shown in bold, and the TEV cleavage site sequence is underlined. The consensus sequence with the genetic code of GlmS or FabF are shown in capital letters.

Primer	Sequence
GlmS for.	ggggacaagtttgtacaaaaaagcaggcttt**gaaggagatagaacc**ATGTGTGGAATTGTTGGTTATATTGGC
GlmS rev.	ggggaccactttgtacaagaaagctgggtggccctgaaaataaagattctcTTCCACAGTAACTGATTTAGCAAG
FabF for.	ggggacaagtttgtacaaaaaagcaggctttgagaatctttattttcagggcATGAGTCAAAATAAAAGAGTAGTTATTACAGGTATG
FabF rev.	ggggaccactttgtacaagaaagctgggtggTTATGCTTCAAATTTCTTGAATACTAATAC

pET-28a(+) plasmids encoding the point mutants GlmS_C2A and FabF_C165A were purchased from *TwistBioscience.* Codon optimization for *E. coli* expression was performed. The sequences of the purchased plasmids are shown in the appendix. The sequences were verified by DNA sequencing (*Genewiz*). The plasmids were transformed into chemically competent *E. coli* Bl21 (DE3) cells for protein expression and purification.

Protein overexpression and purification

The protein overexpression and purification methods were adapted from published procedures for the C-terminally-His-tagged-GlmS and its point mutant as well as the N-terminally-His-tagged-FabF and its mutant.[20-22] 2 L of LB containing 100 µg/mL ampicillin (for GlmS and FabF) or 25 µg/mL kanamycin (for the respective point mutants) were inoculated (1:100) with overnight cultures of the corresponding expression strains. Cultures were grown to an OD_{600} of 0.5-0.7 (1.5-2.5 h, 37 °C, 200 rpm), followed by inducing protein overexpression with 1 mM isopropyl-1-thio-β-D-galactopyranoside (IPTG). Cells were incubated at 18 °C and 200 rpm for 20 h. Bacteria were harvested (6,000 x g, 10 min, 4 °C) and washed with 50 mL PBS (6,000 x g, 10 min, 4 °C). The washed pellets were resuspended in 30 mL of the corresponding lysis and wash buffer (GlmS: 50 mM Tris/HCl, pH 7.5, 200 mM NaCl, 10 mM imidazole, 1 mM TCEP, 0.5 mM Na_2Frc_6P, 10% glycerol, supplemented with EDTA-free protease inhibitor cocktail (*Roche*); FabF: 50 mM KPO_4, pH 7.8, 300 mM NaCl, 10 mM imidazole, 10% glycerol) and lysed by sonication on ice (7 min at 30% intensity, 3 min at 50% intensity, 7 min at 30% intensity; Sonopuls HD 2070 ultrasonic rod Sonicator, *Bandelin electronic*). Affinity purification was performed on an ÄKTA pure 25 FPLC protein purification system (*GE Healthcare*) coupled to a fraction collector (F9-C, *GE Healthcare*) at 4 °C. The cleared lysates (18,000 rpm, 4 °C, 1 h) were filtered (0.45 µm PVDF filter, Whatman GD/X25) and loaded onto an equilibrated 5 mL HisTrap HP column (*GE Healthcare*). After washing, the proteins were eluted by using a gradient elution method with the corresponding lysis/wash buffers and elution buffers (500 mM imidazole instead of 10 mM). Fractions containing protein were concentrated (centrifugal filters, 10 kDa cut-off, 3,200 x g, 4 °C), and for GlmS and GlmS_C2A, the C-terminal His_6-tag was proteolytically removed by using TEV protease. The proteins were further purified by size exclusion chromatography (SEC) using a 120 mL HiLoad 16/60 Superdex 200 prep grade column (*GE Healthcare*) with respective SEC buffers (GlmS: 50 mM Tris/HCl, pH 7.5, 1 mM TCEP, 0.5 mM Na_2Frc_6P, 10% glycerol; FabF: 25 mM Tris/HCl, pH 7.8, 300 mM NaCl, 0.2 mM TCEP, 10% glycerol). Proteins were concentrated (centrifugal filters, 10 kDa cut-off, 3,200 x g, 4 °C) and the final concentration measured by using a NanoDrop (*Thermo*). Protein purity was verified by SDS-PAGE and by intact protein mass spectrometry measurements. Protein aliquots were stored at -80 °C

SDS-PAGE

Loading gels consisted of 4% (w/v) acrylamide (in 50 mM Tris, pH 6.8) and resolving gels of 12.5% (w/v) acrylamide (in 300 mM Tris, pH 8.8). The running buffer was a Tris-glycine buffer (25 mM Tris, 192 mM glycine, 0.1% (w/v) SDS, pH 8.3). The protein marker Peqlab peqGold (*VWR*) and fluorescent marker BenchMark Fluorescent Protein Standard (*Thermo Fisher Scientific*) served as protein standards. Gels were run at 150 V on a EV265 Consort power supply (*Hoefer*). Fluorescence bands were visualized on a LAS-4000 (Fujifilm) using 520 nm EPI excitation wavelength. Gels were stained in Coomassie staining solution (0.25% (w/v) Coomassie Brilliant Blue R-250, 9.2% (v/v) concentrated acetic acid, 45.4% (v/v) ethanol) and destained in 10% (v/v) acetic acid, 40% (v/v) ethanol).

Intact protein MS (IPMS) experiments

For the validation of the identity of expressed and purified proteins and for the determination of covalent I16 modifications on the proteins high-resolution intact protein mass spectra (IPMS) were measured.

For modification analysis of FabF, 10 µL of a 10 µM protein solution in PBS (pH 7.4) and 5 µL **I16** (400 µM in DMSO) or DMSO were added into 85 µL PBS (pH 7.5) and incubated for 1 h at 37 °C and 200 rpm. For modification analysis of GlmS, 10 µL of a 10 µM protein solution in PBS (pH 7.4) and 10 µL **I16** (400 µM in DMSO) or DMSO were added into 80 µL PBS (pH 7.5) and incubated for 1 h at 37 °C and 200 rpm.

FabF samples were measured on a LTQ-FT Ultra (FT-ICR-MS, *Thermo Fisher Scientific*) mass spectrometer with an electrospray ionization source (spray voltage 4.0 kV, tube lens 110 V, capillary voltage 48 V, sheath gas 60 a. u., aux gas 10 a. u., sweep gas 0.2 a. u.) coupled with an UltiMate 3000 HPLC system (*Thermo Fisher Scientific*). The samples were desalted with a MassPREP On-Line Desalting Cartridge (*Waters*). The mass spectrometer was operated in positive ion mode collecting full scans at high resolution (R = 200,000) in a range of m/z = 600-2000. The protein spectra were deconvoluted using the UniDec algorithm.

GlmS samples were measured on a Synapt XS ESI-TOF mass spectrometer (*Waters*) equipped with an ACQUITY HPLC system (*Waters*). Samples were run on a C4 column and a 0–95% acetonitrile gradient was applied. The mass spectrometer was operated in positive ion mode collecting full scans in a range of m/z = 400-4000. Further instrument settings: cone gas: 50 L/h; desolvation gas: 600 L/h; source temperature: 100 °C; desolvation temperature: 250 °C; nebulizer: 6 bar; cone voltage: 70 V. The raw data protein spectra were deconvoluted with the MassLynx software V.4.2 (*Waters*).

Gel-based labeling experiments

Purified protein (GlmS wildtype or C2A mutant; FabF wildtype or C165A mutant; 1-5 µM final concentration) were spiked into native S. aureus NCTC 8325 lysate (1 mg/mL) to give a final volume of 50 µL. As control, the mutant proteins in lysate or lysate without additional proteins was used. The lysates were treated with **I16** (1 µL, 50x stock in DMSO) or with DMSO (1 µL) as control and incubated for 1 h at 23 °C and 400 rpm. IA-Alkyne (1 µL of 0.5-5 mM stock in DMSO) was added and the samples were incubated for 1h at 23 °C and 400 rpm. A click reaction was performed with TAMRA-azide (*Jena bioscience*) by adding 6 µL of a solution containing 3 µL 0.9 mg/mL TBTA ligand in 4:1 tBuOH/DMSO, 1 µL 50 mM $CuSO_4$ in water, 1 µL 13 mg/mL TCEP in water and 1 µL of 5 mM Rhodamine-azide in DMSO. The samples were incubated for another hour at room temperature, without shaking in the dark. The reactions were quenched by adding 50 µL of 2x Laemmli buffer (125 mM Tris–HCl, 20% (v/v) glycerol, 4% (w/v) SDS, 0.005% (w/v) bromphenol blue, 10% (v/v) 2-mercaptoethanol) and analyzed by SDS-PAGE. The protein marker Peqlab peqGold (*VWR*) and fluorescent marker BenchMark

Fluorescent Protein Standard (*Thermo Fisher Scientific*) served as protein standards. Fluorescence bands were visualized on a LAS-4000 (Fujifilm) using 520 nm EPI excitation wavelength. Protein loading was visualized by Coomassie staining.

GDH-coupled GlmS assay

The glutaminase activity of GlmS was measured in a coupled enzymatic assay using bovine glutamate dehydrogenase (GDH, glycerol solution (50%), *Merck*). The protocol was adapted from a previously described assay.[20, 21] All solutions were prewarmed to 37 °C before the assay was started. In a transparent 96-well plate, GlmS or the C2A mutant (20 µg/mL final concentration) was preincubated for 30 min at 37 °C with **I16** (in DMSO, 40/100/200 µM final concentration), iodoacetamide (IAA, in DMSO, 100 µM final concentration) or DMSO (3%) in presence of Frc_6P (10 mM final concentration) in the assay buffer (100 mM KPO_4, pH 7.5, 50 mM KCl, 1 mM EDTA). As background control, the mixture without protein was used. The assay was started by adding a freshly prepared mixture containing 3.36 U GDH, glutamine (in assay buffer, 10 mM final concentration), and APAD (in assay buffer, 1 mM final concentration). The total volume was 100 µL. After GDH addition, the activity was monitored continuously at 363 nm for 3 h, following the APADH production, with an Infinite M200 Pro microplate reader (*Tecan Group*). To determine the glutaminase activity of GlmS, the increase of absorption in the linear range was calculated (after deduction of the background) and normalized to the DMSO-treated control samples. The assay was conducted in n = 5 (wt inhibition) or n = 3 (wt vs. mutant) independent experiments with three technical replicates each. Of note, it is assumed that the recombinant GlmS in this study is not as active as the wild-type protein due to an overhang of the TEV-cleavage sequence at the C-terminus.

A control assay was performed to ensure that cysteine reactive compounds do not impact GDH activity. In a transparent 96-well plate, GDH (3.36 U) was incubated for 3 min at 37 °C with I16 (in DMSO, 200 µM final concentration), IAA (in DMSO, 100 µM final concentration) or DMSO (3%) in presence of APAD (1 mM final concentration) in the assay buffer (100 mM KPO_4, pH 7.5, 50 mM KCl, 1 mM EDTA). After adding glutamic acid (200 µM final concentration), the activity was monitored continuously at 363 nm for 8 min, following the APADH production, with an Infinite M200 Pro microplate reader (*Tecan Group*). The total volume was 100 µL. To determine the dehydrogenase activity of GDH, the increase of absorption in the linear range was calculated (after deduction of the background) and normalized to the DMSO-treated control samples. The assay was conducted in five independent experiments with three technical replicates each.

Chemical Synthesis

General information

Commercially available starting materials, reagents, screening compounds and anhydrous solvents were obtained from *Sigma Aldrich, Merck, Thermo Fisher Scientific, TCI, Arcos, VWR, Roth, Enamine,* and *Alfa Aesar*, and used without further purification. All air and moisture sensitive reactions were carried out under argon atmosphere (Ar4.6) in flame-dried reaction flasks. Anhydrous solvents and water-sensitive liquids were transferred using argon flushed syringes. Chromatography was performed using silica gel [40-63 µm (Si 60)] from *Merck.* The mixture of solvents is given as the ratio of volume. Technical solvents used for workup steps and for column chromatography were used without further purification. The following abbreviations are used for solvents: P (pentane), DCM (dichlormethane), EtOAc (ethyl acetate), Et_2O (diethyl ether), MTBE (methyl *tert*-butyl ether).

Analytical Methods

Analytical Thin Layer Chromatography (TLC)

Qualitative TLC was performed on silica gel plates (aluminium) from *Merck* (0.25 mm silica 60, F254). For visualization, TLC plates were observed under UV-light (λ = 254 and 366 nm) and/or stained with $KMnO_4$ (3.00 g $KMnO_4$, 20.0 g K_2CO_3 and 5.00 mL 5% NaOH in 300 mL water) with subsequent heat treatment (ca. 250 °C)

NMR-Spectroscopy

^1H and ^{13}C NMR spectra were recorded at 298 K either on *Bruker* AVHD-300, AVHD-400, AVHD-500 instruments or on an AV-II-500 equipped with a cryo probe head. Chemical shifts (δ) are reported in parts per million (ppm). Spectra were referenced to residual proton and carbon signals of the deuterated solvent d6-DMSO: δ (1H) = 2.50 ppm, δ (13C) = 39.52 ppm. The following abbreviations are used to describe NMR coupling patterns: s (singlet), d (doublet), t (triplet), q (quartet), m (multiplet). The coupling constants, J, are reported in Hertz (Hz)

Mass-Spectrometry

High-resolution mass spectrometry (HR-MS) was performed on a LTQ-FT Ultra, a LTQ Orbitrap XL, or a Q Exactive Plus from *Thermo Fisher Scientific* (ESI). EI results were obtained from a *Thermo Fisher Scientific* DFS-HRMS spectrometer.

Elemental Analysis

Elemental analysis (EA) was performed by an in-house service on a *HEKATech* EURO-EA with HT pyrolysis.

Synthetic procedures and analytical data

General synthesis scheme

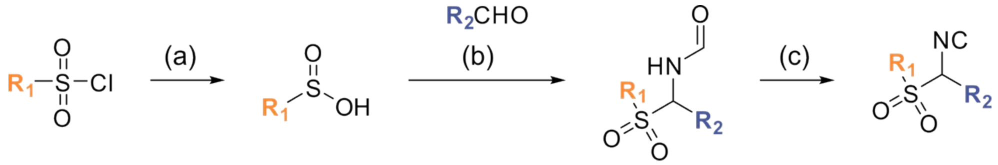

General synthesis scheme for **I16** derivatives. Reagents and conditions: (a) Na_2SO_3, $NaHCO_3$, water, 80 °C; HCl, water, rt; (b) formamide, TMSCl, MeCN, toluene, 50 °C; (c) $POCl_3$, NEt_3, -40/0 °C.

Sulfinic acids

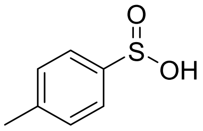

4-methylbenzenesulfinic acid

p-Toluenesulfinic acid sodium salt (2.0 g, 11.22 mmol, 1 eq.) was dissolved in 15 mL water. 13 mL MTBE were added and HCl (1 mL, 11.22 mmol, 1 eq.) was added dropwise over 5 minutes. The solution was stirred for 20 minutes. Subsequently, the phases were separated and 15 mL toluene were added to the organic phase and evaporated until a white solid was obtained. The solid was collected by filtration and rinsed with hexane. The product was dried under vacuum and used without further purification. The free sulfinic acid was obtained as a white powder (1.213 g, 7.777 mol, 69.3%).

TLC (EtOAc): R_f = 0.16 [UV, $KMnO_4$]

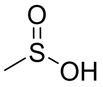

Methanesulfinic acid

Methanesulfinic acid sodium salt (500 mg, 4.9 mmol, 1 eq.) was dissolved in 30 mL acetone and stirred for 20 min. 0.4 mL 37% HCl (4.9 mmol, 1 eq.) were added dropwise and the mixture stirred for additional 20 min. A white solid precipitate was filtered from the solution and dried under vacuum for 1h. The free sulfinic acid was obtained as a white powder (355 mg, 4.43 mmol, 90.5%) and used without further purification.

TLC (EtOAc:Hx = 3:2): Rf = 0.12 [UV, $KMnO_4$]

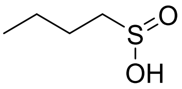

Butane-1-sulfinic acid

As previously described[23], butane-1-sulfonyl chloride (783 mg, 5 mmol, 1 eq.) was added to a solution of sodium sulfite (1.26 g, 10 mmol, 2 eq.) and sodium hydrogencarbonate (840 mg, 10 mmol, 2 eq.) in 15 mL water. The reaction mixture was stirred at 80 °C overnight. The aqueous layer was extracted thrice with DCM and then acidified with HCl. Then, the product was extracted from the aqueous phase with MTBE (3x) and concentrated in vacuo. The free sulfinic acid was obtained as a clear oil (416 mg, 3.41 mmol, 68.1%).

TLC (EtOAc:Hx = 3:2): R_f =0.26 [UV, $KMnO_4$]

^{1}H NMR (400 MHz, DMSO) δ 2.62 – 2.54 (m, 2H), 1.59 – 1.47 (m, 2H), 1.44 – 1.29 (m, 2H), 0.88 (t, *J* = 7.3 Hz, 3H).

^{13}C NMR (101 MHz, DMSO) δ 57.5, 23.3, 21.4, 13.7.

HRMS (ESI) m/z for $[M-H]^-$ calcd: 121.0329, found: 121.0321.

These data are in accordance with previous literature reports.[23]

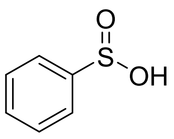

Benzenesulfinic acid

Benzenesulfinic acid sodium salt (2.02 g, 12.31 mmol, 1 eq.) was dissolved in 15 mL water. 13 mL MTBE were added and HCl (1.1 mL, 13.28 mmol, 1.08 eq.) was added dropwise over 5 minutes. The solution was stirred for 15 minutes. Subsequently, the phases were separated, and the aqueous phase extracted thrice with MTBE. The organic solvent was removed in vacuo. The free sulfinic acid was obtained as a white powder (1.726 g, 12.14 mol, 98.6%) and used without further purification.

TLC (EtOAc): R_f = 0.25 [UV]

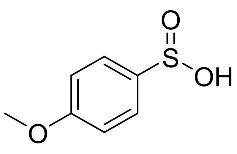

4-methoxybenzenesulfinic acid

As previously described[23], 4-methoxybenzenesulfonyl chloride (2.06 g, 9.97 mmol, 1 eq.) was added to a solution of sodium sulfite (3.70 g, 29.4 mmol, 3 eq.) and sodium hydrogencarbonate (2.44 g, 29.0 mmol, 3 eq.) in 15 mL water. The reaction mixture was stirred at 80 °C for two hours. The aqueous layer was extracted thrice with DCM and then acidified with HCl. Then, the product was extracted from the aqueous phase with MTBE (3x) and concentrated in vacuo. The sulfinic acid was obtained as white solid (904 mg, 5.52 mmol, 52.7%).

TLC (EtOAc): R_f =0.26 [UV]

^{1}H NMR (400 MHz, DMSO) δ 7.59 (d, J = 8.8 Hz, 2H), 7.09 (d, J = 8.8 Hz, 2H), 3.81 (s, 3H).

^{13}C NMR (101 MHz, DMSO) δ 161.6, 140.5, 126.3, 114.3, 55.5.

HRMS (ESI) m/z for $[M-H]^-$ calcd: 171.0121, found: 171.0112.

These data are in accordance with previous literature reports.[23]

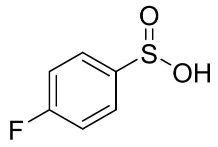

4-fluorobenzenesulfinic acid

As previously described[23], 4-fluorobenzenesulfonyl chloride (3.00 g, 15.4 mmol, 1 eq.) was added to a solution of sodium sulfite (3.99 g, 31.7 mmol, 2.1 eq.) and sodium hydrogencarbonate (2.61 g, 31.1 mmol, 2.0 eq.) in 15 mL water. The reaction mixture was stirred at 80 °C for four hours. The aqueous layer was extracted thrice with DCM and then acidified with HCl. Then, the product was extracted from the aqueous phase with MTBE (3x) and concentrated in vacuo. The sulfinic acid was obtained as pale yellow solid (2.24 g, 13.8 mmol, 89.6%).

TLC (EtOAc): R_f =0.30 [UV]

^{1}H NMR (400 MHz, DMSO) δ 7.78 – 7.68 (m, 2H), 7.44 – 7.34 (m, 2H).

^{13}C NMR (101 MHz, DMSO) δ 163.8 (d, J = 248.3 Hz), 145.1 (d, J = 2.9 Hz), 127.3 (d, J = 9.2 Hz), 116.1 (d, J = 22.4 Hz).

HRMS (ESI) m/z for $[M-H]^-$ calcd: 159.9922, found: 159.9911.

These data are in accordance with previous literature reports.[23]

Formamides

General procedure:

The procedure was adapted from published protocols.[24, 25] Aldehyde (2-10 mmol, 1 eq.) was dissolved in a 1:1 mixture of dry acetonitrile and dry toluene (4-12 mL). Formamide (2.5 eq.) and TMSCl (1.1 eq.) were added, and the mixture was stirred for 0-1 h at room temperature, before sulfinic acid (1.5 eq.) was added. The reaction was stirred at 50 °C overnight. Methyl tert-butyl ether (2-4 mL) was added to the cooled reaction mixture and stirred for 5 min. before adding water (8-16 mL). The mixture was put on ice. If a precipitate formed within 2 h, it was collected by filtration, washed with cold methyl *tert*-butyl ether and dried. If not, the aqueous phase was separated and extracted with ethyl acetate. The combined organic layers were washed with brine, dried over sodium sulfate and concentrated under reduced pressure. Compounds were either used without further purification, resuspended by sonication in methyl tert-butyl ether, filtered and dried, or purified by column chromatography**.**

For all formamides, only the main rotamer is reported.

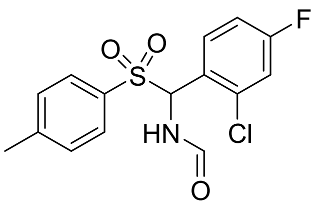

***N*-((2-chloro-4-fluorophenyl)(tosyl)methyl)formamide**

Obtained as a white solid (1.96 g, 5.73 mmol, 57.2%) after resuspension and sonication in methyl *tert*-butyl ether.

TLC (EtOAc:Hx = 1:1): R_f = 0.46 [UV]

^{1}H NMR (300 MHz, DMSO) δ 9.96 (dd, *J* = 10.3, 1.4 Hz, 1H), 8.02 (d, *J* = 1.2 Hz, 1H), 7.81 (dd, *J* = 8.8, 6.0 Hz, 1H), 7.72 – 7.62 (m, 2H), 7.55 (dd, *J* = 8.9, 2.7 Hz, 1H), 7.49 – 7.39 (m, 3H), 6.78 (d, *J* = 10.3 Hz, 1H), 2.41 (s, 3H).

^{13}C NMR (75 MHz, DMSO) δ 162.4 (d, *J* = 251.1 Hz), 160.5, 145.4, 135.4 (d, *J* = 10.9 Hz), 133.1, 132.0 (d, *J* = 9.5 Hz), 130.0, 129.0, 125.4 (d, *J* = 3.6 Hz), 116.9 (d, *J* = 25.6 Hz), 115.2 (d, *J* = 21.6 Hz), 65.8, 21.2.

HRMS (ESI) m/z for $[M+H]^+$ calcd: 342.0362, found: 342.0363.

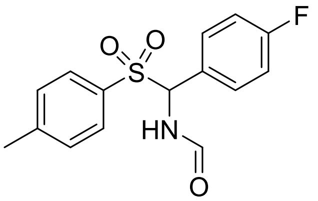

***N*-((4-fluorophenyl)(tosyl)methyl)formamide**

Obtained as an off-white solid (520 mg, 1.69 mmol, 53.7%) and used without further purification.

TLC (EtOAc:P = 3:2): R_f = 0.50 [UV]

^{1}H NMR (500 MHz, DMSO) δ 9.79 (dd, J = 10.6, 1.6 Hz, 1H), 7.96 (d, J = 1.3 Hz, 1H), 7.76 – 7.69 (m, 2H), 7.65 – 7.60 (m, 2H), 7.45 – 7.41 (m, 2H), 7.31 – 7.24 (m, 2H), 6.47 (d, J = 10.6 Hz, 1H), 2.40 (s, 3H).

^{13}C NMR (126 MHz, DMSO) δ 162.8 (d, J = 246.3 Hz), 160.3, 145.0, 133.3, 131.8 (d, J = 8.6 Hz), 129.7, 129.2, 126.7 (d, J = 3.0 Hz), 115.4 (d, J = 21.7 Hz), 69.4, 21.2.

HRMS (ESI) m/z for $[M+Na]^+$ calcd.: 330.0570, found: 330.0566.

These data are in accordance with previous literature reports. Literature spectra were recorded in deuterated chloroform.[26]

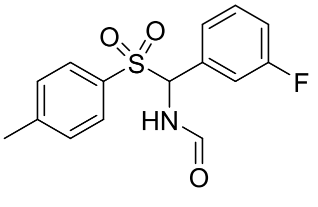

***N*-((3-fluorophenyl)(tosyl)methyl)formamide**

Obtained as slightly yellow solid (610 mg, 1.98 mmol, 63.0%) and used without further purification.

TLC (EtOAc:P = 3:2): R_f = 0.56 [UV]

^{1}H NMR (500 MHz, DMSO) δ 9.82 (dd, J = 10.6, 1.5 Hz, 1H), 7.98 (d, J = 1.4 Hz, 1H), 7.74 (d, J = 8.3 Hz, 2H), 7.53 – 7.35 (m, 5H), 7.33 – 7.25 (m, 1H), 6.52 (d, J = 10.6 Hz, 1H), 2.40 (s, 3H).

^{13}C NMR (126 MHz, DMSO) δ 161.8 (d, J = 244.0 Hz), 160.4, 145.1, 133.2, 133.0 (d, J = 7.9 Hz), 130.4 (d, J = 8.3 Hz), 129.7,129.3, 125.8 (d, J = 2.8 Hz), 116.5 (d, J = 7.2 Hz), 116.4 (d, J = 9.3 Hz), 69.6, 21.2.

HRMS (ESI) m/z for $[M+Na]^+$ calcd.: 330.0570, found: 330.0567.

These data are in accordance with previous literature reports.[27]

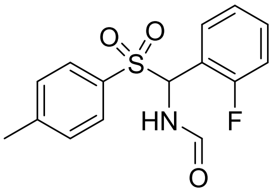

***N*-((2-fluorophenyl)(tosyl)methyl)formamide**

Obtained as a white solid (1.27 g, 4.13 mmol, 57.0%) and used without further purification.

TLC (EtOAc:P = 3:2): R_f = 0.54 [UV]

^{1}H NMR (500 MHz, DMSO) δ 9.91 (dd, J = 10.4, 1.4 Hz, 1H), 8.02 (d, J = 1.3 Hz, 1H), 7.69 – 7.64 (m, 3H), 7.60 – 7.47 (m, 1H), 7.48 – 7.42 (m, 2H), 7.34 (td, J = 7.6, 1.2 Hz, 1H), 7.27 (ddd, J = 9.6, 8.4, 1.2 Hz, 1H), 6.55 (d, J = 10.5 Hz, 1H), 2.41 (s, 3H).

^{13}C NMR (101 MHz, DMSO) δ 165.6, 160.6, 160.1 (d, J = 248.2 Hz), 133.0, 132.0 (d, J = 8.7 Hz), 130.2 (d, J = 2.4 Hz), 129.9, 129.0, 124.8 (d, J = 3.5 Hz), 117.9 (d, J = 14.0 Hz), 115.5 (d, J = 22.0 Hz), 63.6 (d, J = 3.4 Hz), 21.2.

HRMS (ESI) m/z for $[M+Na]^+$ calcd.: 330.0570, found: 330.0565.

These data are in accordance with previous literature reports. Literature spectra were recorded in deuterated chloroform.[28]

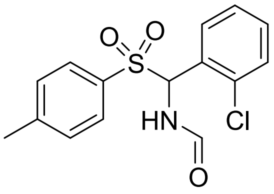

***N*-((2-chlorophenyl)(tosyl)methyl)formamide**

Obtained as a white solid (462 mg, 1.43 mmol, 66.7%) after purification by column chromatography (EtOAc:Hx = 3:2).

TLC (EtOAc:Hx = 3:2): R_f = 0.61 [UV]

^{1}H NMR (500 MHz, DMSO) δ 9.95 (dd, J = 10.4, 1.5 Hz, 1H), 8.01 (d, J = 1.3 Hz, 1H), 7.76 (dd, J = 7.4, 2.1 Hz, 1H), 7.67 (d, J = 8.3 Hz, 1H), 7.60 – 7.40 (m, 5H), 6.83 (d, J = 10.3 Hz, 1H), 2.41 (s, 3H).

These data are in accordance with previous literature reports. Literature spectra were recorded in deuterated chloroform.[28]

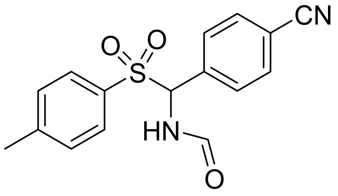

***N*-((4-cyanophenyl)(tosyl)methyl)formamide**

Obtained as a white solid (506 mg, 1.61 mmol, 70.3%) after purification by column chromatography (EtOAc:Hx = 3:2).

TLC (EtOAc:Hx = 3:2): R_f = 0.42 [UV]

^{1}H NMR (400 MHz, DMSO) δ 9.86 (dd, J = 10.6, 1.5 Hz, 1H), 7.98 (d, J = 1.3 Hz, 1H), 7.94 (d, J = 8.4 Hz, 2H), 7.79 (d, J = 8.4 Hz, 2H), 7.74 (d, J = 8.3 Hz, 2H), 7.45 (d, J = 8.0 Hz, 2H), 6.62 (d, J = 10.6 Hz, 1H), 2.41 (s, 3H).

^{13}C NMR (101 MHz, DMSO) δ 160.3, 145.2, 135.7, 133.0, 132.2, 130.4, 129.7, 129.2, 118.4, 112.2, 69.6, 21.2.

HRMS (ESI) m/z for $[M+Na]^+$ calcd.: 337.0617, found: 337.0612.

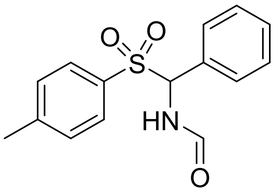

***N*-(phenyl(tosyl)methyl)formamide**

Obtained as a white solid (851 mg, 2.94 mmol, 68.9%) after purification by column chromatography (EtOAc:Hx = 3:1).

TLC (EtOAc:P = 3:2): R_f = 0.47 [UV]

^{1}H NMR (400 MHz, DMSO) δ 9.77 (dd, J = 10.6, 1.5 Hz, 1H), 7.96 (d, J = 1.4 Hz, 1H), 7.71 (d, J = 8.3 Hz, 2H), 7.62 – 7.50 (m, 2H), 7.50 – 7.32 (m, 5H), 6.38 (d, J = 10.6 Hz, 1H), 2.41 (s, 3H).

^{13}C NMR (101 MHz, DMSO) δ 160.2, 144.8, 133.4, 130.3, 129.6, 129.4, 129.1, 128.3, 70.2, 21.1.

HRMS (ESI) m/z for [M+Na]$^+$ calcd.: 312.0665, found: 312.0660.

These data are in accordance with previous literature reports. Literature spectra were recorded in deuterated chloroform.[28]

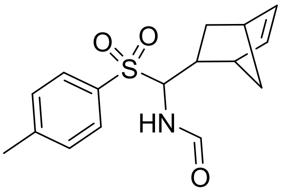

***N*-(bicyclo[2.2.1]hept-5-en-2-yl(tosyl)methyl)formamide**

Obtained as a slightly orange solid (463 mg, 1.52 mmol, 61.8%) after purification by column chromatography (EtOAc:Hx = 3:2).

TLC (EtOAc:Hx = 3:2): R_f = 0.43 [UV]

^{1}H NMR (400 MHz, DMSO) δ 9.17 – 8.95 (m, 1H), 7.89 – 7.81 (m, 1H), 7.74 – 7.61 (m, 2H), 7.50 – 7.37 (m, 2H), 6.26 – 5.58 (m, 2H), 4.62 – 4.38 (m, 1H), 3.24 – 2.65 (m, 3H), 2.39 (s, 3H), 1.96 – 1.73 (m, 1H), 1.45 – 0.42 (m, 3H).

^{13}C NMR (101 MHz, DMSO) δ 160.2, 159.8, 144.5, 144.5, 138.5, 138.0, 134.2, 134.1, 132.7, 131.3, 129.5, 129.5, 129.0, 71.9, 70.6, 49.1, 47.4, 45.0, 44.4, 42.3, 40.8, 37.6, 36.9, 30.6, 29.8, 21.1.

The spectral complexity results from stereoisomers of the bicyclic ring system.

HRMS (ESI) m/z for [M+Na]$^+$ calcd.: 328.0978, found: 328.0972.

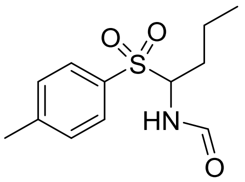

***N*-(1-tosylbutyl)formamide**

Obtained as a slightly orange solid (565 mg, 2.21 mmol, 53.1%) after purification by column chromatography (EtOAc:Hx = 3:2).

TLC (EtOAc:Hx = 3:2): R_f = 0.46 [UV]

^{1}H NMR (400 MHz, DMSO) δ 8.90 (d, J = 10.0 Hz, 1H), 7.94 – 7.89 (m, 1H), 7.74 – 7.65 (m, 2H), 7.47 – 7.39 (m, 2H), 5.11 (ddd, J = 11.2, 9.8, 3.2 Hz, 1H), 2.40 (s, 3H), 1.97 – 1.49 (m, 2H), 1.47 – 1.20 (m, 2H), 0.89 – 0.81 (m, 3H).

^{13}C NMR (101 MHz, DMSO) δ 160.8, 144.6, 133.5, 129.7, 129.0, 66.8, 27.8, 21.1, 18.0, 13.1.

HRMS (ESI) m/z for [M+Na]$^+$ calcd.: 278.0821, found: 278.0818.

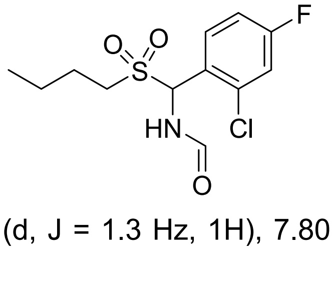

***N*-((butylsulfonyl)(2-chloro-4-fluorophenyl)methyl)formamide**

Obtained as a colourless oil (293 mg, 0.95 mmol, 50.3 %) after purification by column chromatography (EtOAc:Hx = 7:3).

TLC (EtOAc:Hx = 7:3): R_f =0.54 [UV]

^{1}H NMR (500 MHz, DMSO) δ 10.01 (dd, J = 10.1, 1.5 Hz, 1H), 8.24 (d, J = 1.3 Hz, 1H), 7.80 (dd, J = 8.8, 6.0 Hz, 1H), 7.60 (dd, J = 8.8, 2.7 Hz, 1H), 7.43 (td,

J = 8.5, 2.7 Hz, 1H), 6.76 (d, J = 10.2 Hz, 1H), 3.15 (t, J = 8.0 Hz, 2H), 1.81 – 1.63 (m, 2H), 1.47 – 1.34 (m, 2H), 0.89 (t, J = 7.4 Hz, 3H).

^{13}C NMR (126 MHz, DMSO) δ 162.5 (d, J = 250.6 Hz), 161.3, 135.3 (d, J = 10.9 Hz), 132.0 (d, J = 9.5 Hz), 124.9 (d, J = 3.4 Hz), 117.1 (d, J = 25.1 Hz), 115.2 (d, J = 21.6 Hz), 62.7, 49.5, 23.7, 21.0, 13.5.

HRMS (ESI) m/z for $[M+Na]^+$ calcd.: 330.0337, found: 330.0333.

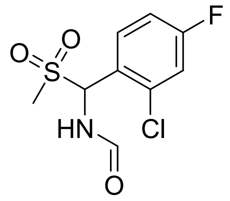

***N*-((2-chloro-4-fluorophenyl)(methylsulfonyl)methyl)formamide**

Obtained as a white solid (692 mg, 2.60 mmol, 91.9%) and used without further purification.

TLC (EtOAc:Hx = 3:2): R_f = 0.30 [UV]

^{1}H NMR (400 MHz, DMSO) δ 10.00 – 9.93 (m, 1H), 8.25 (d, J = 1.3 Hz, 1H), 7.79 (dd, J = 8.9, 6.0 Hz, 1H), 7.60 (dd, J = 8.8, 2.7 Hz, 1H), 7.42 (td, J = 8.5, 2.7 Hz, 1H), 6.72 (d, J = 10.1 Hz, 1H), 3.04 (s, 3H).

^{13}C NMR (101 MHz, DMSO) δ 162.4 (d, J = 250.8 Hz), 161.2, 135.2 (d, J = 11.0 Hz), 131.9 (d, J = 9.6 Hz), 125.0 (d, J = 3.5 Hz), 117.1 (d, J = 25.3 Hz), 115.1 (d, J = 21.7 Hz), 64.6, 38.3.

HRMS (ESI) m/z for $[M+Na]^+$ calcd.: 287.9868, found: 287.9864.

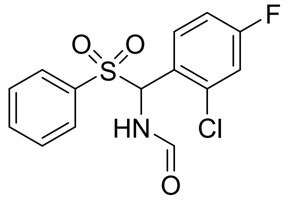

***N*-((2-chloro-4-fluorophenyl)(phenylsulfonyl)methyl)formamide**

Obtained as a white solid (395 mg, 1.21 mmol, 31.9%) after purification by column chromatography (EtOAc:P = 6:4).

TLC (EtOAc:P = 6:4): R_f =0.53 [UV]

^{1}H NMR (400 MHz, DMSO) δ 9.99 (dd, J = 10.4, 1.4 Hz, 1H), 8.03 (d, J = 1.3 Hz, 1H), 7.87 – 7.73 (m, 4H), 7.69 – 7.62 (m, 2H), 7.55 (dd, J = 8.9, 2.7 Hz, 1H), 7.46 (td, J = 8.5, 2.7 Hz, 1H), 6.81 (d, J = 10.4 Hz, 1H).

^{13}C NMR (101 MHz, DMSO) δ 162.5 (d, J = 251.0 Hz), 160.5, 136.0, 135.3 (d, J = 10.9 Hz), 134.7, 132.0 (d, J = 9.6 Hz), 129.5, 128.9, 125.2 (d, J = 3.5 Hz), 116.9 (d, J = 25.3 Hz), 115.2 (d, J = 21.7 Hz), 65.8.

HRMS (ESI) m/z for $[M+Na]^+$ calcd.: 350.0024, found: 350.0006.

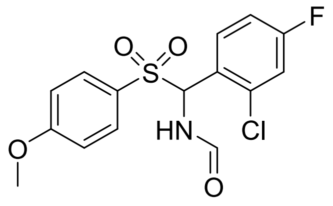

***N*-((2-chloro-4-fluorophenyl)((4-methoxyphenyl)sulfonyl)methyl)formamide**

Obtained as a slightly yellow solid (238 mg, 0.67 mmol, 20.6%) after purification by column chromatography (EtOAc:P = 6:4).

TLC (EtOAc:P = 6:4): R_f =0.40 [UV]

^{1}H NMR (400 MHz, DMSO) δ 9.94 (dd, J = 10.4, 1.4 Hz, 1H), 8.03 (d, J = 1.3 Hz, 1H), 7.80 (dd, J = 8.9, 6.0 Hz, 1H), 7.72 – 7.64 (m, 2H), 7.55 (dd, J = 8.8, 2.7 Hz, 1H), 7.45 (td, J = 8.6, 2.8 Hz, 1H), 7.19 – 7.11 (m, 2H), 6.74 (d, J = 10.4 Hz, 1H), 3.86 (s, 3H).

^{13}C NMR (101 MHz, DMSO) δ 164.0, 162.4 (d, J = 250.6 Hz), 160.5, 135.3 (d, J = 11.2 Hz), 131.9 (d, J = 9.5 Hz), 131.3, 127.2, 125.5 (d, J = 3.6 Hz), 116.9 (d, J = 25.4 Hz), 115.1 (d, J = 21.7 Hz), 114.8, 65.8, 55.9.

HRMS (ESI) m/z for $[M+Na]^+$ calcd.: 380.0130, found: 380.0112.

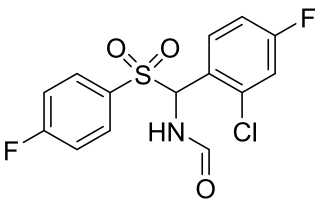

***N*-((2-chloro-4-fluorophenyl)((4-fluorophenyl)sulfonyl)methyl)formamide**

Obtained as a slightly yellow solid (523 mg, 1.51 mmol, 38.8%) after purification by column chromatography (EtOAc:P = 1:1).

TLC (EtOAc:P = 1:1): R_f =0.50 [UV]

^{1}H NMR (400 MHz, DMSO) δ 10.04 – 9.97 (m, 1H), 8.04 (d, J = 1.3 Hz, 1H), 7.98 – 7.77 (m, 3H), 7.60 – 7.34 (m, 4H), 6.80 (d, J = 10.3 Hz, 1H).

^{13}C NMR (101 MHz, DMSO) δ 165.7 (d, J = 254.1 Hz), 165.7, 162.5 (d, J = 251.1 Hz), 160.6, 135.3 (d, J = 10.9 Hz), 132.3 (d, J = 10.1 Hz), 132.2 (d, J = 2.9 Hz), 132.0 (d, J = 9.6 Hz), 124.9 (d, J = 3.5 Hz), 117.0 (d, J = 25.5 Hz), 116.9 (d, J = 23.2 Hz), 115.3 (d, J = 21.7 Hz), 66.0.

HRMS (ESI) m/z for $[M+Na]^+$ calcd.: 367.9930, found: 367.9911.

Isocyanides

General procedures:

The procedures were adapted from published protocols.[24, 25, 29-31]

Method A: Respective formamide (0.3-0.8 mmol) was solubilized in dry tetrahydrofuran (4 mL). Phosphoryl chloride (2 eq.) was added dropwise at room temperature. The mixture was stirred for a few minutes before cooling to 0 °C. Triethylamine (9 eq.) was added dropwise over 15 minutes and the mixture was stirred for another hour at 0 °C. The reaction was quenched by adding saturated sodium hydrogencarbonate solution. The mixture was extracted with diethyl ether and the combined organic layers were washed with brine, dried over sodium sulfate, and concentrated in vacuo. The product was purified by column chromatography or recrystallization in isopropanol.

Method B: Respective formamide (0.64 mmol) was solubilized in dry tetrahydrofuran (4 mL) and then cooled to -40 °C. Phosphoryl chloride (3 eq.) was added dropwise. The mixture was stirred for 40 minutes before triethylamine (5 eq.) was added dropwise over 15 minutes. The mixture was stirred for 2 hours at -40 °C. The reaction was quenched by adding saturated sodium hydrogencarbonate solution. The mixture was extracted with dichloromethane and the combined organic layers were washed with brine, dried over sodium sulfate, and concentrated in vacuo. The product was purified by column chromatography.

Method C: Respective formamide (0.42-0.66 mmol) was solubilized in dry tetrahydrofuran (4 mL) and then cooled to -10 °C. Phosphoryl chloride (3 eq.) was added dropwise. Next, triethylamine (5 eq.) was added dropwise over 15 minutes. The mixture was stirred for 1-3 hours at -5 °C. The reaction was quenched by adding saturated sodium hydrogencarbonate solution. The mixture was extracted with diethyl ether and the combined organic layers were washed with brine, dried over sodium sulfate, and concentrated in vacuo. The product was purified by column chromatography.

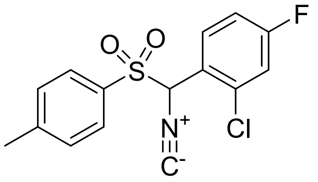

2-chloro-4-fluoro-1-(isocyano(tosyl)methyl)benzene

Synthesis method A. The product was obtained as a slightly yellow solid (105 mg, 0.32 mmol, 55.4%) after purification by column chromatography (P:Et_2O = 7:3).

TLC (P:Et_2O = 4:1): R_f = 0.48 [UV]

^{1}H NMR (400 MHz, DMSO) δ 7.75 – 7.68 (m, 2H), 7.62 (dd, *J* = 8.7, 2.6 Hz, 1H), 7.58 – 7.50 (m, 3H), 7.42 (td, *J* = 8.5, 2.7 Hz, 1H), 7.01 (s, 1H), 2.46 (s, 3H).

^{13}C NMR (101 MHz, DMSO) δ 164.4, 163.0 (d, *J* = 252.5 Hz), 146.9, 135.3 (d, *J* = 11.2 Hz), 131.8 (d, *J* = 10.0 Hz), 130.6, 130.2, 129.9, 121.7 (d, *J* = 3.6 Hz), 117.6 (d, *J* = 25.6 Hz), 115.5 (d, *J* = 22.1 Hz), 71.4, 21.3.

HRMS (EI) m/z for $[M]^+$ calcd.: 323.0178, found: 323.0181.

EA: calcd. for $C_{15}H_{11}ClFNO_2S$: C, 55.65; H, 3.42; N, 4.33; S, 9,90. Found: C, 55.83; H, 3.41; N, 4.28; S, 10,07.

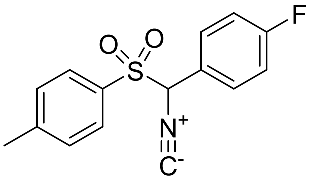

1-fluoro-4-(isocyano(tosyl)methyl)benzene

Synthesis method A. The product was obtained as a slightly yellow solid (161 mg, 0.56 mmol, 86.1%) after purification by column chromatography (P:Et_2O = 7:3).

TLC (P:Et_2O = 7:3): R_f = 0.32 [UV]

^{1}H NMR (500 MHz, DMSO) δ 7.67 (d, J = 8.4 Hz, 2H), 7.53 (d, J = 8.1 Hz, 2H), 7.44 – 7.37 (m, 2H), 7.38 – 7.31 (m, 2H), 2.45 (s, 3H).

^{13}C NMR (126 MHz, DMSO) δ 164.1, 163.4 (d, J = 248.0 Hz), 146.7, 131.0 (d, J = 9.1 Hz), 130.5, 130.1, 129.9, 123.1 (d, J = 2.9 Hz), 116.0 (d, J = 22.3 Hz), 73.8, 21.3.

HRMS (EI) m/z for $[M]^+$ calcd.: 289.0567, found: 289.0586.

These data are in accordance with previous literature reports. Literature spectra were recorded in deuterated chloroform.[26]

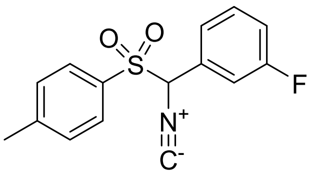

1-fluoro-3-(isocyano(tosyl)methyl)benzene

Synthesis method A. The product was obtained as a yellow solid (51 mg, 0.18 mmol, 27.1%) after purification by column chromatography (P:Et_2O = 7:3).

TLC (P:Et_2O = 7:3): R_f = 0.33 [UV]

^{1}H NMR (400 MHz, DMSO) δ 7.69 (d, J = 8.4 Hz, 1H), 7.60 – 7.49 (m, 2H), 7.41 (tdd, J = 8.6, 2.6, 1.0 Hz, 1H), 7.21 (dt, J = 7.1, 1.1 Hz, 1H), 7.15 (dt, J = 9.5, 2.0 Hz, 1H), 7.04 (s, 1H), 2.46 (s, 2H).

^{13}C NMR (101 MHz, DMSO) δ 164.4, 161.7 (d, J = 245.4 Hz), 146.7, 131.1 (d, J = 7.9 Hz), 130.4, 130.1, 129.8, 129.2 (d, J = 7.8 Hz), 124.7 (d, J = 3.1 Hz), 117.7 (d, J = 20.8 Hz), 115.5 (d, J = 23.5 Hz), 73.8, 21.3.

HRMS (ESI) m/z for $[M-H]^-$ calcd.: 288.0500, found: 288.0497.

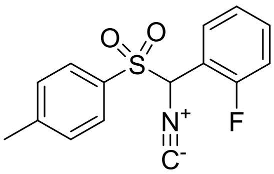

1-fluoro-2-(isocyano(tosyl)methyl)benzene

Synthesis method A. The product was obtained as a yellow solid (156 mg, 0.54 mmol, 83.1%) after purification by column chromatography (P:Et_2O = 7:3).

TLC (P:Et_2O = 7:3): R_f = 0.34 [UV]

^{1}H NMR (500 MHz, DMSO) δ 7.69 (d, J = 8.3 Hz, 2H), 7.64 – 7.56 (m, 1H), 7.53 (d, J = 8.1 Hz, 2H), 7.41 - 7.28 (m, 3H), 7.09 (s, 1H), 2.46 (s, 3H).

^{13}C NMR (101 MHz, DMSO) δ 164.0, 156.0 (d, J = 252.3 Hz), 146.7, 133.3 (d, J = 8.5 Hz), 130.6, 130.1, 129.9, 129.8, 125.1 (d, J = 3.8 Hz), 116.1 (d, J = 20.8 Hz), 114.6 (d, J = 12.9 Hz), 69.8, 21.3.

HRMS (EI) m/z for $[M]^+$ calcd.: 289.0567, found: 289.0589.

These data are in accordance with previous literature reports. Literature spectra were recorded in deuterated chloroform.[28]

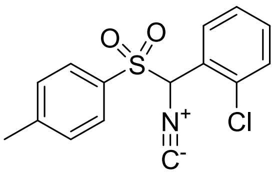

1-chloro-2-(isocyano(tosyl)methyl)benzene

Synthesis method A. The product was obtained as a white solid (80 mg, 0.26 mmol, 42.4%) after purification by column chromatography (Hx:Et_2O = 3:2).

TLC (Hx:Et_2O = 3:2): R_f = 0.48 [UV]

^{1}H NMR (400 MHz, DMSO) δ 7.71 (d, J = 8.3 Hz, 2H), 7.63 – 7.47 (m, 6H), 7.01 (s, 1H), 2.46 (s, 3H).

^{13}C NMR (101 MHz, DMSO) δ 164.2, 146.8, 134.0, 132.6, 130.9, 130.2, 130.1, 129.9, 129.9, 127.9, 125.0, 72.0, 21.3.

HRMS (ESI) m/z for $[M-H]^-$ calcd.: 304.0204, found: 304.0202.

These data are in accordance with previous literature reports. Literature spectra were recorded in deuterated chloroform.[28]

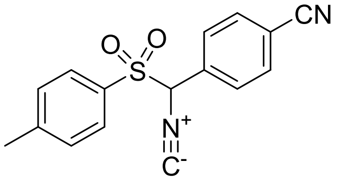

4-(isocyano(tosyl)methyl)benzonitrile

Synthesis method B. The product was obtained as an off-white solid (52 mg, 0.18 mmol, 27.6%) after purification by column chromatography (Hx:EtOAc = 1:1).

TLC (EtOAc:Hx = 3:7): R_f = 0.41 [UV]

¹H NMR (400 MHz, DMSO) δ 7.98 (d, J = 8.4 Hz, 2H), 7.68 (d, J = 8.4 Hz, 2H), 7.60 – 7.51 (m, 4H), 7.17 (s, 1H), 2.46 (s, 3H).

¹³C NMR (101 MHz, DMSO) δ 164.9, 146.9, 132.7, 131.7, 130.2, 130.2, 129.9, 129.4, 118.0, 113.4, 74.0, 21.2.

HRMS (EI) m/z for $[M]^+$ calcd.: 296.0614, found: 296.0609.

These data are in accordance with previous literature reports. Literature spectra were recorded in deuterated chloroform.[29]

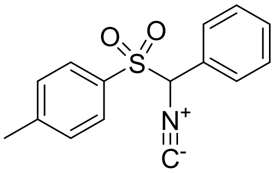

1-((isocyano(phenyl)methyl)sulfonyl)-4-methylbenzene

Synthesis method A. The product was obtained as an off-white solid (55 mg, 0.20 mmol, 29.4%) after recrystallization in isopropanol.

TLC (EtOAc:P = 3:2): R_f = 0.84 [UV]

¹H NMR (300 MHz, DMSO) δ 7.71 – 7.62 (m, 2H), 7.59 – 7.43 (m, 5H), 7.48 – 7.32 (m, 2H), 6.98 (s, 1H), 2.45 (s, 3H).

¹³C NMR (75 MHz, DMSO) δ 163.9, 146.4, 130.7, 130.6, 123.0, 129.8, 128.7, 128.6, 126.8, 74.5, 21.2.

HRMS (EI) m/z for $[M]^+$ calcd.: 271.0662, found: 271.0658.

These data are in accordance with previous literature reports. Literature spectra were recorded in deuterated chloroform.[28]

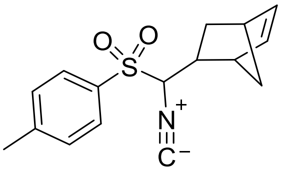

5-(isocyano(tosyl)methyl)bicyclo[2.2.1]hept-2-ene

Synthesis method A. The product was obtained as a beige oil (90 mg, 0.31 mmol, 47.8%) after purification by column chromatography (P:Et_2O = 7:3).

TLC (P:Et_2O = 7:3): R_f = 0.68[UV]

¹H NMR (400 MHz, DMSO) δ 7.89 – 7.82 (m, 2H), 7.58 – 7.50 (m, 2H), 6.32 – 6.19 (m, 1H), 5.98 – 5.86 (m, 1H), 5.09 – 4.77 (m, 1H), 3.17 – 2.97 (m, 1H), 2.90 – 2.78 (m, 1H), 2.72 – 2.52 (m, 1H), 2.45 (s, 3H), 1.99 – 1.81 (m, 1H), 1.39 – 1.24 (m, 2H), 1.07 – 0.80 (m, 1H).

¹³C NMR (101 MHz, DMSO) δ 163.5, 163.2, 146.2, 139.4, 138.6, 131.7, 131.6, 131.6, 130.7, 130.1, 130.0, 129.7, 129.6, 75.7, 75.2, 49.0, 47.7, 45.9, 44.9, 42.5, 41.3, 38.1, 37.8, 29.8, 29.4, 21.2.

The spectral complexity results from stereoisomers of the bicyclic ring system.

HRMS (ESI) m/z for $[M+H]^+$ calcd: 288.1053, found: 288.1049.

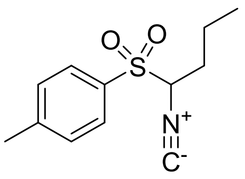

1-((1-isocyanobutyl)sulfonyl)-4-methylbenzene

Synthesis method A. The product was obtained as an orange oil (153 mg, 0.64 mmol, 82.3%) after purification by column chromatography (P:Et_2O = 7:3).

TLC (P:Et_2O = 7:3): R_f = 0.53[UV]

¹H NMR (400 MHz, DMSO) δ 7.88 – 7.81 (m, 2H), 7.60 – 7.52 (m, 2H), 5.62 (dd, J = 10.4, 3.6 Hz, 1H), 2.46 (s, 3H), 2.01 – 1.88 (m, 1H), 1.77 – 1.63 (m, 1H), 1.60 – 1.33 (m, 2H), 0.92 (t, J = 7.4 Hz, 3H).

¹³C NMR (101 MHz, DMSO) δ 163.0, 146.3, 131.2, 130.2, 129.3, 71.5, 29.6, 21.2, 18.1, 12.9.

HRMS (EI) m/z for $[M]^+$ calcd.: 237.0818, found: 237.0798.

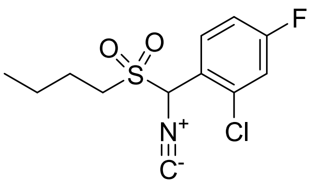

1-((butylsulfonyl)(isocyano)methyl)-2-chloro-4-fluorobenzene

Synthesis method A. The product was obtained as an orange oil (41 mg, 0.14 mmol, 43.6%) after purification by column chromatography (P:Et_2O = 7:3).

TLC (P:Et_2O = 7:3): R_f = 0.77 [UV]

¹H NMR (400 MHz, DMSO) δ 7.72 (dd, J = 8.9, 5.9 Hz, 1H), 7.67 (dd, J = 8.7, 2.6 Hz, 1H), 7.46 (td, J = 8.5, 2.7 Hz, 1H), 7.01 (s, 1H), 3.60 – 3.49 (m, 2H), 1.80 (tdd, J = 14.4, 6.9, 1.5 Hz, 2H), 1.47 (hd, J = 7.4, 2.0 Hz, 2H), 0.93 (t, J = 7.4 Hz, 3H).

¹³C NMR (101 MHz, DMSO) δ 164.4, 163.05 (d, J = 252.6 Hz), 135.2 (d, J = 11.0 Hz), 132.0 (d, J = 10.0 Hz), 121.2 (d, J = 3.5 Hz), 117.6 (d, J = 25.7 Hz), 115.6 (d, J = 22.1 Hz), 68.6, 50.11, 23.2, 20.9, 13.4.

HRMS (EI) m/z for $[M]^+$ calcd.: 289.0334, found: 289.0350.

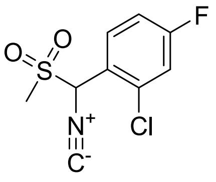

2-chloro-4-fluoro-1-(isocyano(methylsulfonyl)methyl)benzene

Synthesis method A. The product was obtained as a white solid (74 mg, 0.30 mmol, 39.7%) after purification by column chromatography (EtOAc:Hx = 1:1).

TLC (EtOAc:Hx= 1:1): R_f = 0.36 [UV]

¹H NMR (400 MHz, DMSO) δ 7.73 (dd, J = 8.9, 5.9 Hz, 1H), 7.67 (dd, J = 8.8, 2.7 Hz, 1H), 7.46 (td, J = 8.5, 2.7 Hz, 1H), 7.04 (s, 1H), 3.40 (s, 3H).

¹³C NMR (101 MHz, DMSO) δ 134.3, 163.1 (d, J = 252.4 Hz), 135.2 (d, J = 11.3 Hz), 132.0 (d, J = 10.0 Hz), 121.2 (d, J = 3.5 Hz), 117.6 (d, J = 25.6 Hz), 115.6 (d, J = 22.0 Hz), 69.3, 38.0.

HRMS (ESI) m/z for $[M-H]^-$ calcd: 245.9797, found: 245.9792.

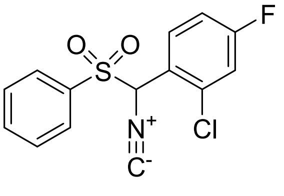

2-chloro-4-fluoro-1-(isocyano(phenylsulfonyl)methyl)benzene

Synthesis method C. The product was obtained as an off white solid (81 mg, 0.26 mmol, 39.4%) after purification by column chromatography (P:Et_2O = 7:3).

TLC (P:Et_2O = 7:3): R_f = 0.48 [UV]

¹H NMR (400 MHz, DMSO) δ 7.95 – 7.87 (m, 1H), 7.87 – 7.80 (m, 2H), 7.80 – 7.70 (m, 2H), 7.61 (dd, J = 8.8, 2.7 Hz, 1H), 7.57 (dd, J = 8.9, 5.9 Hz, 1H), 7.43 (td, J = 8.5, 2.6 Hz, 1H), 7.07 (s, 1H).

^{13}C NMR (101 MHz, DMSO) δ 164.6, 163.1 (d, J = 252.6 Hz), 135.9, 135.3 (d, J = 11.2 Hz), 133.6, 131.9 (d, J = 10.1 Hz), 129.9, 129.8, 121.6 (d, J = 3.5 Hz), 117.6 (d, J = 25.7 Hz), 115.6 (d, J = 22.1 Hz), 71.4.

HRMS (ESI) m/z for $[M-H]^-$ calcd: 307.9954, found: 307.9951.

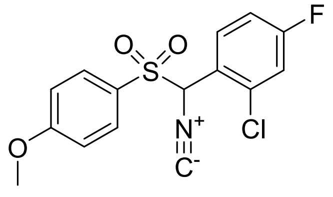

2-chloro-4-fluoro-1-(isocyano((4-methoxyphenyl)sulfonyl)methyl)benzene

Synthesis method C. The product was obtained as an orange solid (85 mg, 0.25 mmol, 59.5%) after purification by column chromatography (P:Et_2O = 6:4).

TLC (P:Et_2O = 6:4): R_f = 0.32 [UV]

^{1}H NMR (400 MHz, DMSO) δ 7.76 – 7.68 (m, 2H), 7.60 (dd, J = 8.8, 2.6 Hz, 1H), 7.52 (dd, J = 8.9, 5.9 Hz, 1H), 7.42 (td, J = 8.5, 2.6 Hz, 1H), 7.28 – 7.19 (m, 2H), 6.97 (s, 1H), 3.90 (s, 3H).

^{13}C NMR (101 MHz, DMSO) δ 164.89, 164.2, 163.0 (d, J = 250.8 Hz), 135.2 (d, J = 11.1 Hz), 132.4, 131.7 (d, J = 10.0 Hz), 124.5, 122.0 (d, J = 3.5 Hz), 117.5 (d, J = 25.6 Hz), 115.5 (d, J = 21.9 Hz), 115.0, 71.5, 56.1.

HRMS (ESI) m/z for $[M-H]^-$ calcd: 338.0059, found: 338.0058.

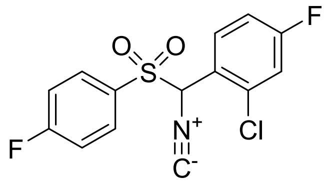

2-chloro-4-fluoro-1-(((4-fluorophenyl)sulfonyl)(isocyano)methyl)benzene

Synthesis method C. The product was obtained as a yellow solid (62 mg, 0.19 mmol, 36.5%) after purification by column chromatography (P:Et_2O = 7:3).

TLC (P:Et_2O = 7:3): R_f = 0.59 [UV]

^{1}H NMR (400 MHz, DMSO) δ 7.95 – 7.85 (m, 2H), 7.66 – 7.53 (m, 4H), 7.43 (td, J = 8.5, 2.7 Hz, 1H), 7.12 (s, 1H).

^{13}C NMR (101 MHz, DMSO) δ 166.4 (d, J = 255.8 Hz), 164.7, 163.1 (d, J = 252.8 Hz), 135.3 (d, J = 11.2 Hz), 133.4 (d, J = 10.5 Hz), 131.9 (d, J = 10.0 Hz), 129.8 (d, J = 3.0 Hz), 121.5 (d, J = 3.7 Hz), 117.7 (d, J = 25.7 Hz), 117.2 (d, J = 23.2 Hz), 115.6 (d, J = 22.2 Hz), 71.5.

HRMS (ESI) m/z for $[M-H]^-$ calcd: 325.3860, found: 325.9857.

Benzyl aryl sulfones

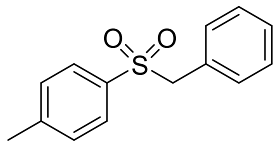

1-(benzylsulfonyl)-4-methylbenzene

According to a published procedure[32], *p*-toluenesulfinic acid sodium salt (3.96 g, 22.2 mmol, 1.1 eq.) was dissolved in 20 mL 1,2-dimethoxyethane at room temperature. Benzyl chloride (2.3 mL, 20.0 mmol, 1 eq.) and tetrabutylammonium bromide (350 mg, 1.09 mmol, 0.05 eq.) were added, and the mixture was stirred at 80 °C for 3

TLC (P:EtOAc = 8:2): R_f = 0.43 [UV]

^{1}H NMR (500 MHz, CDCl3) δ 7.60 – 7.47 (m, 2H), 7.37 – 7.31 (m, 1H), 7.31 – 7.23 (m, 4H), 7.14 – 7.08 (m, 2H), 4.31 (s, 2H), 2.43 (s, 3H).

^{13}C NMR (101 MHz, CDCl3) δ 144.8, 135.1, 131.0, 129.6, 128.8, 128.8, 128.7, 128.4, 63.1, 21.8.

These data are in accordance with previous literature reports.[32]

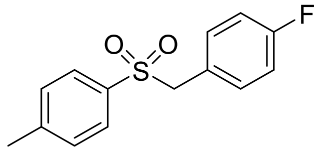

1-fluoro-4-(tosylmethyl)benzene

According to a published procedure[32], *p*-toluenesulfinic acid sodium salt (3.95 g, 22.2 mmol, 1.1 eq.) was dissolved in 20 mL 1,2-dimethoxyethane at room temperature. 4-Fluorobenzyl chloride (2.4 mL, 20.1 mmol, 1 eq.) and tetrabutylammonium bromide (346 mg, 1.07 mmol, 0.05 eq.) were added, and the mixture was stirred at 80 °C for 3

TLC (P:EtOAc = 8:2): R_f = 0.53 [UV]

^{1}H NMR (400 MHz, CDCl3) δ 7.53 – 7.47 (m, 2H), 7.25 (dd, J = 8.5, 2.2 Hz, 2H), 7.11 – 7.01 (m, 2H), 6.99 – 6.90 (m, 2H), 4.25 (s, 2H), 2.41 (s, 3H).

^{13}C NMR (101 MHz, CDCl3) δ 163.1 (d, J = 248.4 Hz), 145.0, 134.9, 132.7 (d, J = 8.6 Hz), 129.7, 128.7, 124.3 (d, J = 3.3 Hz), 115.7 (d, J = 21.7 Hz), 62.1, 21.7.

These data are in accordance with previous literature reports.[32]

Supplementary references

1. B. A. Sampson, R. Misra and S. A. Benson, *Genetics*, 1989, **122**, 491-501.
2. I. Hübner, J. A. Shapiro, J. Hoßmann, J. Drechsel, S. M. Hacker, P. N. Rather, D. H. Pieper, W. M. Wuest and S. A. Sieber, *ACS Central Science*, 2021, **7**, 488-498.
3. T. Reinhardt, K. M. Lee, L. Niederegger, C. R. Hess and S. A. Sieber, *ACS Chemical Biology*, 2022, **17**, 3077-3085.
4. P. Le, E. Kunold, R. Macsics, K. Rox, M. C. Jennings, I. Ugur, M. Reinecke, D. Chaves-Moreno, M. W. Hackl, C. Fetzer, F. A. M. Mandl, J. Lehmann, V. S. Korotkov, S. M. Hacker, B. Kuster, I. Antes, D. H. Pieper, M. Rohde, W. M. Wuest, E. Medina and S. A. Sieber, *Nature Chemistry*, 2020, **12**, 145-158.
5. A. G. Elliott, J. X. Huang, S. Neve, J. Zuegg, I. A. Edwards, A. K. Cain, C. J. Boinett, L. Barquist, C. V. Lundberg, J. Steen, M. S. Butler, M. Mobli, K. M. Porter, M. A. T. Blaskovich, S. Lociuro, M. Strandh and M. A. Cooper, *Nature Communications*, 2020, **11**, 3184.
6. E. Tarrant, G. P. Riboldi, M. R. McIlvin, J. Stevenson, A. Barwinska-Sendra, L. J. Stewart, M. A. Saito and K. J. Waldron, *Metallomics*, 2019, **11**, 183-200.
7. M. Zhu, L. Wang, W. Zhang, Z. Liu, M. Ali, M. Imtiaz and J. He, *Journal of Natural Products*, 2020.
8. J. Cox, M. Y. Hein, C. A. Luber, I. Paron, N. Nagaraj and M. Mann, *Molecular & Cellular Proteomics*, 2014, **13**, 2513-2526.
9. S. Tyanova, T. Temu, P. Sinitcyn, A. Carlson, M. Y. Hein, T. Geiger, M. Mann and J. Cox, *Nature Methods*, 2016, **13**, 731-740.
10. D. Szklarczyk, R. Kirsch, M. Koutrouli, K. Nastou, F. Mehryary, R. Hachilif, A. L. Gable, T. Fang, Nadezhda T. Doncheva, S. Pyysalo, P. Bork, Lars J. Jensen and C. von Mering, *Nucleic Acids Research*, 2023, **51**, D638-D646.
11. D. Szklarczyk, A. L. Gable, D. Lyon, A. Junge, S. Wyder, J. Huerta-Cepas, M. Simonovic, N. T. Doncheva, J. H. Morris, P. Bork, L. J. Jensen and Christian v. Mering, *Nucleic Acids Research*, 2019, **47**, D607-D613.
12. P. R. A. Zanon, L. Lewald and S. M. Hacker, *Angewandte Chemie International Edition*, 2020, **59**, 2829-2836.
13. K. M. Lee, P. Le, S. A. Sieber and S. M. Hacker, *Chemical Communications*, 2020, **56**, 2929-2932.
14. J. Cox and M. Mann, *Nature Biotechnology*, 2008, **26**, 1367-1372.
15. R. R. Chaudhuri, A. G. Allen, P. J. Owen, G. Shalom, K. Stone, M. Harrison, T. A. Burgis, M. Lockyer, J. Garcia-Lara, S. J. Foster, S. J. Pleasance, S. E. Peters, D. J. Maskell and I. G. Charles, *BMC Genomics*, 2009, **10**, 291.
16. C. The UniProt, *Nucleic Acids Research*, 2019, **47**, D506-D515.
17. P. Gaudet, M. S. Livstone, S. E. Lewis and P. D. Thomas, *Briefings in Bioinformatics*, 2011, **12**, 449-462.
18. H. Franken, T. Mathieson, D. Childs, G. M. A. Sweetman, T. Werner, I. Tögel, C. Doce, S. Gade, M. Bantscheff, G. Drewes, F. B. M. Reinhard, W. Huber and M. M. Savitski, *Nature Protocols*, 2015, **10**, 1567-1593.
19. W. Huber, V. J. Carey, R. Gentleman, S. Anders, M. Carlson, B. S. Carvalho, H. C. Bravo, S. Davis, L. Gatto, T. Girke, R. Gottardo, F. Hahne, K. D. Hansen, R. A. Irizarry, M. Lawrence, M. I. Love, J. MacDonald, V. Obenchain, A. K. Oleś, H. Pagès, A. Reyes, P. Shannon, G. K. Smyth, D. Tenenbaum, L. Waldron and M. Morgan, *Nature Methods*, 2015, **12**, 115-121.
20. C. Richez, J. Boetzel, N. Floquet, K. Koteshwar, J. Stevens, B. Badet and M.-A. Badet-Denisot, *Protein Expression and Purification*, 2007, **54**, 45-53.
21. S. Ruegenberg, F. A. M. C. Mayr, I. Atanassov, U. Baumann and M. S. Denzel, *Nature Communications*, 2021, **12**, 2176.
22. P. Edwards, J. Sabo Nelsen, J. G. Metz and K. Dehesh, *FEBS Letters*, 1997, **402**, 62-66.
23. D. Bhatt, H. Y. Kim and K. Oh, *Organic Letters*, 2023, **25**, 5697-5701.
24. U. Velaparthi, C. P. Darne, J. Warrier, P. Liu, H. Rahaman, K. Augustine-Rauch, K. Parrish, Z. Yang, J. Swanson, J. Brown, G. Dhar, A. Anandam, V. K. Holenarsipur, K. Palanisamy, B. S. Wautlet, M. P. Fereshteh, J. Lippy, A. J. Tebben, S. Sheriff, M. Ruzanov, C. Yan, A. Gupta, A. K.

Gupta, M. Vetrichelvan, A. Mathur, M. Gelman, R. Singh, T. Kinsella, A. Murtaza, J. Fargnoli, G. Vite and R. M. Borzilleri, *ACS Medicinal Chemistry Letters*, 2020, **11**, 172-178.

25. J. Sisko, M. Mellinger, P. W. Sheldrake and N. H. Baine, *Tetrahedron Letters*, 1996, **37**, 8113-8116.
26. A. Divakaran, S. K. Talluri, A. M. Ayoub, N. K. Mishra, H. Cui, J. C. Widen, N. Berndt, J.-Y. Zhu, A. S. Carlson, J. J. Topczewski, E. K. Schonbrunn, D. A. Harki and W. C. K. Pomerantz, *Journal of Medicinal Chemistry*, 2018, **61**, 9316-9334.
27. C. M. Gallati, S. K. Goetzfried, M. Ausserer, J. Sagasser, M. Plangger, K. Wurst, M. Hermann, D. Baecker, B. Kircher and R. Gust, *Dalton Transactions*, 2020, **49**, 5471-5481.
28. S. Fallarini, A. Massarotti, A. Gesù, S. Giovarruscio, G. Coda Zabetta, R. Bergo, B. Giannelli, A. Brunco, G. Lombardi, G. Sorba and T. Pirali, *MedChemComm*, 2016, **7**, 409-419.
29. H. Yu, S. Li, K. E. Schwieter, Y. Liu, B. Sun, J. S. Moore and C. M. Schroeder, *Journal of the American Chemical Society*, 2020, **142**, 4852-4861.
30. F. Saccoliti, V. N. Madia, V. Tudino, A. De Leo, L. Pescatori, A. Messore, D. De Vita, L. Scipione, R. Brun, M. Kaiser, P. Mäser, C. M. Calvet, G. K. Jennings, L. M. Podust, G. Pepe, R. Cirilli, C. Faggi, A. Di Marco, M. R. Battista, V. Summa, R. Costi and R. Di Santo, *Journal of Medicinal Chemistry*, 2019, **62**, 1330-1347.
31. S. A. Salami, X. Siwe-Noundou and R. W. Krause, *Journal*, 2022, **27**.
32. T. Niwa, H. Yorimitsu and K. Oshima, *Tetrahedron*, 2009, **65**, 1971-1976.

Appendix

Amino acid sequences of expressed proteins

S. aureus NCTC 8325 Glutamine-fructose-6-phosphate aminotransferase (UniProt ID: Q2FWA0)

Wild type sequence:

MCGIVGYIGYDNAKELLLKGLEKLEYRGYDSAGIAVVNDDNTTVFKEKGRIAELRKVADSSDFDGPVG
IGHTRWATHGVPNHENSHPHQSSNGRFTLVHNGVIENYEELKGEYLQGVSFISETDTEVIVQLVEYFS
NQGLSTEEAFTKVVSLLHGSYALGLLDAEDKDTIYVAKNKSPLLLGVGEGFNVIASDALAMLQVTSEY
KEIHDHEIVIVKKDEVIIKDADGNVVERDSYIAEIDASDAEKGVYAHYMLKEIHEQPAVMRRIIQEYQ
DAEGNLKIDQDIINDVKEADRIYVIAAGTSYHAGLVGKEFLEKWAGVPTEVHVASEFVYNMPLLSEKP
LFVYISQSGETADSRAVLVETNKLGHKSLTITNVAGSTLSREADHTLLLHAGPEIAVASTKAYTAQIA
VLSILSQIVAKEHGREADIDLLRELAKVTTAIEAIVDDAPIMEQIATDFLETTRNAFFIGRTIDYNVS
LEGALKLKEISYIQAEGFAGGELKHGTIALIEEGTPVVGLATQEKVNLSIRGNVKEVVARGAHPCIIS
MEGLEKEGDTYVIPHVHELLTPLVSVVALQLISYYAALHRDLDVDKPRNLAKSVTVE

Expressed amino acid sequence from pET301/CT-Dest plasmid (GlmS):

CGIVGYIGYDNAKELLLKGLEKLEYRGYDSAGIAVVNDDNTTVFKEKGRIAELRKVADSSDFDGPVGI
GHTRWATHGVPNHENSHPHQSSNGRFTLVHNGVIENYEELKGEYLQGVSFISETDTEVIVQLVEYFSN
QGLSTEEAFTKVVSLLHGSYALGLLDAEDKDTIYVAKNKSPLLLGVGEGFNVIASDALAMLQVTSEYK
EIHDHEIVIVKKDEVIIKDADGNVVERDSYIAEIDASDAEKGVYAHYMLKEIHEQPAVMRRIIQEYQD
AEGNLKIDQDIINDVKEADRIYVIAAGTSYHAGLVGKEFLEKWAGVPTEVHVASEFVYNMPLLSEKPL
FVYISQSGETADSRAVLVETNKLGHKSLTITNVAGSTLSREADHTLLLHAGPEIAVASTKAYTAQIAV
LSILSQIVAKEHGREADIDLLRELAKVTTAIEAIVDDAPIMEQIATDFLETTRNAFFIGRTIDYNVSL
EGALKLKEISYIQAEGFAGGELKHGTIALIEEGTPVVGLATQEKVNLSIRGNVKEVVARGAHPCIISM
EGLEKEGDTYVIPHVHELLTPLVSVVALQLISYYAALHRDLDVDKPRNLAKSVTVEENLYFQGHPAFL
YKVVIMHHHHHH

After TEV cleavage:

CGIVGYIGYDNAKELLLKGLEKLEYRGYDSAGIAVVNDDNTTVFKEKGRIAELRKVADSSDFDGPVGI
GHTRWATHGVPNHENSHPHQSSNGRFTLVHNGVIENYEELKGEYLQGVSFISETDTEVIVQLVEYFSN
QGLSTEEAFTKVVSLLHGSYALGLLDAEDKDTIYVAKNKSPLLLGVGEGFNVIASDALAMLQVTSEYK
EIHDHEIVIVKKDEVIIKDADGNVVERDSYIAEIDASDAEKGVYAHYMLKEIHEQPAVMRRIIQEYQD
AEGNLKIDQDIINDVKEADRIYVIAAGTSYHAGLVGKEFLEKWAGVPTEVHVASEFVYNMPLLSEKPL
FVYISQSGETADSRAVLVETNKLGHKSLTITNVAGSTLSREADHTLLLHAGPEIAVASTKAYTAQIAV
LSILSQIVAKEHGREADIDLLRELAKVTTAIEAIVDDAPIMEQIATDFLETTRNAFFIGRTIDYNVSL
EGALKLKEISYIQAEGFAGGELKHGTIALIEEGTPVVGLATQEKVNLSIRGNVKEVVARGAHPCIISM
EGLEKEGDTYVIPHVHELLTPLVSVVALQLISYYAALHRDLDVDKPRNLAKSVTVEENLYFQ

Expressed amino acid sequence from pET-28a(+) plasmid (point mutant GlmS_C2A):

AGIVGYIGYDNAKELLLKGLEKLEYRGYDSAGIAVVNDDNTTVFKEKGRIAELRKVADSSDFDGPVGI
GHTRWATHGVPNHENSHPHQSSNGRFTLVHNGVIENYEELKGEYLQGVSFISETDTEVIVQLVEYFSN
QGLSTEEAFTKVVSLLHGSYALGLLDAEDKDTIYVAKNKSPLLLGVGEGFNVIASDALAMLQVTSEYK
EIHDHEIVIVKKDEVIIKDADGNVVERDSYIAEIDASDAEKGVYAHYMLKEIHEQPAVMRRIIQEYQD
AEGNLKIDQDIINDVKEADRIYVIAAGTSYHAGLVGKEFLEKWAGVPTEVHVASEFVYNMPLLSEKPL
FVYISQSGETADSRAVLVETNKLGHKSLTITNVAGSTLSREADHTLLLHAGPEIAVASTKAYTAQIAV
LSILSQIVAKEHGREADIDLLRELAKVTTAIEAIVDDAPIMEQIATDFLETTRNAFFIGRTIDYNVSL
EGALKLKEISYIQAEGFAGGELKHGTIALIEEGTPVVGLATQEKVNLSIRGNVKEVVARGAHPCIISM
EGLEKEGDTYVIPHVHELLTPLVSVVALQLISYYAALHRDLDVDKPRNLAKSVTVEENLYFQGLEHHH
HHH

After TEV cleavage:

AGIVGYIGYDNAKELLLKGLEKLEYRGYDSAGIAVVNDDNTTVFKEKGRIAELRKVADSSDFDGPVGI
GHTRWATHGVPNHENSHPHQSSNGRFTLVHNGVIENYEELKGEYLQGVSFISETDTEVIVQLVEYFSN
QGLSTEEAFTKVVSLLHGSYALGLLDAEDKDTIYVAKNKSPLLLGVGEGFNVIASDALAMLQVTSEYK

EIHDHEIVIVKKDEVIIKDADGNVVERDSYIAEIDASDAEKGVYAHYMLKEIHEQPAVMRRIIQEYQD
AEGNLKIDQDIINDVKEADRIYVIAAGTSYHAGLVGKEFLEKWAGVPTEVHVASEFVYNMPLLSEKPL
FVYISQSGETADSRAVLVETNKLGHKSLTITNVAGSTLSREADHTLLLHAGPEIAVASTKAYTAQIAV
LSILSQIVAKEHGREADIDLLRELAKVTTAIEAIVDDAPIMEQIATDFLETTRNAFFIGRTIDYNVSL
EGALKLKEISYIQAEGFAGGELKHGTIALIEEGTPVVGLATQEKVNLSIRGNVKEVVARGAHPCIISM
EGLEKEGDTYVIPHVHELLTPLVSVVALQLISYYAALHRDLDVDKPRNLAKSVTVEENLYFQ

S. aureus NCTC 8325 3-oxoacyl-[acyl-carrier-protein] synthase 2 (UniProt ID: Q2FZR9)

Wild type sequence:

MSQNKRVVITGMGALSPIGNDVKTTWENALKGVNGIDKITRIDTEPYSVHLAGELKNFNIEDHIDKKE
ARRMDRFTQYAIVAAREAVKDAQLDINENTADRIGVWIGSGIGGMETFEIAHKQLMDKGPRRVSPFFV
PMLIPDMATGQVSIDLGAKGPNGATVTACATGTNSIGEAFKIVQRGDADAMITGGTEAPITHMAIAGF
SASRALSTNDDIETACRPFQEGRDGFVMGEGAGILVIESLESAQARGANIYAEIVGYGTTGDAYHITA
PAPEGEGGSRAMQAAMDDAGIEPKDVQYLNAHGTSTPVGDLNEVKAIKNTFGEAAKHLKVSSTKSMTG
HLLGATGGIEAIFSALSIKDSKVAPTIHAVTPDPECDLDIVPNEAQDLDITYAMSNSLGFGGHNAVLV
FKKFEA

Expressed amino acid sequence from pET300/NT-Dest plasmid (FabF):

MHHHHHHITSLYKKAGFENLYFQGMSQNKRVVITGMGALSPIGNDVKTTWENALKGVNGIDKITRIDT
EPYSVHLAGELKNFNIEDHIDKKEARRMDRFTQYAIVAAREAVKDAQLDINENTADRIGVWIGSGIGG
METFEIAHKQLMDKGPRRVSPFFVPMLIPDMATGQVSIDLGAKGPNGATVTACATGTNSIGEAFKIVQ
RGDADAMITGGTEAPITHMAIAGFSASRALSTNDDIETACRPFQEGRDGFVMGEGAGILVIESLESAQ
ARGANIYAEIVGYGTTGDAYHITAPAPEGEGGSRAMQAAMDDAGIEPKDVQYLNAHGTSTPVGDLNEV
KAIKNTFGEAAKHLKVSSTKSMTGHLLGATGGIEAIFSALSIKDSKVAPTIHAVTPDPECDLDIVPNE
AQDLDITYAMSNSLGFGGHNAVLVFKKFEA

Expressed amino acid sequence from pET-28a(+) plasmid (point mutant FabF_C165A):

GSSHHHHHHSSGLVPRGSHMMSQNKRVVITGMGALSPIGNDVKTTWENALKGVNGIDKITRIDTEPYS
VHLAGELKNFNIEDHIDKKEARRMDRFTQYAIVAAREAVKDAQLDINENTADRIGVWIGSGIGGMETF
EIAHKQLMDKGPRRVSPFFVPMLIPDMATGQVSIDLGAKGPNGATVTA**A**ATGTNSIGEAFKIVQRGDA
DAMITGGTEAPITHMAIAGFSASRALSTNDDIETACRPFQEGRDGFVMGEGAGILVIESLESAQARGA
NIYAEIVGYGTTGDAYHITAPAPEGEGGSRAMQAAMDDAGIEPKDVQYLNAHGTSTPVGDLNEVKAIK
NTFGEAAKHLKVSSTKSMTGHLLGATGGIEAIFSALSIKDSKVAPTIHAVTPDPECDLDIVPNEAQDL
DITYAMSNSLGFGGHNAVLVFKKFEA

Sequences of plasmids used in this study

pET301/CT-Dest_GlmS

GATCTCGATCCCGCGAAATTAATACGACTCACTATAGGGGAATTGTGAGCGGATAACAATTCCCCTCTAGAAATAATTTTGTTTAAACTTTAAA
TATACATAATCACAAGTTTGTACAAAAAAGCAGGCTTTGAAGGAGATAGAACCATGTGTGGAATTGTTGGTTATATTGGCTATGATAATGCCAA
AGAATTATTATTAAAAGGTTTAGAAAAATTAGAATACAGAGGTTATGACTCTGCAGGTATCGCAGTAGTAAATGATGATAATACAACTGTATTT
AAAGAAAAAGGTCGTATTGCAGAATTACGTAAAGTTGCTGATAGTAGCGATTTTGATGGACCTGTTGGAATCGGTCACACACGTTGGGCAACAC
ACGGTGTACCGAATCATGAAAACTCTCATCCACATCAATCATCAAATGGCCGTTTTACTCTAGTTCATAACGGTGTTATTGAAAACTATGAAGA
GTTAAAAGGTGAATACTTACAAGGTGTATCATTCATTTCAGAAACAGATACAGAAGTTATCGTTCAATTAGTTGAATACTTTTCAAATCAAGGA
CTTTCAACTGAAGAAGCATTTACAAAAGTTGTGTCATTATTACATGGTTCATATGCATTAGGTTTATTAGATGCTGAAGACAAAGACACAATCT
ATGTTGCTAAAAATAAATCACCATTATTATTAGGTGTTGGTGAAGGTTTCAATGTTATCGCATCAGACGCACTTGCAATGTTACAAGTGACAAG
CGAATATAAAGAAATCCATGACCATGAAATCGTTATTGTTAAAAAAGATGAAGTTATTATTAAAGATGCAGATGGAAACGTTGTAGAACGTGAT
TCATATATTGCTGAAATTGATGCATCAGATGCTGAAAAAGGTGTTTATGCACACTACATGTTAAAAGAAATTCATGAACAACCAGCAGTAATGC
GTCGTATTATTCAAGAATATCAAGATGCAGAAGGTAACTTGAAAATTGATCAAGACATCATCAATGATGTTAAAGAAGCAGACCGCATTTACGT
TATTGCAGCAGGTACAAGCTACCATGCAGGTTTAGTAGGTAAAGAATTTTTAGAAAAATGGGCTGGCGTACCAACTGAAGTACACGTTGCATCA
GAGTTTGTCTACAACATGCCATTATTATCTGAAAAACCATTGTTCGTTTATATTTCTCAATCAGGTGAAACTGCAGATAGCCGCGCCGTATTAG
TTGAAACTAATAAATTAGGTCATAAATCATTAACAATCACTAATGTTGCAGGTTCAACTTTATCACGTGAAGCAGACCACACATTGTTATTACA
CGCGGGTCCTGAAATCGCAGTTGCATCTACAAAAGCATATACTGCACAAATTGCAGTATTATCAATCTTGTCTCAAATCGTTGCAAAAGAGCAT
GGTCGTGAAGCAGATATTGATTTATTGAGAGAATTAGCAAAAGTAACAACAGCAATAGAAGCAATTGTTGACGATGCACCAATTATGGAACAAA
TTGCTACAGATTTCTTAGAAACAACACGCAATGCATTCTTTATCGGACGTACTATTGACTATAACGTAAGTTTAGAAGGTGCGTTAAAACTTAA
AGAAATTTCTTACATTCAAGCAGAAGGTTTTGCTGGTGGAGAACTTAAACATGGTACAATTGCCTTAATCGAAGAAGGTACACCAGTTGTAGGT
TTAGCAACACAAGAGAAAGTTAATTTATCAATTCGTGGTAACGTTAAAGAGGTAGTAGCACGTGGTGCACATCCATGTATTATTTCTATGGAGG
GTCTTGAAAAAGAAGGCGACACTTATGTCATTCCTCATGTACATGAATTGTTAACGCCATTAGTATCAGTGGTTGCATTACAATTAATTTCATA
CTATGCAGCATTACACAGAGATTTAGATGTTGATAAACCACGTAACCTTGCTAAATCAGTTACTGTGGAAGAGAATCTTTATTTTCAGGGCCAC

CCAGCTTTCTTGTACAAAGTGGTGATTATGCATCATCATCATCATCACTAGTAATCGGATCCGGCTGCTAACAAAGCCCGAAAGGAAGCTGAGT
TGGCTGCTGCCACCGCTGAGCAATAACTAGCATAACCCCTTGGGGCCTCTAAACGGGTCTTGAGGGGTTTTTTGCTGAAAGGAGGAACTATATC
CGGATATCCCGCAAGAGGCCCGGCAGTACCGGCATAACCAAGCCTATGCCTACAGCATCCAGGGTGACGGTGCCGAGGATGACGATGAGCGCAT
TGTTAGATTTCATACACGGTGCCTGACTGCGTTAGCAATTTAACTGTGATAAACTACCGCATTAAAGCTAGCTTATCGATGATAAGCTGTCAAA
CATGAGAATTAATTCTTGAAGACGAAAGGGCCTCGTGATACGCCTATTTTTATAGGTTAATGTCATGATAATAATGGTTTCTTAGACGTCAGGT
GGCACTTTTCGGGGAAATGTGCGCGGAACCCCTATTTGTTTATTTTTCTAAATACATTCAAATATGTATCCGCTCATGAGACAATAACCCTGAT
AAATGCTTCAATAATATTGAAAAAGGAAGAGTATGAGTATTCAACATTTCCGTGTCGCCCTTATTCCCTTTTTTGCGGCATTTTGCCTTCCTGT
TTTTGCTCACCCAGAAACGCTGGTGAAAGTAAAAGATGCTGAAGATCAGTTGGGTGCACGAGTGGGTTACATCGAACTGGATCTCAACAGCGGT
AAGATCCTTGAGAGTTTTCGCCCCGAAGAACGTTTTCCAATGATGAGCACTTTTAAAGTTCTGCTATGTGGCGCGGTATTATCCCGTGTTGACG
CCGGGCAAGAGCAACTCGGTCGCCGCATACACTATTCTCAGAATGACTTGGTTGAGTACTCACCAGTCACAGAAAAGCATCTTACGGATGGCAT
GACAGTAAGAGAATTATGCAGTGCTGCCATAACCATGAGTGATAACACTGCGGCCAACTTACTTCTGACAACGATCGGAGGACCGAAGGAGCTA
ACCGCTTTTTTGCACAACATGGGGGATCATGTAACTCGCCTTGATCGTTGGGAACCGGAGCTGAATGAAGCCATACCAAACGACGAGCGTGACA
CCACGATGCCTGCAGCAATGGCAACAACGTTGCGCAAACTATTAACTGGCGAACTACTTACTCTAGCTTCCCGGCAACAATTAATAGACTGGAT
GGAGGCGGATAAAGTTGCAGGACCACTTCTGCGCTCGGCCCTTCCGGCTGGCTGGTTTATTGCTGATAAATCTGGAGCCGGTGAGCGTGGGTCT
CGCGGTATCATTGCAGCACTGGGGCCAGATGGTAAGCCCTCCCGTATCGTAGTTATCTACACGACGGGGAGTCAGGCAACTATGGATGAACGAA
ATAGACAGATCGCTGAGATAGGTGCCTCACTGATTAAGCATTGGTAACTGTCAGACCAAGTTTACTCATATATACTTTAGATTGATTTAAAACT
TCATTTTTAATTTAAAAGGATCTAGGTGAAGATCCTTTTTGATAATCTCATGACCAAAATCCCTTAACGTGAGTTTTCGTTCCACTGAGCGTCA
GACCCCGTAGAAAAGATCAAAGGATCTTCTTGAGATCCTTTTTTTCTGCGCGTAATCTGCTGCTTGCAAACAAAAAAACCACCGCTACCAGCGG
TGGTTTGTTTGCCGGATCAAGAGCTACCAACTCTTTTTCCGAAGGTAACTGGCTTCAGCAGAGCGCAGATACCAAATACTGTCCTTCTAGTGTA
GCCGTAGTTAGGCCACCACTTCAAGAACTCTGTAGCACCGCCTACATACCTCGCTCTGCTAATCCTGTTACCAGTGGCTGCTGCCAGTGGCGAT
AAGTCGTGTCTTACCGGGTTGGACTCAAGACGATAGTTACCGGATAAGGCGCAGCGGTCGGGCTGAACGGGGGGTTCGTGCACACAGCCCAGCT
TGGAGCGAACGACCTACACCGAACTGAGATACCTACAGCGTGAGCTATGAGAAAGCGCCACGCTTCCCGAAGGGAGAAAGGCGGACAGGTATCC
GGTAAGCGGCAGGGTCGGAACAGGAGAGCGCACGAGGGAGCTTCCAGGGGGAAACGCCTGGTATCTTTATAGTCCTGTCGGGTTTCGCCACCTC
TGACTTGAGCGTCGATTTTTGTGATGCTCGTCAGGGGGGCGGAGCCTATGGAAAAACGCCAGCAACGCGGCCTTTTTACGGTTCCTGGCCTTTT
GCTGGCCTTTTGCTCACATGTTCTTTCCTGCGTTATCCCCTGATTCTGTGGATAACCGTATTACCGCCTTTGAGTGAGCTGATACCGCTCGCCG
CAGCCGAACGACCGAGCGCAGCGAGTCAGTGAGCGAGGAAGCGGAAGAGCGCCTGATGCGGTATTTTCTCCTTACGCATCTGTGCGGTATTTCA
CACCGCAATGGTGCACTCTCAGTACAATCTGCTCTGATGCCGCATAGTTAAGCCAGTATACACTCCGCTATCGCTACGTGACTGGGTCATGGCT
GCGCCCCGACACCCGCCAACACCCGCTGACGCGCCCTGACGGGCTTGTCTGCTCCCGGCATCCGCTTACAGACAAGCTGTGACCGTCTCCGGGA
GCTGCATGTGTCAGAGGTTTTCACCGTCATCACCGAAACGCGCGAGGCAGCTGCGGTAAAGCTCATCAGCGTGGTCGTGAAGCGATTCACAGAT
GTCTGCCTGTTCATCCGCGTCCAGCTCGTTGAGTTTCTCCAGAAGCGTTAATGTCTGGCTTCTGATAAAGCGGGCCATGTTAAGGGCGGTTTTT
TCCTGTTTGGTCACTGATGCCTCCGTGTAAGGGGGATTTCTGTTCATGGGGGTAATGATACCGATGAAACGAGAGAGGATGCTCACGATACGGG
TTACTGATGATGAACATGCCCGGTTACTGGAACGTTGTGAGGGTAAACAACTGGCGGTATGGATGCGGCGGGACCAGAGAAAAATCACTCAGGG
TCAATGCCAGCGCTTCGTTAATACAGATGTAGGTGTTCCACAGGGTAGCCAGCAGCATCCTGCGATGCAGATCCGGAACATAATGGTGCAGGGC
GCTGACTTCCGCGTTTCCAGACTTTACGAAACACGGAAACCGAAGACCATTCATGTTGTTGCTCAGGTCGCAGACGTTTTGCAGCAGCAGTCGC
TTCACGTTCGCTCGCGTATCGGTGATTCATTCTGCTAACCAGTAAGGCAACCCCGCCAGCCTAGCCGGGTCCTCAACGACAGGAGCACGATCAT
GCGCACCCGTGGCCAGGACCCAACGCTGCCCGAGATGCGCCGCGTGCGGCTGCTGGAGATGGCGGACGCGATGGATATGTTCTGCCAAGGGTTG
GTTTGCGCATTCACAGTTCTCCGCAAGAATTGATTGGCTCCAATTCTTGGAGTGGTGAATCCGTTAGCGAGGTGCCGCCGGCTTCCATTCAGGT
CGAGGTGGCCCGGCTCCATGCACCGCGACGCAACGCGGGGAGGCAGACAAGGTATAGGGCGGCGCCTACAATCCATGCCAACCCGTTCCATGTG
CTCGCCGAGGCGGCATAAATCGCCGTGACGATCAGCGGTCCAATGATCGAAGTTAGGCTGGTAAGAGCCGCGAGCGATCCTTGAAGCTGTCCCT
GATGGTCGTCATCTACCTGCCTGGACAGCATGGCCTGCAACGCGGGCATCCCGATGCCGCCGGAAGCGAGAAGAATCATAATGGGGAAGGCCAT
CCAGCCTCGCGTCGCGAACGCCAGCAAGACGTAGCCCAGCGCGTCGGCCGCCATGCCGGCGATAATGGCCTGCTTCTCGCCGAAACGTTTGGTG
GCGGGACCAGTGACGAAGGCTTGAGCGAGGGCGTGCAAGATTCCGAATACCGCAAGCGACAGGCCGATCATCGTCGCGCTCCAGCGAAAGCGGT
CCTCGCCGAAAATGACCCAGAGCGCTGCCGGCACCTGTCCTACGAGTTGCATGATAAAGAAGACAGTCATAAGTGCGGCGACGATAGTCATGCC
CCGCGCCCACCGGAAGGAGCTGACTGGGTTGAAGGCTCTCAAGGGCATCGGTCGAGATCCCGGTGCCTAATGAGTGAGCTAACTTACATTAATT
GCGTTGCGCTCACTGCCCGCTTTCCAGTCGGGAAACCTGTCGTGCCAGCTGCATTAATGAATCGGCCAACGCGCGGGGAGAGGCGGTTTGCGTA
TTGGGCGCCAGGGTGGTTTTTCTTTTCACCAGTGAGACGGGCAACAGCTGATTGCCCTTCACCGCCTGGCCCTGAGAGAGTTGCAGCAAGCGGT
CCACGCTGGTTTGCCCCAGCAGGCGAAAATCCTGTTTGATGGTGGTTAACGGCGGGATATAACATGAGCTGTCTTCGGTATCGTCGTATCCCAC
TACCGAGATATCCGCACCAACGCGCAGCCCGGACTCGGTAATGGCGCGCATTGCGCCCAGCGCCATCTGATCGTTGGCAACCAGCATCGCAGTG
GGAACGATGCCCTCATTCAGCATTTGCATGGTTTGTTGAAAACCGGACATGGCACTCCAGTCGCCTTCCCGTTCCGCTATCGGCTGAATTTGAT
TGCGAGTGAGATATTTATGCCAGCCAGCCAGACGCAGACGCGCCGAGACAGAACTTAATGGGCCCGCTAACAGCGCGATTTGCTGGTGACCCAA
TGCGACCAGATGCTCCACGCCCAGTCGCGTACCGTCTTCATGGGAGAAAATAATACTGTTGATGGGTGTCTGGTCAGAGACATCAAGAAATAAC
GCCGGAACATTAGTGCAGGCAGCTTCCACAGCAATGGCATCCTGGTCATCCAGCGGATAGTTAATGATCAGCCCACTGACGCGTTGCGCGAGAA
GATTGTGCACCGCCGCTTTACAGGCTTCGACGCCGCTTCGTTCTACCATCGACACCACCACGCTGGCACCCAGTTGATCGGCGCGAGATTTAAT
CGCCGCGACAATTTGCGACGGCGCGTGCAGGGCCAGACTGGAGGTGGCAACGCCAATCAGCAACGACTGTTTGCCCGCCAGTTGTTGTGCCACG
CGGTTGGGAATGTAATTCAGCTCCGCCATCGCCGCTTCCACTTTTTCCCGCGTTTTCGCAGAAACGTGGCTGGCCTGGTTCACCACGCGGGAAA
CGGTCTGATAAGAGACACCGGCATACTCTGCGACATCGTATAACGTTACTGGTTTCACATTCACCACCCTGAATTGACTCTCTTCCGGGCGCTA
TCATGCCATACCGCGAAAGGTTTTGCGCCATTCGATGGTGTCCGGGATCTCGACGCTCTCCCTTATGCGACTCCTGCATTAGGAAGCAGCCCAG
TAGTAGGTTGAGGCCGTTGAGCACCGCCGCCGCAAGGAATGGTGCATGCAAGGAGATGGCGCCCAACAGTCCCCCGGCCACGGGGCCTGCCACC
ATACCCACGCCGAAACAAGCGCTCATGAGCCCGAAGTGGCGAGCCCGATCTTCCCCATCGGTGATGTCGGCGATATAGGCGCCAGCAACCGCAC
CTGTGGCGCCGGTGATGCCGGCCACGATGCGTCCGGCGTAGAGGATCGA

pET-28a(+)_GlmS_C2A

CCAGCTTGGAGCGAACGACCTACACCGAACTGAGATACCTACAGCGTGAGCTATGAGAAAGCGCCACGCTTCCCGAAGGGAGAAAGGCGGACAG
GTATCCGGTAAGCGGCAGGGTCGGAACAGGAGAGCGCACGAGGGAGCTTCCAGGGGGAAACGCCTGGTATCTTTATAGTCCTGTCGGGTTTCGC
CACCTCTGACTTGAGCGTCGATTTTTGTGATGCTCGTCAGGGGGGCGGAGCCTATGGAAAAACGCCAGCAACGCGGCCTTTTTACGGTTCCTGG
CCTTTTGCTGGCCTTTTGCTCACATGTTCTTTCCTGCGTTATCCCCTGATTCTGTGGATAACCGTATTACCGCCTTTGAGTGAGCTGATACCGC
TCGCCGCAGCCGAACGACCGAGCGCAGCGAGTCAGTGAGCGAGGAAGCGGAAGAGCGCCTGATGCGGTATTTTCTCCTTACGCATCTGTGCGGT
ATTTCACACCGCAATGGTGCACTCTCAGTACAATCTGCTCTGATGCCGCATAGTTAAGCCAGTATACACTCCGCTATCGCTACGTGACTGGGTC
ATGGCTGCGCCCCGACACCCGCCAACACCCGCTGACGCGCCCTGACGGGCTTGTCTGCTCCCGGCATCCGCTTACAGACAAGCTGTGACCGTCT
CCGGGAGCTGCATGTGTCAGAGGTTTTCACCGTCATCACCGAAACGCGCGAGGCAGCTGCGGTAAAGCTCATCAGCGTGGTCGTGAAGCGATTC
ACAGATGTCTGCCTGTTCATCCGCGTCCAGCTCGTTGAGTTTCTCCAGAAGCGTTAATGTCTGGCTTCTGATAAAGCGGGCCATGTTAAGGGCG
GTTTTTTCCTGTTTGGTCACTGATGCCTCCGTGTAAGGGGGATTTCTGTTCATGGGGGTAATGATACCGATGAAACGAGAGAGGATGCTCACGA
TACGGGTTACTGATGATGAACATGCCCGGTTACTGGAACGTTGTGAGGGTAAACAACTGGCGGTATGGATGCGGCGGGACCAGAGAAAAATCAC
TCAGGGTCAATGCCAGCGCTTCGTTAATACAGATGTAGGTGTTCCACAGGGTAGCCAGCAGCATCCTGCGATGCAGATCCGGAACATAATGGTG
CAGGGCGCTGACTTCCGCGTTTCCAGACTTTACGAAACACGGAAACCGAAGACCATTCATGTTGTTGCTCAGGTCGCAGACGTTTTGCAGCAGC
AGTCGCTTCACGTTCGCTCGCGTATCGGTGATTCATTCTGCTAACCAGTAAGGCAACCCCGCCAGCCTAGCCGGGTCCTCAACGACAGGAGCAC
GATCATGCGCACCCGTGGGGCCGCCATGCCGGCGATAATGGCCTGCTTCTCGCCGAAACGTTTGGTGGCGGGACCAGTGACGAAGGCTTGAGCG

AGGGCGTGCAAGATTCCGAATACCGCAAGCGACAGGCCGATCATCGTCGCGCTCCAGCGAAAGCGGTCCTCGCCGAAAATGACCCAGAGCGCTG
CCGGCACCTGTCCTACGAGTTGCATGATAAAGAAGACAGTCATAAGTGCGGCGACGATAGTCATGCCCCGCGCCCACCGGAAGGAGCTGACTGG
GTTGAAGGCTCTCAAGGGCATCGGTCGAGATCCCGGTGCCTAATGAGTGAGCTAACTTACATTAATTGCGTTGCGCTCACTGCCCGCTTTCCAG
TCGGGAAACCTGTCGTGCCAGCTGCATTAATGAATCGGCCAACGCGCGGGGAGAGGCGGTTTGCGTATTGGGCGCCAGGGTGGTTTTTCTTTTC
ACCAGTGAGACGGGCAACAGCTGATTGCCCTTCACCGCCTGGCCCTGAGAGAGTTGCAGCAAGCGGTCCACGCTGGTTTGCCCCAGCAGGCGAA
AATCCTGTTTGATGGTGGTTAACGGCGGGATATAACATGAGCTGTCTTCGGTATCGTCGTATCCCACTACCGAGATATCCGCACCAACGCGCAG
CCCGGACTCGGTAATGGCGCGCATTGCGCCCAGCGCCATCTGATCGTTGGCAACCAGCATCGCAGTGGGAACGATGCCCTCATTCAGCATTTGC
ATGGTTTGTTGAAAACCGGACATGGCACTCCAGTCGCCTTCCCGTTCCGCTATCGGCTGAATTTGATTGCGAGTGAGATATTTATGCCAGCCAG
CCAGACGCAGACGCGCCGAGACAGAACTTAATGGGCCCGCTAACAGCGCGATTTGCTGGTGACCCAATGCGACCAGATGCTCCACGCCCAGTCG
CGTACCGTCTTCATGGGAGAAAATAATACTGTTGATGGGTGTCTGGTCAGAGACATCAAGAAATAACGCCGGAACATTAGTGCAGGCAGCTTCC
ACAGCAATGGCATCCTGGTCATCCAGCGGATAGTTAATGATCAGCCCACTGACGCGTTGCGCGAGAAGATTGTGCACCGCCGCTTTACAGGCTT
CGACGCCGCTTCGTTCTACCATCGACACCACCACGCTGGCACCCAGTTGATCGGCGCGAGATTTAATCGCCGCGACAATTTGCGACGGCGCGTG
CAGGGCCAGACTGGAGGTGGCAACGCCAATCAGCAACGACTGTTTGCCCGCCAGTTGTTGTGCCACGCGGTTGGGAATGTAATTCAGCTCCGCC
ATCGCCGCTTCCACTTTTTCCCGCGTTTTCGCAGAAACGTGGCTGGCCTGGTTCACCACGCGGGAAACGGTCTGATAAGAGACACCGGCATACT
CTGCGACATCGTATAACGTTACTGGTTTCACATTCACCACCCTGAATTGACTCTCTTCCGGGCGCTATCATGCCATACCGCGAAAGGTTTTGCG
CCATTCGATGGTGTCCGGGATCTCGACGCTCTCCCTTATGCGACTCCTGCATTAGGAAGCAGCCCAGTAGTAGGTTGAGGCCGTTGAGCACCGC
CGCCGCAAGGAATGGTGCATGCAAGGAGATGGCGCCCAACAGTCCCCCGGCCACGGGGCCTGCCACCATACCCACGCCGAAACAAGCGCTCATG
AGCCCGAAGTGGCGAGCCCGATCTTCCCCATCGGTGATGTCGGCGATATAGGCGCCAGCAACCGCACCTGTGGCGCCGGTGATGCCGGCCACGA
TGCGTCCGGCGTAGAGGATCGAGATCTCGATCCCGCGAAATTAATACGACTCACTATAGGGGAATTGTGAGCGGATAACAATTCCCCTCTAGAA
ATAATTTTGTTTAACTTTAAGAAGGAGATATACCATGGCAGGTATTGTAGGCTACATTGGCTATGATAATGCAAAAGAATTATTGCTGAAAGGC
TTGGAAAAGCTGGAGTATCGTGGGTATGATTCCGCTGGGATTGCTGTCGTGAATGACGATAATACGACCGTGTTTAAGGAGAAAGGGCGCATTG
CTGAACTGCGGAAAGTCGCTGATTCGTCCGATTTCGATGGGCCGGTAGGCATAGGCCACACTCGTTGGGCGACACATGGAGTTCCGAATCATGA
GAACTCTCACCCCCATCAGTCATCTAATGGGAGATTTACACTGGTGCATAACGGTGTTATAGAAAATTACGAAGAACTGAAAGGTGAGTATTTA
CAGGGCGTGTCCTTTATCAGTGAAACAGATACCGAGGTCATTGTGCAGCTGGTTGAGTACTTCTCTAATCAGGGCTTGAGCACAGAAGAAGCAT
TTACGAAAGTTGTTTCTCTGCTGCACGGCAGCTACGCGCTGGGCCTGCTGGATGCAGAAGATAAAGATACGATTTATGTGGCGAAAAATAAAAG
TCCCTTACTGCTCGGTGTGGGTGAAGGTTTCAACGTTATAGCTAGCGACGCGCTTGCCATGTTACAGGTTACCAGCGAATATAAAGAAATCCAT
GACCACGAAATTGTGATAGTTAAGAAAGATGAGGTCATTATAAAAGATGCAGATGGCAACGTAGTTGAGAGAGATAGCTACATCGCCGAAATCG
ATGCTAGCGATGCTGAAAAAGGTGTGTACGCACATTATATGTTGAAAGAAATACATGAACAGCCGGCTGTCATGCGTCGCATTATTCAGGAGTA
TCAAGACGCAGAAGGCAATTTAAAAATAGACCAAGACATTATCAACGACGTCAAAGAGGCGGATCGGATTTACGTGATCGCTGCTGGCACTTCG
TATCACGCTGGATTGGTTGGGAAAGAATTCCTGGAAAAATGGGCTGGGGTGCCAACAGAAGTGCATGTGGCATCCGAATTCGTCTATAACATGC
CGCTGTTGTCAGAAAAACCCCTCTTCGTTTATATATCCCAGAGCGGTGAAACCGCGGATTCCCGCGCGGTGCTGGTAGAAACTAACAAATTAGG
CCATAAAAGTTTAACGATTACAAATGTTGCGGGCTCAACCTTATCGCGGGAAGCAGACCACACGCTGCTGCTTCATGCTGGCCCTGAAATCGCC
GTCGCGTCTACAAAAGCTTATACCGCCCAGATCGCAGTCCTGTCCATTCTTAGTCAAATTGTAGCGAAGGAACATGGGCGAGAAGCCGATATCG
ACTTACTGCGCGAGTTAGCAAAAGTCACCACAGCGATAGAAGCTATTGTGGACGATGCTCCAATTATGGAACAGATAGCCACGGATTTTCTGGA
AACCACAAGAAATGCATTTTTTATAGGGCGTACGATAGATTATAACGTCTCTTTAGAAGGAGCCCTCAAACTGAAAGAAATCAGCTACATTCAA
GCAGAGGGTTTCGCAGGAGGTGAACTGAAACATGGCACGATCGCCTTGATCGAAGAGGGCACGCCCGTAGTAGGATTGGCCACACAAGAGAAGG
TGAATTTATCGATTCGGGGAAATGTCAAAGAAGTGGTGGCACGTGGTGCCCACCCGTGCATCATTTCAATGGAGGGCCTGGAAAAGGAAGGCGA
TACGTACGTTATACCACACGTACACGAGCTGCTGACACCTCTGGTATCGGTGGTAGCTCTTCAGCTGATCAGTTATTATGCCGCTCTGCACCGC
GATCTCGATGTAGATAAACCTCGTAATCTGGCGAAGAGCGTGACCGTCGAAGAAAACCTGTACTTTCAGGGCCTCGAGCACCACCACCACCACC
ACTGAGATCCGGCTGCTAACAAAGCCCGAAAGGAAGCTGAGTTGGCTGCTGCCACCGCTGAGCAATAACTAGCATAACCCCTTGGGGCCTCTAA
ACGGGTCTTGAGGGGTTTTTTGCTGAAAGGAGGAACTATATCCGGATTGGCGAATGGGACGCGCCCTGTAGCGGCGCATTAAGCGCGGCGGGTG
TGGTGGTTACGCGCAGCGTGACCGCTACACTTGCCAGCGCCCTAGCGCCCGCTCCTTTCGCTTTCTTCCCTTCCTTTCTCGCCACGTTCGCCGG
CTTTCCCCGTCAAGCTCTAAATCGGGGGCTCCCTTTAGGGTTCCGATTTAGTGCTTTACGGCACCTCGACCCCAAAAAACTTGATTAGGGTGAT
GGTTCACGTAGTGGGCCATCGCCCTGATAGACGGTTTTTCGCCCTTTGACGTTGGAGTCCACGTTCTTTAATAGTGGACTCTTGTTCCAAACTG
GAACAACACTCAACCCTATCTCGGTCTATTCTTTTGATTTATAAGGGATTTTGCCGATTTCGGCCTATTGGTTAAAAAATGAGCTGATTTAACA
AAAATTTAACGCGAATTTTAACAAAATATTAACGCTTACAATTTAGGTGGCACTTTTCGGGGAAATGTGCGCGGAACCCCTATTTGTTTATTTT
TCTAAATACATTCAAATATGTATCCGCTCATGAATTAATTCTTAGAAAAACTCATCGAGCATCAAATGAAACTGCAATTTATTCATATCAGGAT
TATCAATACCATATTTTTGAAAAAGCCGTTTCTGTAATGAAGGAGAAAACTCACCGAGGCAGTTCCATAGGATGGCAAGATCCTGGTATCGGTC
TGCGATTCCGACTCGTCCAACATCAATACAACCTATTAATTTCCCCTCGTCAAAAATAAGGTTATCAAGTGAGAAATCACCATGAGTGACGACT
GAATCCGGTGAGAATGGCAAAAGTTTATGCATTTCTTTCCAGACTTGTTCAACAGGCCAGCCATTACGCTCGTCATCAAAATCACTCGCATCAA
CCAAACCGTTATTCATTCGTGATTGCGCCTGAGCGAGACGAAATACGCGATCGCTGTTAAAAGGACAATTACAAACAGGAATCGAATGCAACCG
GCGCAGGAACACTGCCAGCGCATCAACAATATTTTCACCTGAATCAGGATATTCTTCTAATACCTGGAATGCTGTTTTCCCGGGGATCGCAGTG
GTGAGTAACCATGCATCATCAGGAGTACGGATAAAATGCTTGATGGTCGGAAGAGGCATAAATTCCGTCAGCCAGTTTAGTCTGACCATCTCAT
CTGTAACATCATTGGCAACGCTACCTTTGCCATGTTTCAGAAACAACTCTGGCGCATCGGGCTTCCCATACAATCGATAGATTGTCGCACCTGA
TTGCCCGACATTATCGCGAGCCCATTTATACCCATATAAATCAGCATCCATGTTGGAATTTAATCGCGGCCTAGAGCAAGACGTTTCCCGTTGA
ATATGGCTCATAACACCCCTTGTATTACTGTTTATGTAAGCAGACAGTTTTATTGTTCATGACCAAAATCCCTTAACGTGAGTTTTCGTTCCAC
TGAGCGTCAGACCCCGTAGAAAAGATCAAAGGATCTTCTTGAGATCCTTTTTTTCTGCGCGTAATCTGCTGCTTGCAAACAAAAAAACCACCGC
TACCAGCGGTGGTTTGTTTGCCGGATCAAGAGCTACCAACTCTTTTTCCGAAGGTAACTGGCTTCAGCAGAGCGCAGATACCAAATACTGTCCT
TCTAGTGTAGCCGTAGTTAGGCCACCACTTCAAGAACTCTGTAGCACCGCCTACATACCTCGCTCTGCTAATCCTGTTACCAGTGGCTGCTGCC
AGTGGCGATAAGTCGTGTCTTACCGGGTTGGACTCAAGACGATAGTTACCGGATAAGGCGCAGCGGTCGGGCTGAACGGGGGGTTCGTGCACAC
AGC

pET300/NT-Dest_FabF

GATCTCGATCCCGCGAAATTAATACGACTCACTATAGGGGAATTGTGAGCGGATAACAATTCCCCTCTAGAAATAATTTTGTTTAACTTTAAG
AAGGAGATATACATATGCATCATCATCATCATCACATCACAAGTTTGTACAAAAAAGCAGGCTTTGAGAATCTTTATTTTCAGGGCATGAGTCA
AAATAAAAGAGTAGTTATTACAGGTATGGGAGCCCTTTCTCCAATCGGTAATGATGTCAAAACAACATGGGAGAATGCTCTAAAAGGCGTAAAT
GGTATCGATAAAATTACACGTATCGATACTGAACCTTATAGCGTTCACTTAGCAGGAGAACTTAAAAACTTTAATATTGAAGATCATATCGACA
AAAAAGAAGCGCGTCGTATGGATAGATTTACTCAATATGCAATTGTAGCAGCTAGAGAGGCTGTTAAAGATGCGCAATTAGATATCAATGAAAA
TACTGCAGATCGAATCGGTGTATGGATTGGTTCTGGTATCGGTGGTATGGAAACATTTGAAATTGCACATAAACAATTAATGGATAAAGGCCCA
AGACGTGTGAGTCCATTTTTCGTACCAATGTTAATTCCTGATATGGCAACTGGGCAAGTATCAATTGACTTAGGTGCAAAAGGACCAAATGGTG
CAACAGTTACAGCATGTGCAACAGGTACAAATTCAATCGGAGAAGCATTTAAAATTGTGCAACGCGGTGATGCAGATGCAATGATTACTGGTGG
TACAGAAGCACCAATTACTCATATGGCAATTGCTGGTTTCAGTGCAAGTCGAGCGCTTTCTACAAATGATGACATTGAAACAGCATGTCGTCCA
TTCCAAGAAGGTAGAGATGGTTTTGTTATGGGTGAAGGTGCTGGTATTTTAGTAATTGAATCTTTAGAATCAGCACAAGCTCGAGGTGCCAATA
TTTATGCTGAGATAGTTGGCTATGGTACTACAGGTGATGCTTATCATATTACAGCGCCAGCTCCAGAAGGTGAAGGTGGTTCTAGAGCAATGCA
AGCAGCTATGGATGATGCTGGTATTGAACCTAAAGATGTACAATACTTAAATGCCCATGGTACAAGTACTCCTGTTGGTGACTTAAATGAAGTT
AAAGCTATTAAAAATACATTTGGTGAAGCAGCTAAACACTTAAAAGTTAGCTCAACAAAATCAATGACTGGTCACTTACTTGGTGCAACAGGTG
GAATTGAAGCAATCTTCTCAGCGCTTTCAATTAAAGACTCTAAAGTCGCACCGACAATTCATGCGGTAACACCAGATCCAGAATGTGATTTGGA

TATTGTTCCAAATGAAGCGCAAGACCTTGATATTACTTATGCAATGAGTAATAGCTTAGGATTCGGTGGACATAACGCAGTATTAGTATTCAAG
AAATTTGAAGCATAACCACCCAGCTTTCTTGTACAAAGTGGTGATCTAGGTATAATCGGATCCGGCTGCTAACAAAGCCCGAAAGGAAGCTGAG
TTGGCTGCTGCCACCGCTGAGCAATAACTAGCATAACCCCTTGGGGCCTCTAAACGGGTCTTGAGGGGTTTTTTGCTGAAAGGAGGAACTATAT
CCGGATATCCCGCAAGAGGCCCGGCAGTACCGGCATAACCAAGCCTATGCCTACAGCATCCAGGGTGACGGTGCCGAGGATGACGATGAGCGCA
TTGTTAGATTTCATACACGGTGCCTGACTGCGTTAGCAATTTAACTGTGATAAACTACCGCATTAAAGCTAGCTTATCGATGATAAGCTGTCAA
ACATGAGAATTAATTCTTGAAGACGAAAGGGCCTCGTGATACGCCTATTTTTATAGGTTAATGTCATGATAATAATGGTTTCTTAGACGTCAGG
TGGCACTTTTCGGGGAAATGTGCGCGGAACCCCTATTTGTTTATTTTTCTAAATACATTCAAATATGTATCCGCTCATGAGACAATAACCCTGA
TAAATGCTTCAATAATATTGAAAAAGGAAGAGTATGAGTATTCAACATTTCCGTGTCGCCCTTATTCCCTTTTTTGCGGCATTTTGCCTTCCTG
TTTTTGCTCACCCAGAAACGCTGGTGAAAGTAAAAGATGCTGAAGATCAGTTGGGTGCACGAGTGGGTTACATCGAACTGGATCTCAACAGCGG
TAAGATCCTTGAGAGTTTTCGCCCCGAAGAACGTTTTCCAATGATGAGCACTTTTAAAGTTCTGCTATGTGGCGCGGTATTATCCCGTGTTGAC
GCCGGGCAAGAGCAACTCGGTCGCCGCATACACTATTCTCAGAATGACTTGGTTGAGTACTCACCAGTCACAGAAAAGCATCTTACGGATGGCA
TGACAGTAAGAGAATTATGCAGTGCTGCCATAACCATGAGTGATAACACTGCGGCCAACTTACTTCTGACAACGATCGGAGGACCGAAGGAGCT
AACCGCTTTTTTGCACAACATGGGGGATCATGTAACTCGCCTTGATCGTTGGGAACCGGAGCTGAATGAAGCCATACCAAACGACGAGCGTGAC
ACCACGATGCCTGCAGCAATGGCAACAACGTTGCGCAAACTATTAACTGGCGAACTACTTACTCTAGCTTCCCGGCAACAATTAATAGACTGGA
TGGAGGCGGATAAAGTTGCAGGACCACTTCTGCGCTCGGCCCTTCCGGCTGGCTGGTTTATTGCTGATAAATCTGGAGCCGGTGAGCGTGGGTC
TCGCGGTATCATTGCAGCACTGGGGCCAGATGGTAAGCCCTCCCGTATCGTAGTTATCTACACGACGGGGAGTCAGGCAACTATGGATGAACGA
AATAGACAGATCGCTGAGATAGGTGCCTCACTGATTAAGCATTGGTAACTGTCAGACCAAGTTTACTCATATATACTTTAGATTGATTTAAAAC
TTCATTTTTAATTTAAAAGGATCTAGGTGAAGATCCTTTTTGATAATCTCATGACCAAAATCCCTTAACGTGAGTTTTCGTTCCACTGAGCGTC
AGACCCCGTAGAAAAGATCAAAGGATCTTCTTGAGATCCTTTTTTTCTGCGCGTAATCTGCTGCTTGCAAACAAAAAAACCACCGCTACCAGCG
GTGGTTTGTTTGCCGGATCAAGAGCTACCAACTCTTTTTCCGAAGGTAACTGGCTTCAGCAGAGCGCAGATACCAAATACTGTCCTTCTAGTGT
AGCCGTAGTTAGGCCACCACTTCAAGAACTCTGTAGCACCGCCTACATACCTCGCTCTGCTAATCCTGTTACCAGTGGCTGCTGCCAGTGGCGA
TAAGTCGTGTCTTACCGGGTTGGACTCAAGACGATAGTTACCGGATAAGGCGCAGCGGTCGGGCTGAACGGGGGGTTCGTGCACACAGCCCAGC
TTGGAGCGAACGACCTACACCGAACTGAGATACCTACAGCGTGAGCTATGAGAAAGCGCCACGCTTCCCGAAGGGAGAAAGGCGGACAGGTATC
CGGTAAGCGGCAGGGTCGGAACAGGAGAGCGCACGAGGGAGCTTCCAGGGGGAAACGCCTGGTATCTTTATAGTCCTGTCGGGTTTCGCCACCT
CTGACTTGAGCGTCGATTTTTGTGATGCTCGTCAGGGGGGCGGAGCCTATGGAAAAACGCCAGCAACGCGGCCTTTTTACGGTTCCTGGCCTTT
TGCTGGCCTTTTGCTCACATGTTCTTTCCTGCGTTATCCCCTGATTCTGTGGATAACCGTATTACCGCCTTTGAGTGAGCTGATACCGCTCGCC
GCAGCCGAACGACCGAGCGCAGCGAGTCAGTGAGCGAGGAAGCGGAAGAGCGCCTGATGCGGTATTTTCTCCTTACGCATCTGTGCGGTATTTC
ACACCGCAATGGTGCACTCTCAGTACAATCTGCTCTGATGCCGCATAGTTAAGCCAGTATACACTCCGCTATCGCTACGTGACTGGGTCATGGC
TGCGCCCCGACACCCGCCAACACCCGCTGACGCGCCCTGACGGGCTTGTCTGCTCCCGGCATCCGCTTACAGACAAGCTGTGACCGTCTCCGGG
AGCTGCATGTGTCAGAGGTTTTCACCGTCATCACCGAAACGCGCGAGGCAGCTGCGGTAAAGCTCATCAGCGTGGTCGTGAAGCGATTCACAGA
TGTCTGCCTGTTCATCCGCGTCCAGCTCGTTGAGTTTCTCCAGAAGCGTTAATGTCTGGCTTCTGATAAAGCGGGCCATGTTAAGGGCGGTTTT
TTCCTGTTTGGTCACTGATGCCTCCGTGTAAGGGGGATTTCTGTTCATGGGGGTAATGATACCGATGAAACGAGAGAGGATGCTCACGATACGG
GTTACTGATGATGAACATGCCCGGTTACTGGAACGTTGTGAGGGTAAACAACTGGCGGTATGGATGCGGCGGGACCAGAGAAAAATCACTCAGG
GTCAATGCCAGCGCTTCGTTAATACAGATGTAGGTGTTCCACAGGGTAGCCAGCAGCATCCTGCGATGCAGATCCGGAACATAATGGTGCAGGG
CGCTGACTTCCGCGTTTCCAGACTTTACGAAACACGGAAACCGAAGACCATTCATGTTGTTGCTCAGGTCGCAGACGTTTTGCAGCAGCAGTCG
CTTCACGTTCGCTCGCGTATCGGTGATTCATTCTGCTAACCAGTAAGGCAACCCCGCCAGCCTAGCCGGGTCCTCAACGACAGGAGCACGATCA
TGCGCACCCGTGGCCAGGACCCAACGCTGCCCGAGATGCGCCGCGTGCGGCTGCTGGAGATGGCGGACGCGATGGATATGTTCTGCCAAGGGTT
GGTTTGCGCATTCACAGTTCTCCGCAAGAATTGATTGGCTCCAATTCTTGGAGTGGTGAATCCGTTAGCGAGGTGCCGCCGGCTTCCATTCAGG
TCGAGGTGGCCCGGCTCCATGCACCGCGACGCAACGCGGGGAGGCAGACAAGGTATAGGGCGGCGCCTACAATCCATGCCAACCCGTTCCATGT
GCTCGCCGAGGCGGCATAAATCGCCGTGACGATCAGCGGTCCAATGATCGAAGTTAGGCTGGTAAGAGCCGCGAGCGATCCTTGAAGCTGTCCC
TGATGGTCGTCATCTACCTGCCTGGACAGCATGGCCTGCAACGCGGGCATCCCGATGCCGCCGGAAGCGAGAAGAATCATAATGGGGAAGGCCA
TCCAGCCTCGCGTCGCGAACGCCAGCAAGACGTAGCCCAGCGCGTCGGCCGCCATGCCGGCGATAATGGCCTGCTTCTCGCCGAAACGTTTGGT
GGCGGGACCAGTGACGAAGGCTTGAGCGAGGGCGTGCAAGATTCCGAATACCGCAAGCGACAGGCCGATCATCGTCGCGCTCCAGCGAAAGCGG
TCCTCGCCGAAAATGACCCAGAGCGCTGCCGGCACCTGTCCTACGAGTTGCATGATAAAGAAGACAGTCATAAGTGCGGCGACGATAGTCATGC
CCCGCGCCCACCGGAAGGAGCTGACTGGGTTGAAGGCTCTCAAGGGCATCGGTCGAGATCCCGGTGCCTAATGAGTGAGCTAACTTACATTAAT
TGCGTTGCGCTCACTGCCCGCTTTCCAGTCGGGAAACCTGTCGTGCCAGCTGCATTAATGAATCGGCCAACGCGCGGGGAGAGGCGGTTTGCGT
ATTGGGCGCCAGGGTGGTTTTTCTTTTCACCAGTGAGACGGGCAACAGCTGATTGCCCTTCACCGCCTGGCCCTGAGAGAGTTGCAGCAAGCGG
TCCACGCTGGTTTGCCCCAGCAGGCGAAAATCCTGTTTGATGGTGGTTAACGGCGGGATATAACATGAGCTGTCTTCGGTATCGTCGTATCCCA
CTACCGAGATATCCGCACCAACGCGCAGCCCGGACTCGGTAATGGCGCGCATTGCGCCCAGCGCCATCTGATCGTTGGCAACCAGCATCGCAGT
GGGAACGATGCCCTCATTCAGCATTTGCATGGTTTGTTGAAAACCGGACATGGCACTCCAGTCGCCTTCCCGTTCCGCTATCGGCTGAATTTGA
TTGCGAGTGAGATATTTATGCCAGCCAGCCAGACGCAGACGCGCCGAGACAGAACTTAATGGGCCCGCTAACAGCGCGATTTGCTGGTGACCCA
ATGCGACCAGATGCTCCACGCCCAGTCGCGTACCGTCTTCATGGGAGAAAATAATACTGTTGATGGGTGTCTGGTCAGAGACATCAAGAAATAA
CGCCGGAACATTAGTGCAGGCAGCTTCCACAGCAATGGCATCCTGGTCATCCAGCGGATAGTTAATGATCAGCCCACTGACGCGTTGCGCGAGA
AGATTGTGCACCGCCGCTTTACAGGCTTCGACGCCGCTTCGTTCTACCATCGACACCACCACGCTGGCACCCAGTTGATCGGCGCGAGATTTAA
TCGCCGCGACAATTTGCGACGGCGCGTGCAGGGCCAGACTGGAGGTGGCAACGCCAATCAGCAACGACTGTTTGCCCGCCAGTTGTTGTGCCAC
GCGGTTGGGAATGTAATTCAGCTCCGCCATCGCCGCTTCCACTTTTTCCCGCGTTTTCGCAGAAACGTGGCTGGCCTGGTTCACCACGCGGGAA
ACGGTCTGATAAGAGACACCGGCATACTCTGCGACATCGTATAACGTTACTGGTTTCACATTCACCACCCTGAATTGACTCTCTTCCGGGCGCT
ATCATGCCATACCGCGAAAGGTTTTGCGCCATTCGATGGTGTCCGGGATCTCGACGCTCTCCCTTATGCGACTCCTGCATTAGGAAGCAGCCCA
GTAGTAGGTTGAGGCCGTTGAGCACCGCCGCCGCAAGGAATGGTGCATGCAAGGAGATGGCGCCCAACAGTCCCCCGGCCACGGGGCCTGCCAC
CATACCCACGCCGAAACAAGCGCTCATGAGCCCGAAGTGGCGAGCCCGATCTTCCCCATCGGTGATGTCGGCGATATAGGCGCCAGCAACCGCA
CCTGTGGCGCCGGTGATGCCGGCCACGATGCGTCCGGCGTAGAGGATCGA

pET-28a(+)_FabF_C165A

CCAGCTTGGAGCGAACGACCTACACCGAACTGAGATACCTACAGCGTGAGCTATGAGAAAGCGCCACGCTTCCCGAAGGGAGAAAGGCGGACAG
GTATCCGGTAAGCGGCAGGGTCGGAACAGGAGAGCGCACGAGGGAGCTTCCAGGGGGAAACGCCTGGTATCTTTATAGTCCTGTCGGGTTTCGC
CACCTCTGACTTGAGCGTCGATTTTTGTGATGCTCGTCAGGGGGGCGGAGCCTATGGAAAAACGCCAGCAACGCGGCCTTTTTACGGTTCCTGG
CCTTTTGCTGGCCTTTTGCTCACATGTTCTTTCCTGCGTTATCCCCTGATTCTGTGGATAACCGTATTACCGCCTTTGAGTGAGCTGATACCGC
TCGCCGCAGCCGAACGACCGAGCGCAGCGAGTCAGTGAGCGAGGAAGCGGAAGAGCGCCTGATGCGGTATTTTCTCCTTACGCATCTGTGCGGT
ATTTCACACCGCAATGGTGCACTCTCAGTACAATCTGCTCTGATGCCGCATAGTTAAGCCAGTATACACTCCGCTATCGCTACGTGACTGGGTC
ATGGCTGCGCCCCGACACCCGCCAACACCCGCTGACGCGCCCTGACGGGCTTGTCTGCTCCCGGCATCCGCTTACAGACAAGCTGTGACCGTCT
CCGGGAGCTGCATGTGTCAGAGGTTTTCACCGTCATCACCGAAACGCGCGAGGCAGCTGCGGTAAAGCTCATCAGCGTGGTCGTGAAGCGATTC
ACAGATGTCTGCCTGTTCATCCGCGTCCAGCTCGTTGAGTTTCTCCAGAAGCGTTAATGTCTGGCTTCTGATAAAGCGGGCCATGTTAAGGGCG
GTTTTTTCCTGTTTGGTCACTGATGCCTCCGTGTAAGGGGGATTTCTGTTCATGGGGGTAATGATACCGATGAAACGAGAGAGGATGCTCACGA
TACGGGTTACTGATGATGAACATGCCCGGTTACTGGAACGTTGTGAGGGTAAACAACTGGCGGTATGGATGCGGCGGGACCAGAGAAAAATCAC
TCAGGGTCAATGCCAGCGCTTCGTTAATACAGATGTAGGTGTTCCACAGGGTAGCCAGCAGCATCCTGCGATGCAGATCCGGAACATAATGGTG
CAGGGCGCTGACTTCCGCGTTTCCAGACTTTACGAAACACGGAAACCGAAGACCATTCATGTTGTTGCTCAGGTCGCAGACGTTTTGCAGCAGC
AGTCGCTTCACGTTCGCTCGCGTATCGGTGATTCATTCTGCTAACCAGTAAGGCAACCCCGCCAGCCTAGCCGGGTCCTCAACGACAGGAGCAC

GATCATGCGCACCCGTGGGGCCGCCATGCCGGCGATAATGGCCTGCTTCTCGCCGAAACGTTTGGTGGCGGGACCAGTGACGAAGGCTTGAGCG
AGGGCGTGCAAGATTCCGAATACCGCAAGCGACAGGCCGATCATCGTCGCGCTCCAGCGAAAGCGGTCCTCGCCGAAAATGACCCAGAGCGCTG
CCGGCACCTGTCCTACGAGTTGCATGATAAAGAAGACAGTCATAAGTGCGGCGACGATAGTCATGCCCCGCGCCCACCGGAAGGAGCTGACTGG
GTTGAAGGCTCTCAAGGGCATCGGTCGAGATCCCGGTGCCTAATGAGTGAGCTAACTTACATTAATTGCGTTGCGCTCACTGCCCGCTTTCCAG
TCGGGAAACCTGTCGTGCCAGCTGCATTAATGAATCGGCCAACGCGCGGGGAGAGGCGGTTTGCGTATTGGGCGCCAGGGTGGTTTTTCTTTTC
ACCAGTGAGACGGGCAACAGCTGATTGCCCTTCACCGCCTGGCCCTGAGAGAGTTGCAGCAAGCGGTCCACGCTGGTTTGCCCCAGCAGGCGAA
AATCCTGTTTGATGGTGGTTAACGGCGGGATATAACATGAGCTGTCTTCGGTATCGTCGTATCCCACTACCGAGATATCCGCACCAACGCGCAG
CCCGGACTCGGTAATGGCGCGCATTGCGCCCAGCGCCATCTGATCGTTGGCAACCAGCATCGCAGTGGGAACGATGCCCTCATTCAGCATTTGC
ATGGTTTGTTGAAAACCGGACATGGCACTCCAGTCGCCTTCCCGTTCCGCTATCGGCTGAATTTGATTGCGAGTGAGATATTTATGCCAGCCAG
CCAGACGCAGACGCGCCGAGACAGAACTTAATGGGCCCGCTAACAGCGCGATTTGCTGGTGACCCAATGCGACCAGATGCTCCACGCCCAGTCG
CGTACCGTCTTCATGGGAGAAAATAATACTGTTGATGGGTGTCTGGTCAGAGACATCAAGAAATAACGCCGGAACATTAGTGCAGGCAGCTTCC
ACAGCAATGGCATCCTGGTCATCCAGCGGATAGTTAATGATCAGCCCACTGACGCGTTGCGCGAGAAGATTGTGCACCGCCGCTTTACAGGCTT
CGACGCCGCTTCGTTCTACCATCGACACCACCACGCTGGCACCCAGTTGATCGGCGCGAGATTTAATCGCCGCGACAATTTGCGACGGCGCGTG
CAGGGCCAGACTGGAGGTGGCAACGCCAATCAGCAACGACTGTTTGCCCGCCAGTTGTTGTGCCACGCGGTTGGGAATGTAATTCAGCTCCGCC
ATCGCCGCTTCCACTTTTTCCCGCGTTTTCGCAGAAACGTGGCTGGCCTGGTTCACCACGCGGGAAACGGTCTGATAAGAGACACCGGCATACT
CTGCGACATCGTATAACGTTACTGGTTTCACATTCACCACCCTGAATTGACTCTCTTCCGGGCGCTATCATGCCATACCGCGAAAGGTTTTGCG
CCATTCGATGGTGTCCGGGATCTCGACGCTCTCCCTTATGCGACTCCTGCATTAGGAAGCAGCCCAGTAGTAGGTTGAGGCCGTTGAGCACCGC
CGCCGCAAGGAATGGTGCATGCAAGGAGATGGCGCCCAACAGTCCCCCGGCCACGGGGCCTGCCACCATACCCACGCCGAAACAAGCGCTCATG
AGCCCGAAGTGGCGAGCCCGATCTTCCCCATCGGTGATGTCGGCGATATAGGCGCCAGCAACCGCACCTGTGGCGCCGGTGATGCCGGCCACGA
TGCGTCCGGCGTAGAGGATCGAGATCTCGATCCCGCGAAATTAATACGACTCACTATAGGGGAATTGTGAGCGGATAACAATTCCCCTCTAGAA
ATAATTTTGTTTAACTTTAAGAAGGAGATATACCATGGGCAGCAGCCATCATCATCATCATCACAGCAGCGGCCTGGTGCCGCGCGGCAGCCAT
ATGATGTCCCAAAACAAGAGAGTTGTTATCACAGGCATGGGTGCACTTTCCCCCATCGGCAATGACGTTAAAACGACTTGGGAGAACGCTCTGA
AGGGCGTCAACGGCATCGACAAAATCACTCGGATCGACACAGAGCCGTATAGTGTACATTTAGCCGGAGAGCTGAAGAACTTCAATATTGAGGA
CCATATCGATAAGAAGGAAGCCCGACGCATGGACCGTTTCACGCAGTACGCGATAGTTGCGGCTCGTGAGGCAGTTAAGGATGCCCAGCTGGAC
ATCAACGAGAACACGGCCGACCGCATCGGAGTGTGGATAGGCTCAGGCATTGGTGGCATGGAGACCTTCGAGATAGCACACAAGCAGCTGATGG
ACAAGGGCCCCCGTCGCGTAAGCCCATTCTTTGTGCCTATGTTAATTCCTGACATGGCCACAGGTCAAGTCAGTATCGATCTGGGAGCGAAGGG
CCCCAACGGGGCTACGGTCACGGCAGCTGCGACTGGAACAAATAGTATTGGAGAAGCCTTTAAAATCGTGCAGCGAGGCGACGCAGACGCCATG
ATCACGGGTGGGACCGAGGCTCCGATCACCCACATGGCGATAGCTGGTTTCTCTGCGTCACGGGCGTTATCTACCAACGATGACATCGAGACAG
CCTGTCGGCCCTTCCAAGAGGGTCGCGACGGTTTCGTGATGGGCGAGGGAGCAGGTATCCTGGTTATTGAATCCCTCGAATCCGCTCAGGCTCG
CGGCGCTAATATATACGCTGAAATCGTCGGGTACGGAACCACTGGAGACGCCTATCATATTACAGCGCCTGCGCCTGAGGGCGAGGGCGGGTCC
CGTGCGATGCAAGCGGCTATGGATGACGCCGGAATAGAACCGAAGGACGTACAATATCTCAATGCACACGGCACGAGCACCCCTGTGGGAGATC
TGAACGAAGTCAAGGCAATAAAGAATACGTTCGGTGAAGCTGCCAAGCACCTCAAAGTCAGCAGCACGAAGAGTATGACGGGGCACTTGCTCGG
GGCCACGGGTGGCATTGAAGCAATATTCTCCGCTCTCAGCATTAAGGACAGCAAAGTAGCACCGACCATTCACGCAGTAACTCCAGATCCGGAG
TGCGACCTTGATATCGTTCCGAACGAAGCGCAAGATCTTGACATTACGTACGCTATGTCAAATAGCCTTGGATTCGGCGGCCACAACGCGGTTC
TGGTCTTCAAGAAGTTTGAAGCCTAGCTCGAGCACCACCACCACCACCACTGAGATCCGGCTGCTAACAAAGCCCGAAAGGAAGCTGAGTTGGC
TGCTGCCACCGCTGAGCAATAACTAGCATAACCCCTTGGGGCCTCTAAACGGGTCTTGAGGGGTTTTTTGCTGAAAGGAGGAACTATATCCGGA
TTGGCGAATGGGACGCGCCCTGTAGCGGCGCATTAAGCGCGGCGGGTGTGGTGGTTACGCGCAGCGTGACCGCTACACTTGCCAGCGCCCTAGC
GCCCGCTCCTTTCGCTTTCTTCCCTTCCTTTCTCGCCACGTTCGCCGGCTTTCCCCGTCAAGCTCTAAATCGGGGGCTCCCTTTAGGGTTCCGA
TTTAGTGCTTTACGGCACCTCGACCCCAAAAAACTTGATTAGGGTGATGGTTCACGTAGTGGGCCATCGCCCTGATAGACGGTTTTTCGCCCTT
TGACGTTGGAGTCCACGTTCTTTAATAGTGGACTCTTGTTCCAAACTGGAACAACACTCAACCCTATCTCGGTCTATTCTTTTGATTTATAAGG
GATTTTGCCGATTTCGGCCTATTGGTTAAAAAATGAGCTGATTTAACAAAAATTTAACGCGAATTTTAACAAAATATTAACGCTTACAATTTAG
GTGGCACTTTTCGGGGAAATGTGCGCGGAACCCCTATTTGTTTATTTTTCTAAATACATTCAAATATGTATCCGCTCATGAATTAATTCTTAGA
AAAACTCATCGAGCATCAAATGAAACTGCAATTTATTCATATCAGGATTATCAATACCATATTTTTGAAAAAGCCGTTTCTGTAATGAAGGAGA
AAACTCACCGAGGCAGTTCCATAGGATGGCAAGATCCTGGTATCGGTCTGCGATTCCGACTCGTCCAACATCAATACAACCTATTAATTTCCCC
TCGTCAAAAATAAGGTTATCAAGTGAGAAATCACCATGAGTGACGACTGAATCCGGTGAGAATGGCAAAAGTTTATGCATTTCTTTCCAGACTT
GTTCAACAGGCCAGCCATTACGCTCGTCATCAAAATCACTCGCATCAACCAAACCGTTATTCATTCGTGATTGCGCCTGAGCGAGACGAAATAC
GCGATCGCTGTTAAAAGGACAATTACAAACAGGAATCGAATGCAACCGGCGCAGGAACACTGCCAGCGCATCAACAATATTTTCACCTGAATCA
GGATATTCTTCTAATACCTGGAATGCTGTTTTCCCGGGGATCGCAGTGGTGAGTAACCATGCATCATCAGGAGTACGGATAAAATGCTTGATGG
TCGGAAGAGGCATAAATTCCGTCAGCCAGTTTAGTCTGACCATCTCATCTGTAACATCATTGGCAACGCTACCTTTGCCATGTTTCAGAAACAA
CTCTGGCGCATCGGGCTTCCCATACAATCGATAGATTGTCGCACCTGATTGCCCGACATTATCGCGAGCCCATTTATACCCATATAAATCAGCA
TCCATGTTGGAATTTAATCGCGGCCTAGAGCAAGACGTTTCCCGTTGAATATGGCTCATAACACCCCTTGTATTACTGTTTATGTAAGCAGACA
GTTTTATTGTTCATGACCAAAATCCCTTAACGTGAGTTTTCGTTCCACTGAGCGTCAGACCCCGTAGAAAAGATCAAAGGATCTTCTTGAGATC
CTTTTTTTCTGCGCGTAATCTGCTGCTTGCAAACAAAAAAACCACCGCTACCAGCGGTGGTTTGTTTGCCGGATCAAGAGCTACCAACTCTTTT
TCCGAAGGTAACTGGCTTCAGCAGAGCGCAGATACCAAATACTGTCCTTCTAGTGTAGCCGTAGTTAGGCCACCACTTCAAGAACTCTGTAGCA
CCGCCTACATACCTCGCTCTGCTAATCCTGTTACCAGTGGCTGCTGCCAGTGGCGATAAGTCGTGTCTTACCGGGTTGGACTCAAGACGATAGT
TACCGGATAAGGCGCAGCGGTCGGGCTGAACGGGGGGTTCGTGCACACAGC

^{1}H-, ^{13}C-NMR-Spectra

Sulfinic acids

Butane-1-sulfinic acid

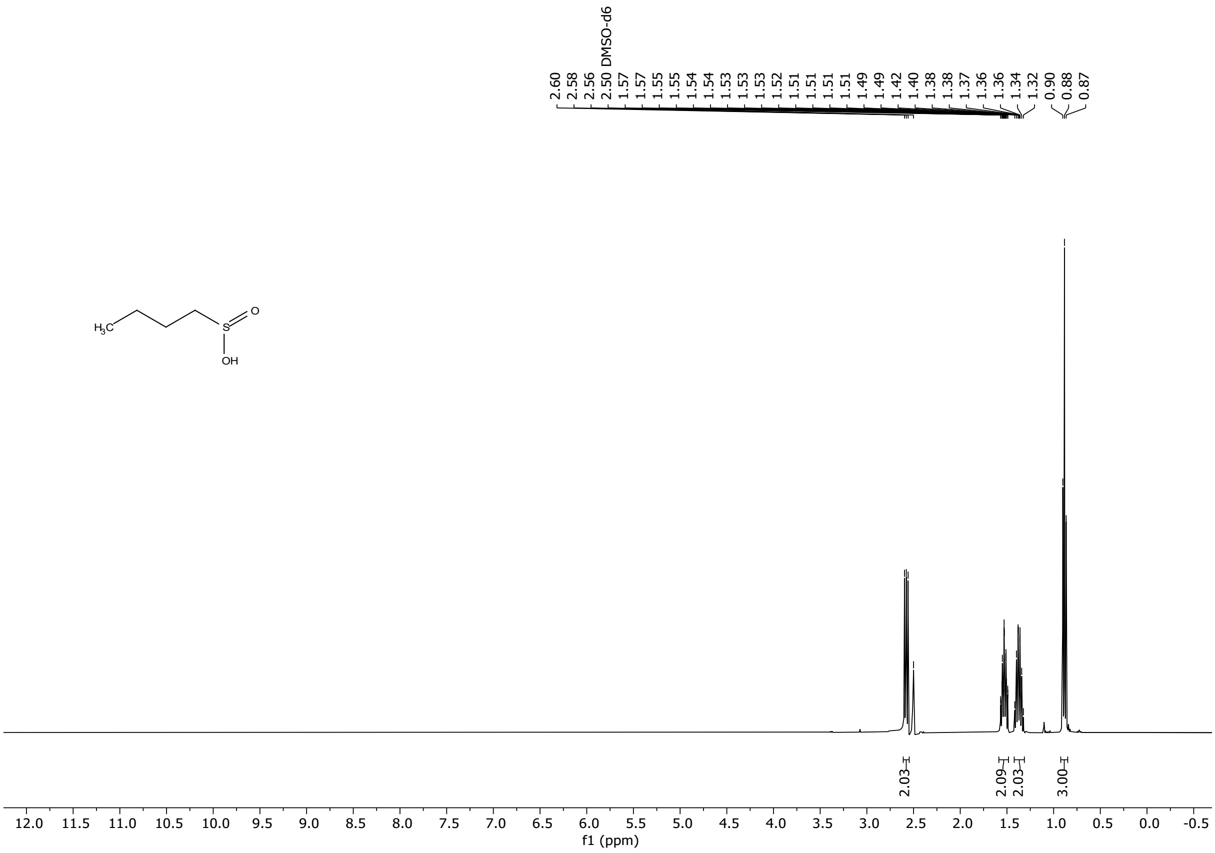

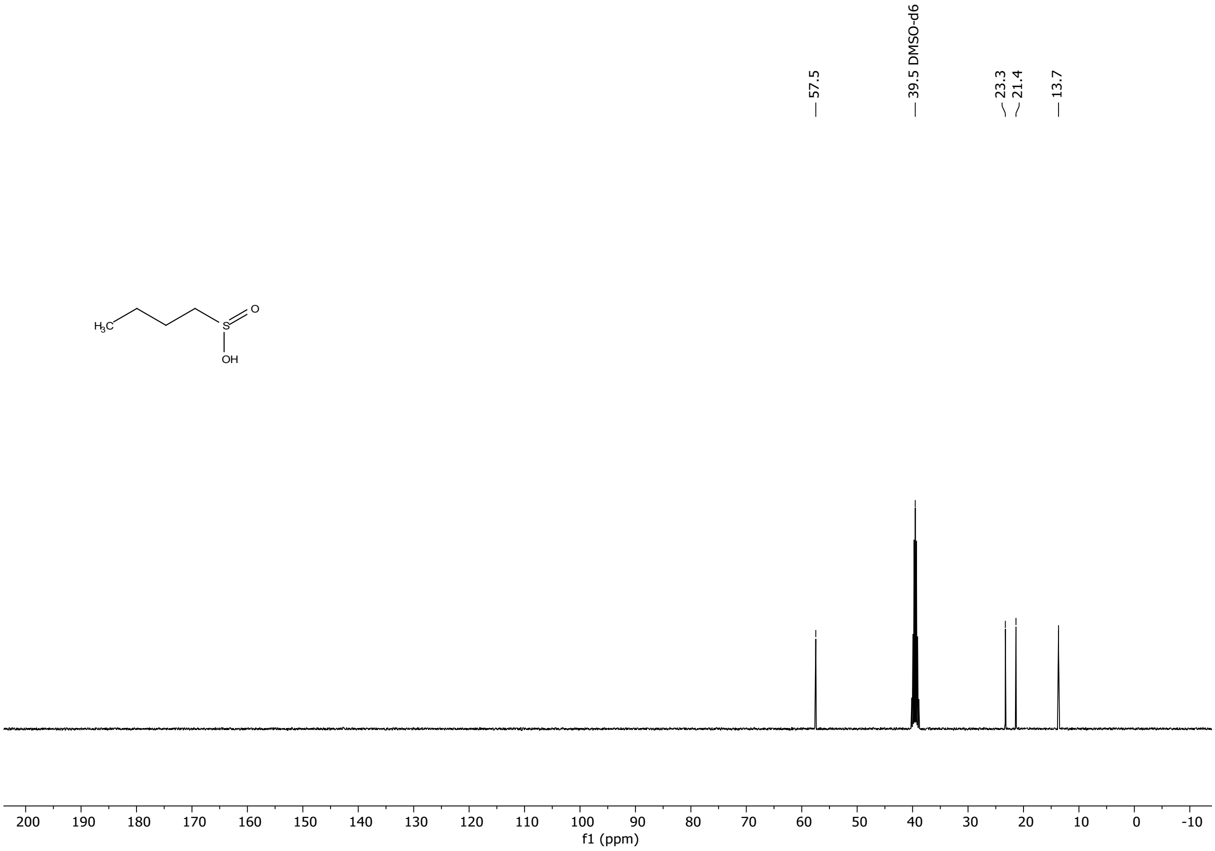

4-methoxybenzenesulfinic acid

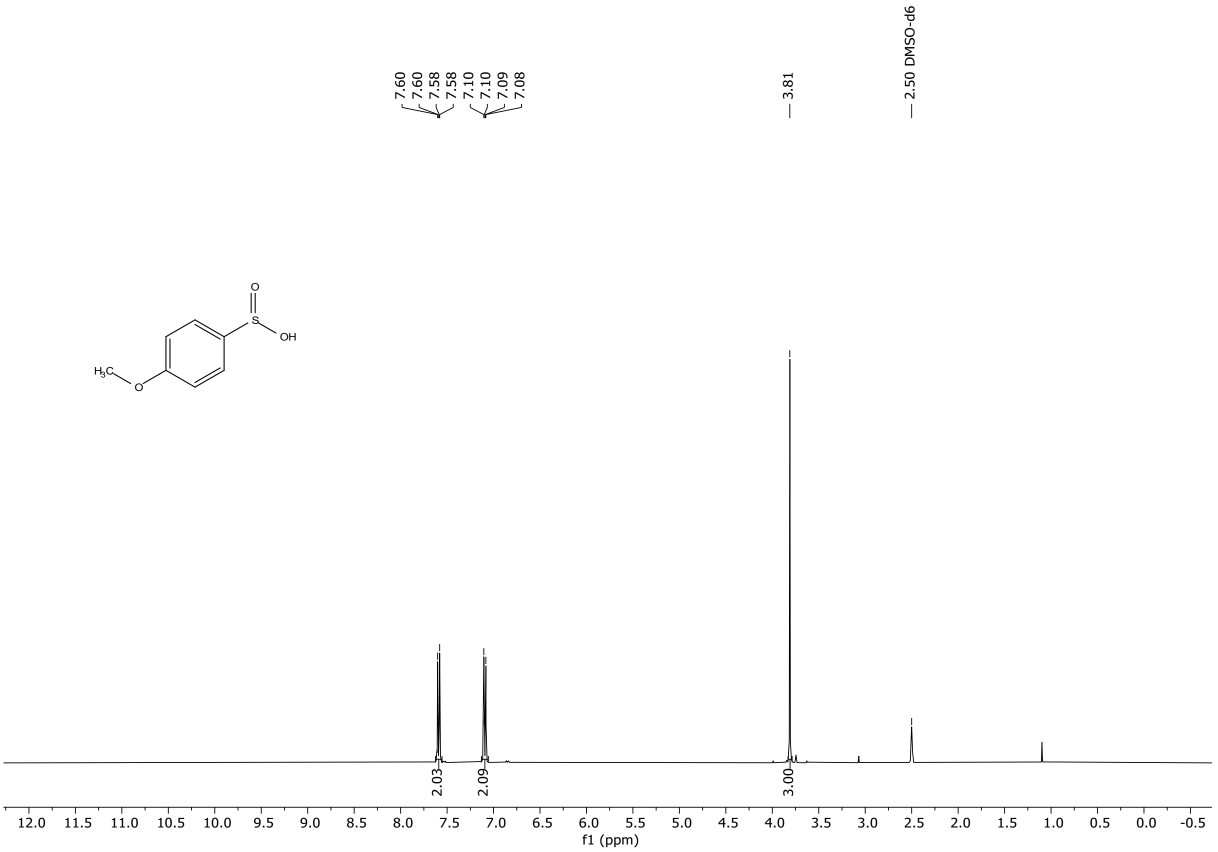

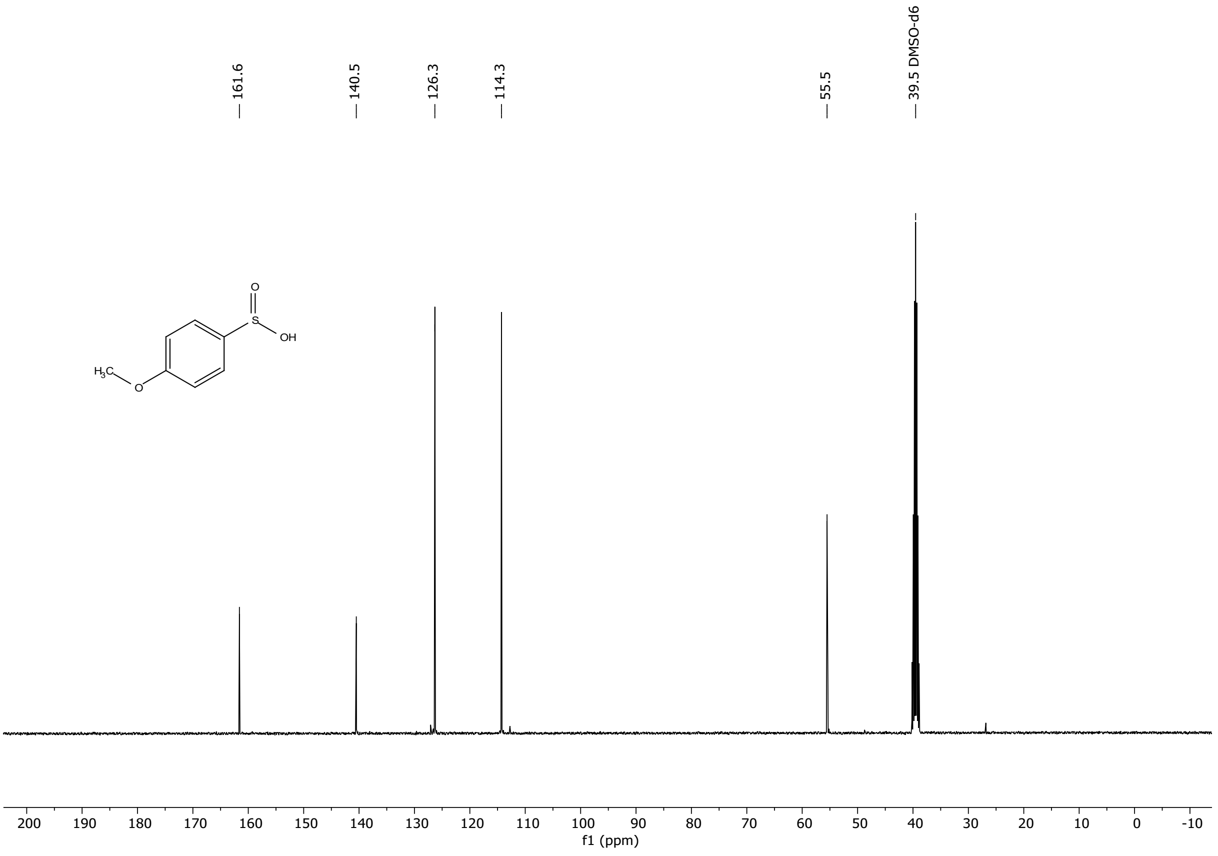

4-fluorobenzenesulfinic acid

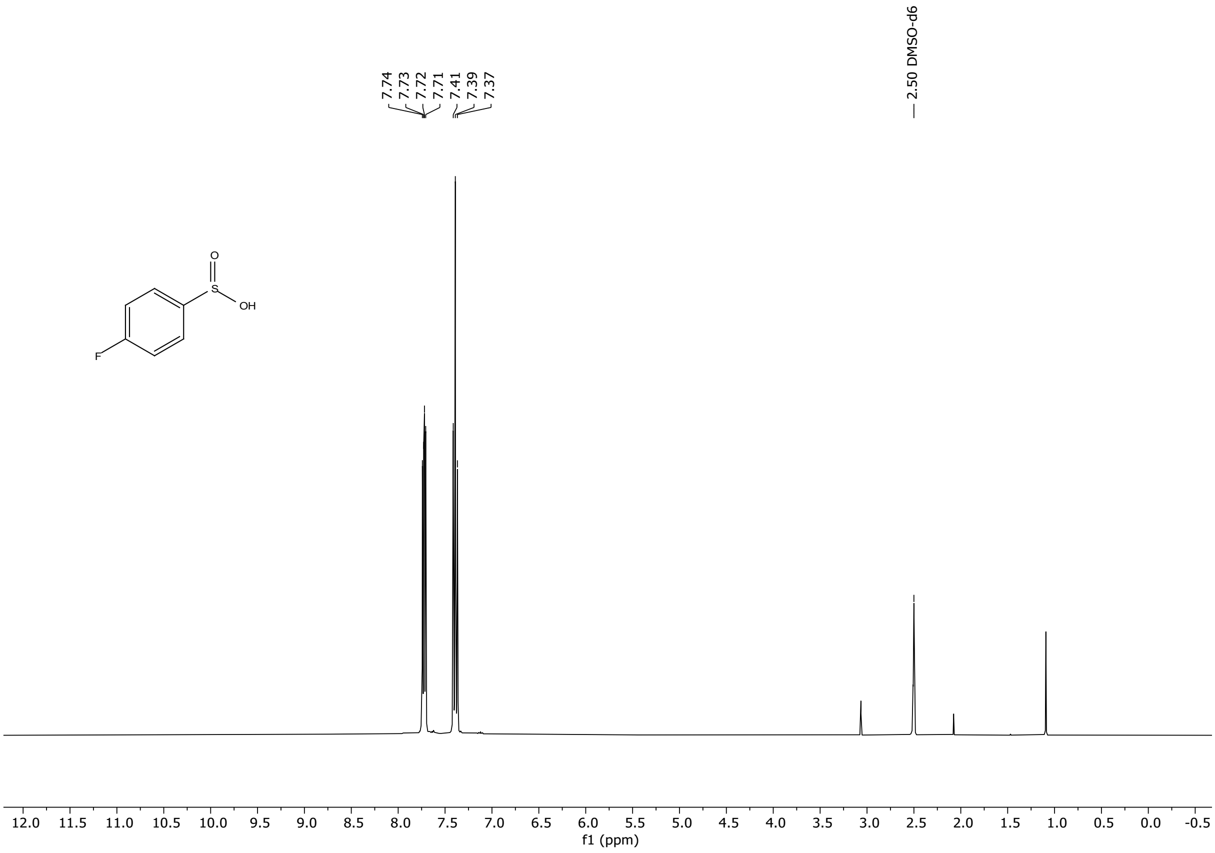

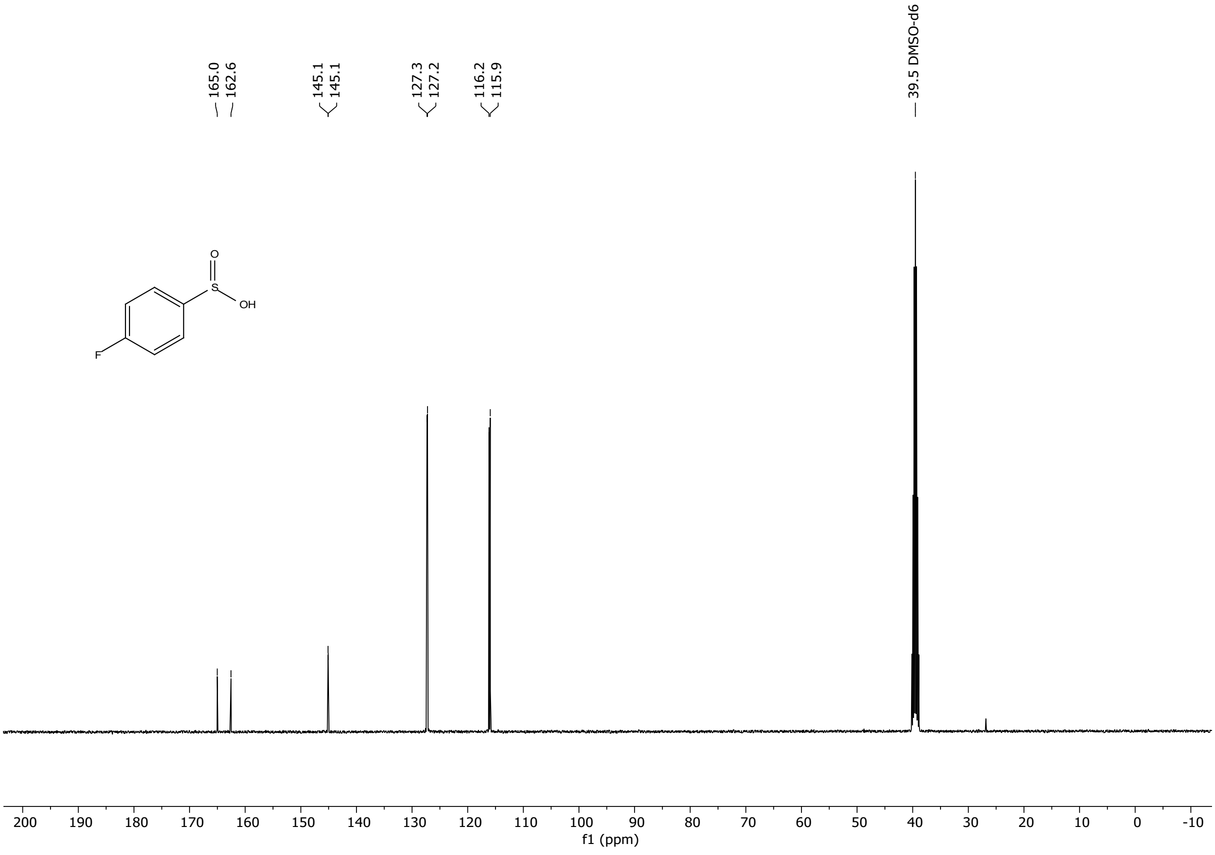

Formamides

N-((2-chloro-4-fluorophenyl)(tosyl)methyl)formamide

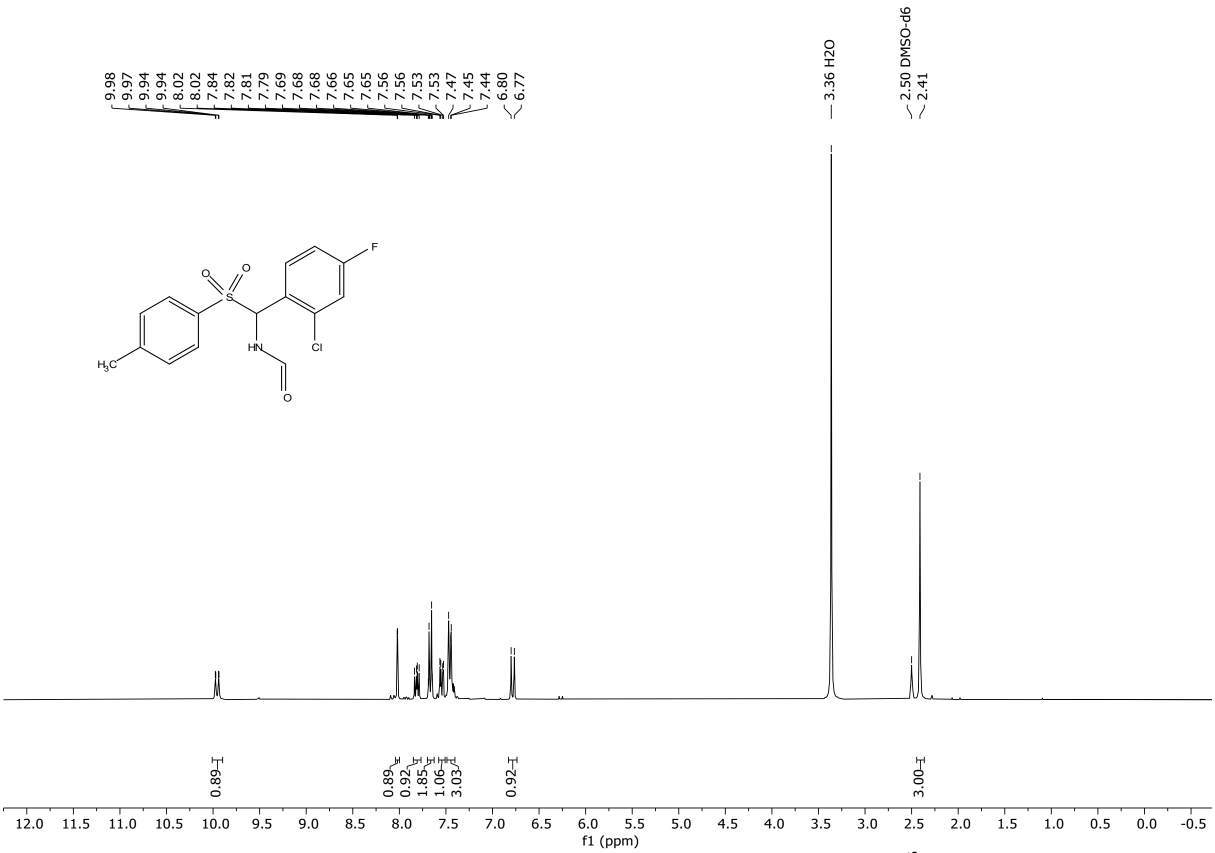

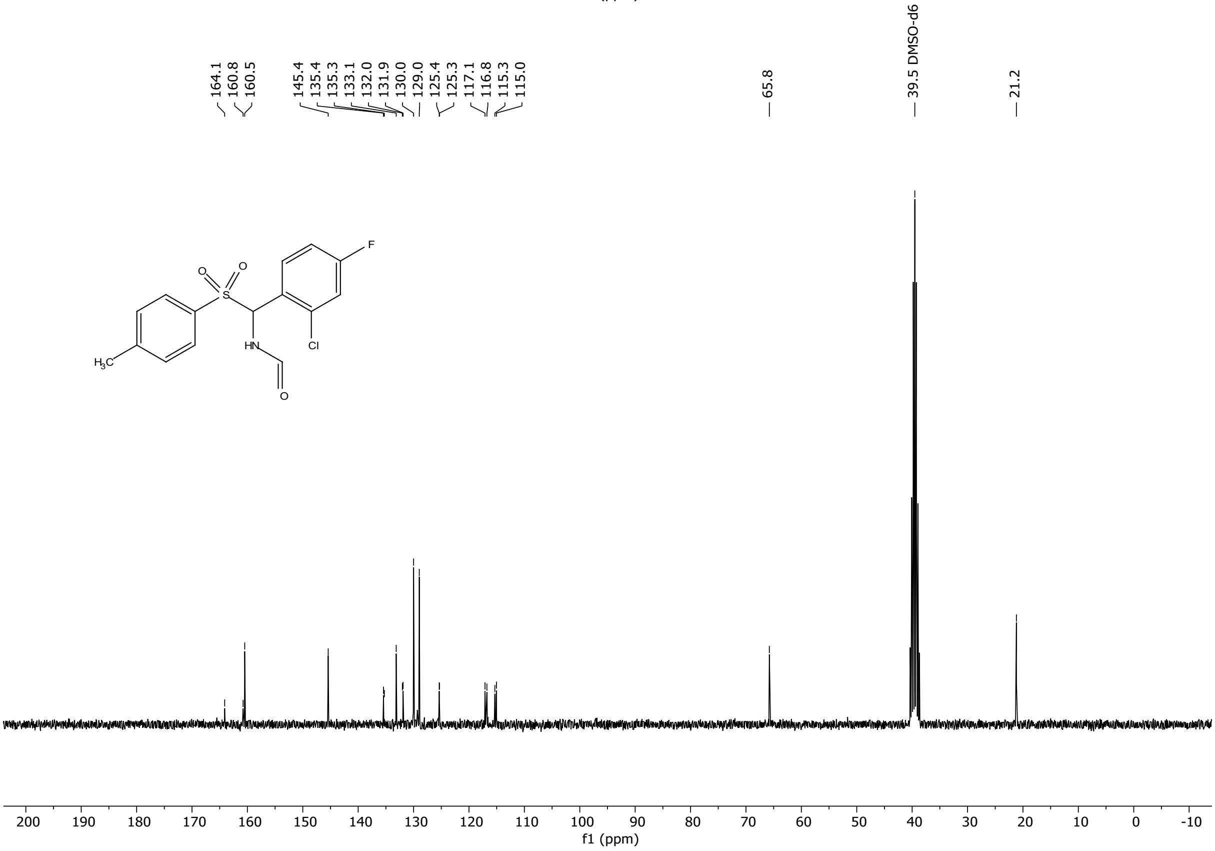

N-((4-fluorophenyl)(tosyl)methyl)formamide

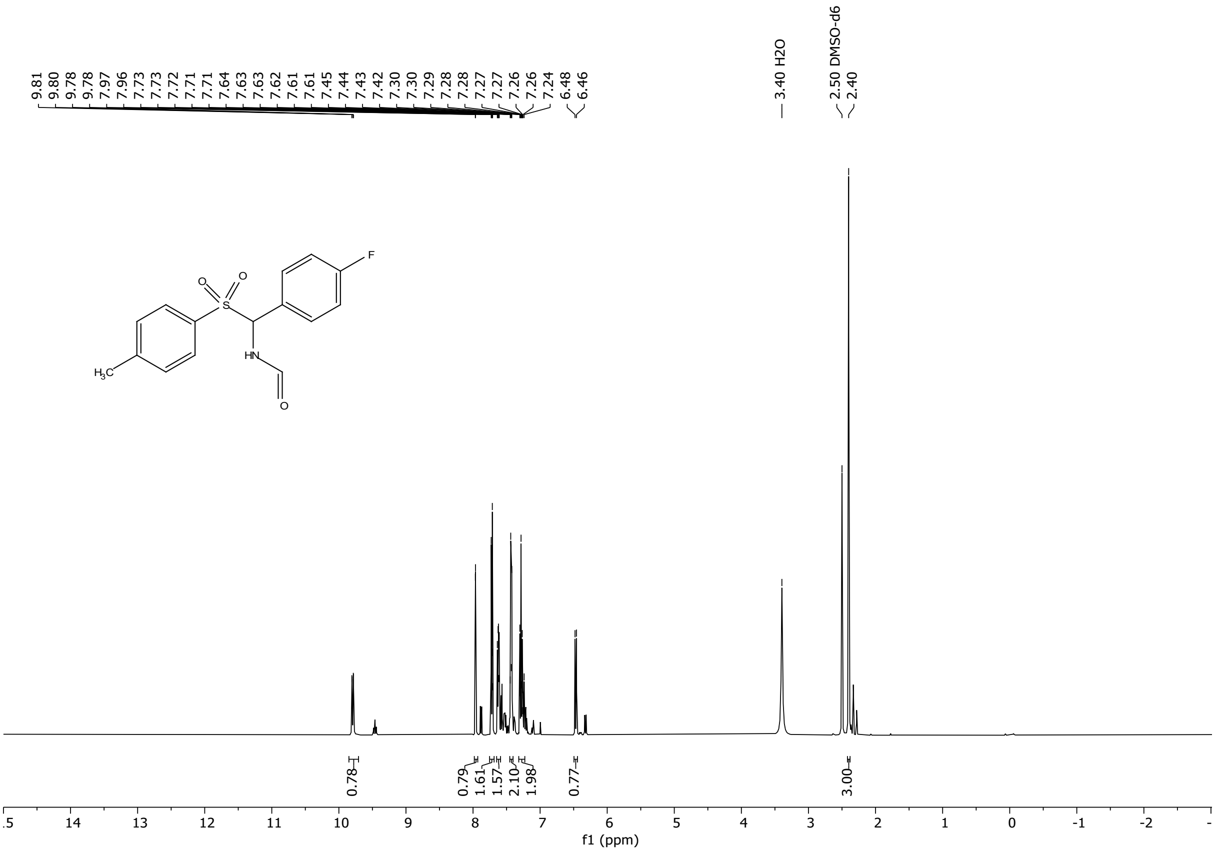

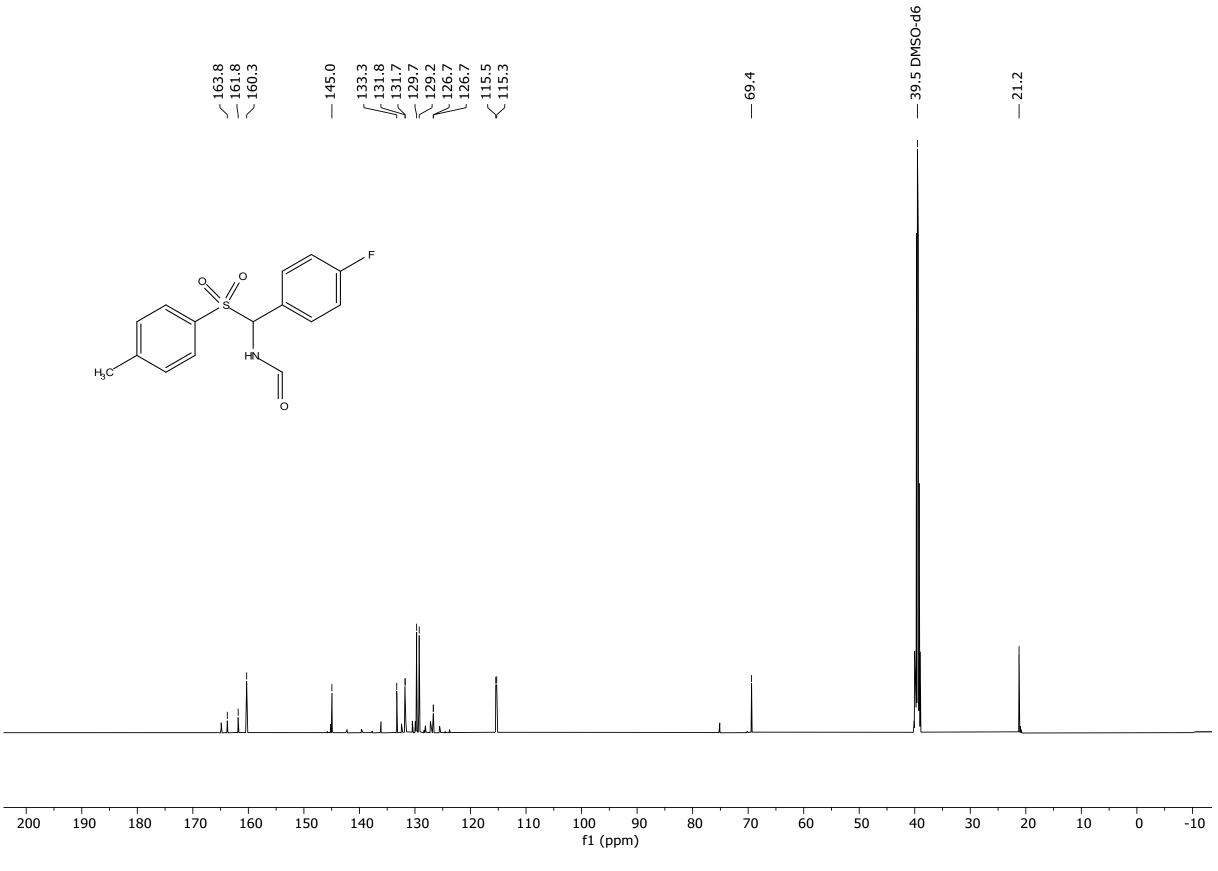

N-((3-fluorophenyl)(tosyl)methyl)formamide

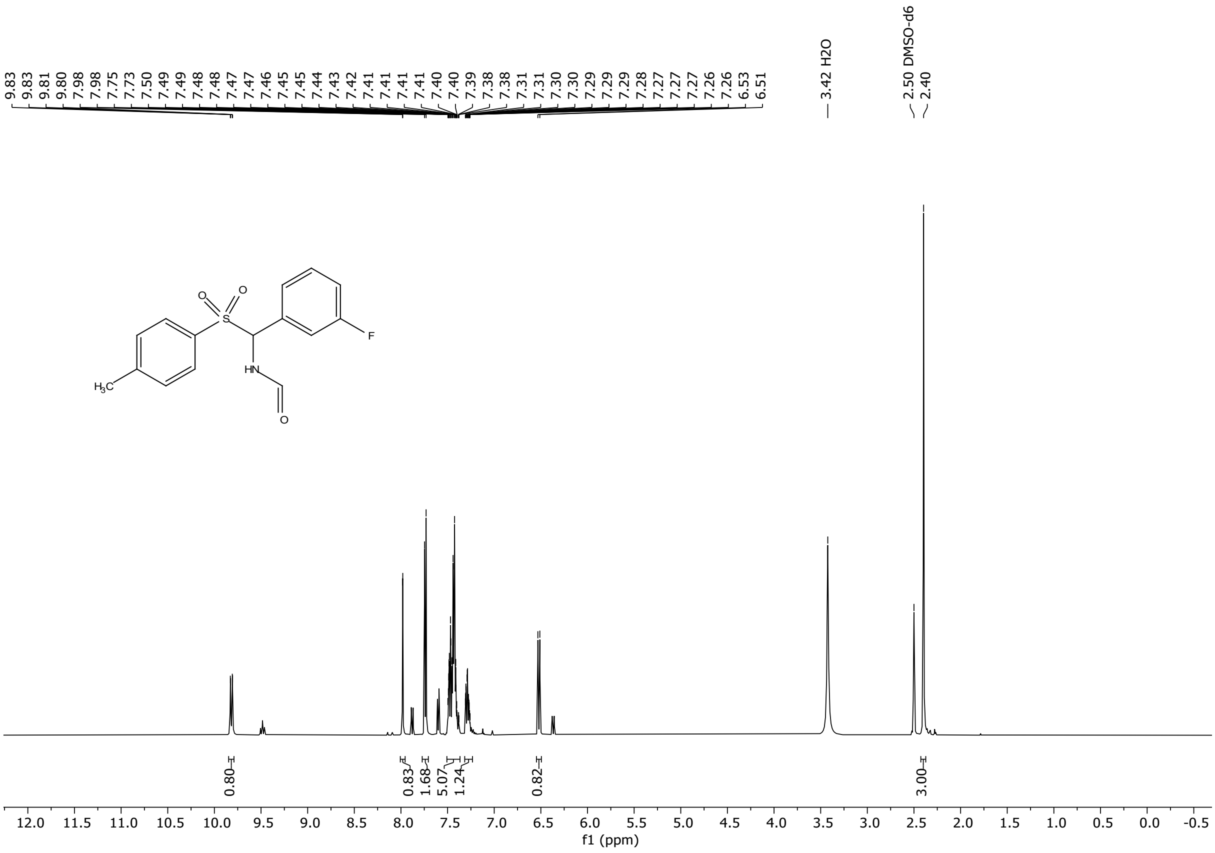

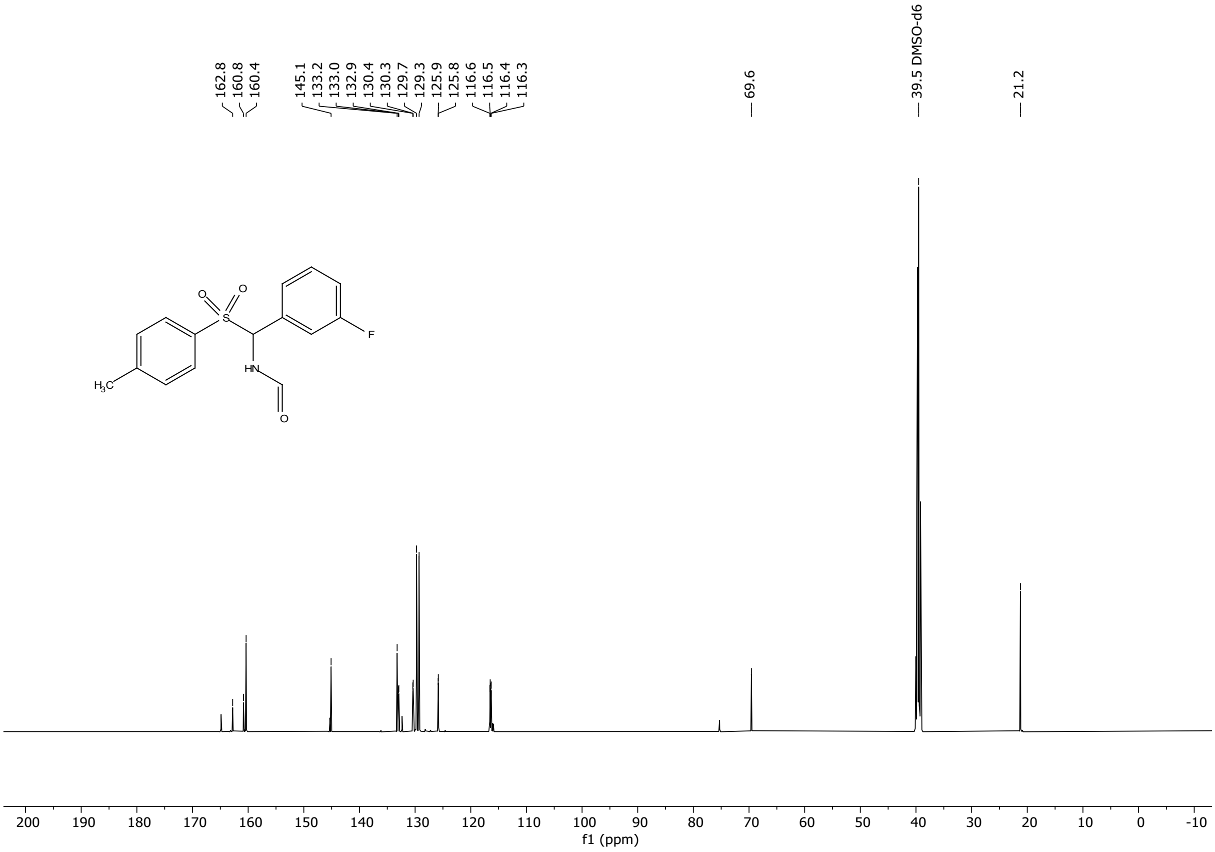

N-((2-fluorophenyl)(tosyl)methyl)formamide

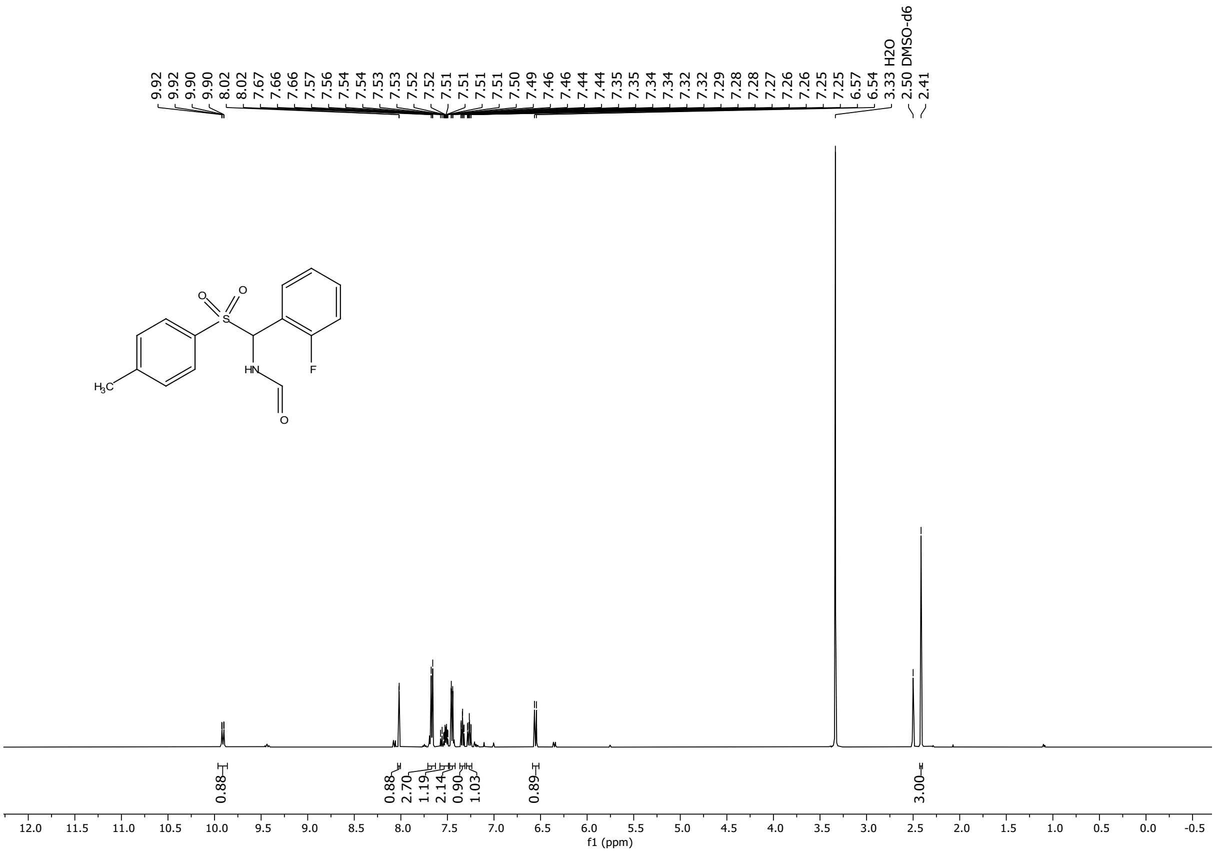

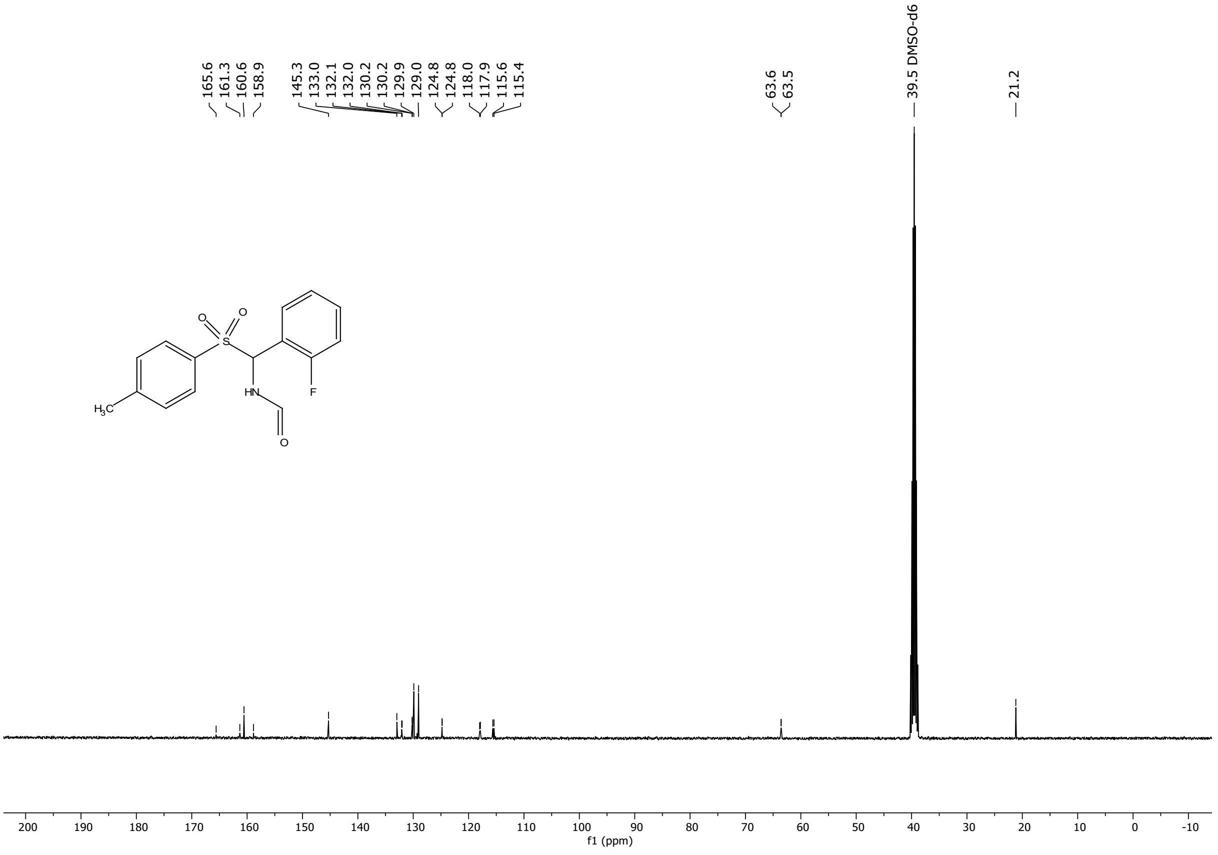

N-((2-chlorophenyl)(tosyl)methyl)formamide

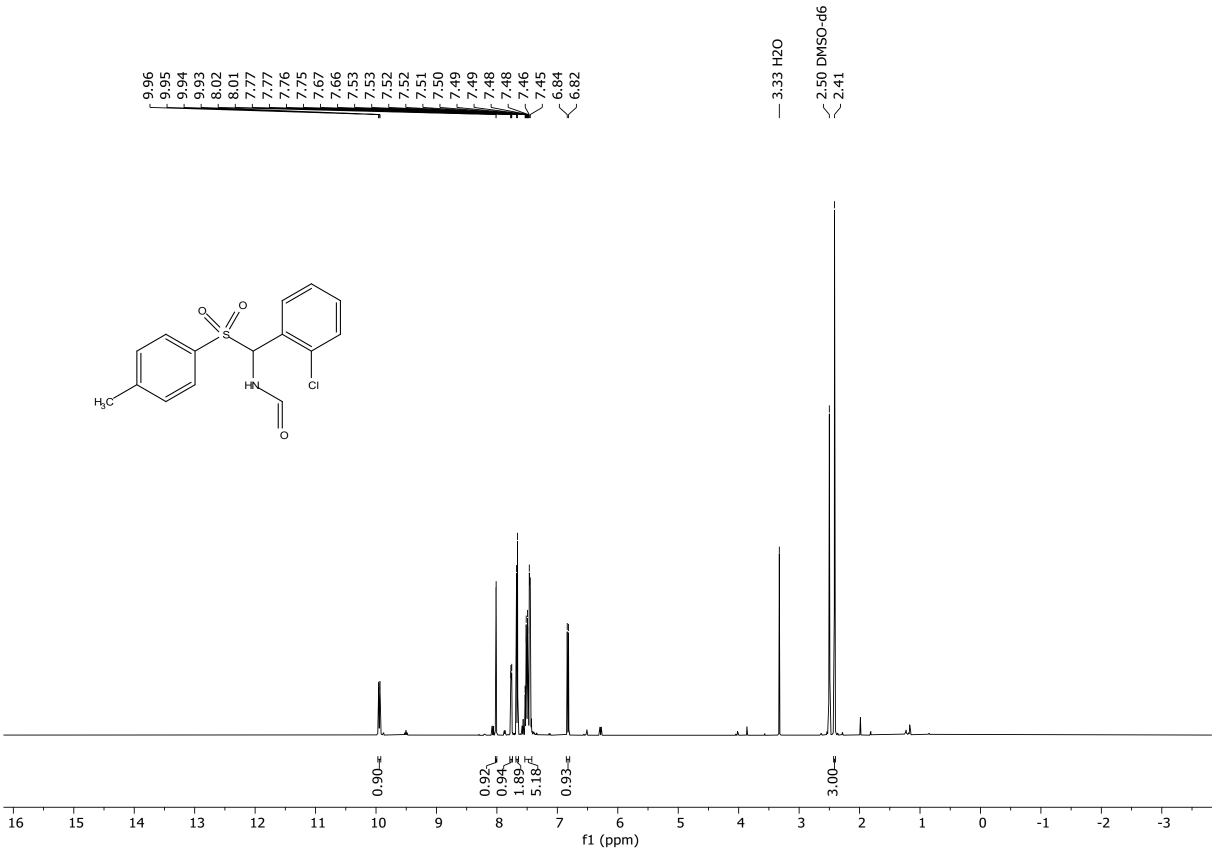

N-((4-cyanophenyl)(tosyl)methyl)formamide

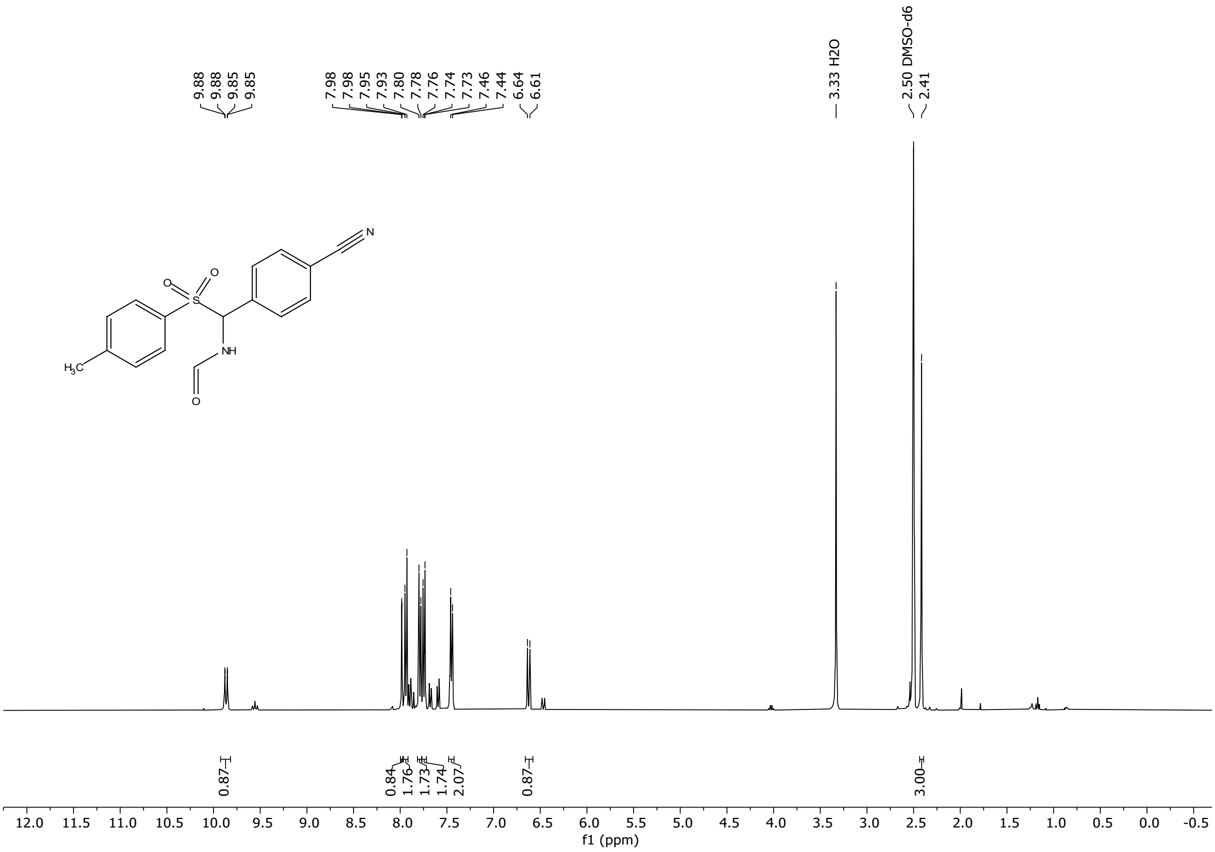

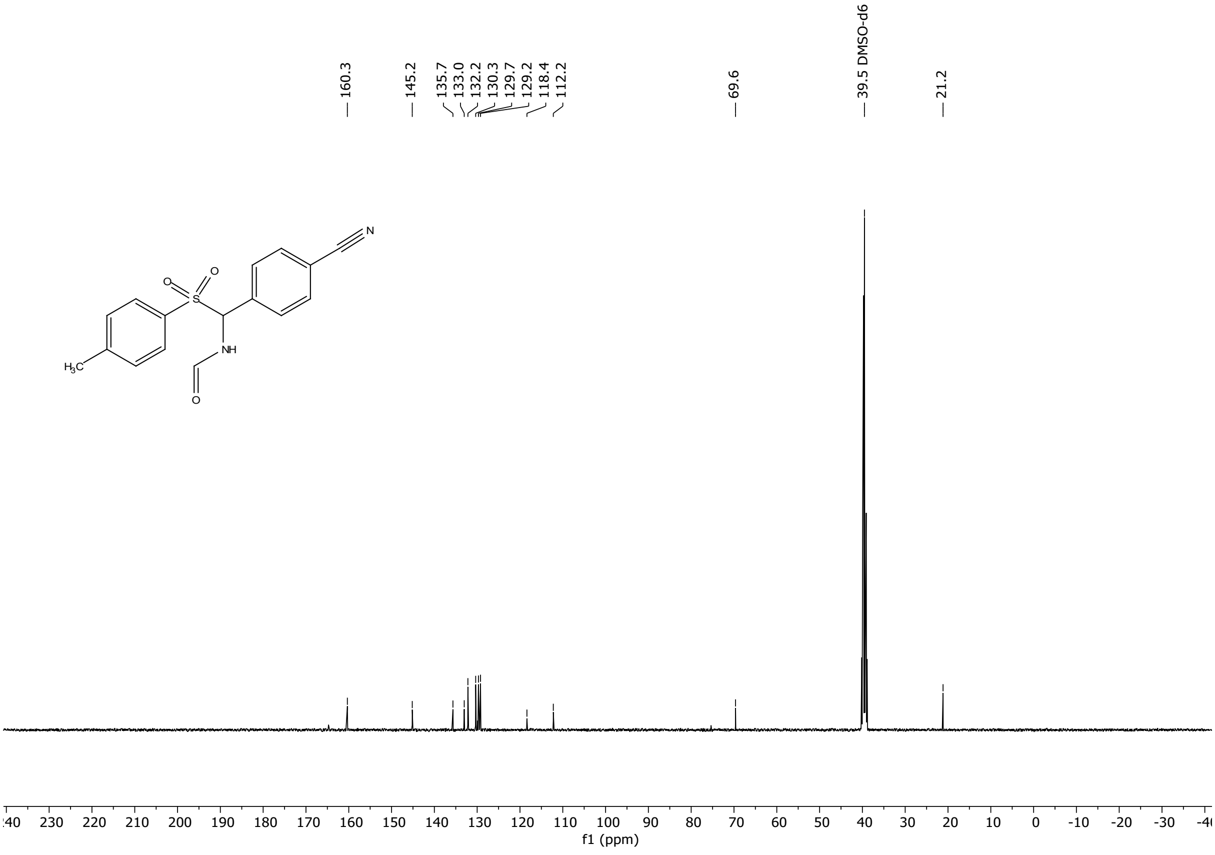

N-(phenyl(tosyl)methyl)formamide

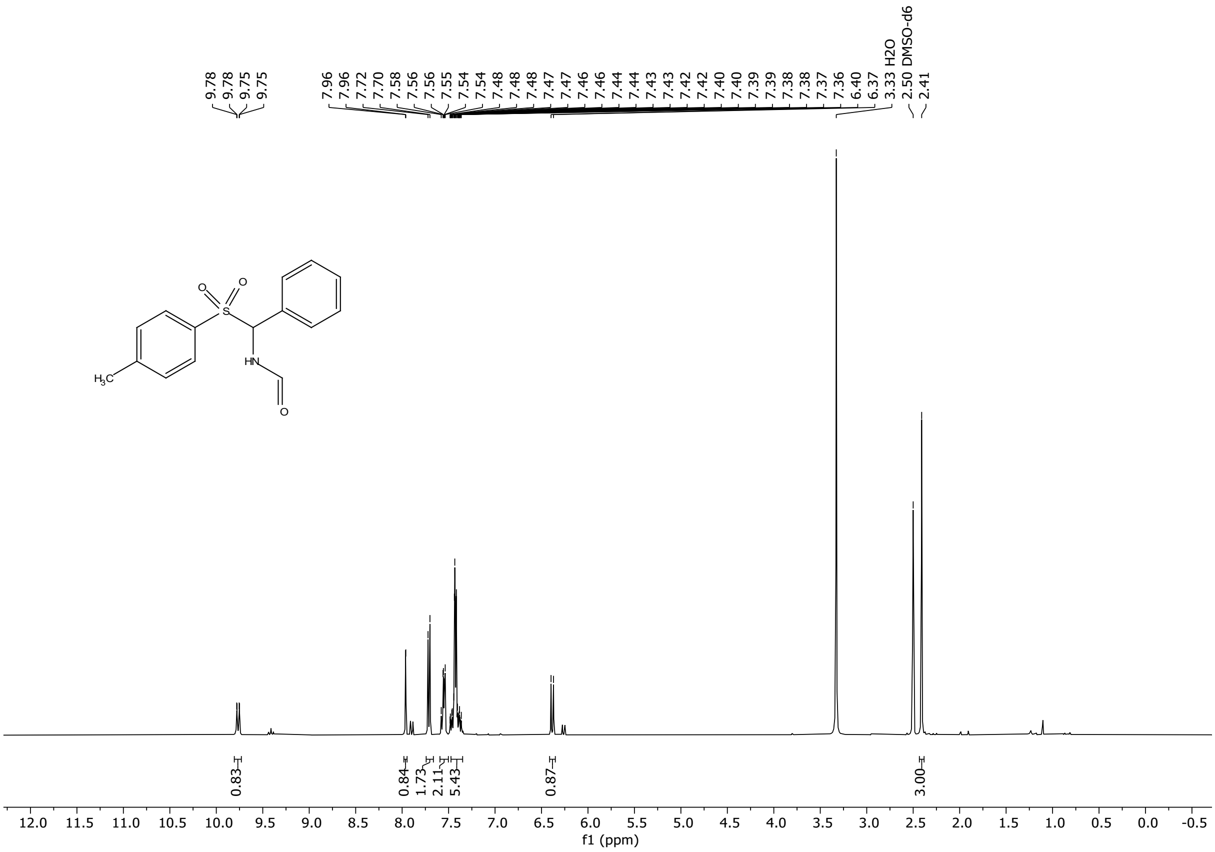

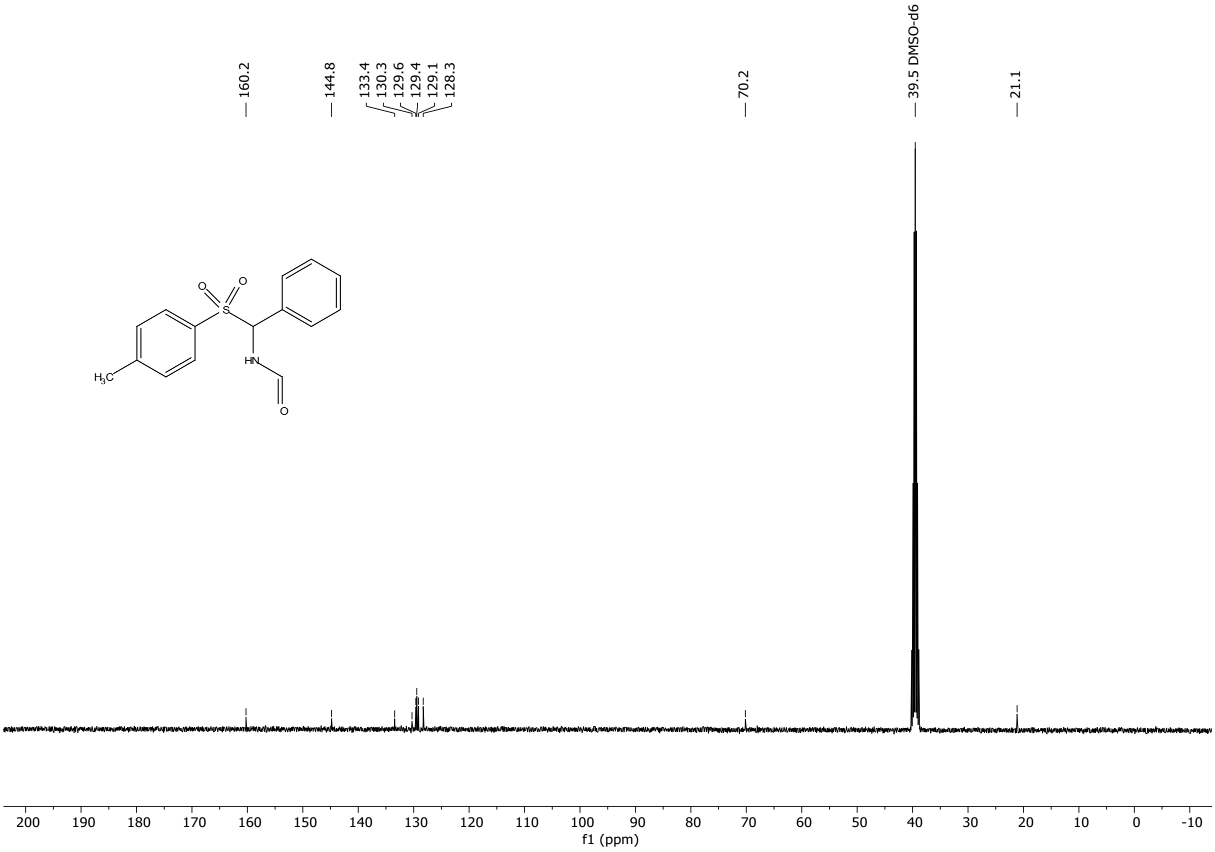

N-(bicyclo[2.2.1]hept-5-en-2-yl(tosyl)methyl)formamide

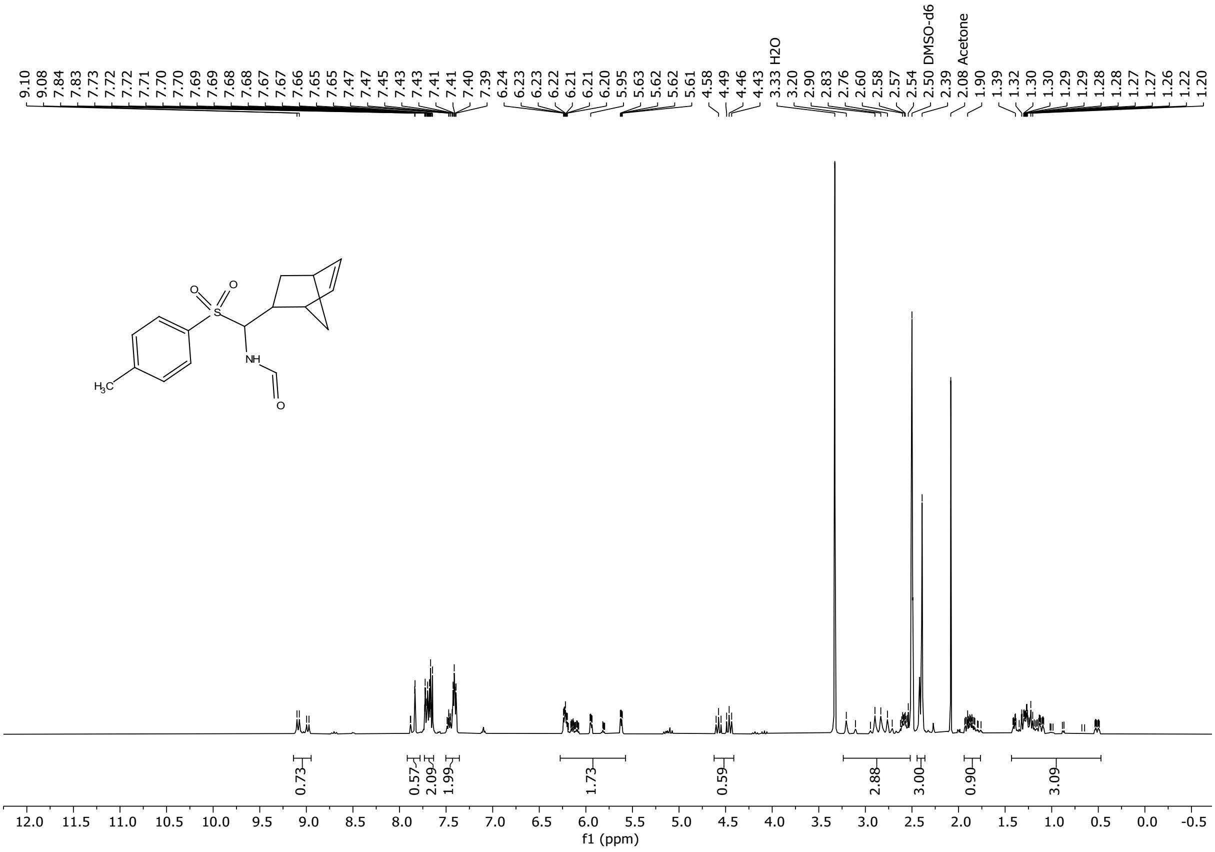

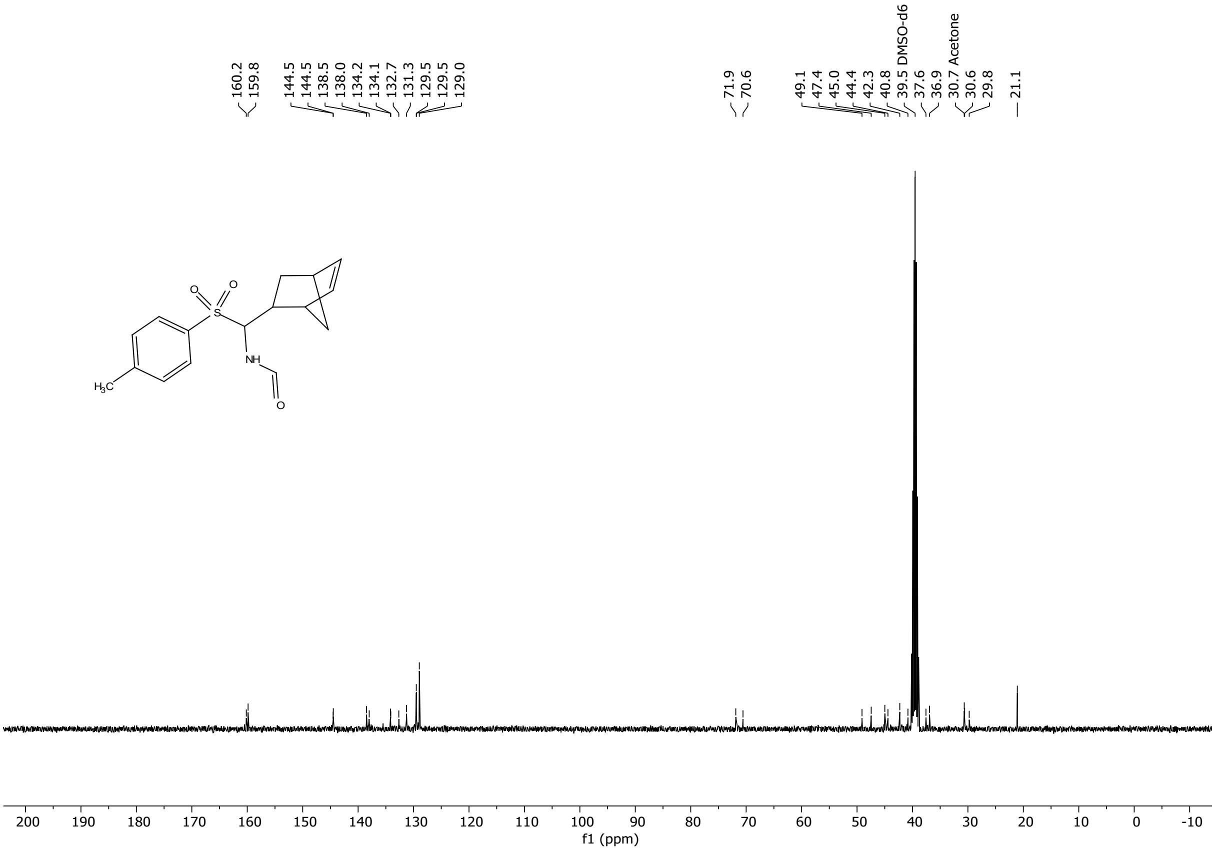

N-(1-tosylbutyl)formamide

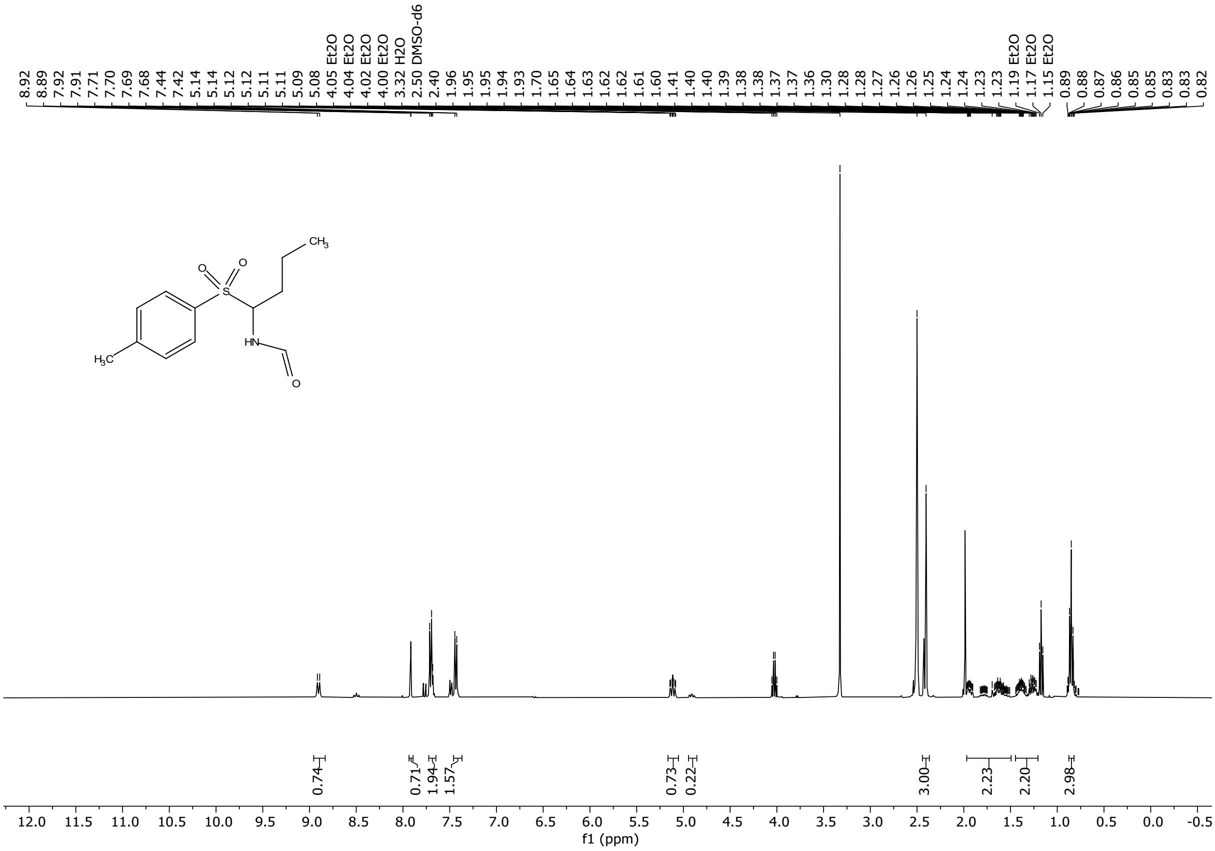

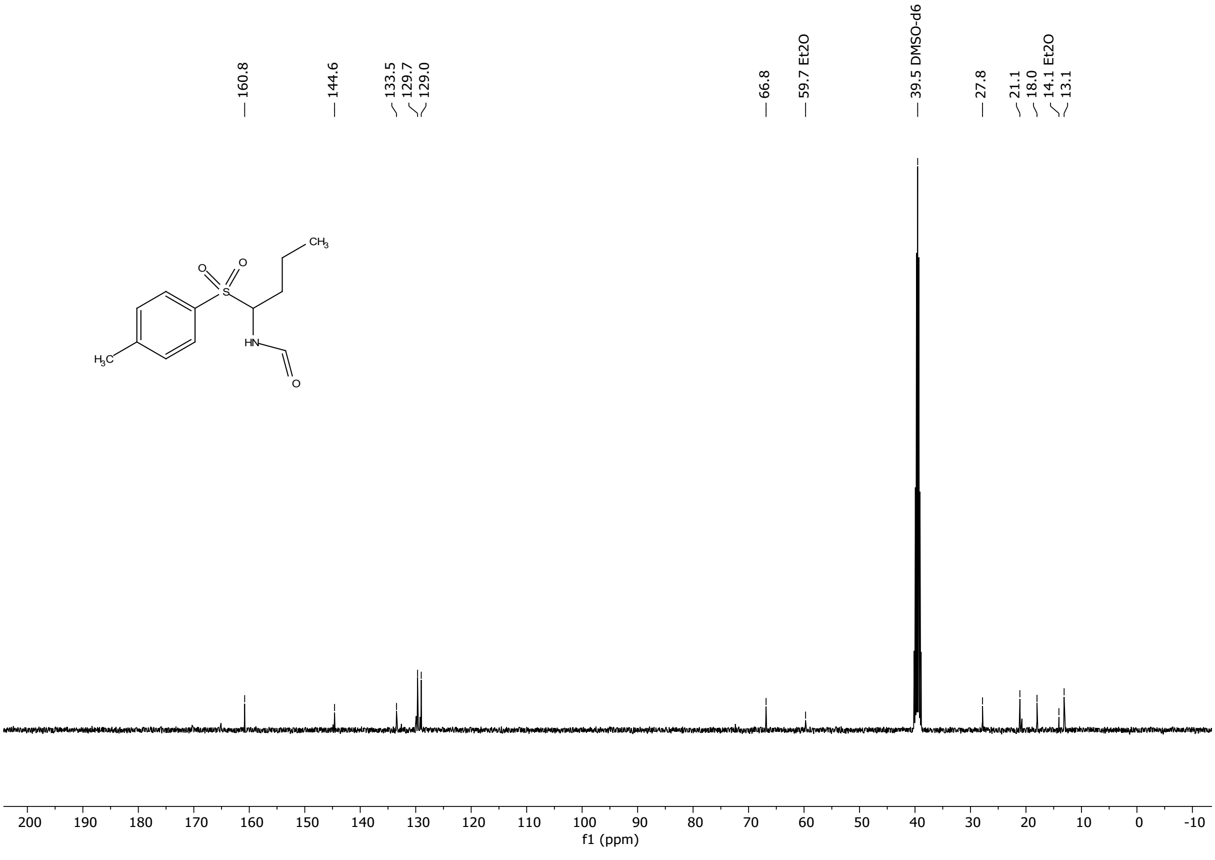

N-((butylsulfonyl)(2-chloro-4-fluorophenyl)methyl)formamide

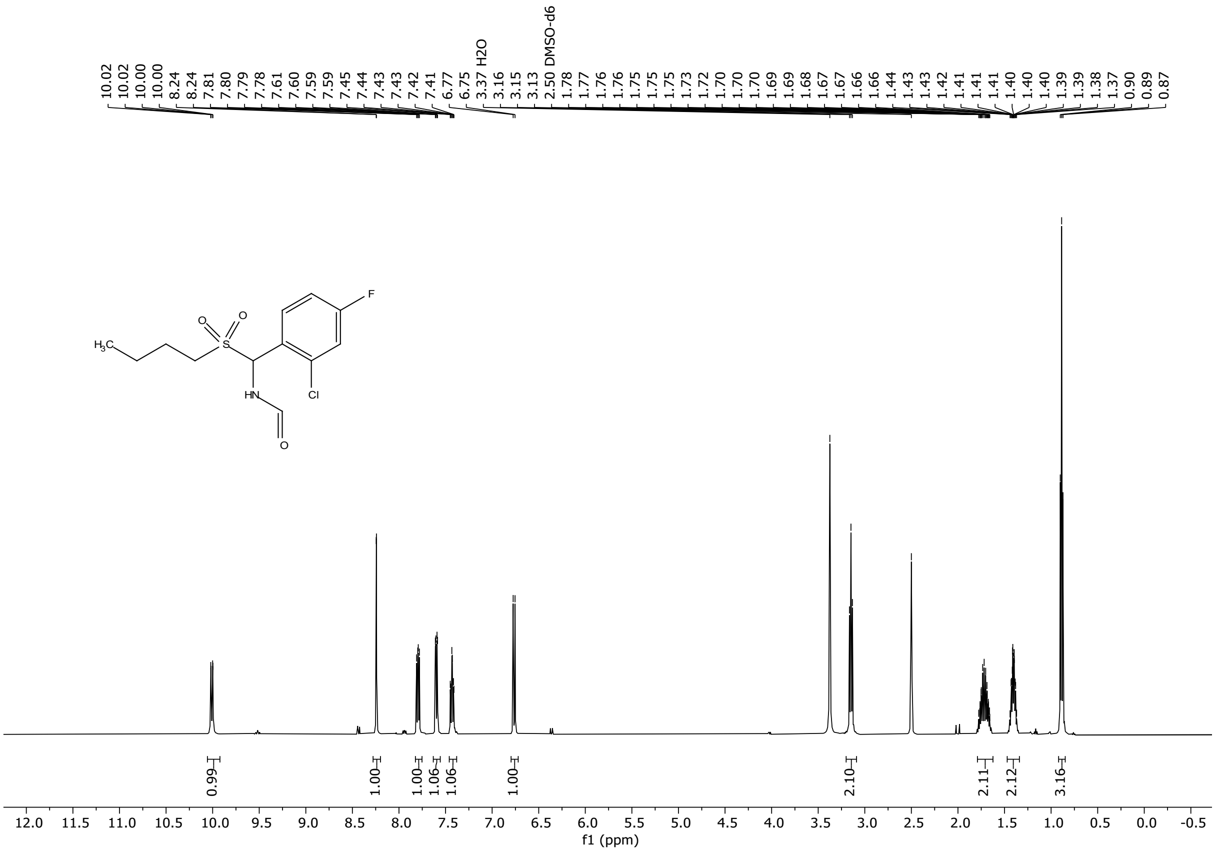

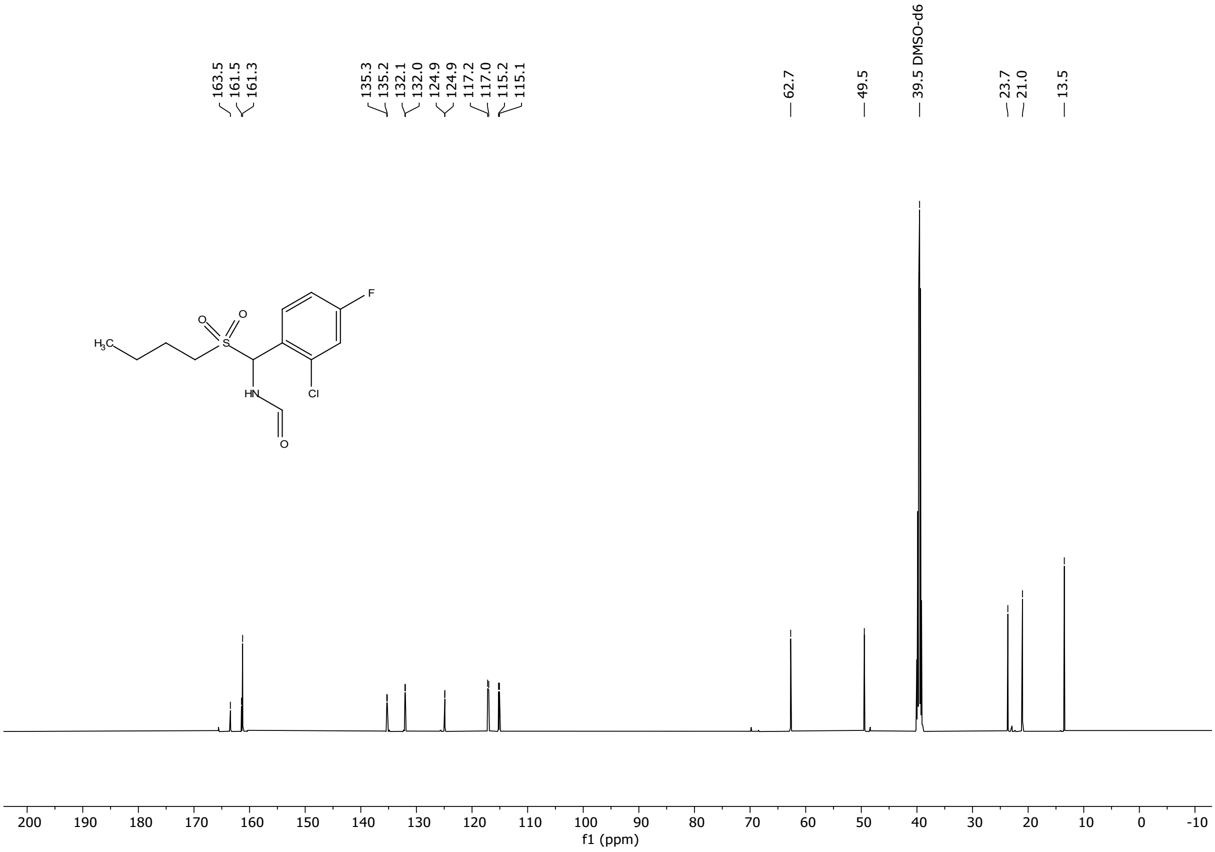

N-((2-chloro-4-fluorophenyl)(methylsulfonyl)methyl)formamide

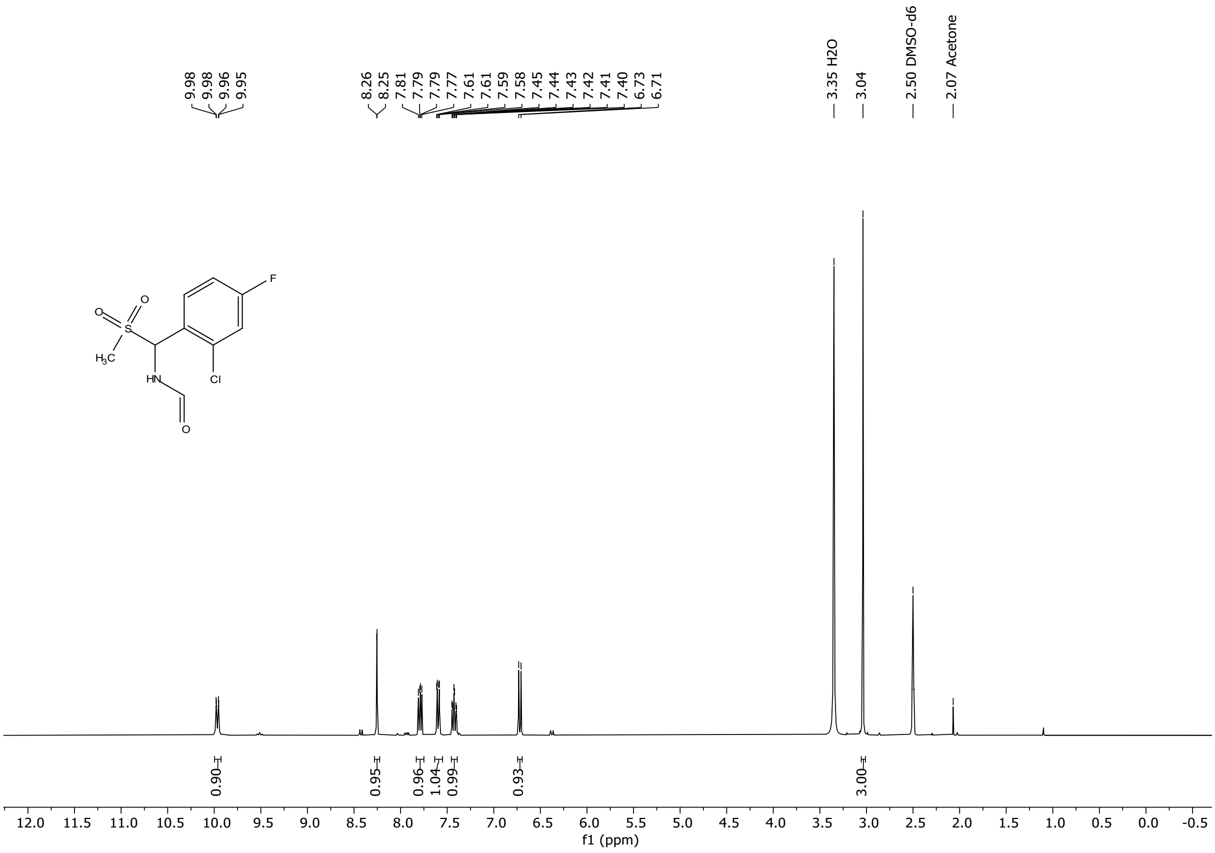

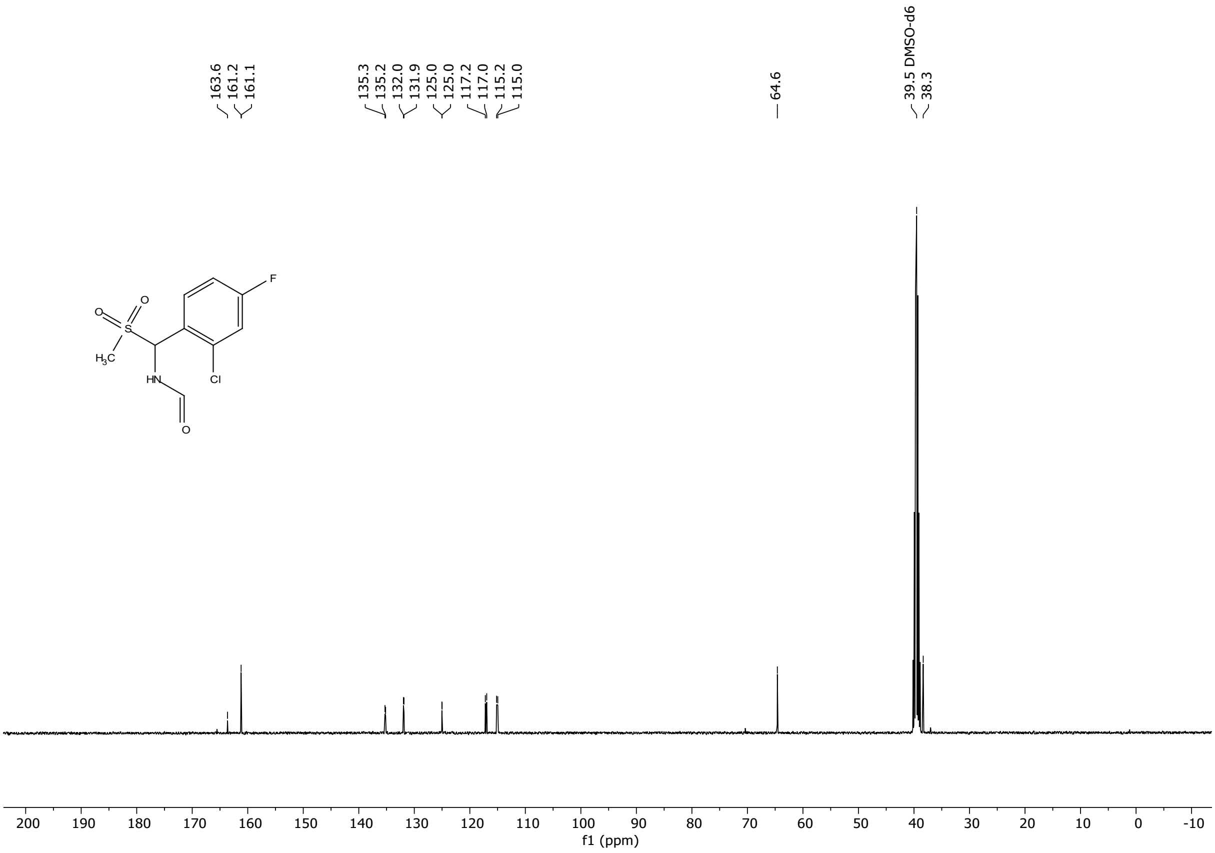

N-((2-chloro-4-fluorophenyl)(phenylsulfonyl)methyl)formamide

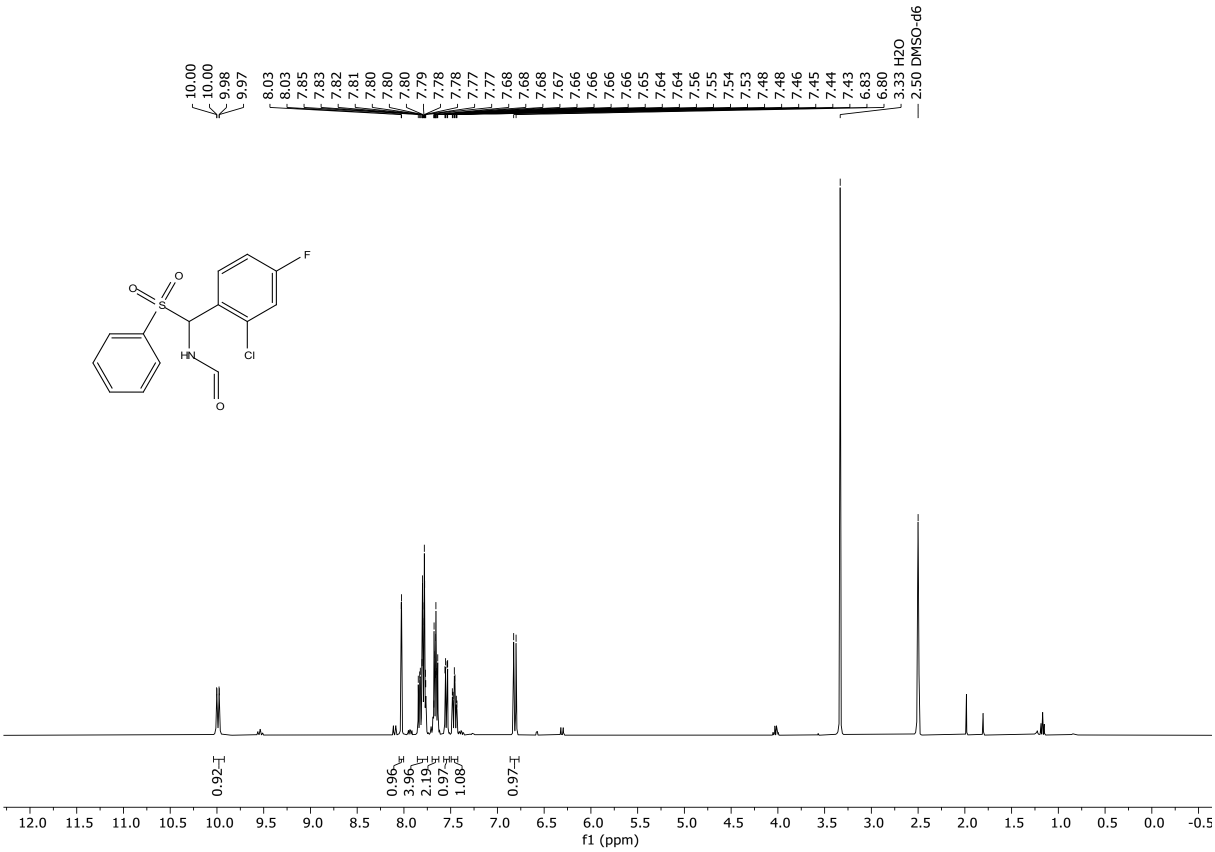

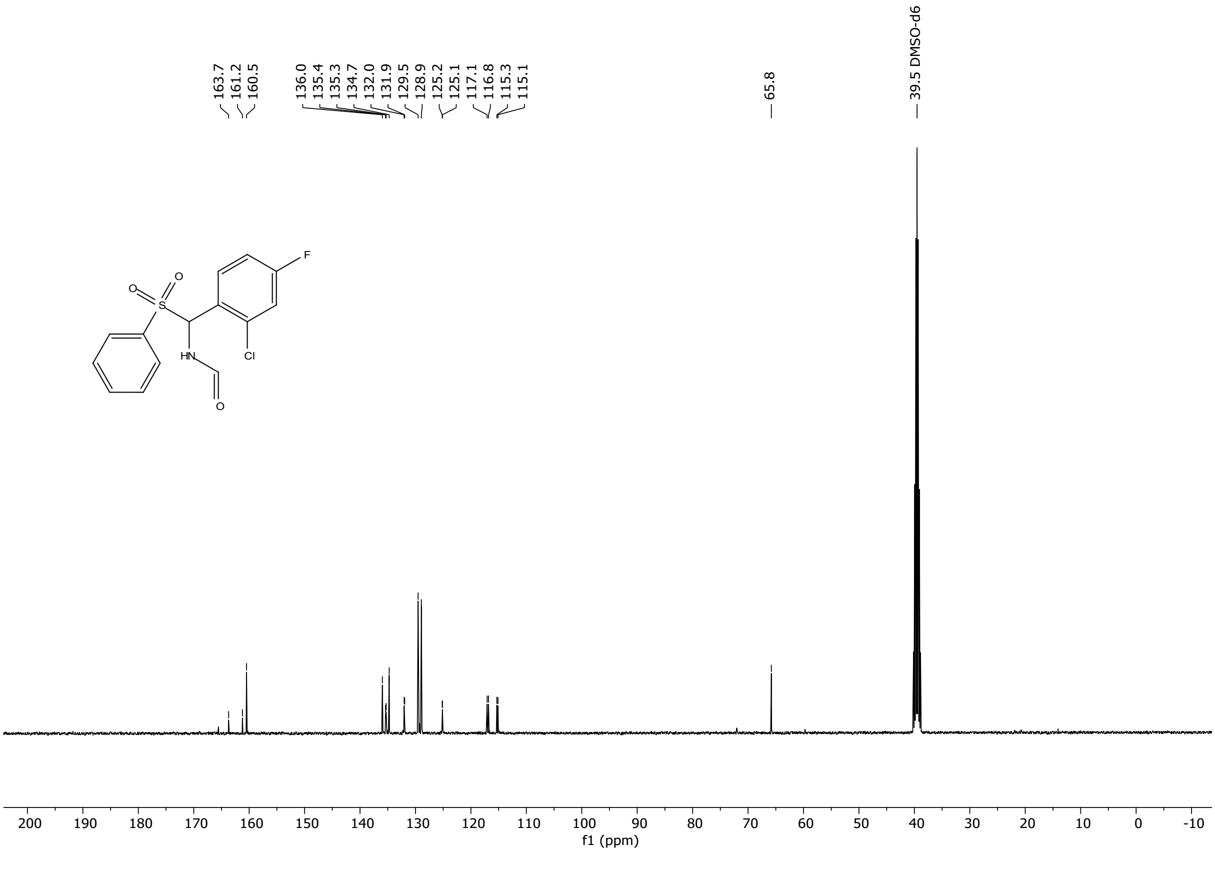

N-((2-chloro-4-fluorophenyl)((4-methoxyphenyl)sulfonyl)methyl)formamide

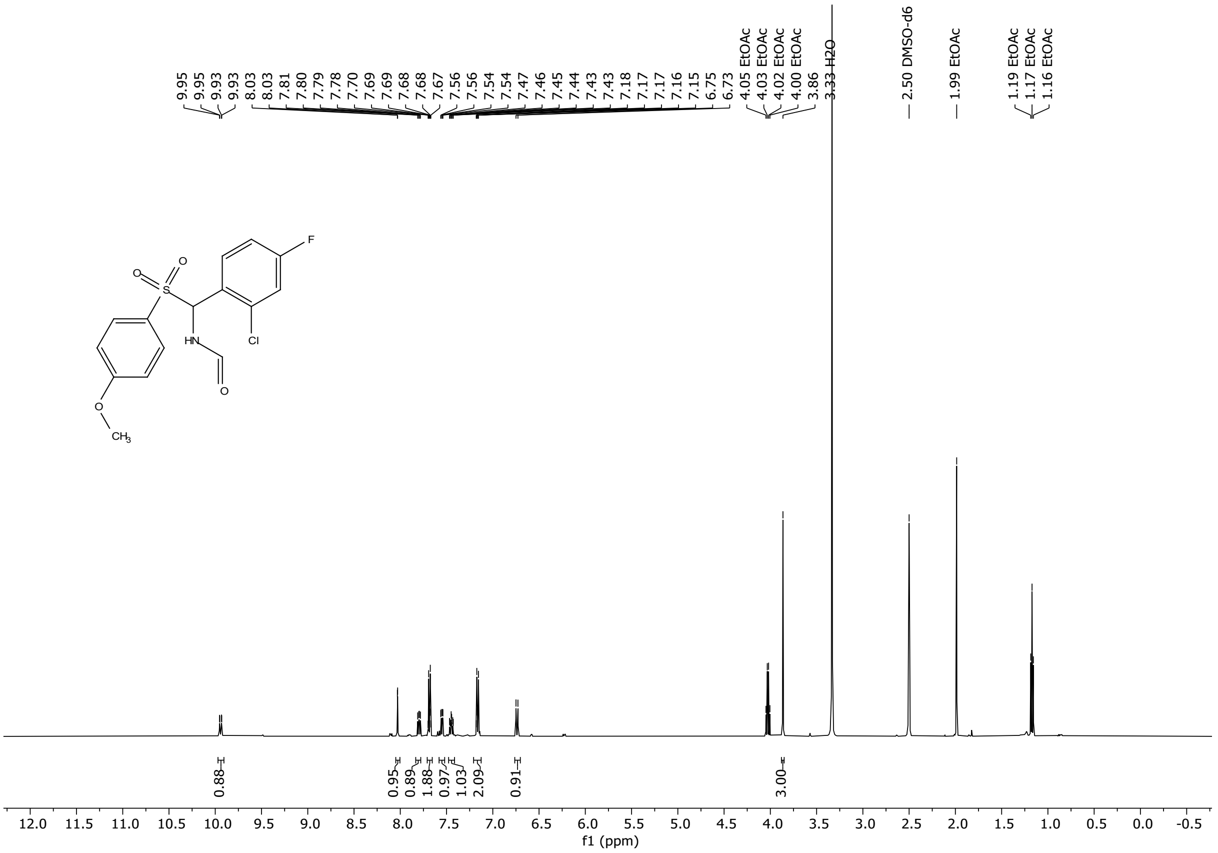

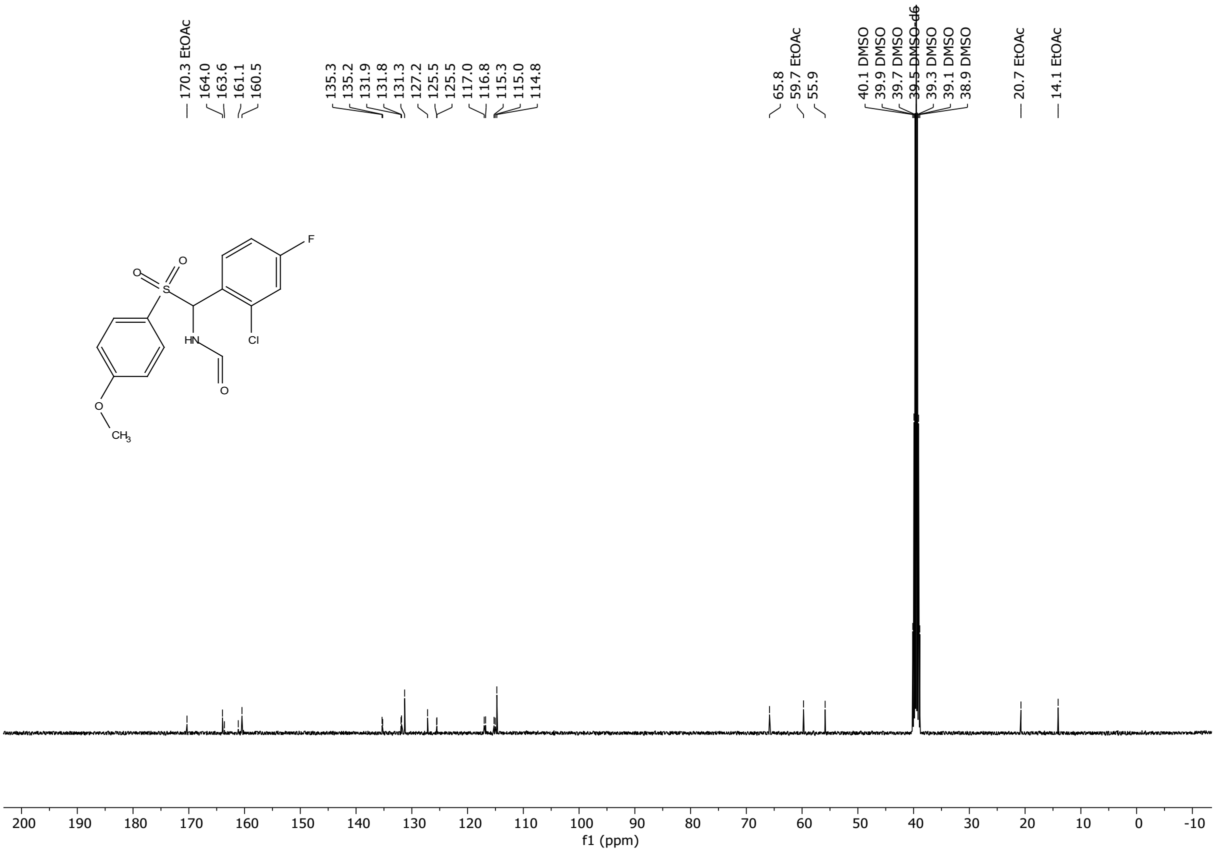

N-((2-chloro-4-fluorophenyl)((4-fluorophenyl)sulfonyl)methyl)formamide

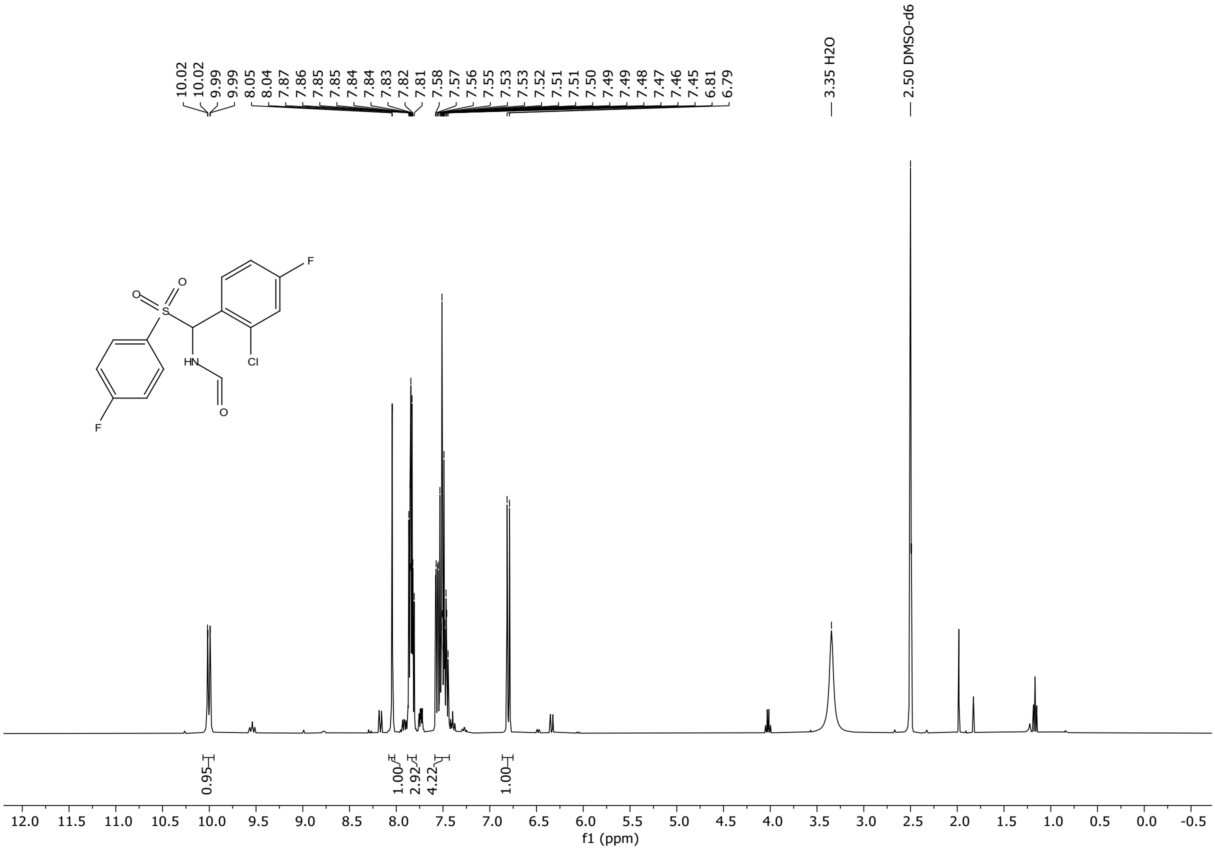

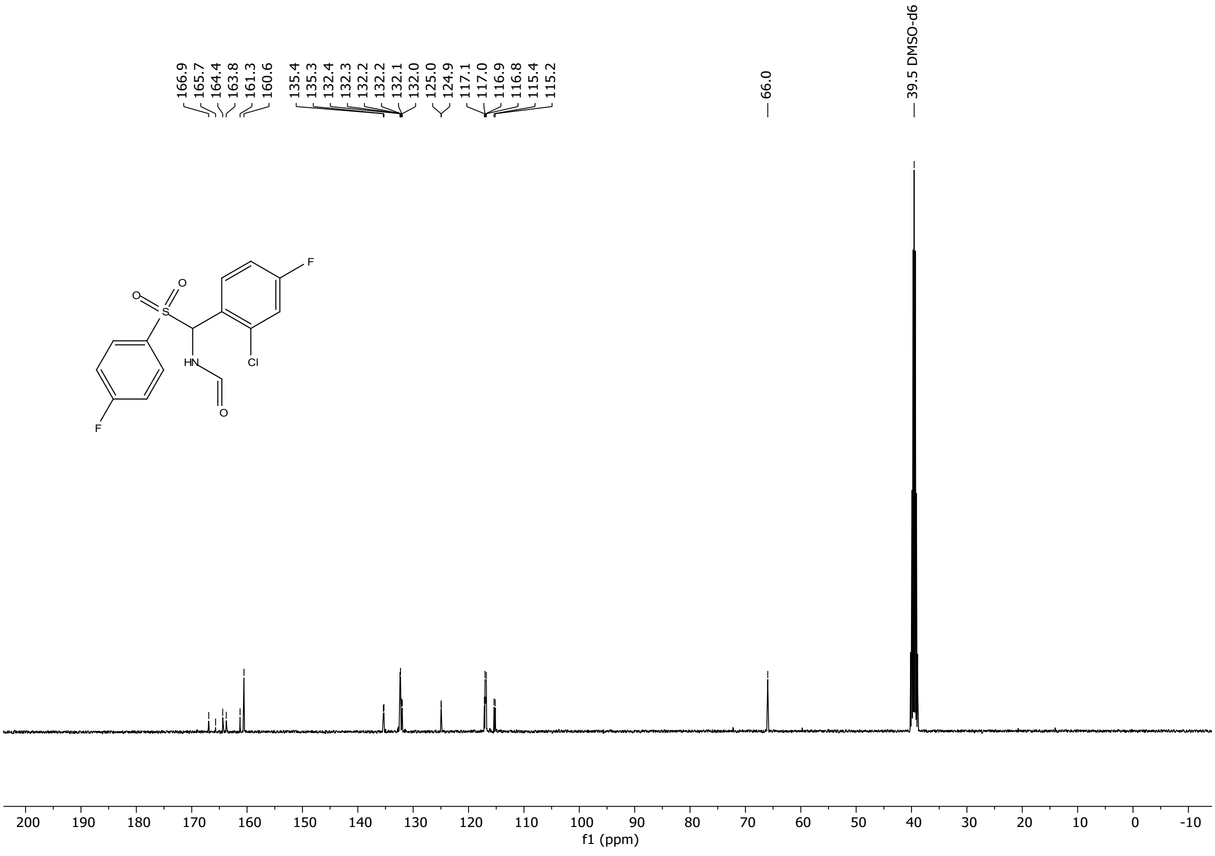

Isocyanides

2-chloro-4-fluoro-1-(isocyano(tosyl)methyl)benzene

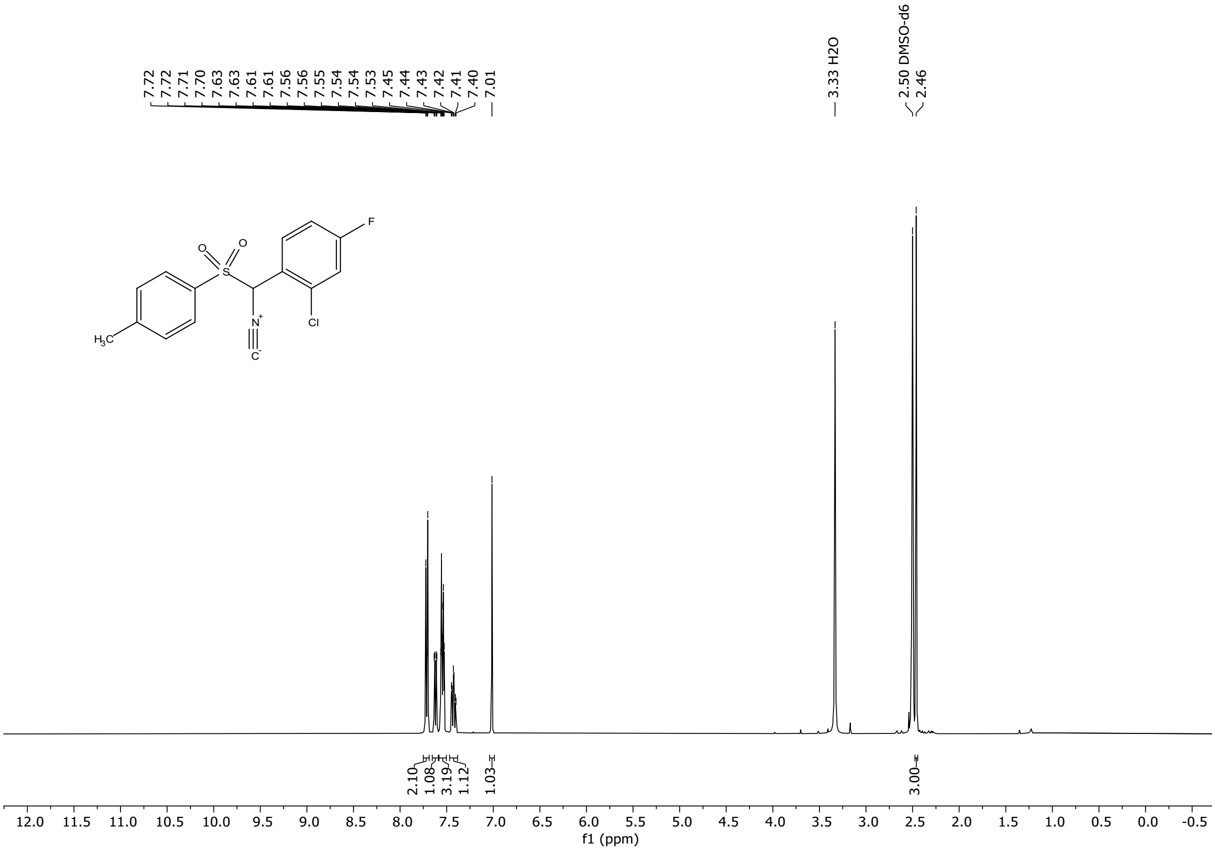

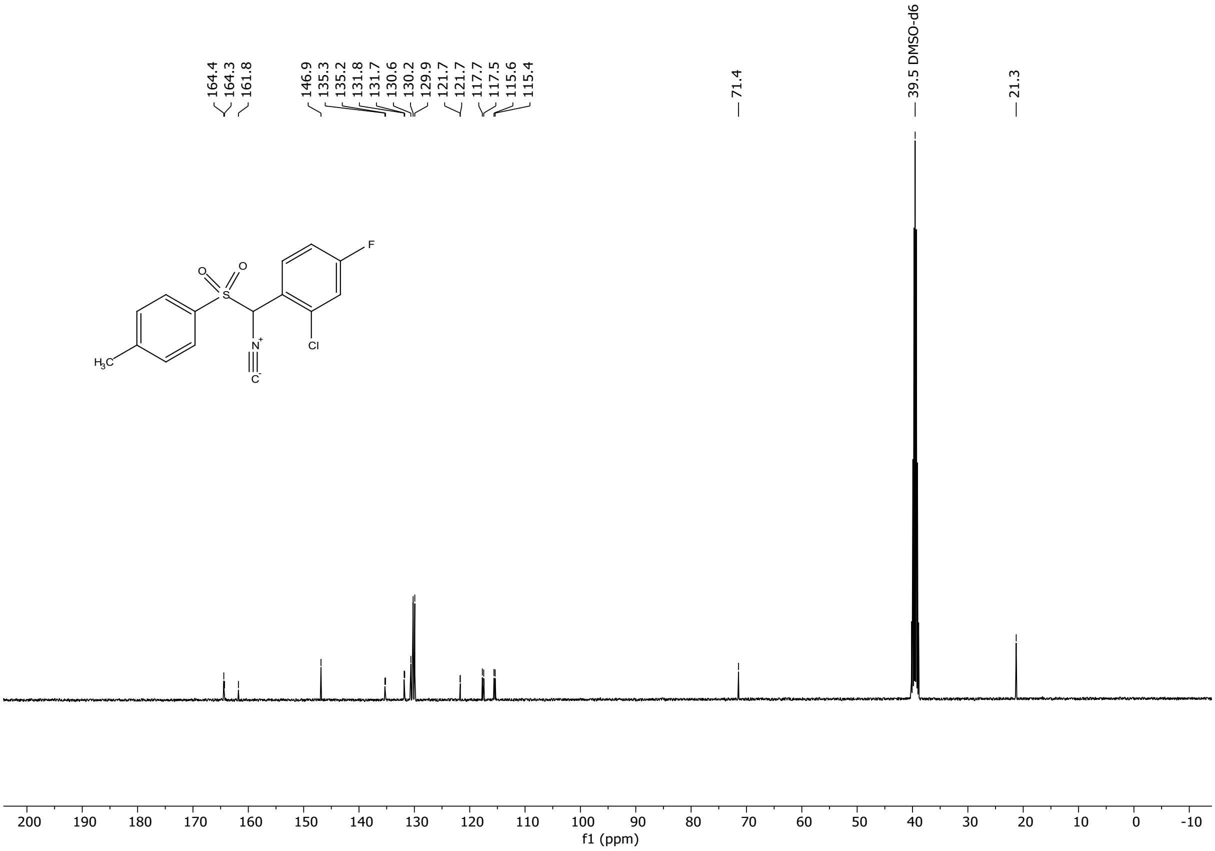

1-fluoro-4-(isocyano(tosyl)methyl)benzene

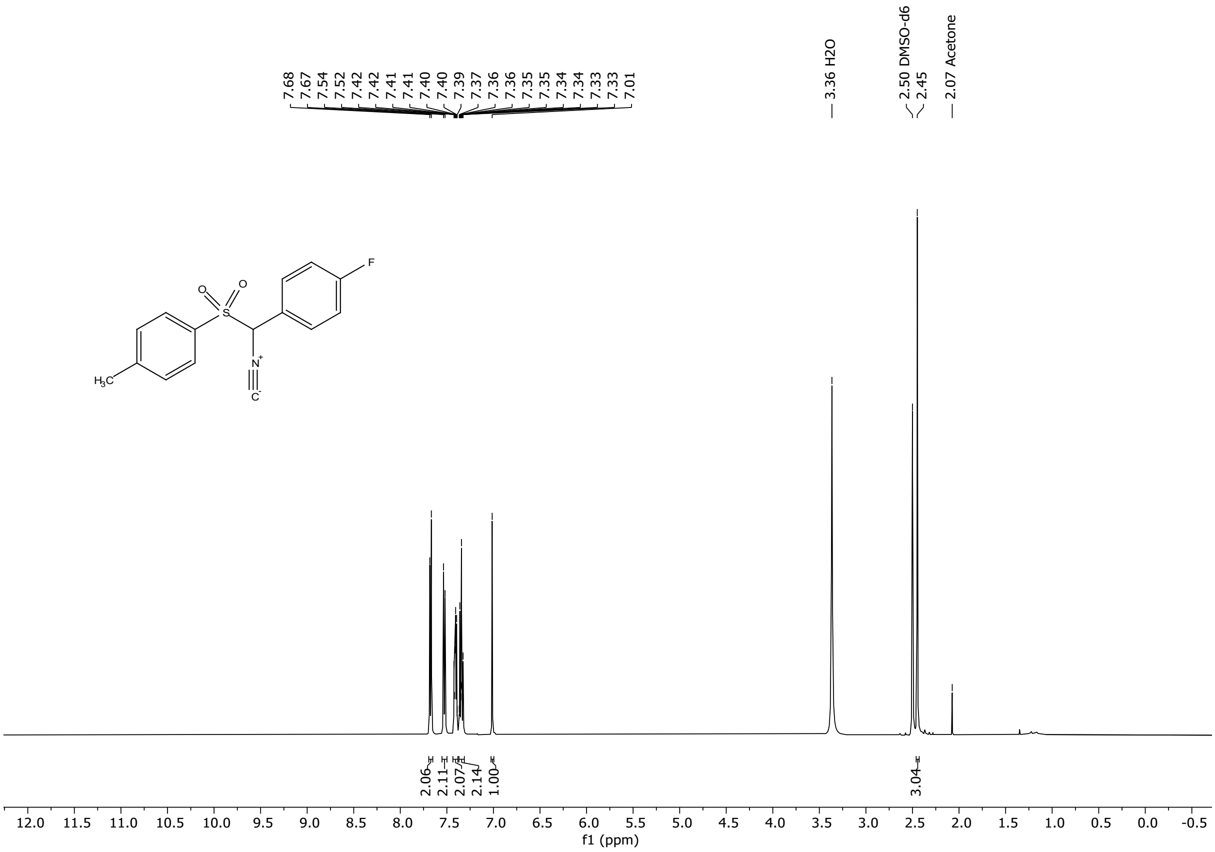

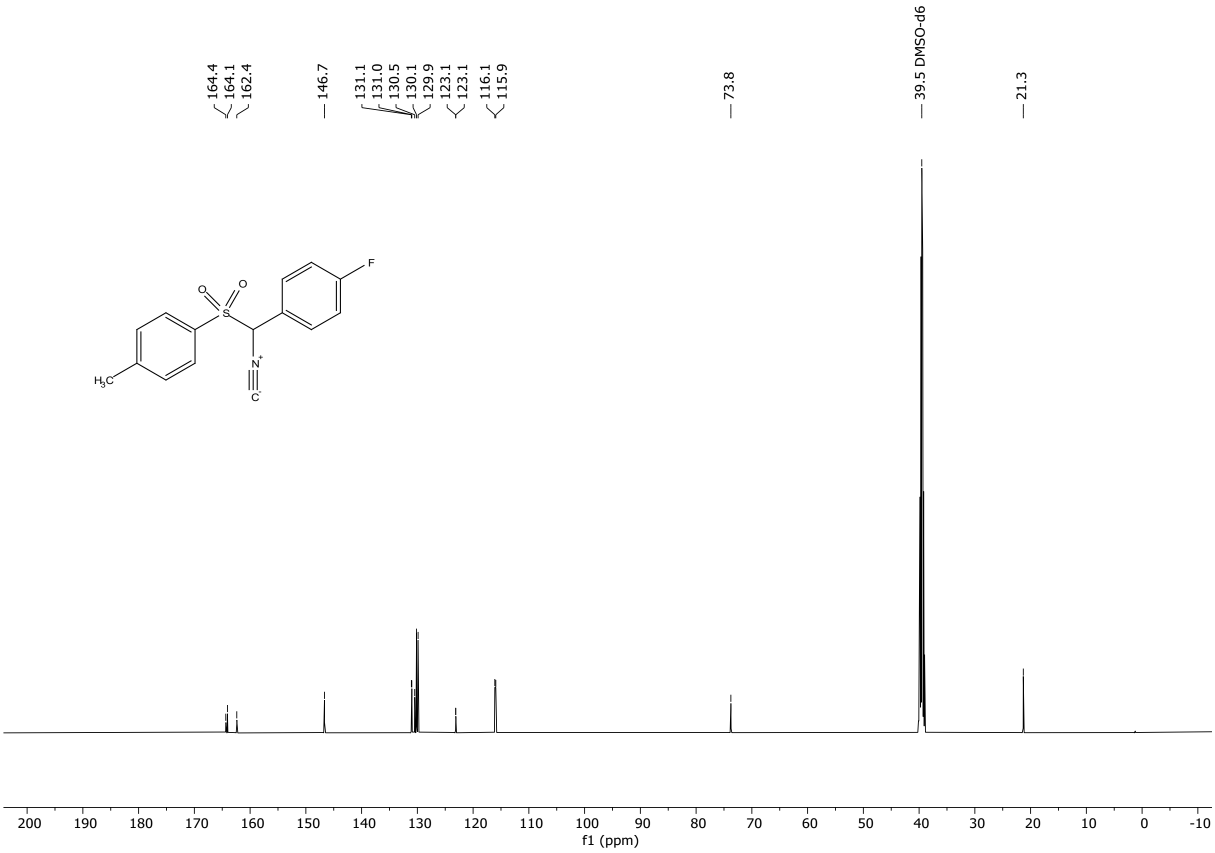

1-fluoro-3-(isocyano(tosyl)methyl)benzene

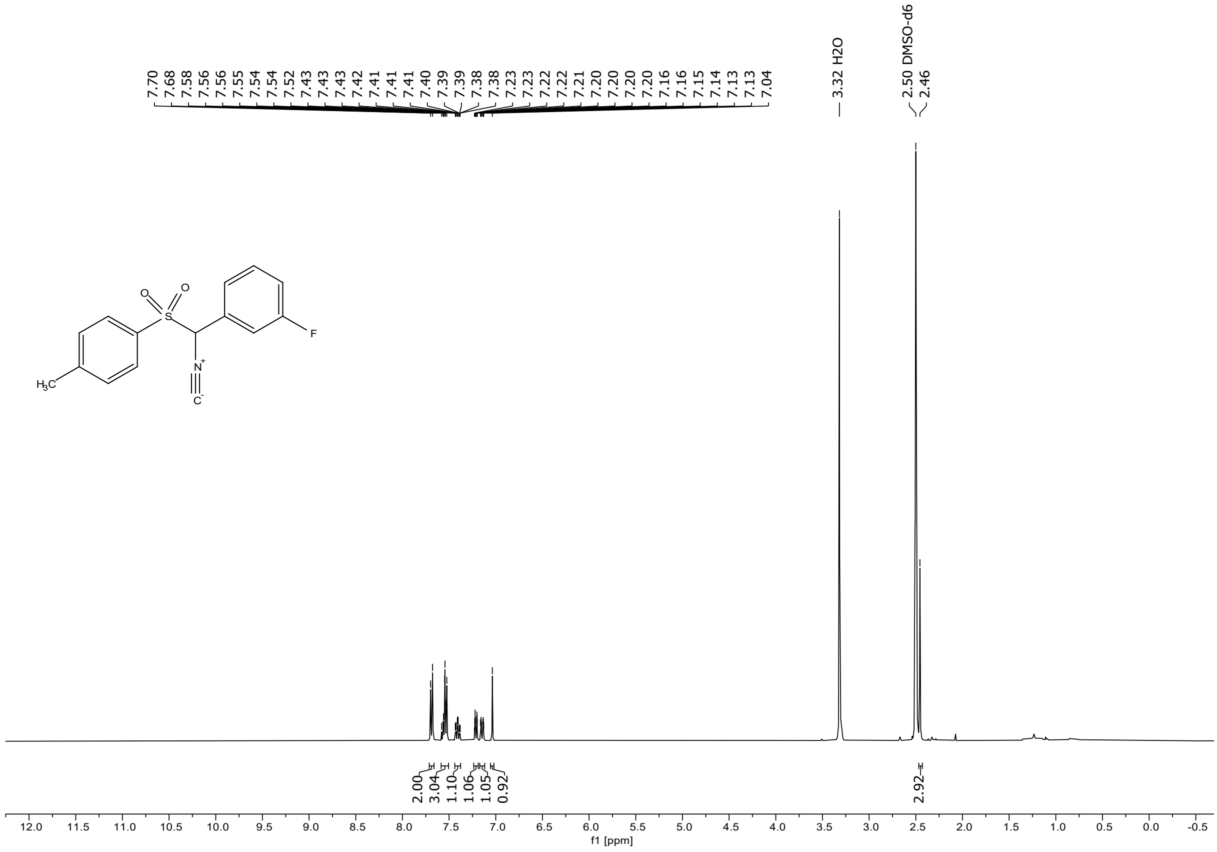

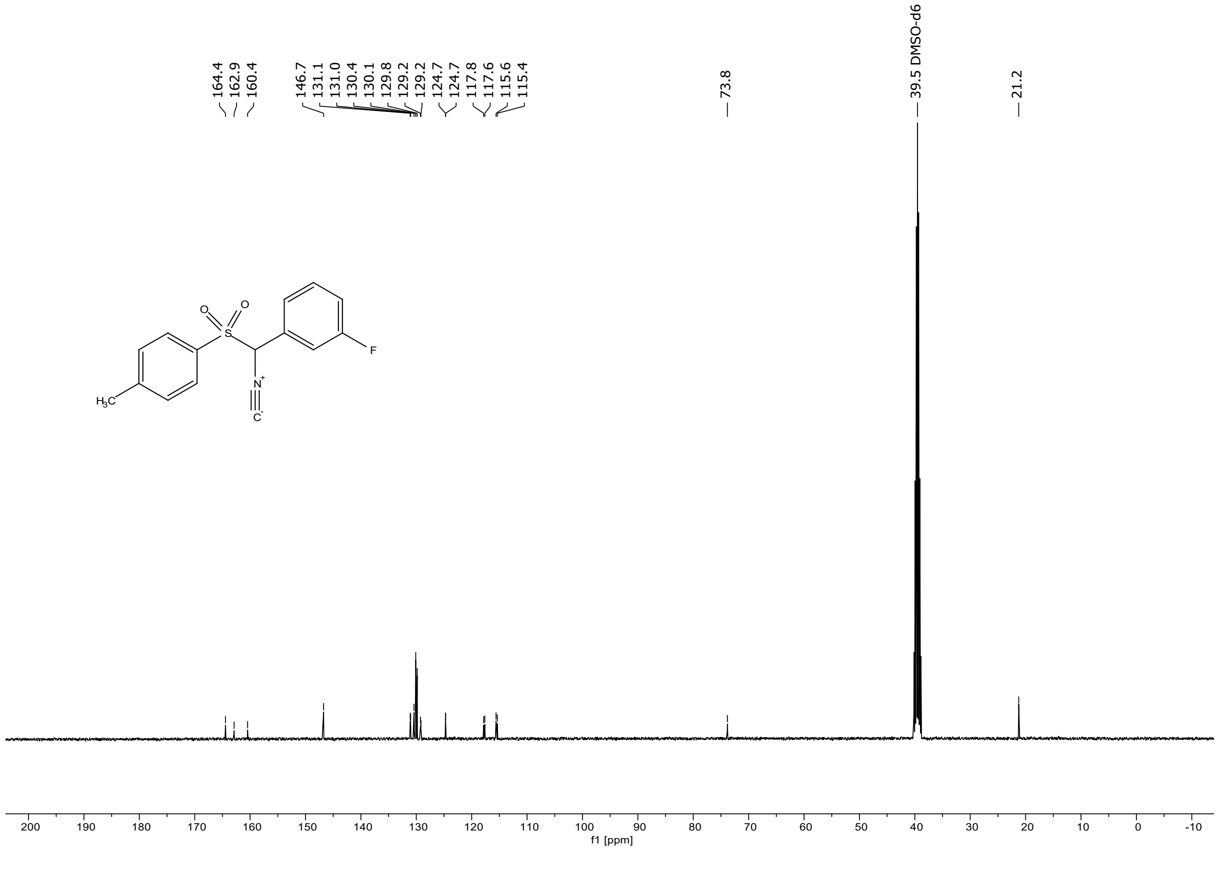

1-fluoro-2-(isocyano(tosyl)methyl)benzene

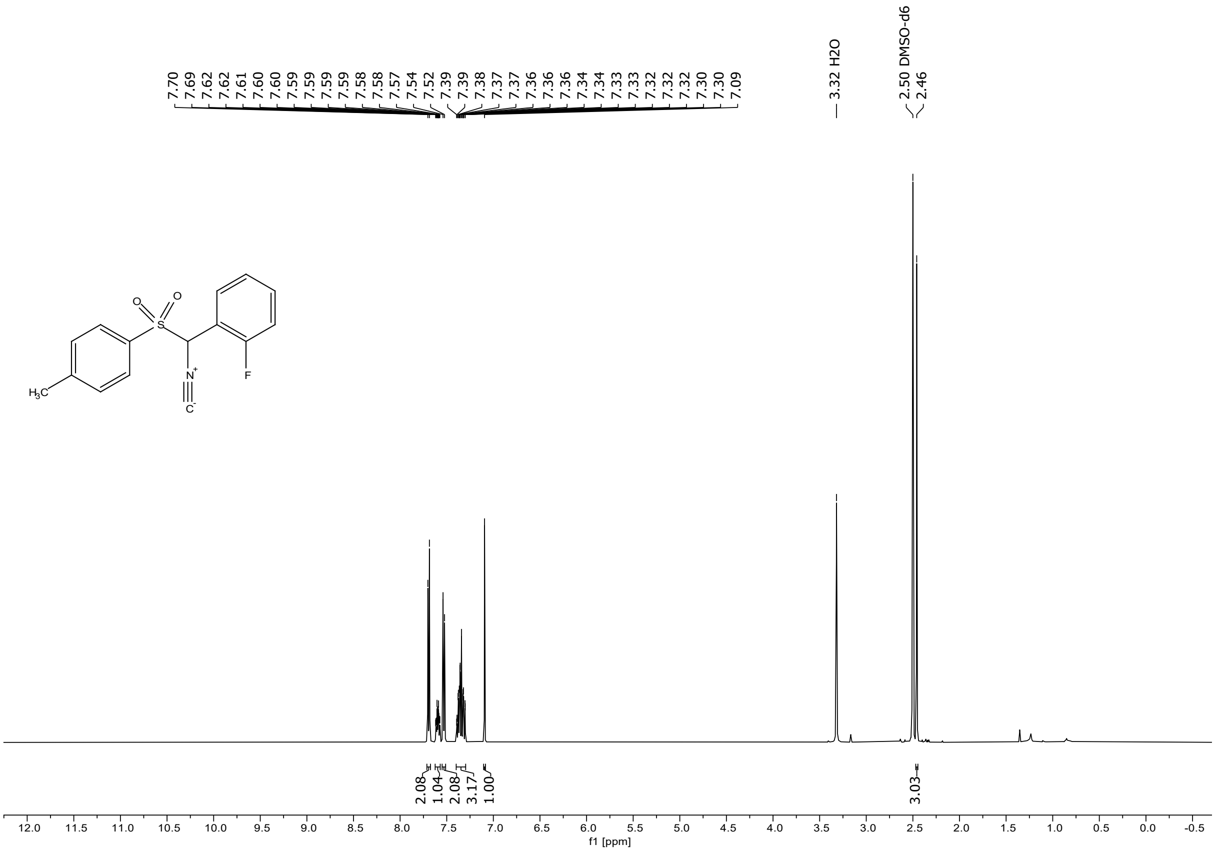

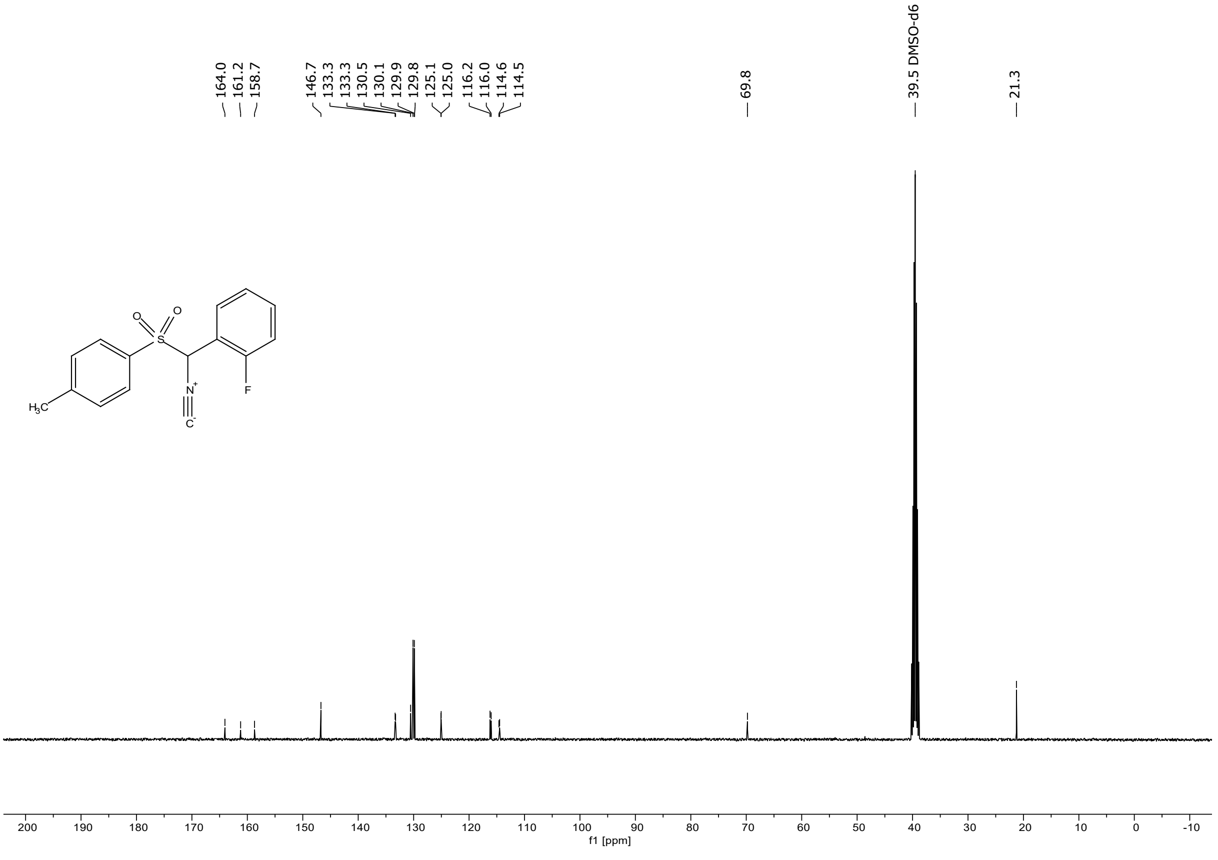

1-chloro-2-(isocyano(tosyl)methyl)benzene

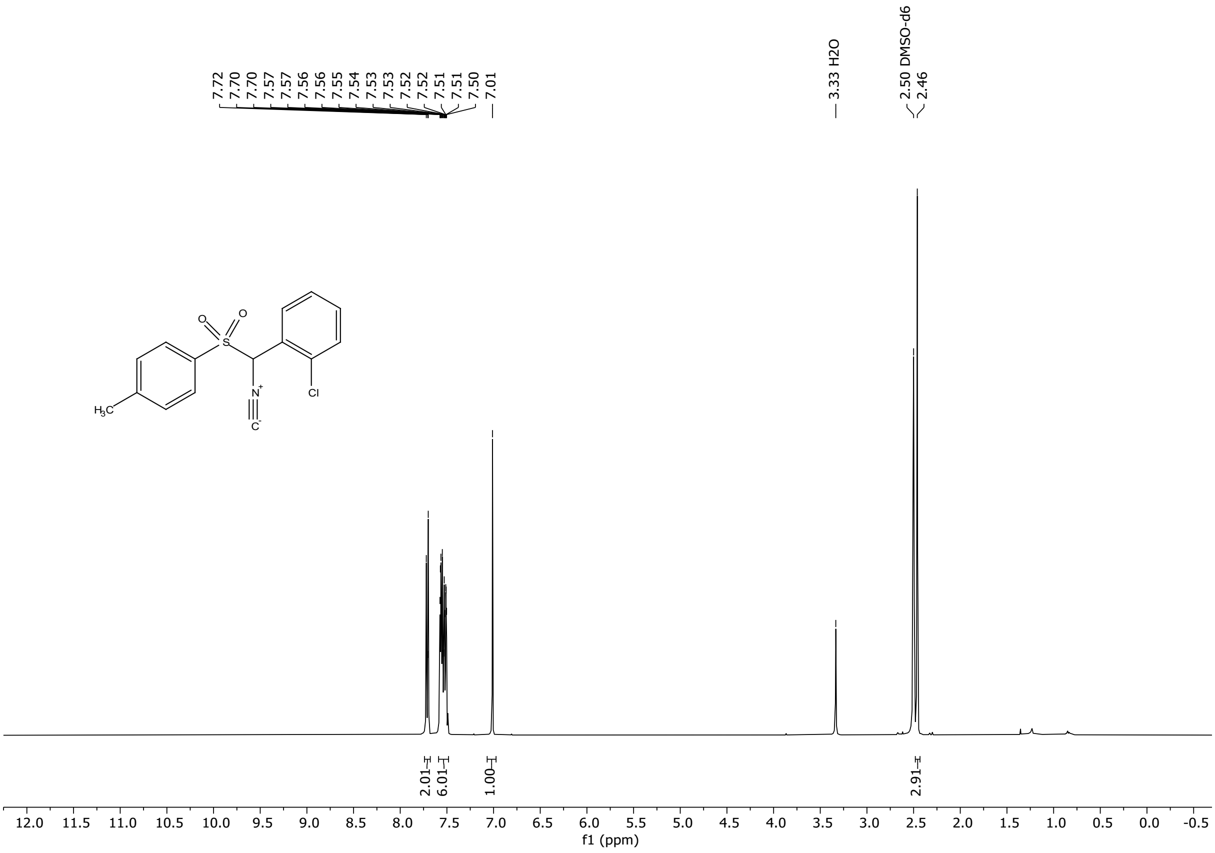

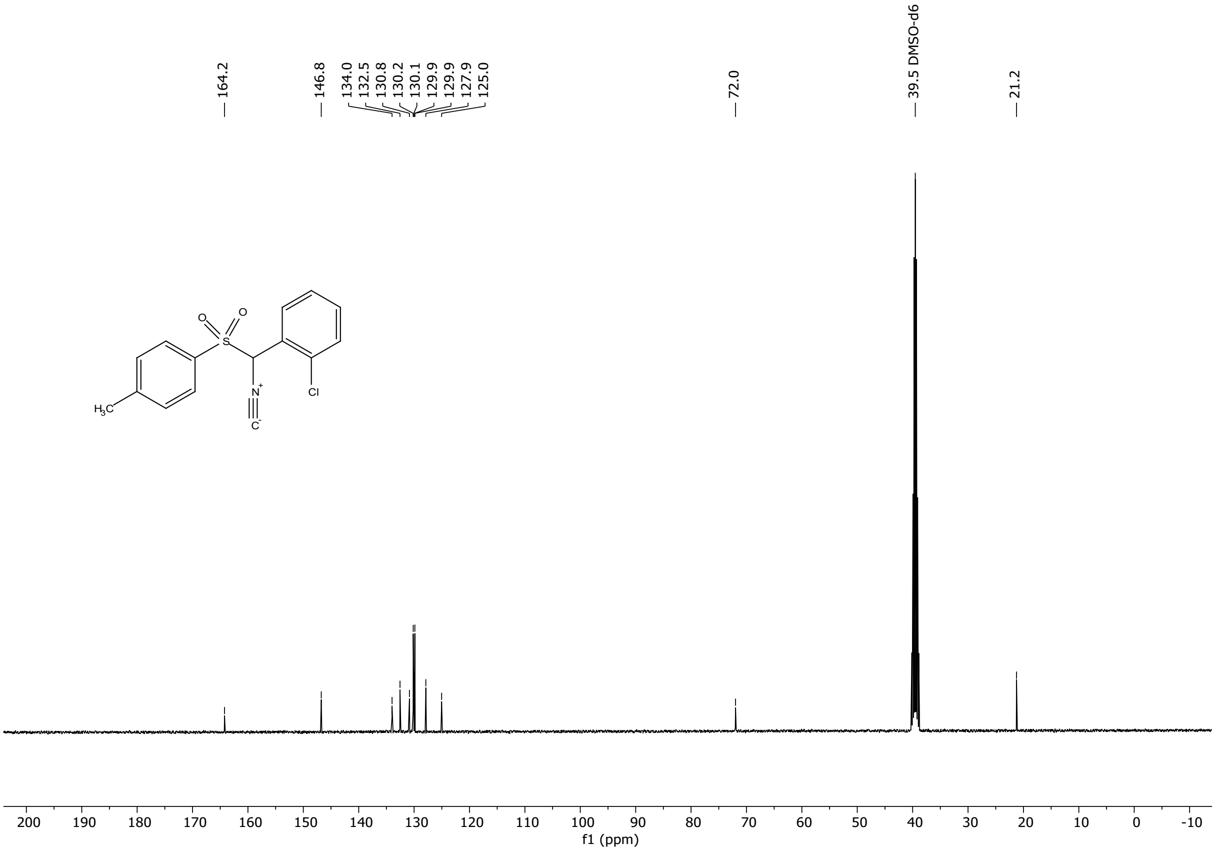

4-(isocyano(tosyl)methyl)benzonitrile

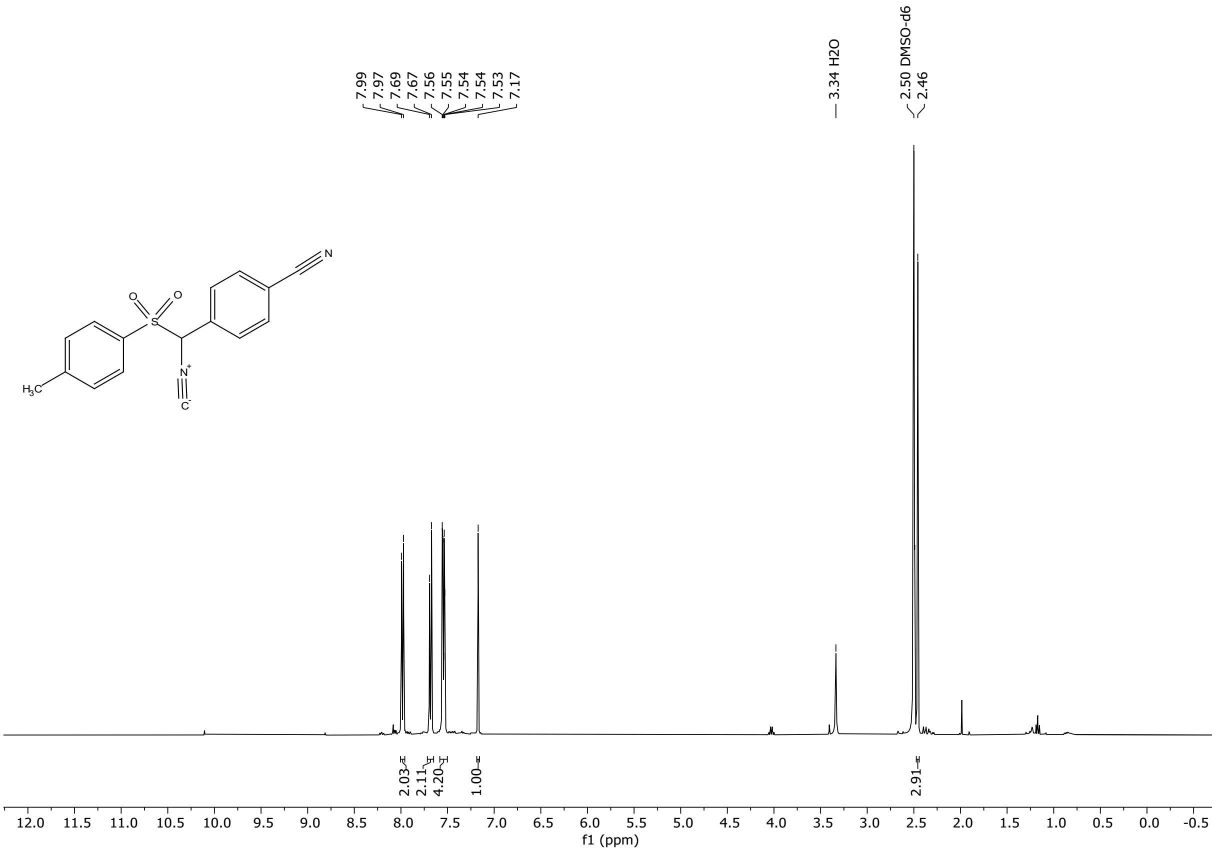

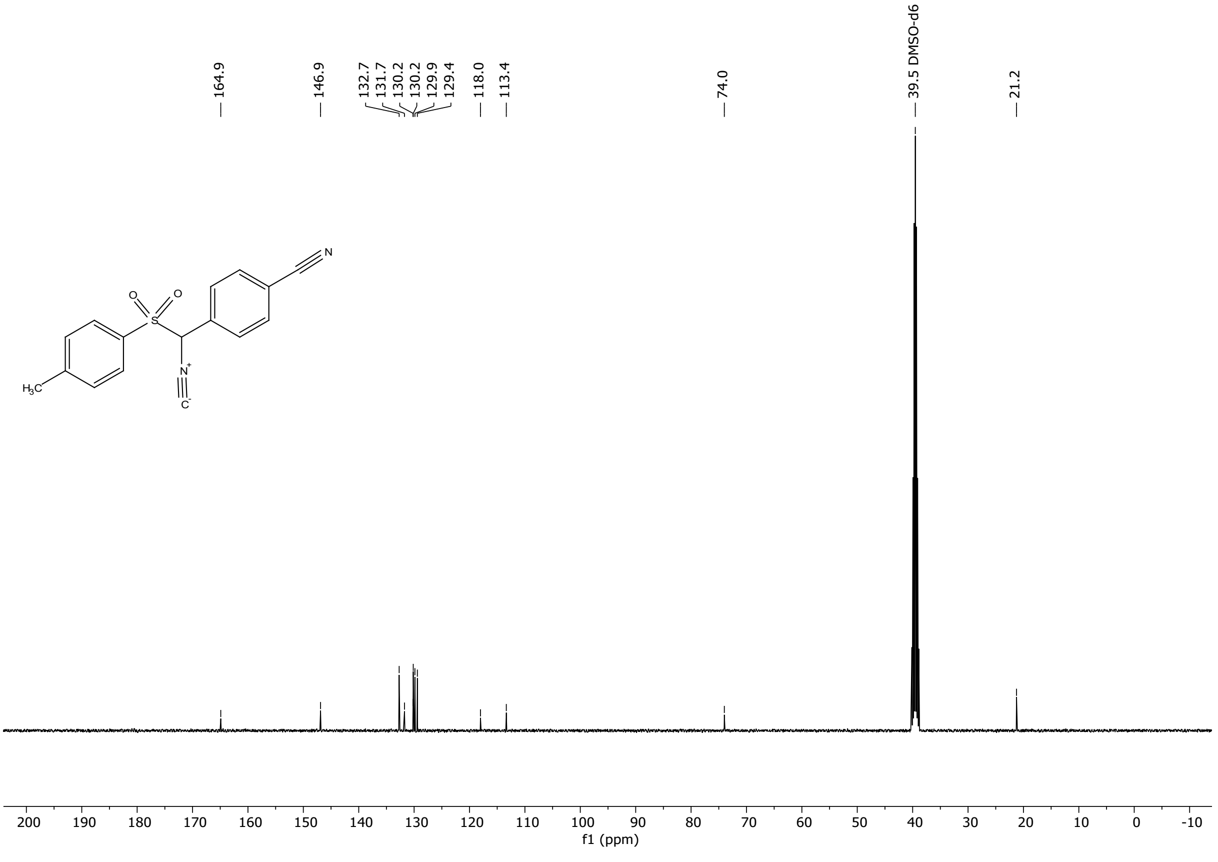

1-((isocyano(phenyl)methyl)sulfonyl)-4-methylbenzene

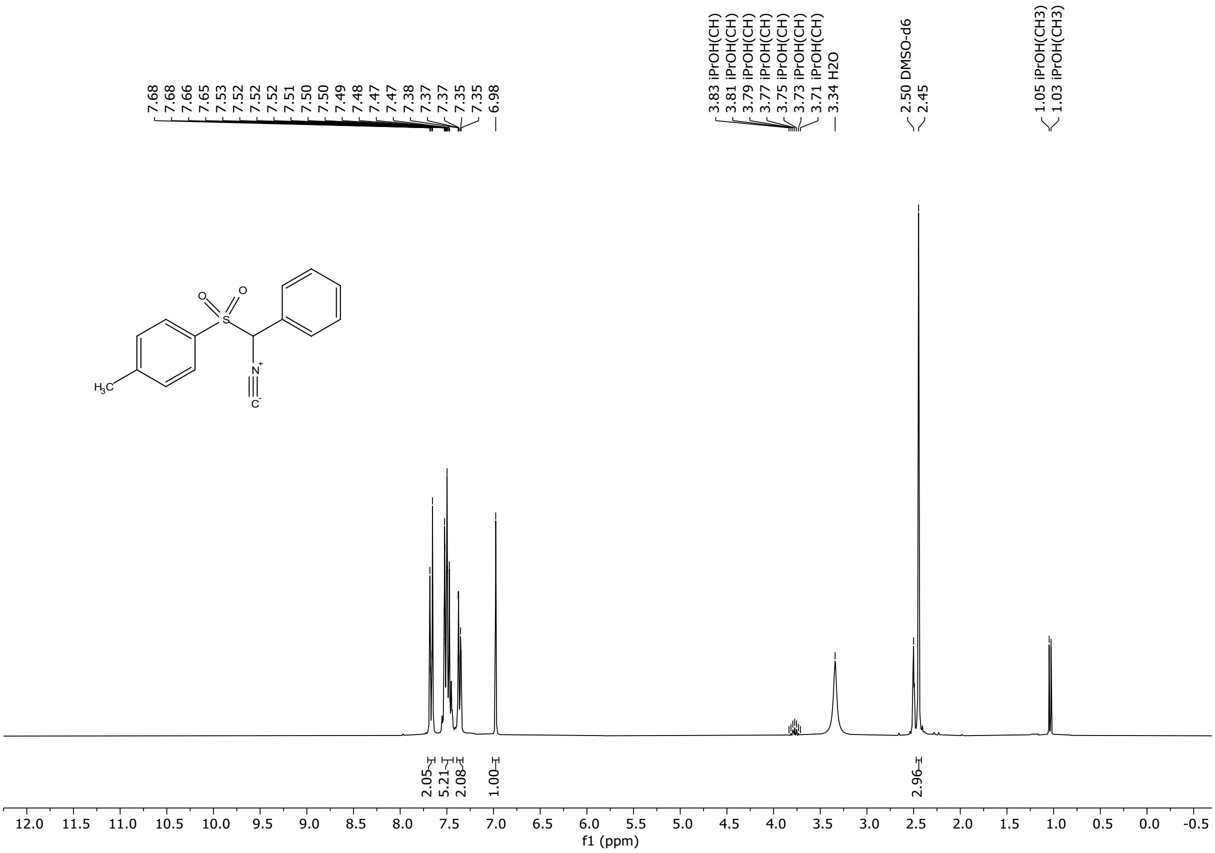

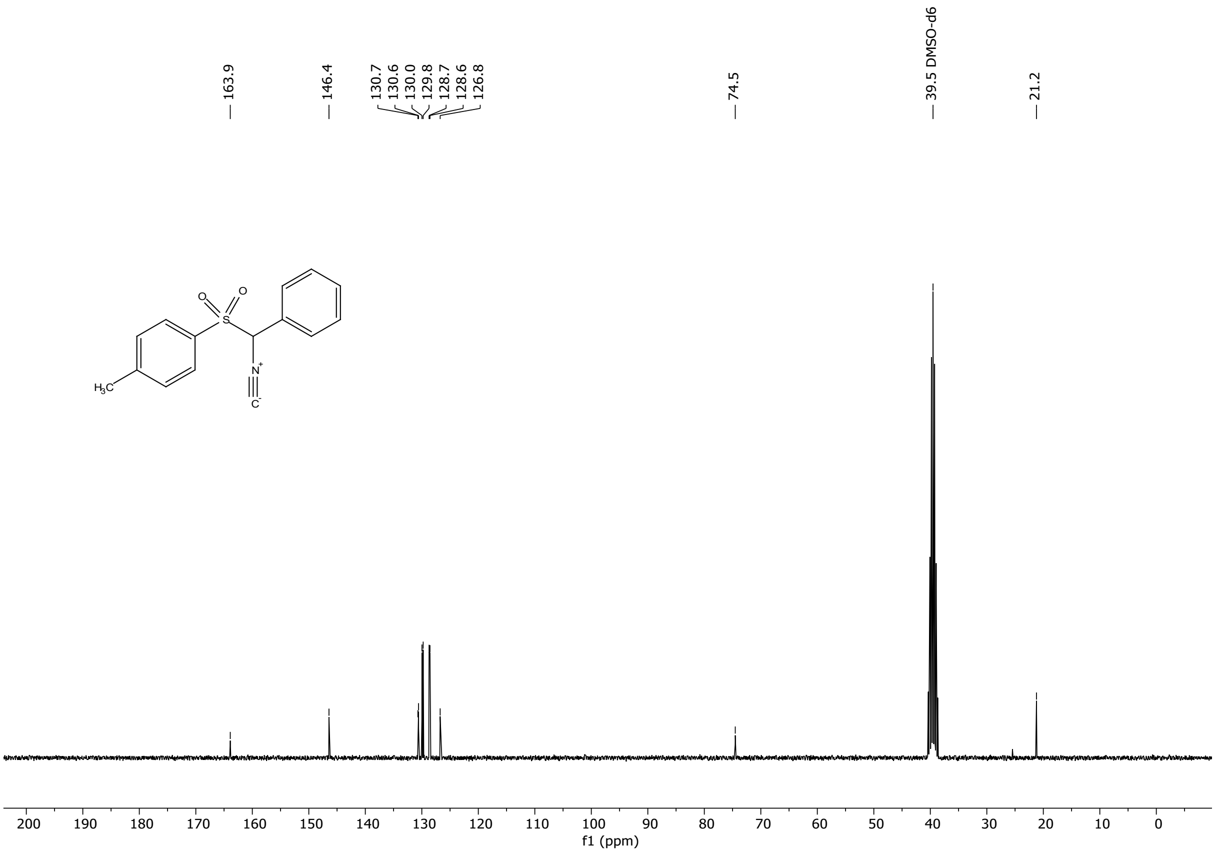

5-(isocyano(tosyl)methyl)bicyclo[2.2.1]hept-2-ene

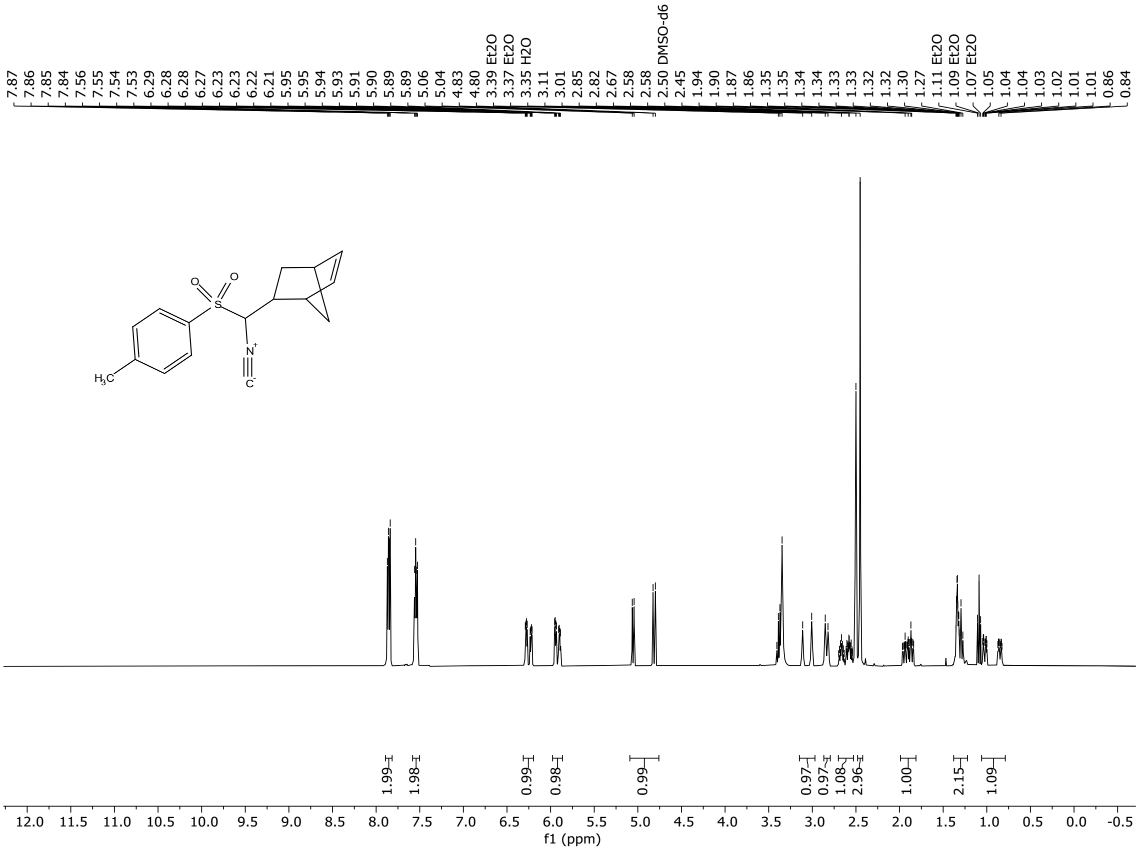

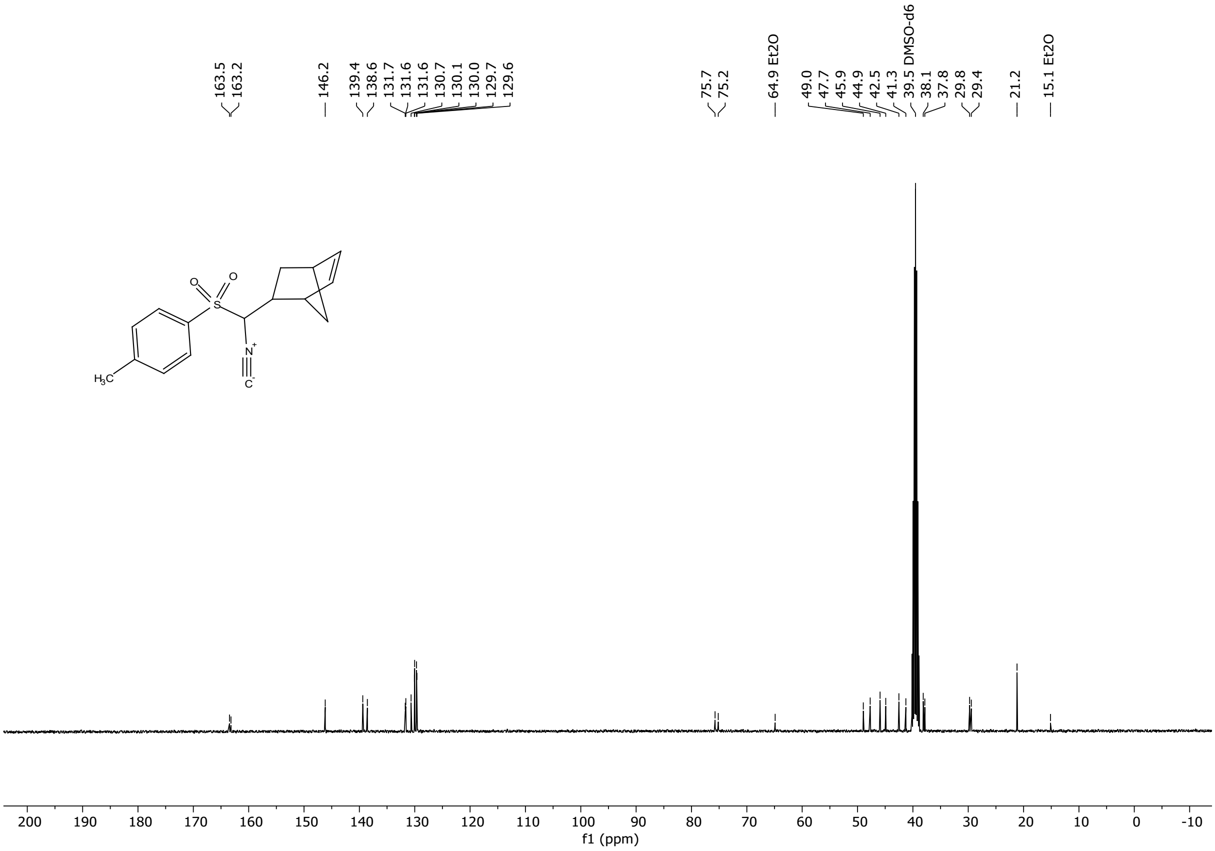

1-((1-isocyanobutyl)sulfonyl)-4-methylbenzene

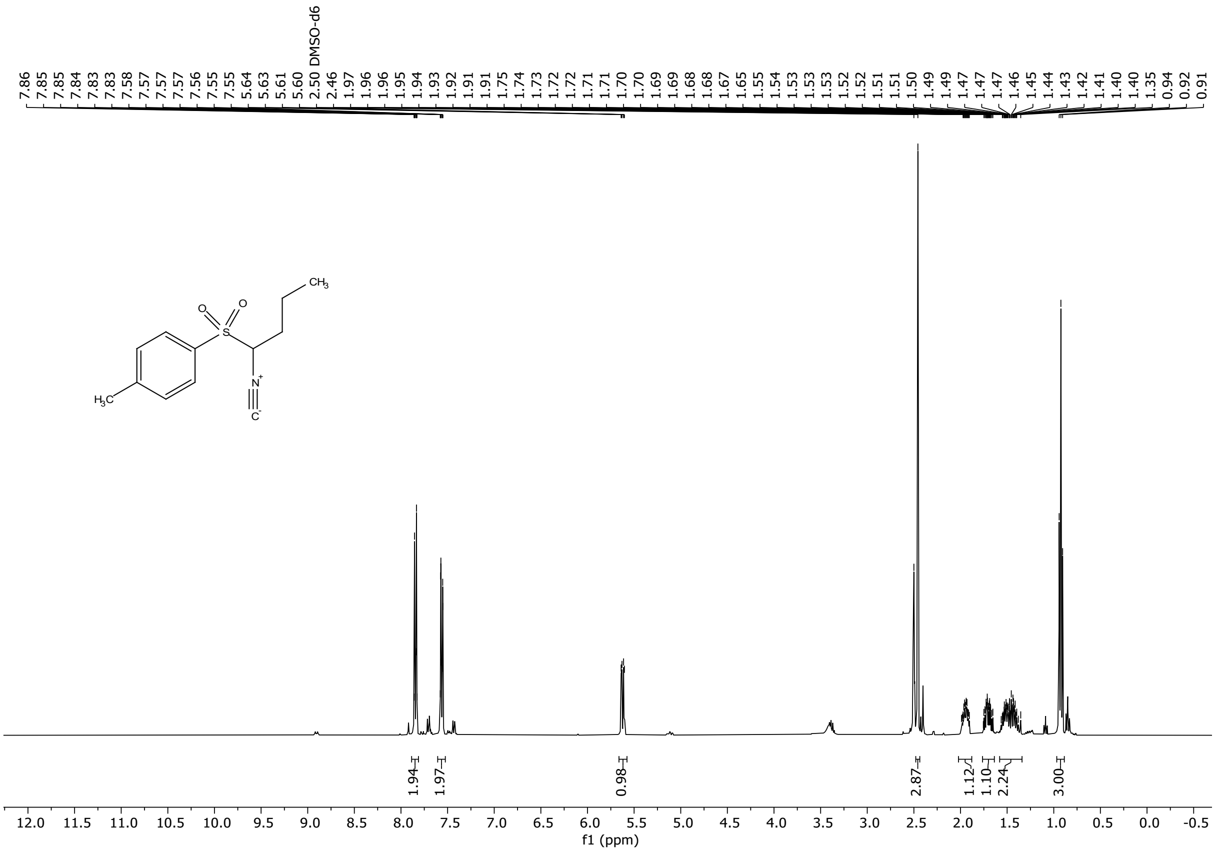

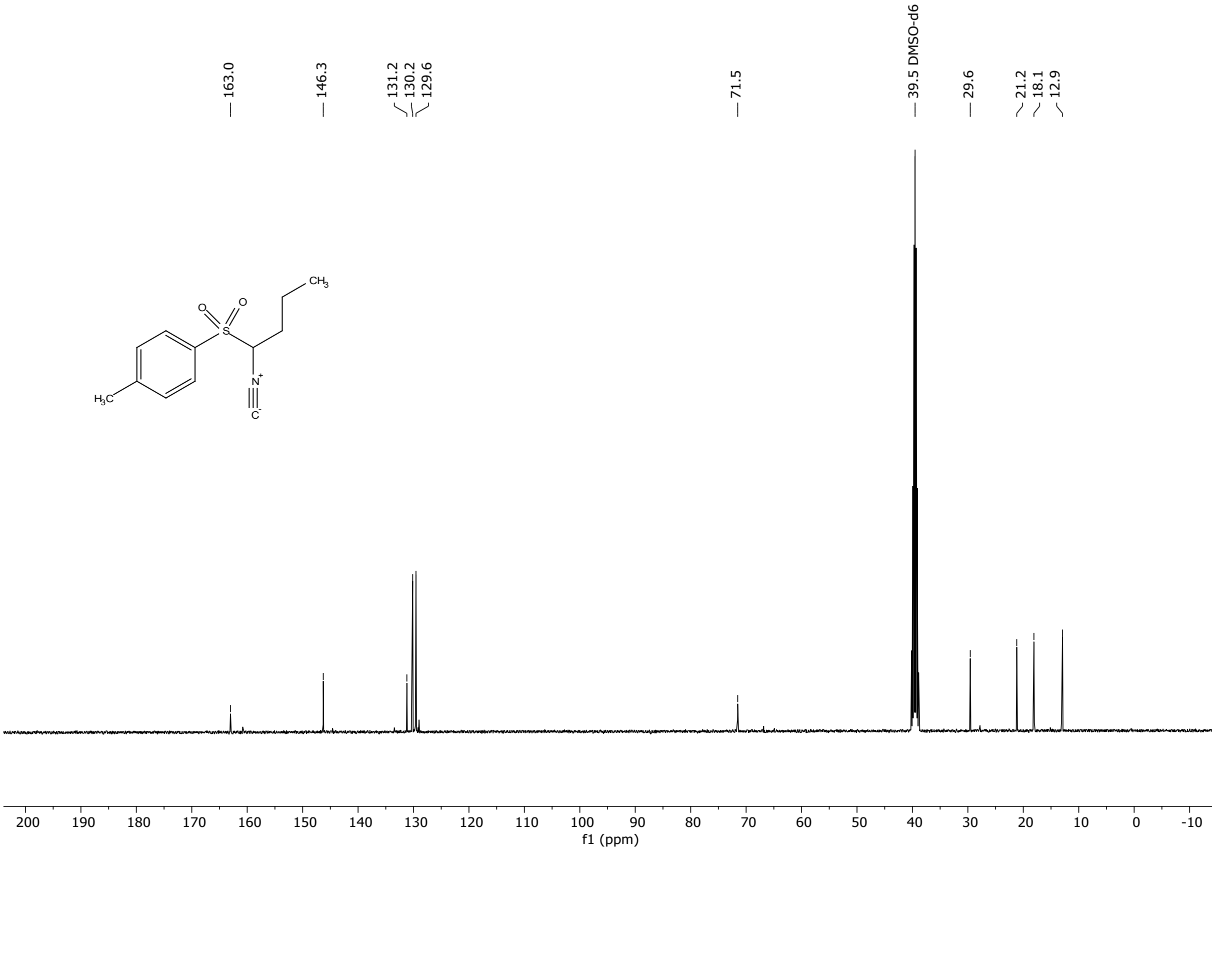

1-((butylsulfonyl)(isocyano)methyl)-2-chloro-4-fluorobenzene

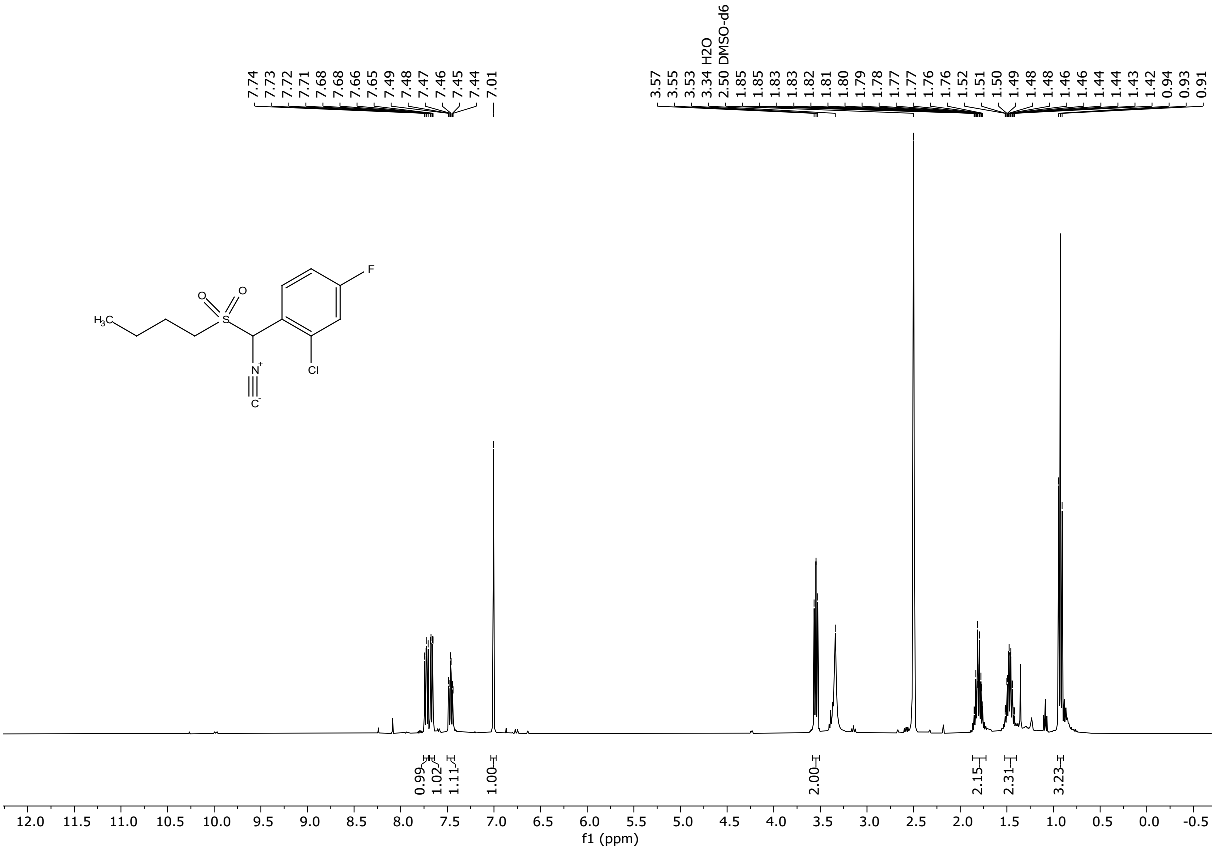

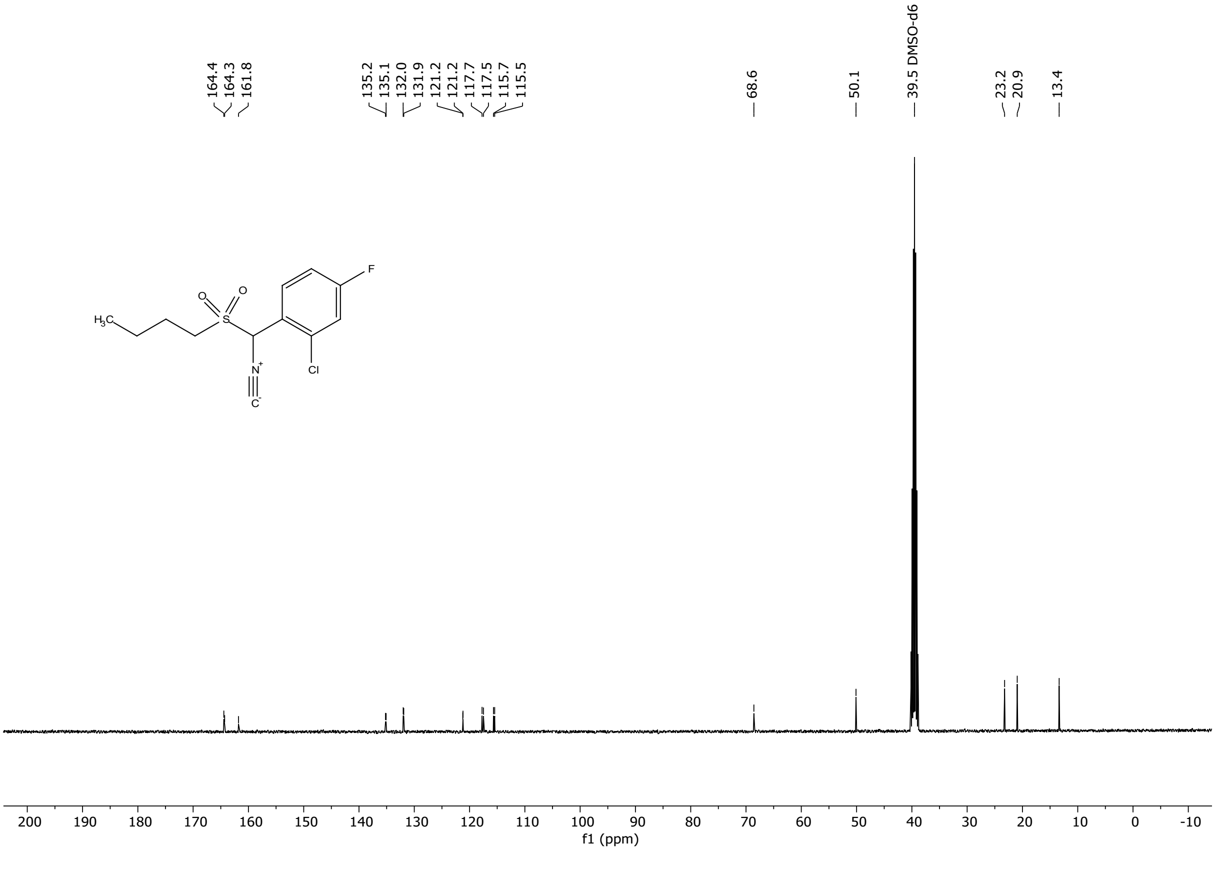

2-chloro-4-fluoro-1-(isocyano(methylsulfonyl)methyl)benzene

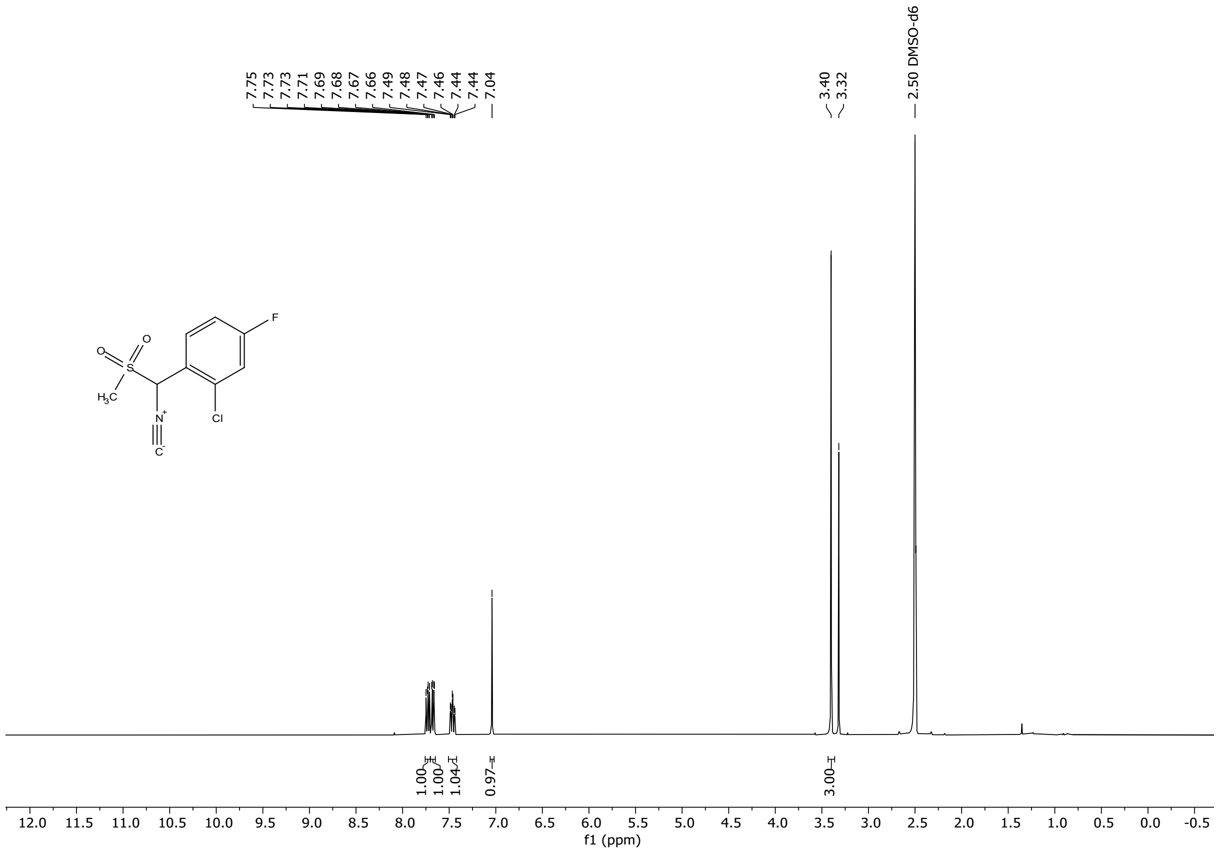

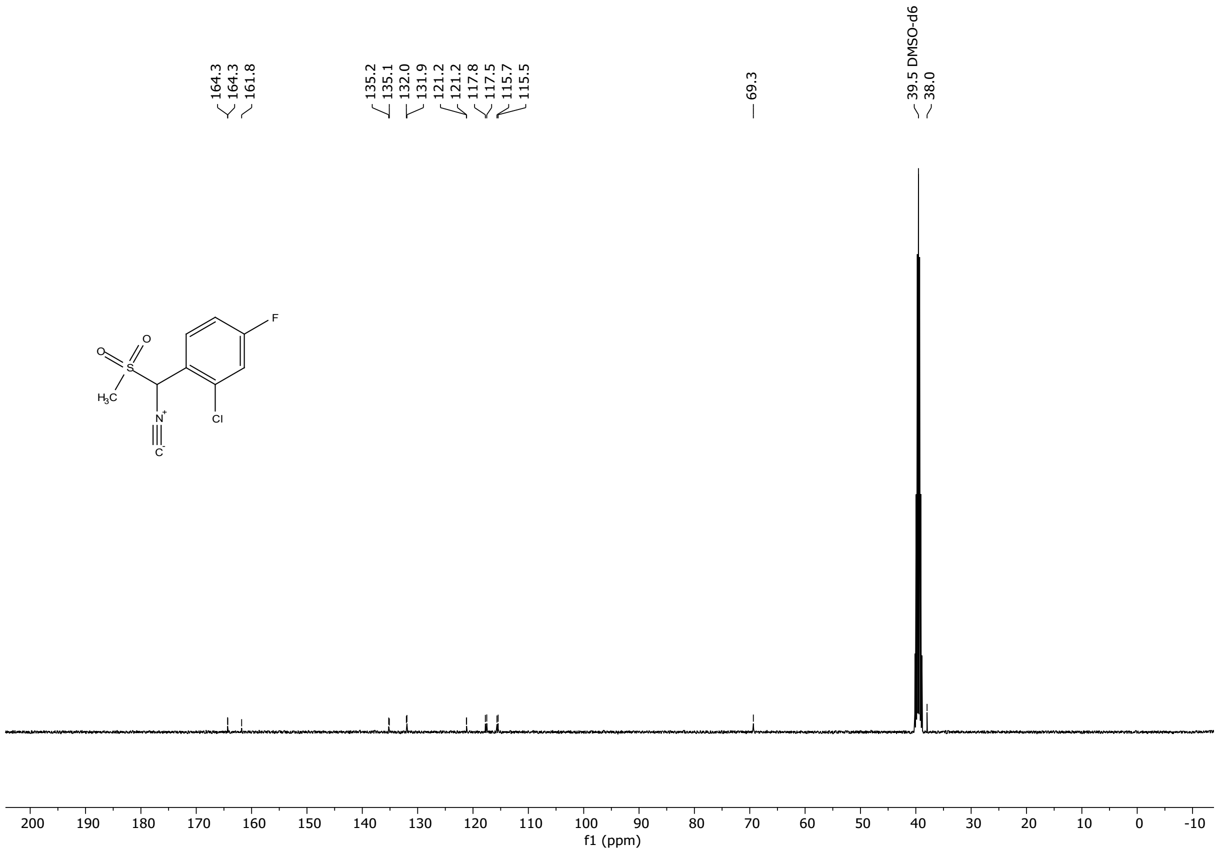

2-chloro-4-fluoro-1-(isocyano(phenylsulfonyl)methyl)benzene

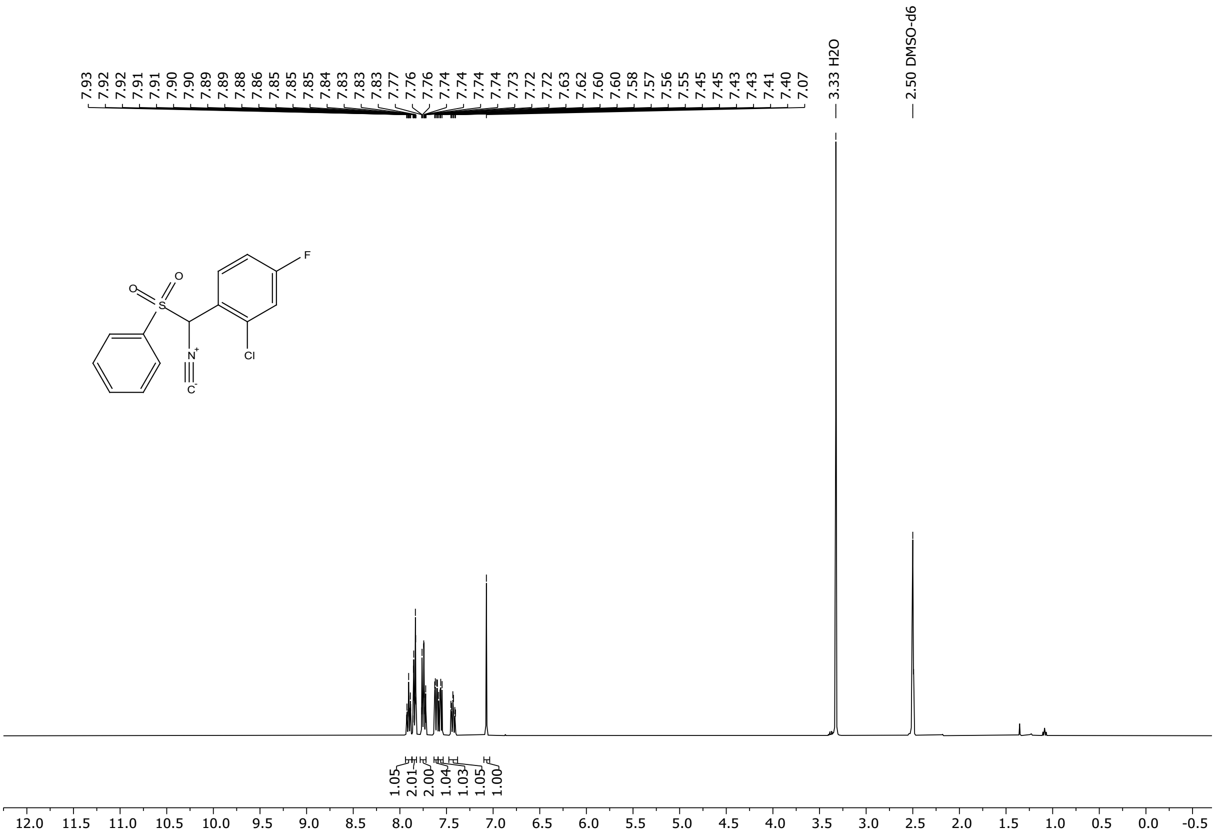

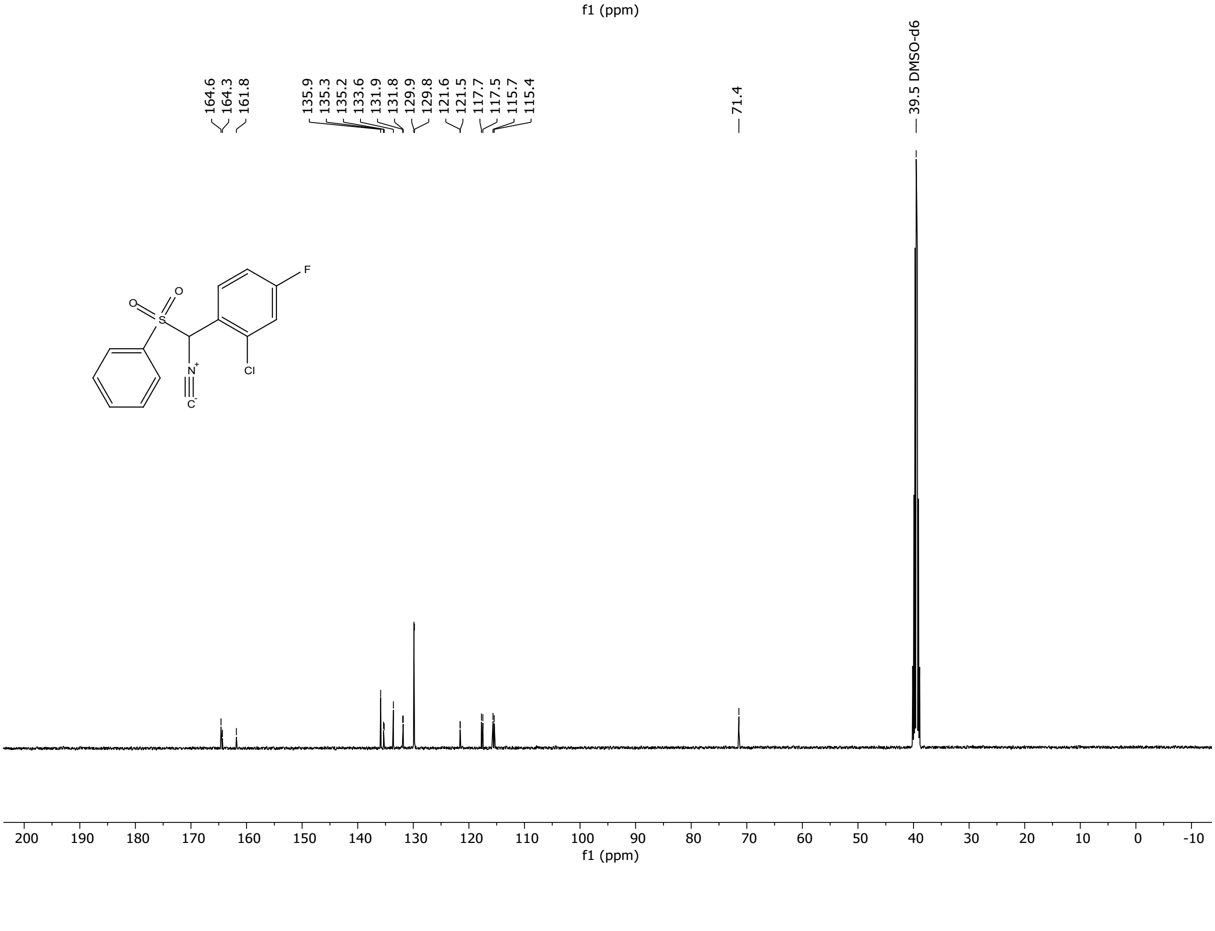

2-chloro-4-fluoro-1-(isocyano((4-methoxyphenyl)sulfonyl)methyl)benzene

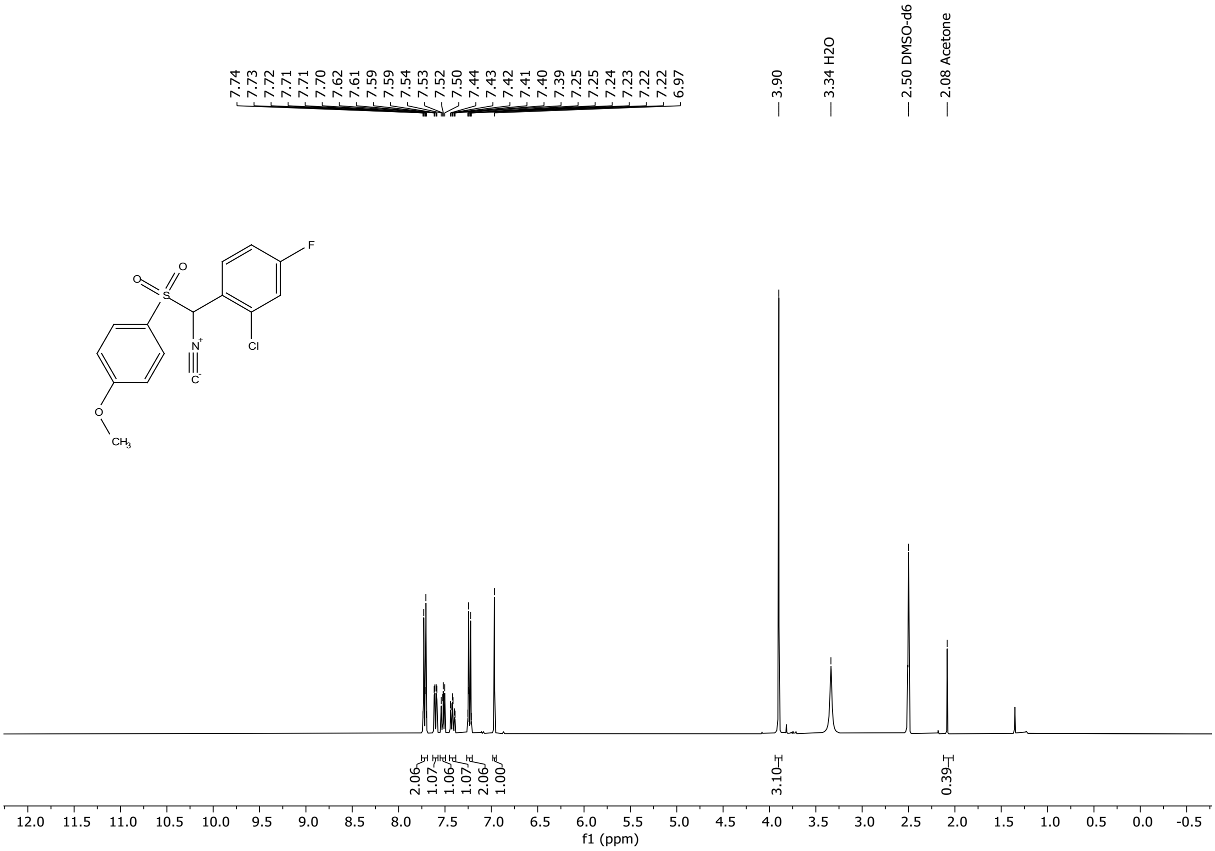

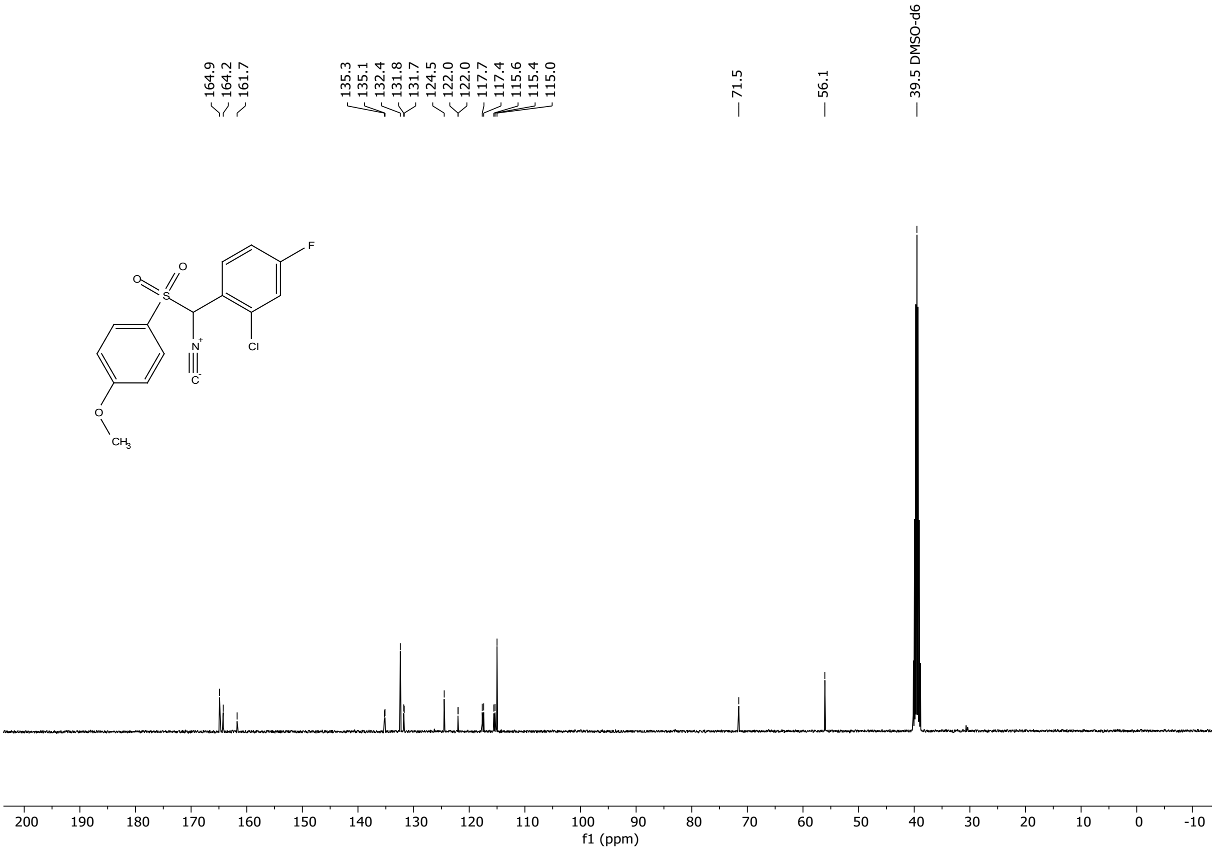

2-chloro-4-fluoro-1-(((4-fluorophenyl)sulfonyl)(isocyano)methyl)benzene

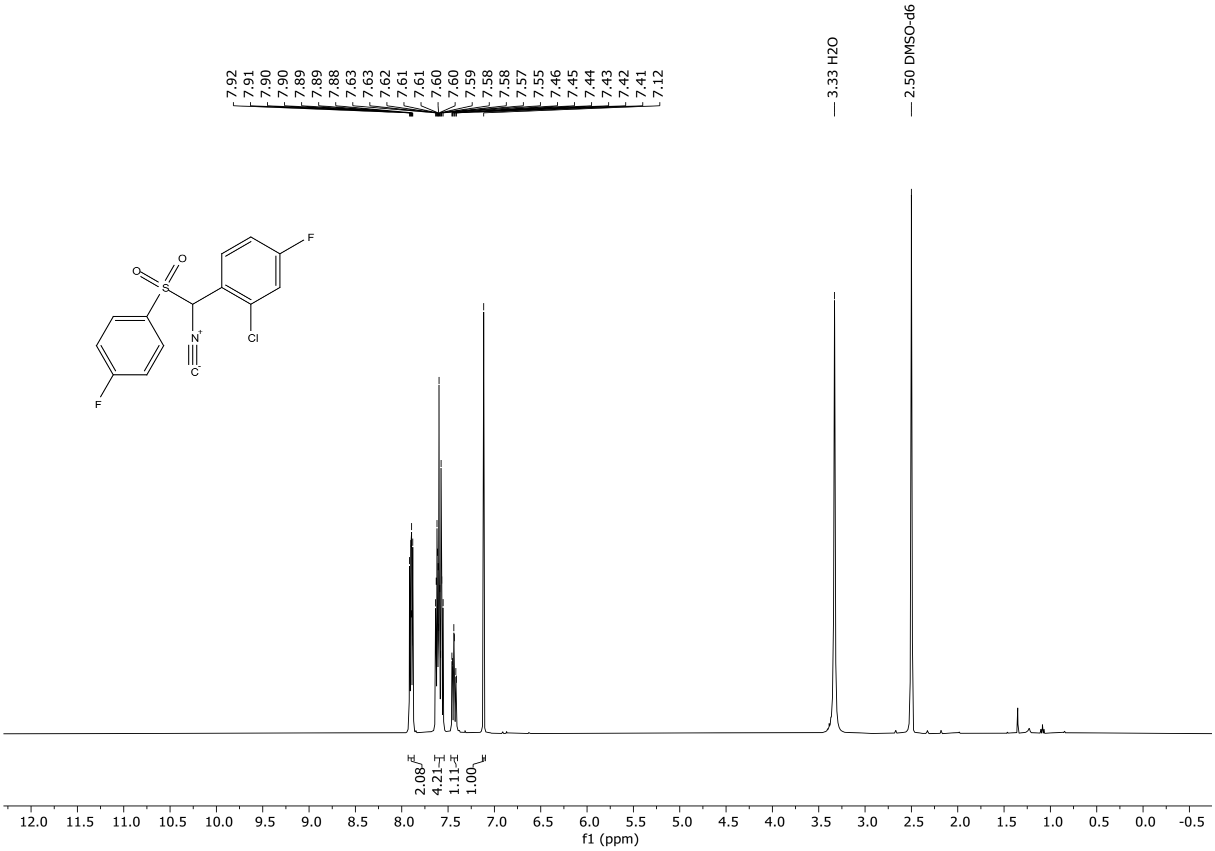

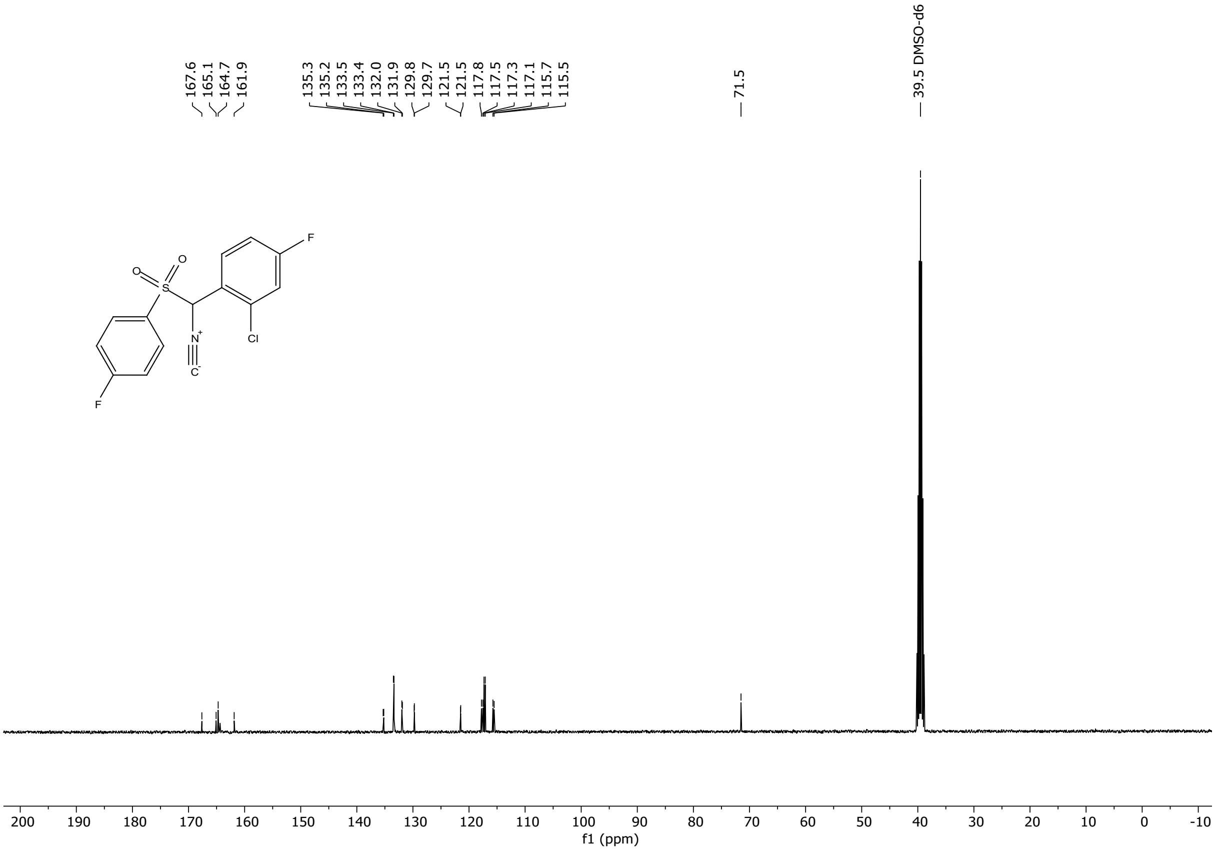

Benzyl aryl sulfones

1-(benzylsulfonyl)-4-methylbenzene

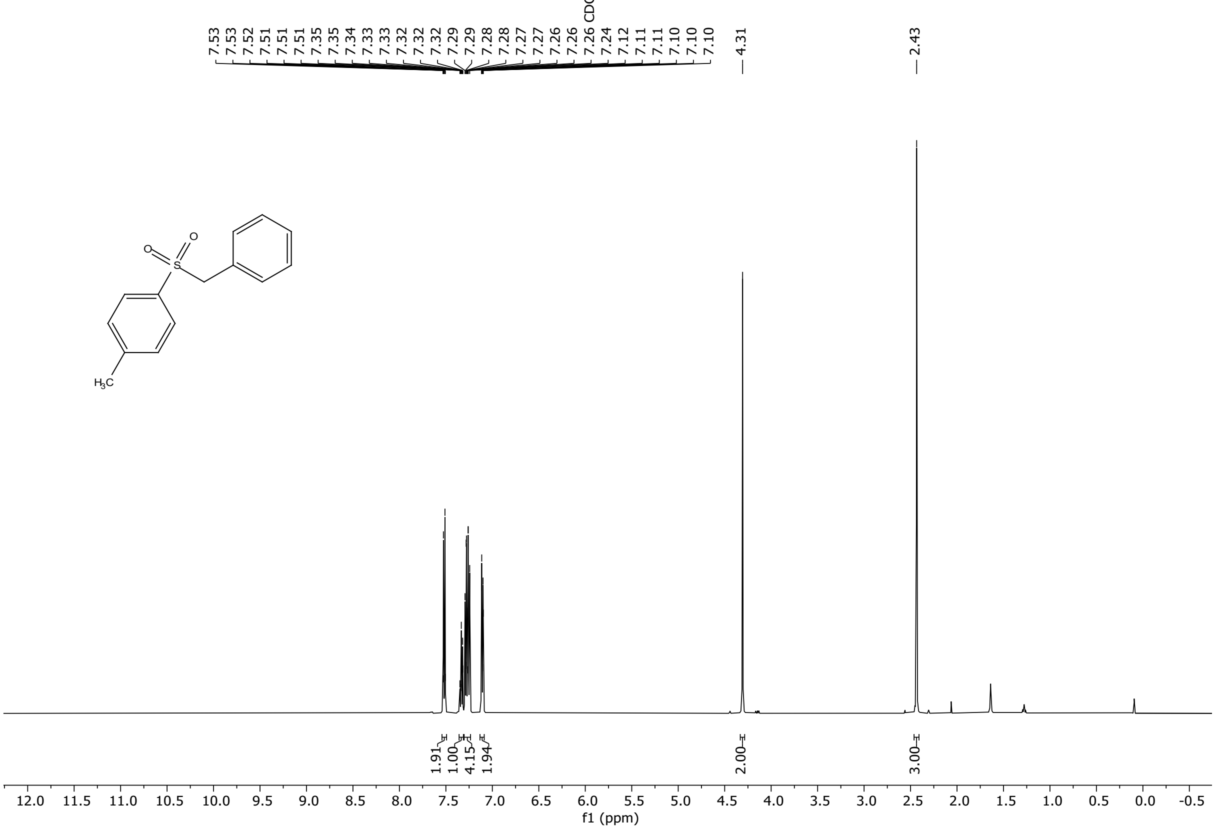

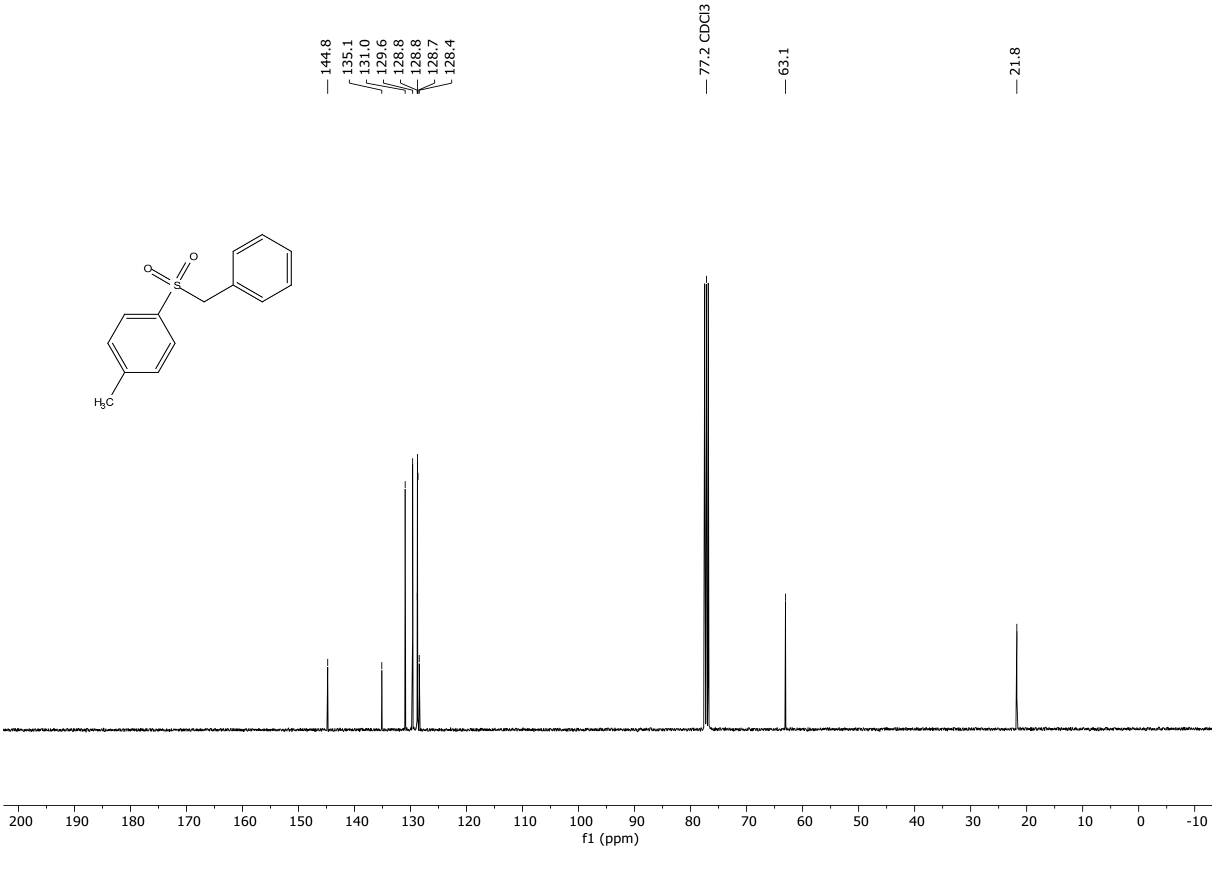

1-fluoro-4-(tosylmethyl)benzene

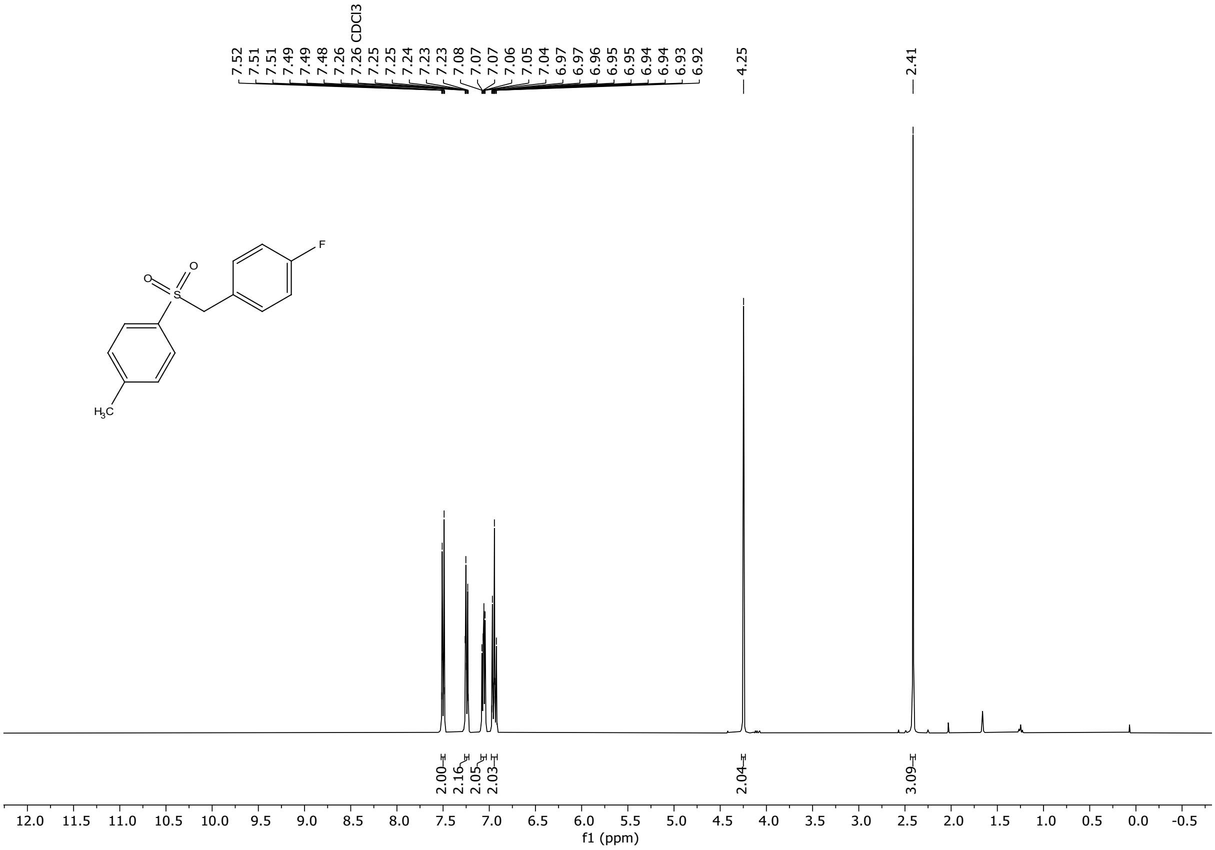

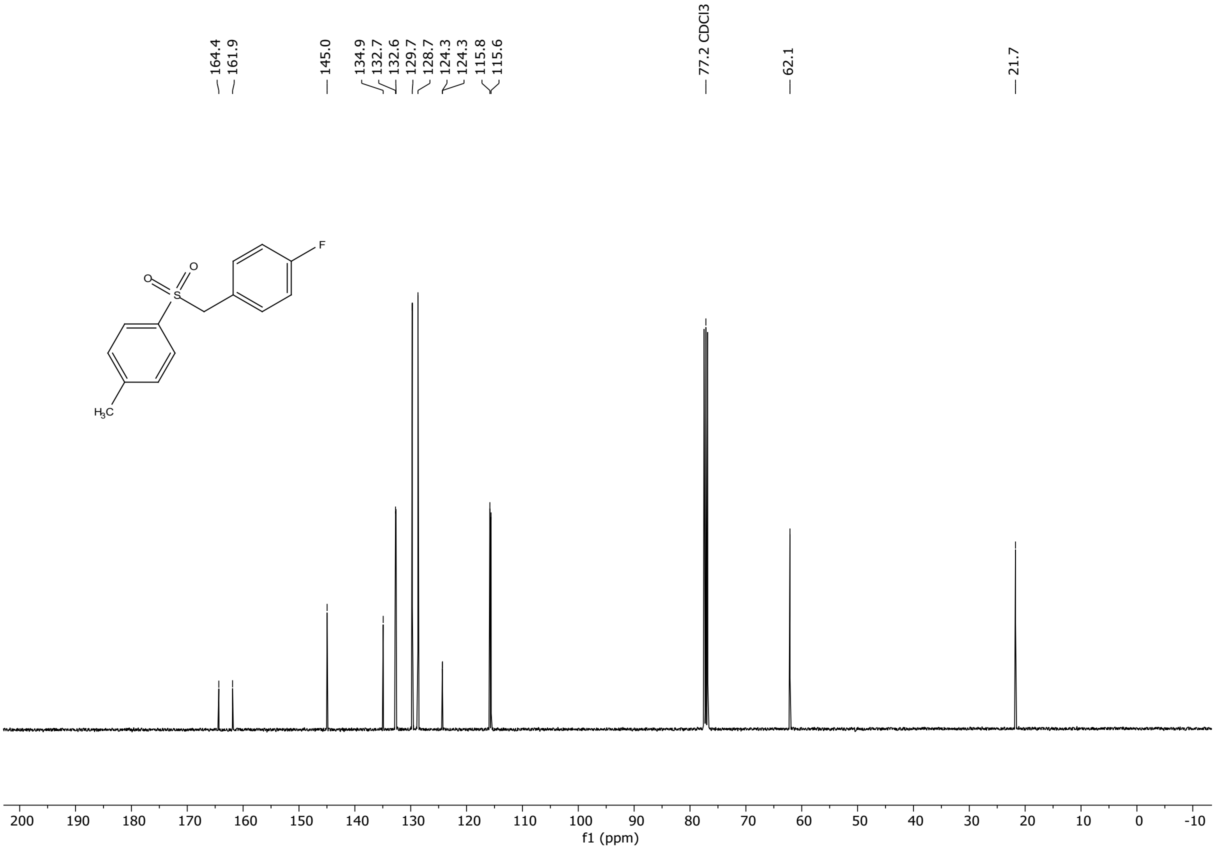

Supplement: SC-015-D4SC01940G-s001 [file SC-015-D4SC01940G-s001.pdf]
